# Revisiting the structure/function relationships of H/ACA(-like) RNAs: a unified model for Euryarchaea and Crenarchaea

**DOI:** 10.1093/nar/gkv756

**Published:** 2015-08-03

**Authors:** Claire Toffano-Nioche, Daniel Gautheret, Fabrice Leclerc

**Affiliations:** I2BC, Institute for Integrative Biology of the Cell, CEA, CNRS, Université Paris Sud, 1 avenue de la terrasse, 91198 Gif sur Yvette, France

## Abstract

A structural and functional classification of H/ACA and H/ACA-like motifs is obtained from the analysis of the H/ACA guide RNAs which have been identified previously in the genomes of Euryarchaea (Pyrococcus) and Crenarchaea (Pyrobaculum). A unified structure/function model is proposed based on the common structural determinants shared by H/ACA and H/ACA-like motifs in both Euryarchaea and Crenarchaea. Using a computational approach, structural and energetic rules for the guide:target RNA-RNA interactions are derived from structural and functional data on the H/ACA RNP particles. H/ACA(-like) motifs found in Pyrococcus are evaluated through the classification and their biological relevance is discussed. Extra-ribosomal targets found in both Pyrococcus and Pyrobaculum might support the hypothesis of a gene regulation mediated by H/ACA(-like) guide RNAs in archaea.

## INTRODUCTION

The H/ACA guide RNAs are part of a RNP machinery including several proteins (L7Ae, Cbf5, Nop10 and Gar1) which catalyzes the uridine-to-pseudouridine isomerization. Homologs of the eukaryotic snoRNA H/ACA, these guide RNAs are found in a widespread number of Archaea, in Euryarchaea (*Archaeoglobus fulgidus* (1), *Haloferax volcanii* (2), *Pyrococcus abyssi* (3–5)), in Crenarchaea (*Sulfolobus solfataricus* (6)) and in Nanoarchaea (*Nanoarchaeum equitans* (7)). Computational screens for H/ACA RNAs and their potential targets in the archaeal genomes suggest they are present in a variable number of copies among all the archaeal phyla (5,8). The natural targets are ribosomal RNAs but other RNAs might be targeted with functional implications (9,10). In eukaryotes, other targets include the U2 snRNA (11) which is modified in *S. cerevisiae* under particular conditions (12). Designed mRNAs including a nonsense codon were shown to be fully expressed when modified by the H/ACA guide RNP machinery at the first position of stop codons (13). On the other hand, snoRNAs have also been involved in unusual roles: in alternative splicing in the case of the C/D box guide RNA HBII-52 (14) or as one of the RNA biomarkers for non-small-cell lung cancer in the case of the H/ACA guide RNA snoRA42 (15).

**Figure 1.**
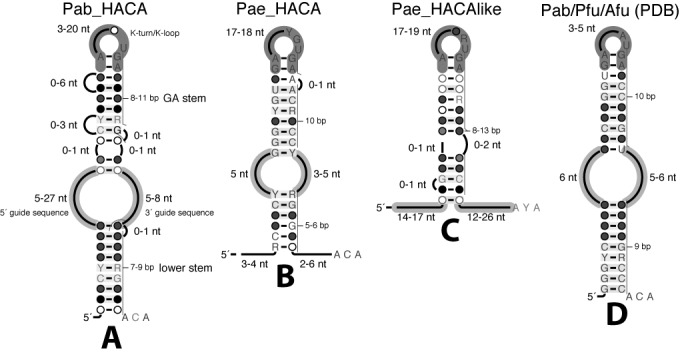
Structure/Function Models of H/ACA guide RNAs from structural and functional studies. **(A)** Consensus functional model of H/ACA motif in *Pyrococcus abyssi* (Supplementary Data: Figure S1 and Listing 1). **(B)** Consensus functional model of H/ACA motif in *Pyrobaculum aerophilum* (Supplementary Data: Figure S2 and Listing 2). **(C)** Consensus functional model of H/ACA-like motif in *Pyrobaculum aerophilum* (Supplementary Data: Figure S3 and Listing 3). **(D)** Consensus structural model of H/ACA motif for crystallized chimeric RNAs from *Pyrococcus furiosus* and *Archaeaglobus fulgidus* (Supplementary Data: Figure S4 and Listing 4). The consensus model for *Pyrococcus abyssi* was derived from the Stockholm alignment of all H/ACA motifs as previously folded (5) and represented using R2R (see Materials and Methods). The consensus models for *Pyrobaculum aerophilum* were derived from the Stockholm alignment of the two canonical H/ACA motifs (sR201 and sR202) or that of the other H/ACA-like motifs (sR203 to sR210) (16). The consensus structural model was derived from the alignment of the three guide RNAs included in the 3D structures of the full H/ACA RNP assemblies (L7Ae, Nop10, Cbf5 with or without Gar1) determined by X-ray crystallography (PDB IDs: 2HVY (17), 3HAX (18), 3LWO (19)).

Extensive structural and functional studies have focused on the H/ACA RNAs and RNPs from *Pyrococcus*: *P. abyssi* (4,5,20–25), *P. horikoshii* and *P. furiosus* (3,5,17–19,26–31) which is the only genus with so many genes for this class of sRNA (seven H/ACA genes corresponding to 11 H/ACA motifs). These studies describe the structure/function relationships for this RNA guide machinery regarding: the RNA fold, the RNA:RNA interactions between the guide and its target(s) and the RNA:protein contacts (between L7Ae and the K-turn or K-loop motif, between Cbf5 and the ACA box, etc.). The H/ACA motif is well described as a stem-loop-stem motif closed by an apical loop and terminated by an ACA box at the 3’ end (32) (Figure 1). In archaea, the L7Ae ribosomal protein is part of the H/ACA RNP particle and specifically binds to a K-turn motif which is embedded in the upper stem or merged within the apical loop as a K-loop. The lower stem includes between 7 and 9 base-pairs in the canonical H/ACA motifs and the distance between the ACA box and the base-pair closing the internal loop in the upper stem is reported to be between 14 and 16 nt (1) (3’ strands of the internal loop and lower stem); in the H/ACA motifs from *P. abyssi*, an empirical constraint between 14 and 15 nt was proposed (5) (Figure 1A). On the other hand, no well-defined constraint is proposed regarding the position of the K-turn or K-loop motif with respect to the internal loop. The distance from the base-pair closing the internal loop in the upper stem to the second G:A base-pair of the K-turn or K-loop motif, defined as ‘GA stem’ (Figure 1), spans from 8 to 11 base-pairs (5). In *Haloferax volcanii*, the length of the GA stem ranges from 8 (2) to 9 (1,20,33) and 10 base-pairs (1,20).

More recently, the discovery of ‘atypical’ H/ACA motifs in the *Pyrobaculum* genus has suggested an alternate way for this machinery to assemble and achieve its function (16). These non-canonical H/ACA motifs, further designated as H/ACA-like motifs, are detected specifically in this genus of Crenarchaea and differ from the canonical H/ACA motifs in that the lower stem is truncated. They generally include two long free single-stranded regions at both 5’ and 3’ ends forming a pseudo-internal loop and the generic hairpin with an embedded K-turn motif. Two more canonical H/ACA-like motifs are also found (sR201 and sR202). From a structural point of view, one may consider that sR201 and sR202 do correspond to canonical H/ACA motifs (Figure 1B): the motif sR201 was identified previously as a canonical H/ACA motif in *P. aerophylum* (23,34,35). These two particular guide RNAs exhibit a lower stem although slightly shorter (5–6 base pairs) than that of the canonical H/ACA motifs found in *Pyrococcus* (8–9 base pairs) while the other eight guide RNAs are fully truncated with no lower stem (Figure 1C). The GA stem includes exactly 10 base-pairs in both H/ACA motifs (Figure 1A,1B) but it is much more variable in the H/ACA-like motifs with 8 to 13 base-pairs (16) (Figure 1C). When searching for new H/ACA(-like) motifs in the archaeal genomes, an even looser constraint is generally used for the GA stem: between 5 and 12 base-pairs (5,8,23,33). In all the 3D structures of the full H/ACA RNP complexes (L7Ae, Nop10, Cbf5 and Gar1) in presence or not of a target RNA (PDB IDs: 2HVY, 3LWO, 3HAX) (17–19), the GA stem is 10 base-pairs long (Figure 1D): it orients the K-turn or K-loop motif within the RNP complex so that L7Ae binds the guide RNA and interacts with Nop10. There are striking differences between the structure/function models in *Pyrococcus* and *Pyrobaculum* which are representative of the fold diversity of H/ACA guide RNAs in other archaea (Figure 1). All the consensus models for natural H/ACA guide RNAs show pretty loose constraints in the length of the lower stem, GA stem and internal loop (Figure 1A–C) which contrast with the tight constraints for the consensus model derived from crystallized RNAs in H/ACA sRNP (Figure 1D). Unfortunately, there are only a few functional studies where a specific H/ACA guide RNA was shown to modify a specific rRNA or tRNA target. Full experimental validations are limited to a few organisms: *Archaeoglobus fulgidus* (1,20), *P. abyssi* (23) and *Haloferax volcanii* (2); only partial experimental validations are available for: *Pyrobaculum aerophilum* (16) and *Sulfolobus solfataricus* (6,24).

The present study is primarily focused on the canonical H/ACA motifs from *Pyrococcus abyssi* to identify the structural determinants associated with the function of pseudo-uridylation and defines a new structural and functional classification of those motifs. The additional data available for a limited number of other archaea mentioned above (*Archaeoglobus fulgidus*, *Haloferax volcanii* and *Sulfolobus solfataricus*) is used to check the validity of the proposed classification to discriminate between H/ACA guide:target duplexes which are productive versus non-productive (leading or not to the pseudo-uridylation of the target at the expected position, respectively). We will also examine whether the H/ACA(-like) motifs found in *Pyrobaculum* can fit into a unified structure/function model in spite of their structural specificities. From the structural point of view, we will ask whether the model can also be extended to the modification of tRNAs. From the functional point of view, we will address the question of the functional role played by the H/ACA-like motifs proposed recently in *P. abyssi* or that of other non-productive folds.

## MATERIALS AND METHODS

### RNA-seq data and annotations

The full RNA-seq data on *P. abyssi* are available from the work published recently (36) and from its supplementary material (http://rna.igmors.u-psud.fr/suppl_data/Pyro). Most of the sequences of the H/ACA motifs from *P. abyssi* are available in RFAM (37,38): Pab21 (RFAM ID: RF00065), Pab35 or HgcF (RFAM ID: RF00058), Pab40 or HgcG (RFAM ID: RF00064), Pab105 or HgcE (RFAM ID: RF00060), Pab19, Pa160 and Pab91 (5). In the particular case of Pab21, its location corresponds to a ncRNA annotated as a C/D box guide RNA (snoR9) at positions 230,449-575(-) in the genome in agreement with the transcriptome obtained recently (36). Actually, the H/ACA guide Pab21 is located at the 5’ end of the same transcript (230,517-575). Pab35 or HgcF (318,231-398) and Pab40 or HgcG (382,332-604) correspond to H/ACA guide RNAs identified previously (5) that are not explicitly reported as H/ACA guide RNAs. The transcribed RNAs slightly differ from the original annotations: Pab35 (318,227-410) is extended by a few nucleotides on both 5’ and 3’ ends while Pab40 (382,389-622) is shortened on its 5’ end (starting at the 1st position of the H/ACA motif) and extended on its 3’ end.

### Structural alignments, representations and analysis

The STOCKHOLM alignments obtained from RFAM and other sources were edited to split the RNA genes within multiple motifs (Pab35: Pab35.1, Pab35.2, Pab35.3; Pab105: Pab105.1, Pab105.2; Pab40: Pab40.1, Pab40.2) into their corresponding unique motifs. All the individual H/ACA motifs were then merged to generate a full alignment. For the sake of graphical representation, the motifs with a large internal loop, with a single strand region exceeding 10 nucleotides (Pab40.2 and Pab19), were omitted. The 2D structure representations were generated from the STOCKHOLM alignments using the R2R program (43). The structure representations of the guide:target RNA pairs are also generated using the R2R program. The corresponding pairing energies are calculated using the RNAsnoop utility (44) from the Vienna Package (version 2.0) (45). In the analysis of the energy contributions to the duplex stability, we refer to the 5’ and 3’ duplex as defined in RNAsnoop.

In the case of the H/ACA-like motifs from *Pyrobaculum* and other new H/ACA(-like) motifs (from *Pyrococcus*), the alignments were obtained from the UCSC genome browser in MAF format (41,42) and then converted into an aligned FASTA and STOCKHOLM formats. Finally, R2R (43) decorators were added to generate the 2D structure representations. The 2D representations of hybrid guide:target RNAs were generated using R2R with specific options and decorators; some detailed examples to reproduce this kind of representations are provided elsewhere (46). The structural analysis of the H/ACA sRNAs was performed using X3DNA (version 2.1) (47) and Curves+(48).

### Computational RNomics

**Figure 2.**
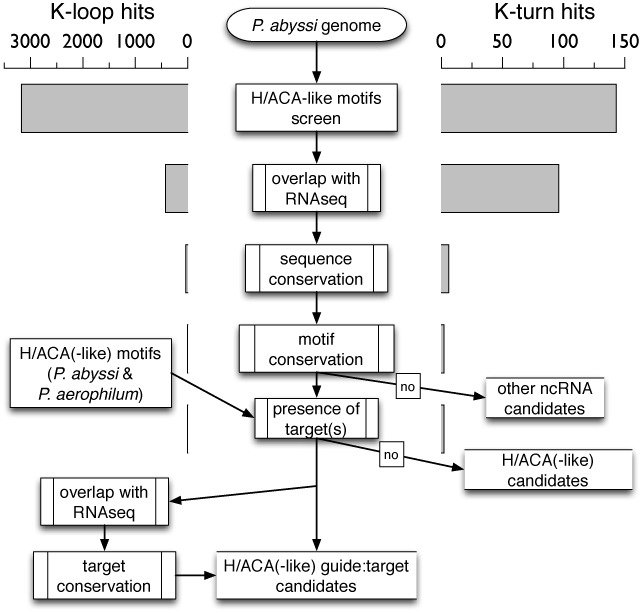
Workflow for the identification of H/ACA(-like) motifs and potential targets in *Pyrococcus abyssi* and *Pyrobaculum aerophilum*. The genome is initially screened for the presence of H/ACA-like motifs using a descriptor-based approach (RNAMotif (39)). The number of hits is given at each step of the workflow as an indicative value for the motifs carrying either a K-turn or K-loop submotif. The first filter is the selection of motifs in the non-coding transcriptome (S-MART scripts (36,40)), then the selection of conserved sequences and motifs among other archaea (UCSC archaea genome browser (41,42)) and finally the selection of motifs that have potential target(s) in the genome which are themselves transcribed and conserved among other archaea.

A multi-step workflow is used to search for possible H/ACA-like motifs in the genome of *P. abyssi* (49,50) (Figure 2). The H/ACA-like motifs are identified using the descriptor-based approach implemented in RNAMotif (39). Based on the 2D structure of the H/ACA(-like) motifs identified in *Pyrobaculum* (16), six families are defined to consider all possible candidates with different stem lengths and bulge positions (Supplementary Material: Listings 5–16). The two canonical H/ACA motifs *Pae* sR201 & sR202 are defined using a unique descriptor. The following sRNAs: *Pae* sR204, *Pae* sR208, *Pae* sR209, *Pae* sR210 are also defined by a common descriptor with a bulge nucleotide one base pair downstream (3’ end) from the two GA pairs of the K-turn motif. Each of the other sRNAs (*Pae* sR203, *Pae* sR205, *Pae* sR206, *Pae* sR207) is defined by a unique descriptor. The substitution between K-turn and K-loop motifs is allowed: two descriptors are used for each of the six families of sRNAs. All the descriptors are provided (as text files) in the supplementary material.

A series of S-MART scripts (40) is used to extract the H/ACA(-like) motifs that lie in the non-coding transcriptome of *P. abyssi*. A minimum overlap of 50 nucleotides is required to filter out the initial hits. The sequence and motif conservation are verified using the UCSC archaeal genome browser (41,42). A combination of two softwares: locaRNA (51) and RNAalifold (52) are used to identify optimal and sub-optimal 2D structures which are consistent with the H/ACA(-like) fold. The search for associated targets is performed using the RIsearch program (53), using the recommended extension penalty (‘-d 30’). The potential RNA targets are identified by searching through the whole genome matching sequences that can associate with a pseudo-guide sequence containing the 5’ and 3’ duplex elements from the internal loop of the guide RNA. This pseudo-guide sequence is obtained by merging the 5’ and 3’ duplex elements from the internal loop of the guide RNA separated by a di-nucleotide spacer equivalent to the di-nucleotide spacer containing the targeted U position. In order to impose a 5’-UN-3’ sequence constraint in the target RNAs, the di-nucleotide spacers in the pseudo-guide sequences are defined by the complementary element: 5’-NA-3’. The target RNA candidates are selected based upon a RIsearch energy score of −16 kcal/mol or lower which corresponds to the less favorable energy score obtained for validated guide-pair targets (with a deviation of 1 kcal/mol). Only target sequences with at most two bulge nucleotides are tolerated but no bulge nucleotide is allowed on the 5’ duplex (5’ end of the paired target sequence) upstream from the predicted modified position.

After a guide:target pair is identified, two additional filters are applied. The first filter is the presence of the target sequence in a region of the genome of *P. abyssi* which is expressed (36). The second filter is the conservation of the target in both *P. abyssi* and *P. aerophilum* when it is found: (a) in homologous genes identified using the BBH approach (54,55) (Blast queries (56): E-value < 1e-4) or (b) in the same gene family (e.g. tRNA synthetases).

## RESULTS

### Pyrococcus abyssi as a model for H/ACA guide RNAs

In archaea, the larger existing set of structural and functional data includes 23 H/ACA guide:target pairs that were tested in *P. abyssi* on 21 potential pseudo-uridylated positions in the 16S and 23S rRNA: the 11 H/ACA RNA motifs may potentially target several positions. Conversely, one given position may happen to be a potential target for two different H/ACA guide RNAs: this is the case for two targeted positions: S922 in the 16S rRNA and L2672 in the 23S rRNA (5) (Supplementary Figure S1). Among the 23 experimentally tested guide:target pairs, there are 15 ‘productive’ RNA:RNA duplexes uniquely associated with 15 ‘true targets’ validated experimentally *in vitro* (Figure S1(a)). On the other hand, there are 8 ‘non-productive’ RNA:RNA duplexes associated with 8 targets: 6 ‘false targets’ which are not modified positions and 2 redundant ‘true targets’ which are modified by two productive RNA:RNA duplexes (Supplementary Figure S1(b)). This small set of data from a unique source is used, in this work, as a learning set to derive some structural, energetic and pairing rules based on a detailed analysis of discriminant features (consistent with the phylogenetic data) that determine whether a H/ACA guide:target duplex is productive or non-productive.

**Figure 3.**
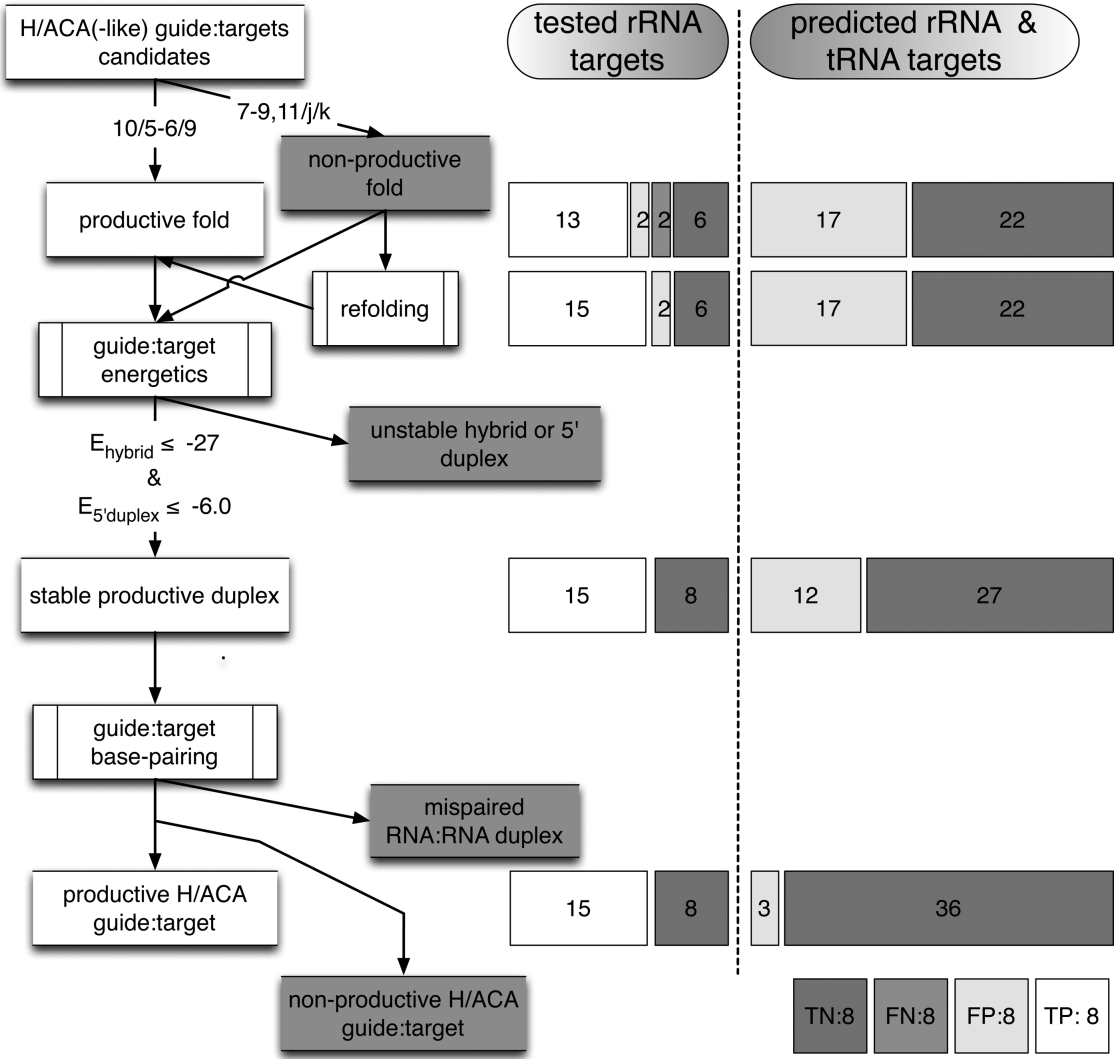
Workflow for the classification of productive/non-productive H/ACA guide:target complexes. The tested rRNA targets refer to the 23 H/ACA guide:target complexes in *P. abyssi* validated experimentally (5) (Table 1). The two false negative hits were removed after refolding a non-productive fold to an alternative productive fold (Pa105.1-L2554 and Pa19-S1017). Seven false positive hits were removed based on the energy cutoff value for the 5’ duplex stability (2 hits: Pa35.2-L2250 and Pa105.2-S995 in the tested targets and 5 hits in the predicted targets). The full set of rRNA and tRNA targets (tested and predicted) include alternative potential rRNA or tRNA positions targeted by the original productive and non-productive folds plus alternative folds generated from the original ones by the modification of the length of the GA stem to 8, 9 or 11 base-pairs; the new guide:pair candidates with a potential rRNA or tRNA target are indicated in Table S1. There are 3 remaining false positive hits which correspond to tRNA positions that are not expected to be modified. True positive (TP): productive guide:pair duplex classified as productive; False positive (FP): non-productive guide:pair classified as productive; False negative (FN): productive guide:pair duplex classified as non-productive; True negative (TN): non-productive guide:pair duplex classified as non-productive.

A genome-wide search for alternative RNA targets based on sequence matching and energetic rules provides new targets distributed either in the RNA genes coding rRNA or tRNA or in other regions of the genome such as CDS (Figure 3). The pseudo-uridylations are modifications which are well characterized in the rRNAs and tRNAs from *P. abyssi*. Thus, additional targets in these two classes of RNA can be mostly considered as false positive hits and be used as a test set. The additional data available for a limited number of other archaea mentioned above (*Archaeoglobus fulgidus*, *Haloferax volcanii* and *Sulfolobus solfataricus*) will also allow us to check the validity of the structural and functional classification for productive/non-productive H/ACA guide:target duplexes.

### Productive/non-productive H/ACA guide RNAs

The attempt to identify the structural determinants specific to the productive and non-productive H/ACA guide RNAs is based on two initial assumptions about the respective length of the GA and lower stems corresponding to the more probable structure/function model. Although there are some structural variabilities in the H/ACA motifs from *P. abyssi* as shown in the consensus model (Figure 1A), 14 out of 15 productive H/ACA folds exhibit a 10 base-pairs GA stem consistent with the 3D structural data (Figure 1D and Supplementary Figure S4) while 7 out of 8 non-productive folds exhibit 8, 9 or 11 base-pairs (Supplementary Figure S1). The lower stem includes 9 base-pairs in 13 out of the 23 H/ACA folds; it is truncated by one or two base-pairs in the other cases but the distance between the ACA box and the first base-pair from the GA stem closing the internal loop is between 14 and 16 nucleotides and only between 14 and 15 for the productive folds. For the sake of classification, a structural notation is created to refer to the fold type which is identified by a three-number code: ‘i/j/k’ where i, j and k correspond to the lengths of the GA stem, 3’ guide sequence and lower stem, respectively (Figure 1A). For example, the crystallized H/ACA RNAs have two possible folds: ‘10/5/9’ and ‘10/6/9’ which are considered as two main families (Figure 1D).

The set of 23 H/ACA guide:target complexes correspond to the 15 productive (Supplementary Figure S1(a)) and eight non-productive guide:target duplexes (Supplementary Figure S1(b)) tested experimentally in *P. abyssi*. They are annotated with the name of the H/ACA motif (e.g. Pa91) and that of the associated target in the large (e.g. L2685) or small (e.g. S27) ribosomal subunit: P91-L2685 refers to the RNA fold targeting position 2685 in 23S rRNA. All these complexes can be properly classified using a set of three rules including: (a) the fold type, (b) the calculated energies of RNA:RNA duplexes for both the full hybrid duplex and the 5’ hybrid duplex (pairing between the 3’ guide sequence of the guide RNA with the 5’ region of the target RNA upstream from the potential modified U position) and (c) the base pairing in the duplex. The workflow based on these rules is described in details in Figure 3: the 15 productive folds and 8 non-productive folds are properly re-classified as true positive and true negative hits. The full list is provided in Table 1 which especially includes the fold notation and the calculated energies. The classification is then tested using a range of alternative folds for each guide RNA and its associated potential targets in rRNAs and tRNAs.

**Table 1.** Functional and structural features from known H/ACA guide RNAs in *Pyrococcus* targeting 16S and 23S rRNAs

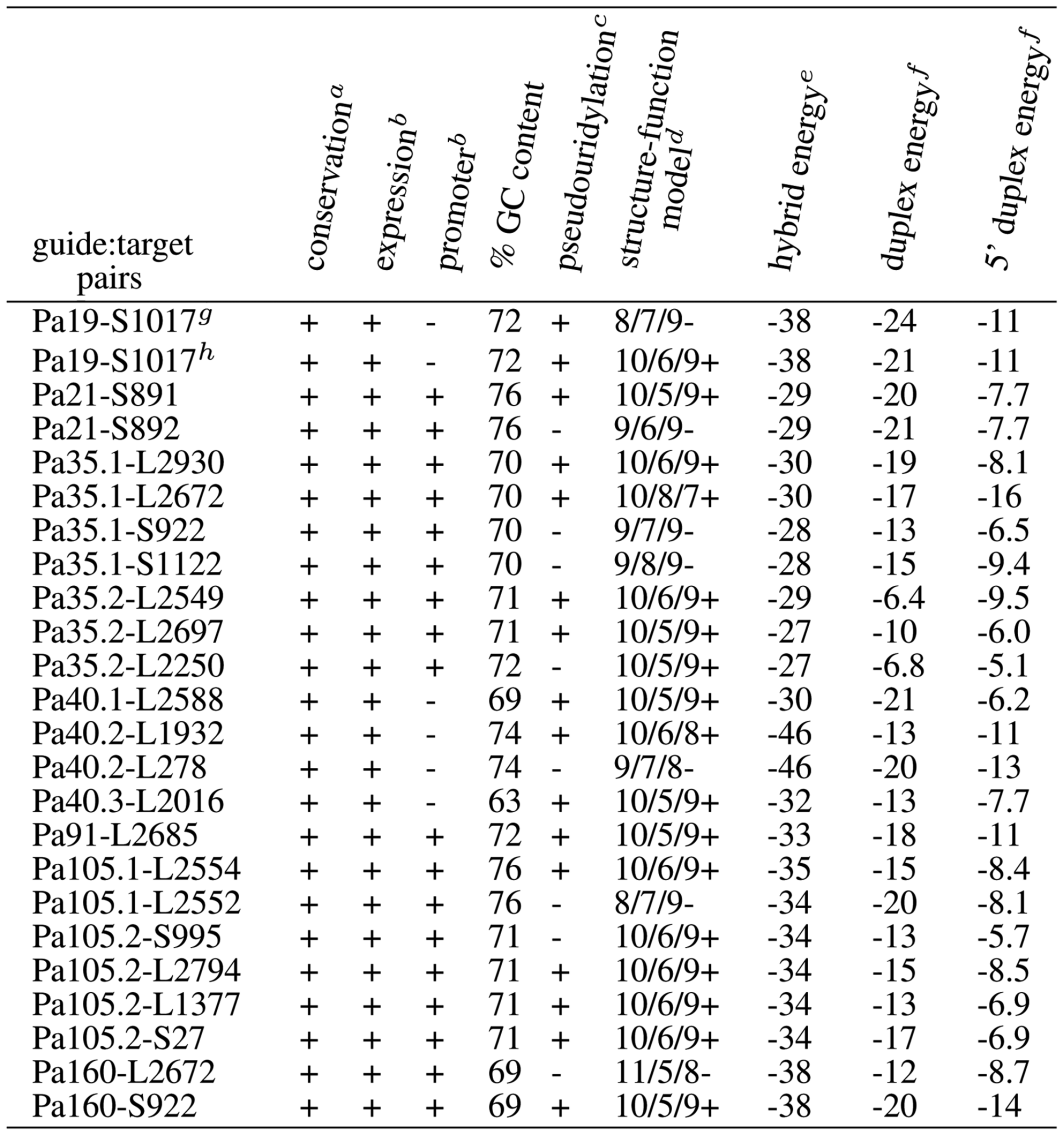									

^*a*^as provided by the UCSC genome browser (41,42).

^*b*^as determined from RNA-seq data (36).

^*c*^as determined from previous work by RT-CMCT analysis and *in vitro* activity of pseudouridylation (5).

^*d*^structure-function model as proposed from the ‘productive’/‘non-productive’ classification (the ± sign indicates whether it is predicted to be productive or not); the listed models also include some additional models that optimize the hybrid and duplex energies: 10/6/8 (Pa40.2-L1932) and 10/8/7 (Pa35.1-L2672) which are derived from 10/5/9 and 10/6/9 by opening the lower stem by one or two base-pairs (Supplementary Figure S7), respectively.

^*e*^as calculated by RNAsnoop (kcal/mol) from the Vienna RNA package (44).

^*f*^the duplex energy includes both the 5’ and 3’ duplex energies with a correction factor (+4.1 kcal/mol).

^*g*^the Pab19-S1017 pairing proposed previously is based on a 8/7/9 model (5).

^*h*^refolded based on a 10/6/9 model.

**Figure 4.**
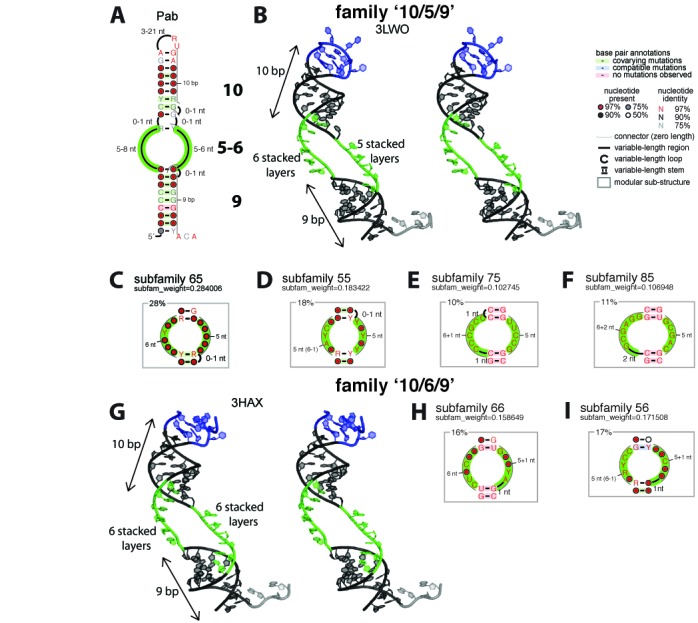
Structure/Function Model of H/ACA guide RNAs in *Pyrococcus abyssi*: ‘10/5/9’ and ‘10/6/9’ fold families. **(A)** The consensus 2D structure is obtained from a curated STOCKHOLM alignment modified from the RFAM entries for H/ACA guide RNAs in P. abyssi (RFAM (37,38) IDs: RF00058, RF00060, RF00064, RF00065). **(B)** 3D structure of the chimeric guide RNA from *Pyrococcus furiosus* is shown in a stereo view as it is in the H/ACA sRNP complex including its RNA target (PDB ID: 3LWO). **(C)** Subfamily 65 (28%): 6 and 5 nucleotides in the 5’ and 3’ guide sequences of the internal loop, respectively. **(D)** Subfamily 55 subfamily (18%). **(E)** Subfamily 75 (10%). **(F)** Subfamily 85 (11%). **(G)** 3D structure of the chimeric guide RNA derived from Afu46 (from *Archaeoglobus fulgidus* from the ‘10/6/9’ family; only the RNA component of H/ACA RNP complex (PDB ID: 3HAX) is shown in the stereoview. **(H)** Subfamily 66 (16%). **(I)** Subfamily 56 (17%). The Stockholm alignment is provided in the supplementary material (Listing 17). The graphical representations of the full 2D structures and the internal loop subfamilies were generated using R2R (see Materials and Methods). The internal loop of the H/ACA motif is shaded in green, the K-turn or K-loop motifs in blue and the ANA motif in grey.

The productive folds are classified in the two fold families ‘10/5/9’ and ‘10/6/9’ (Figure 4 and Supplementary Listing 17). The ‘10/5/9’ fold family is consistent with the 3D structure of a chimeral H/ACA RNA from *P. furiosus* (3D: Figure 4B; 2D: Supplementary Figure S4) while the ‘10/6/9’ fold family is consistent with the 3D structure of another chimeral H/ACA RNA (3D: Figure 4G; 2D: Supplementary Figure S4) derived from *Archaeoglobus fulgidus* (Afu46) which is assembled with the H/ACA ribonucleoproteins from *P. furiosus* (PDB ID: 3HAX) (17). In the ‘10/6/9’ fold family, the internal loop is extended by the addition of one nucleotide in the 3’ guide sequence which folds with 6 stacked nucleotides (Figure 4G–I). As a result, the number of stacked layers from the ANA box to the closing base-pair of the GA stem differs in the two fold families ‘10/5/9’ and ‘10/6/9’ with 14 and 15 nucleotides, respectively. Different structural subfamilies can then be distinguished based on the size of the 5’ guide sequence of the internal loop, e.g. the subfamily 65 corresponds to an asymmetric internal loop with 6 and 5 residues, respectively. It corresponds to the more represented subfamily (28%) with 4 H/ACA guide RNAs (Figure 4C and Supplementary Figure S5). In the ‘10/5/9’ family, there are three other subfamilies which are variations in the size of the internal loop which is either shortened or extended by one or two nucleotides on either sides (subfamilies: 55, 75 and 85: Figure 4D–F). The ‘10/6/9’ family includes only two subfamilies where the internal loop may be symmetrical (subfamily 66: Figure 4H) or asymmetrical (subfamily 56: Figure 4I). Overall, we can define two major families and six subfamilies corresponding to variations in the size of internal loop. The details of each productive fold in the different families and subfamilies are given in the Supplementary Material (Supplementary Figure S5).

In the set of 23 guide:target pairs, any change (addition or deletion of one base-pair or more) in the length of the GA stem is deleterious for the pseudo-uridylation activity (Supplementary Figure S6). On the opposite, mutual compensations in the length of the 5’ guide sequence and lower stem preserve the activity while a continuous stretch of 14 or 15 nucleotides is maintained between the ACA box and the first base-pair from the GA stem (Supplementary Figure S7).

### Refolding non-productive guide RNAs

The two false negative hits correspond to Pa105.1-L2554 and Pa19-S1017 (Figure 3) which can be refolfed and fit into the ‘10/6/9’ family. In the case of Pa105.1-L2554, the initial model was based on a ‘9/7/8’ fold type (Supplementary Figure S1). The new fold type proposed here involves a A bulged-in nucleotide (Figure S5) which is consistent with the phylogenetic data on *Thermococcus* where the bulge nucleotide is substituted by a wobble base-pair (Supplementary Figure S8).

**Figure 5.**
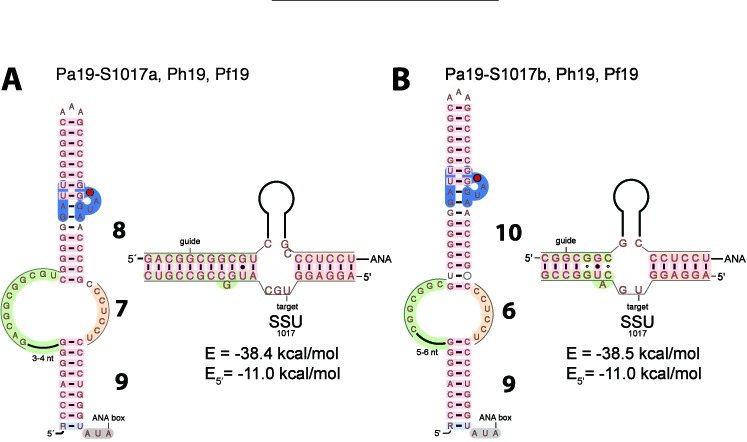
Models of guide:target pairs of Pa19. **(A)** Non-productive Pa19-S1017a model proposed previously (‘8/7/9’) (5). **(B)** New productive Pa19-S1017b model (‘10/6/9’).

In the case of Pa19-S1017, a non-productive fold with only 8 base-pairs in the GA stem, 16 nucleotides between the ANA box and the GA stem and a 3 nucleotide target spacer (between the 5’ and 3’ regions of the target matching the 3’ and 5’ guide sequences, respectively) was proposed previously (5) (Figure 5A and Supplementary Listing 18). The alternate productive fold is similar to that proposed for Pa105.1-L2554a (Figure 5B and Supplementary Listing 19). The bulge nucleotide is a U which may not stack in the GA stem but rather loop out to the minor groove and still induce a compensatory twist in the stem as shown in some known RNA structures (57). In both cases, the GA stem includes three stacked G-A base-pairs at positions 30-33:55-57 in Pa19-S1017 (Figure 5) and 22-24:48-50 in Pa105.1-L2554 (Supplementary Figure S5) which probably contribute to the accommodation of the K-turn motif in the appropriate position and orientation in the RNP particle by adjusting the helical twist. The energetics of the guide:target duplex is very similar for both folds (see next section and Table 1).

Alternative productive or non-productive folds were generated for all the H/ACA motifs by varying the length of the GA stem from 8 to 11 base-pairs in agreement with the proposed range in the previous models (Figure 1A). In total, 62 guide:target pairs were obtained (predicted rRNA and tRNA targets, Figure 3) corresponding to the 23 original complexes mentioned above (tested rRNA targets) and 39 additional pairs (predicted rRNA and tRNA targets) associated with false targets in rRNAs and tRNAs (Table S1). Among the new guide:target pairs, 22 candidates are expected to be non-productive folds while 17 are expected to be productive folds. The refolding of Pa19-S1017 and Pa105.1-L2554 eliminated the two false negative hits at this stage and led to a distribution with: 15 true positive hits (tested targets), 19 false positive (2 tested targets and 17 predicted targets) and 28 true negative hits (6 tested targets and 22 predicted targets).

### Energetics of guide:target complexes

**Figure 6.**
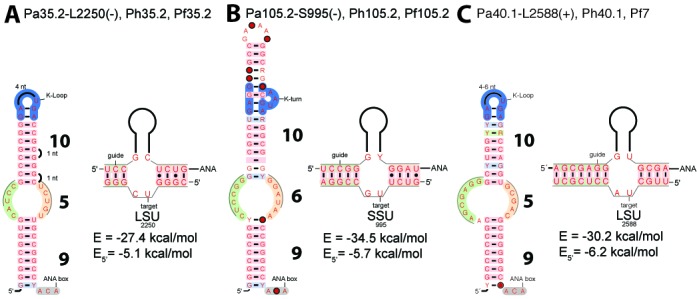
Models of productive (+) and non-productive (-) guide:target pairs Pa35.2, Pa105.2 and Pa40.1. **(A)** Productive fold for Pa35.2 and its false target L2250. **(B)** Productive fold for Pa105.2 and its false target S995. **(C)** Productive fold for Pa40.1 and its true target L2588. The duplexes are represented with the following color code: orange for the 5’ duplex, green for the 3’ duplex. The energy values are those calculated by RNAsnoop. Each RNA fold is annotated with the names of the H/ACA motif and its associated target in the large (L) or small (S) ribosomal subunit indicated by the position which is modified in the rRNAs.

In the data set including the tested rRNA targets (Figure 3), the two false positive hits correspond to: Pa35.2-L2250 and Pa105.2-S995 which both adopt a productive fold from the two main families: ‘10/5/9’ and ‘10/6/9’, respectively. However, the energetics of the RNA:RNA duplex indicates that these two guide:target complexes have a particularly weak 5’ duplex including 2 wobble base-pairs. The comparison of three guide:target complexes with similar energies for the 5’ duplex suggests a cutoff value of −6.0 kcal/mol is discriminant. The two non-productive guide:target complexes: Pa35.2-L2250 (Figure 6A) and Pa105.2-S995 (Figure 6B) exhibit a 5’ duplex energy above −6.0 kcal/mol (−5.1 and −5.7 kcal/mol, respectively) while the productive guide:target complex Pa40.1-L2588 (Figure 6C) exhibit a slightly more favorable energy (−6.2 kcal/mol). On the other hand, the 3’ duplex energy does not appear to be a discriminant criterion: the 3’ duplex is reduced to a single base-pair in the case of Pa35.2-L2549 (3’ duplex energy of −1 kcal/mol) but the 5’ duplex energy (−9.5 kcal/mol) is below the cutoff value (Supplementary Figure S9). In the set of experimentally tested targets, all the 23 guide:target pairs are correctly re-classified as 15 true positive and 8 true negative hits based on the fold type and stability of the RNA:RNA complex (−27 kcal/mol) and 5’ duplex (−6.0 kcal/mol). In the complementary data set including predicted rRNA and tRNA targets (Supplementary Table S1 and Figure 3), 5 additional false positive hits were further removed and re-classified as true negative hits based on the energy cutoff for the 5’ duplex ending up with a total of 12 remaining false positive hits and 35 true negative hits (8 tested targets and 27 predicted targets).

### Base-pairing in guide:target hybrid duplexes

In the productive guide:target complexes, there is no occurrence of mismatch or more than one wobble base-pair especially in the 5’ duplex. The presence of wobble base-pairs in the 5’ duplex tends to destabilize the RNA:RNA hybrid duplex (Figure 6) and no more than one wobble is tolerated (Supplementary Figure S9B). Based on theses observations, we set an additional constraint to exclude candidates carrying base-pairing anomalies located close to the pseudo-uridylation site in the H/ACA RNP. They include especially the presence of mismatches or wobble base-pairs at the last position of the 5’ duplex (n-1) or at the first position of the 3’ duplex (n+2) which are expected to perturb the kinetics of association/dissociation of the guide:pair complex at the targeted U-position. One particular anomaly corresponds to the presence of a 3 nucleotides spacer between the 5’ and 3’ duplex elements of the target, a feature which was proposed in a previous model for the Pa19-S1017 guide:target complex (5) (Figure 5A). This feature is present in two guide:targets pairs (Pa19-L655 and Pa160-L1318; see Table S1) associated with potential targeted positions which are not modified in the 23S rRNA of *P. abyssi*. As shown experimentally, the presence of a third unpaired residue next to the targeted position is unfavorable for the pseudo-uridylation activity (31). Thus, this feature is rather considered an anomaly. The full list of base-pairing anomalies next to the targeted U position is detailed in the supplementary material (Table S1). In the predicted targets, 9 false positive hits are re-classified as true negatives (Figure 3) based on the described base-pairing anomalies. Only 3 false positive hits remain corresponding to tRNA targets: Pa19-tR8(TAG), Pa19-tR8(CAG) and Pa105.2-tR27(GGG) (Table S1).

### Validation in other Euryarchaea

The H/ACA guide genes which are present in *Thermococcus* genomes, in particular in *T. kodakaerensis*, are all orthologs of *Pyrococcus* genes as described previously (5). Only minor sequence variations are observed between *P. abyssi* and *T. kodakaerensis*; the major variation is the substitution in Pa105.1 of the A bulge at the bottom of the GA stem by a Watson-Crick base-pair which restores a more canonical stem (Supplementary Figure S8). In *Archaeoglobus fulgidus*, all the validated H/ACA guide RNAs (five motifs) fit in the subfamilies described previously in *Pyrococcus*: 55, 56, 65, 66 and 75 (Supplementary Figure S10 and Supplementary Listing 20). The H/ACA folds are compatible with productive guide RNAs where Af190, Af4.1, Af4.2b and Af46 can associate with their rRNA targets to allow the pseudo-uridylation at the respective positions: S1004, S1167, L2601 and L2639 as shown by Tang *et al*. (1). It was later suggested that Af52 is not functional for pseudo-uridylation especially because it does not carry any K-turn or K-loop motif (20). Thus, Af52 is not expected to modify the position L2878 proposed initially (1,20). Af4.3 was also proposed to guide the pseudo-uridylation at L1364 but the RNA fold that would target this position (only 9 base-pairs in the GA stem when opening the basal G-U base-pair: see Af4.3 in Supplementary Figure S10) is not consistent with a productive complex according to our classification. On the other hand, Af4.3 can still guide the modification of L1970 (23); it is closely related to Pa40.3 and its phylogenetically related motifs in *P. horikoshii* (Ph40.3) and *P. furiosus* (Pf7.3) which target the position L2016. Another related H/ACA motif is also found in *Methanocaldococcus jannaschii* where it can guide the modification at the equivalent position L2015 (Supplementary Figure S11).

In phylogenetically more distant Euryarchaea such as: *Haloferax volcanii*, *Haloarcula marismortui*, *Halobacterium salinarum*, *Haloquadratum walsbyi* and others, H/ACA RNA guides have also been identified (2,33). In the case of *H. volcanii* and the related species mentioned above, the RNA fold proposed based on the association between the guide RNA and its target(s) corresponds to the ‘10/5/9’ fold for the first H/ACA motif (Hvo1 targeting L2621) or the ‘9/7/9’ and ‘11/8/9’ folds for the second motif (Hvo2 targeting L1956 and L1958, respectively) (33). The two latter motifs can be reclassified into a productive fold. A slightly modified ‘10/5/9’ fold is supported by multiple sequence-structure alignment for Hvo1 (Supplementary Figure S12). In the case of Hvo2, a productive fold ‘10/6/9’ can be obtained by extending the GA stem and including an third unpaired residue between the 5’ and 3’ paired sequences of the target as proposed in a previous model of association (5) (Figure 5A); the resulting ‘10/6/9’ fold is annotated Hvo2a (Supplementary Figure S13). The ‘11/8/9’ alternative fold for Hvo2 includes more than five mismatches in the GA stem to accommodate the target sequence (33). However, a slight change in the pairing of the GA stem and positions of bulge nucleotides both in the guide and target RNAs can make it switch to an alternative ‘10/6/9’ fold suited for the pseudo-uridylation of L1958 (Hvo2b, see Supplementary Figure S14). Hvo1 and Hvo2a/Hvo2b can be folded according to ‘10/5/9’ and ‘10/6/9’ models to form the following hybrid RNA duplexes: Hvo1-L2621 (Supplementary Figure S15), Hvo2a-L1956 (Supplementary Figure S16) and Hvo2b-L1958 (Supplementary Figure S17).

**Figure 7.**
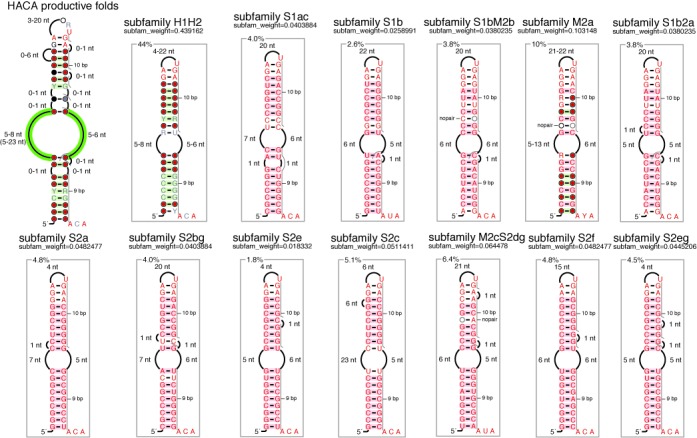
Consensus H/ACA Motif and Descriptor for productive folds as identified in: *P. abyssi*, *A. fulgidus* and *H. volcanii*. The 13 subfamilies are annotated by referring to the presence of unpaired nucleotides whether they are bulged in (M and ‘nopair’ annotation) or bulged out (S) and located in the lower stem (stem 1) or in the GA stem (stem 2): e.g. S1ac indicates the presence of two bulged out nucleotides (a and c) in stem 1 while S1bM2b involves both a bulged out nucleotide in stem 1 (S1b) and a bulged in nucleotide in stem 2 (M2b). The 3’ guide sequence is usually between 5 and 8 nucleotides except in two particular cases: Pa19 (12nt) and Pa40.2 (23nt). RNAMotif descriptors are defined for each subfamily where the internal loop is defined in a generic way with 5–8 nucleotides for the 5’ guide sequence and 5–6 nucleotides for the 3’ guide sequence (Listings 22–24).

Altogether, these results indicate that all the known H/ACA motifs from *Archaeoglobus* and *Haloferax* which have been experimentally validated also fit into the same classification proposed for *Pyrococcus*. All the knowledge on the productive H/ACA folds from these three genus is summarized in a general consensus structure (Figure 7) obtained from a global alignment of the productive folds in each representative species: *P. abyssi*, *A. fulgidus* and *H. volcanii* (Supplementary Figure S18 and Supplementary Listing 21). The consensus motif includes structural variations associated with the six subfamilies already described (Figure 4C–F, H–I) and the presence of bulge nucleotides at different positions in the two stems. Taking into account only the bulge positions, we can define 13 different subfamilies (Figure 7) that cover all the known cases of productive folds. A motif descriptor associated with each subfamily is provided where the internal loop is defined in a generic way: 5–8 nucleotides in the 5’ guide sequence and 5–6 nucleotides in the 3’ guide sequence (Listings 22–24). In *Haloferax volcanii* for example, the search for productive H/ACA motifs based on the 13 descriptors allowed us to find 106 new motifs in the intergenic regions among which less than 30% have a sequence conservation with a closely related species (data not shown).

### Validity for tRNAs substrates

In the Euryarchaea *M. jannaschii* and *P. furiosus*, differential roles were identified for Cbf5 (TruB/Pus4 family) corresponding to two distinct pseudo-uridylation pathways: a guide RNA-dependent pathway for rRNA modification and a guide RNA-independent pathway for the U55 sequence-specific tRNA modification (28,30,58). In the general case of tRNA modifications, the canonical pseudo-uridylation pathway is based on other Ψ-synthases (PsuX/Pus10 family). In sulfolobales and other Crenarchaea (such as: *Aeropyrum pernix* and *Metalloshaera sedula*), a guide RNA-independent pathway based on an additional Ψ-synthase (TruD/Pus7 family) also operates on tRNAs at position U35 of tRNA$^{Tyr}_{GUA}$. In sulfolobales and related species, this Ψ35-synthase is deficient but rescued through the guide RNA-dependent pathway (24). The U35 position can be potentially modified through the guide RNA-dependent pathway in five species where a compatible H/ACA guide RNA was identified: *Sulfolobus solfataricus*, *Sulfolobus tokodaii*, *Sulfolobus acidocaldarius*, *Aeropyrum pernix*, *Metallosphaera sedula* (24). This guide RNA-dependent pathway was validated experimentally for *Sulfolobus solfataricus*: the H/ACA RNA guide Sso1 corresponds to a ‘10/5/9’ fold which is also found in *A. pernix* (Supplementary Figure S19). In spite of the specificities associated with tRNA as targets of the H/ACA RNP machinery (24,58), the classification seems to be also valid for this class of RNA substrates.

### Relevance of the classification in Pyrobaculum

**Figure 8.**
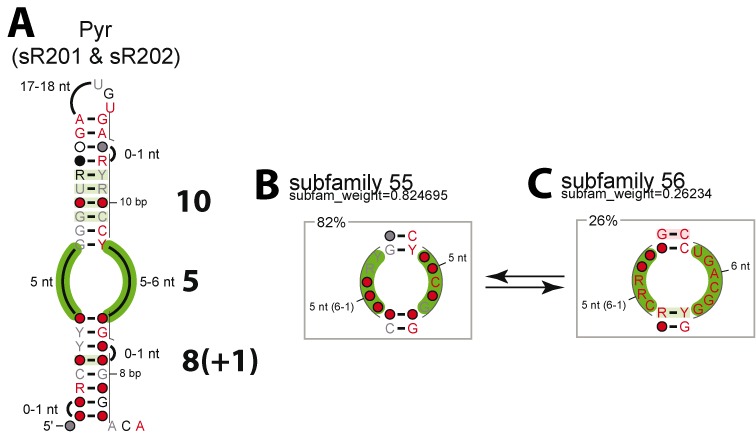
Canonical H/ACA Motifs in *Pyrobaculum* (Pae_sR201 & Pae_sR202). **(A)** Consensus structure of the H/ACA motifs. **(B)** Structural subfamily 55. **(C)** Structural subfamily 56. The two subfamilies apply to alternative folds for the same H/ACA motif in some specific *Pyrobaculum* species (Supplementary Figure S20).

From the data provided on the H/ACA-like guide RNAs in *Pyrobaculum* (16), we propose a structural classification which separates the ten sRNA (from Pae_sR201 to Pae_sR210) into three main families which differ by how close they are from the canonical H/ACA motifs found in other archaea, especially in *Pyrococcus*. The first family includes sR201 and sR202 which can be considered as canonical H/ACA motifs (‘10/5/8’ or ‘10/5/8(+1)’ fold), as shown in Figure 8: the two subfamilies 55 and 56 are already known in the motifs from *Pyrococcus* (Figure 4, Supplementary Listing 35). However, only the sRNA from the subfamily 55 (or equivalent) are expected to be productive. All the H/ACA motifs from the subfamily 55 exhibit 10 base-pairs between the internal loop and the K-turn motif. On the other hand, the motifs from the subfamily 56 only includes 9 base-pairs; the only two members of this subfamily are from *P. calidifontis* and *P. arsenaticum* (sR202). These two sRNA may switch from the subfamily 56 to the subfamily 55 by shortening the internal loop by one nucleotide which is then included in the GA stem (Supplementary Figure S20). This leads to increase the number of stacked layers from 9 to 10 to restore a productive configuration. The minor variation with respect to the canonical H/ACA motif is the length of the lower stem: 8 instead of 9 base-pairs (Supplementary Figure S21). We can assume that the presence of bulge(s) and/or mismatch(es) in the lower stem allows the ANA box to be accommodated in a similar way into the H/ACA RNP particle. Besides, the ANA box is well conserved in all the motifs sR201 and sR202 from the different species of *Pyrobaculum*. A single variation is observed in *P. calidifontis* where the ANA box is replaced by a CCA box reminiscent to the 3’ end of tRNAs.

**Figure 9.**
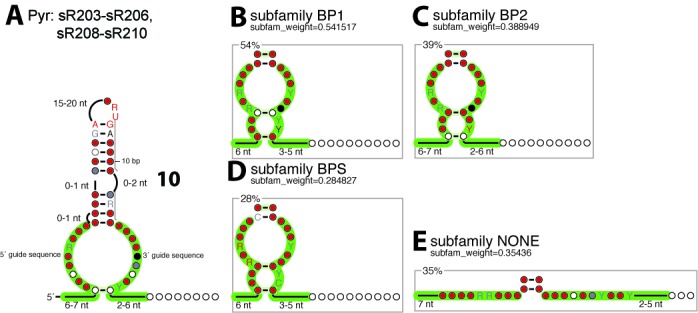
H/ACA-like Motifs in *Pyrobaculum*. **(A)** Consensus structure of all the H/ACA-like motifs. **(B)** Structural subfamily BP1 with one pseudo base-pair corresponding to the (n+6) position in the canonical lower stem. **(C)** Structural subfamily BP2 with one pseudo base-pair corresponding to the (n+9) position in the canonical lower stem. **(D)** Structural subfamily BPS with two pseudo base-pairs corresponding to the (n+6) and (n+9) positions in the canonical lower stem. **(E)** Structural subfamily NONE without any pseudo base-pair.

The second family includes all the other H/ACA-like guide RNAs (sR203 to sR210) except sR207 (Figure 9). Although they are truncated from the lower stem, they generally include one or two possible residual base-pair(s) at the position(s) in the sequence which would be consistent with the subfamilies 55 or 56 from the first family (Figure 9). However, these putative base-pairs are not expected to stabilize the RNA fold especially because they would need to be opened in the RNP complex with the RNA target. The structural notation ‘i/j/k’ (e.g. ‘10/5/9’) is then modified to a simplified notation ‘i/j+k’ (e.g. ‘10/14’) when making the distinction between non-productive/productive folds for H/ACA-like motifs (Table 2).

**Table 2.** Functional and structural features from known H/ACA guide RNAs in *Pyrobaculum* targeting rRNAs and tRNAs

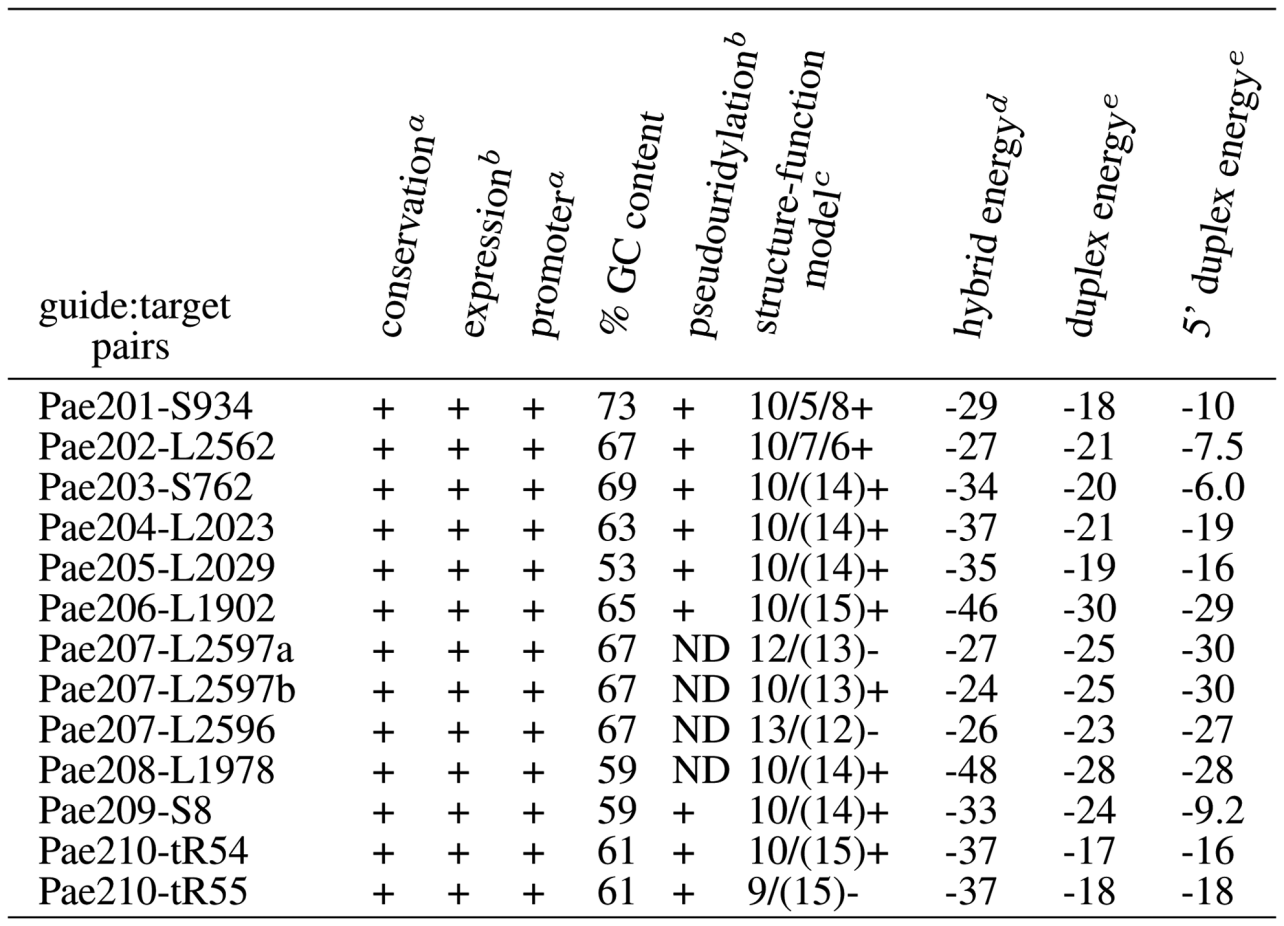									

ND: no direct experimental evidence.

^*a*^as provided by the UCSC genome browser (41,42).

^*b*^as determined from high-throughput pyrosequencing (expression) and experimental validation of RNA targets (productive) (16).

^*c*^structure-function model as proposed from the ‘productive’/‘non-productive’ classification; the +/− sign indicates whether it is predicted to be productive or not.

^*d*^as calculated by RNAsnoop (kcal/mol) from the Vienna RNA package (44).

^*e*^the duplex energy includes both the 5’ and 3’ duplex energies with a correction factor (+4.1 kcal/mol).

From the structural alignment of the H/ACA-like motifs for this family (Supplementary Listing 36), we can propose a classification into four different subfamilies: 64% of the analyzed sequences do contain one or two base-pairing position(s) (subfamilies BPS, BP1 and BP2, Figure 9B–D) reminiscent from the canonical H/ACA motif while 36% lost any trace of the lower stem (subfamily NONE, Figure 9E). The presence of residual base-pairs in about two thirds of the H/ACA-like motifs (Supplementary Figure S22) suggest they may derive from canonical H/ACA motifs degenerated by the accumulation of mutations in the lower stem. The H/ACA-like motifs generally carry an ANA box or a more degenerated NCA box at a distance from the stem which is consistent with the subfamilies 66 or 65 (modulo one residue) and the canonical H/ACA motifs (59%). Some motifs (27%) still carry a degenerated box (NCA) which is downstream of the expected position (up to 10–12 residues). In a few cases (14%), there is no ANA or NCA box (Supplementary Figure S22).

**Figure 10.**
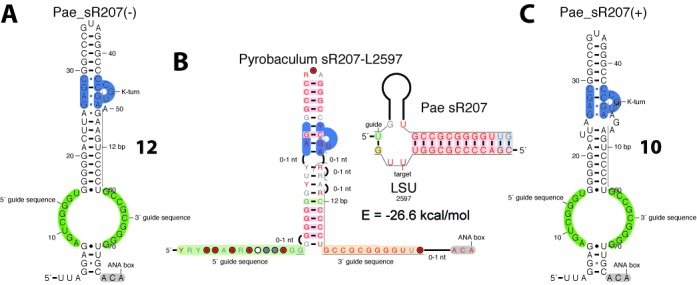
RNA folds and pairing model of the Pae_sR207 guide. **(A)** Non-productive fold proposed by Bernick *et al*. (16) corresponding to a 12/13 model. **(B)** Guide:target complex Pae_sR207-L2597 corresponding to the 12/13 model. **(C)** Productive fold corresponding to a 10/(13) model compatible with the L2597 target.

The third family just includes the remaining guide RNA: Pae_sR207 which can be folded into some intermediate between the H/ACA and H/ACA-like motifs with a short lower stem including 3 canonical base-pairs (Figure 10). The associations between Pae_sR207 and its two ribosomal targets L2597 and L2596 (16) are based on a 12/(13)- (Figure 10A, B and Supplementary Figure S23) and 13/(12)- (Supplementary Figure S24) model of pairing, respectively. There is no direct evidence that these two ribosomal positions are actually modified by Pae_sR207 and none of the paring models is expected to be productive (Table 2). The L2596 position is particularly questionable since it requires the presence of two successive wobble base-pairs closing the GA stem while the presence of a second base-pair (U-G in *P. aerophilum*) is not supported by phylogenetic data (Supplementary Figure S23 and S24). Alternative folds that would restore a productive H/ACA-like motif can be proposed by excluding some nucleotides from the stacking layers of the GA stem (Figure 10C, Supplementary Listing 37). However, there is no compatible compensation that would allow Pae_sR207 to target L2596. Alternative targets were identified in 16S, 23S rRNAs and tRNAs but half of the guide:target pairs exhibit a weak 5’ duplex (above the energy cutoff of −6 kcal/mol) and the other half: some pairing anomalies similar to those already described in *P. abyssi* (Table S2).

### Common gene targets in Pyrococcus and Pyrobaculum

The full genomes of *P. abyssi* and *P. aerophilum* were also explored to identify extra-ribosomal targets (as shown in Figure 3) associated with H/ACA(-like) guide RNAs from both *P. abyssi* and *P. aerophilum*. Since guide RNAs (C/D box or H/ACA box sRNAs) have already been proposed to act as antisense RNAs on a series of genes in *Pyrobaculum* (59), we have focused our analysis on those known genes or functionally related genes that we have also identified as potential common targets of the H/ACA RNP machinery. Out of 246 potential gene targets (data not shown), we have retrieved three identified genes with the following products: the elongation factor EF-2, the isoleucyl-tRNA synthetase (ileS) and the 3-dehydroquinate synthase. These three guide:target complexes correspond to productive or non-productive folds but they are all suboptimal either because of an unstable hybrid or 5’ duplex or the presence of base-pairing anomalies. Nevertheless, many other tRNA synthetases genes (33%) are also potential targets (thrS, cysS, valS, alaS, leuS, pheT, metS, glTX). Among those targets, the leucyl-tRNA synthetase (leuS) satisfies all the energetic and base-pairing rules to be a potential target recognized by 4 different H/ACA(-like) motifs: Pa19, Pa105.2 and Pa160 and Pae_sR207 (Supplementary Figure S25).

## DISCUSSION

### Structural compensations in H/ACA(-like) guide RNAs

In all the 3D structures of the full H/ACA RNP complexes (L7Ae, Nop10, Cbf5 and Gar1) including an RNA substrate or not, the GA stem is composed of exactly 10 base-pairs. The only exception is the case of the incomplete H/ACA RNP complex reconstituted with a composite guide RNA derived from Pf9 (Pa160 ortholog) which exhibits a 9 base-pairs constraint (PDB ID: 2RFK) (29). Interestingly, the L7Ae protein is missing in this particular H/ACA RNP complex. This suggests that the length of the GA stem is indeed a critical structural determinant for the function of the H/ACA guide RNA machinery; the productive H/ACA guide RNAs can be folded or refolded to fit into the consensus model (through a series of structural compensations described previously) with a GA stem including 10 base-pairs or 10 stacked layers which preserve the orientation of the K-turn or K-loop motif in the H/ACA sRNP complex. These findings are consistent with the structure of a functional H/ACA RNP complex (PDB ID: 3HJW and 3HJY) (31) including the H/ACA RNA guide assembled from the Pf9 and Pf6 motifs (PDB ID: 3LWO; Figure 4B and Supplementary Figure S4).

The two crystallized H/ACA guide RNAs (PDB IDs: 3LWO and 3HAX; Figure 4B and Figure 4G) which are representative from both fold families do exhibit the same relative positions of the internal loop, lower stem and ANA box when superposing the two RNP complexes. In the ‘10/6/9’ family, the additional stacked nucleotide in the 3’ end duplex sits at the location of the first base-pair from the lower stem in the ‘10/5/9’ family. This is made possible by a change in the morphology of the lower stem where the RNA helix is more bent in ‘10/6/9’. This more pronounced bending is associated with a more compact helix (smaller helical rise) by almost 1Å and less twisted (smaller helical twist) by about 15 degrees (Table S3). At the junction between the lower stem and the internal loop, the helical twist is large in ‘10/5/9’ (43.1°) but small in ‘10/6/9’ (30.8°) with respect to a canonical A-RNA helix (33.6°). As suggested in a previous work (25), the RNA sequence may have an influence on the intrinsic bending of the lower stem of H/ACA guide RNAs. It is likely that the proteins of the RNP particle also play a non-specific role to induce a bending suitable with its catalytic activity in this portion of the guide RNA.

Some minor structural variations can alter the canonical fold type. In the ‘10/5/9’ family, Pa40.1-L2588 is truncated by one base-pair at the bottom of the lower stem (Supplementary Figure S5). However, the distance between the first base-pair of the lower stem and the ANA box remains the same: 9 nucleotides (8 base-pairs and one unpaired U nucleotide). In other closely related species like *P. horikoshii*, *P. yayanossi* and *T. kodakaraensis*, the first base-pair is preserved as a wobble U-G or U-A base-pair. The second minor variation involves a bulge nucleotide in Pa105.1 (A at position 16: Pa105.1-L2554a in Supplementary Figure S5) which is assumed to be stacked in the GA stem: this conformation is supported by compensatory changes in the motif from *T. kodakaraensis* corresponding to the ortholog gene from *P. abyssi*: the substitution of the bulge nucleotide by a base-pair which is correlated with the substitution of the two additional G-A sheared base-pairs embedded in the GA stem by two canonical base-pairs (Supplementary Figure S8). The presence of both a single bulge nucleotide and two additional sheared G-A base-pairs in the GA stem are expected to allow the adjustment of the helical twist so that the relative position of the K-turn/K-loop motif with respect to the internal loop is preserved (a bulge A nucleotide adopts such a stacked conformation in the 24-mer RNA hairpin coat protein binding site from bacteriophage R17 (PDB ID: 1RHT (60)). A similar structural compensation can be invoked in the case of Pa19 as described in the previous section.

H/ACA guide RNAs from homolog genes in other species can switch between different subfamilies due to the insertion(s) or deletion(s) in the internal loop. In the subfamily 55 (Figure 4D), Pa160 has one homolog in *P. furiosus*: Pf9 that switches to the subfamily 65. The homologs of Pa35.2 in *Thermococcus kodakaraensis* and Pa105.2 in *P. furiosus* switch from subfamily 56 (Figure 4I) to 66 (Figure 4H). The homolog of Pa40.1 in *T. kodakaraensis* switches from subfamily 85 (Figure 4F) to 65 (data not shown).

In *Pyrobaculum*, the H/ACA guide RNAs (sR201 & sR202) adopt typical productive folds from the ‘10/5/9’ family (Supplementary Figure S21). The only structural particularity is the one base-pair shortening of the lower stem (8 base-pairs) usually compensated by the presence of one or two bulge nucleotides and wobble or mismatches (especially at the first positions of the stem). The distance constraint between the ANA box and the closing base-pair of the GA stem would be then reduced to only 13 nucleotides. However, the original distance constraint equivalent to 14 or 15 nucleotides can be satisfied by either stacking one bulge nucleotide or elongating the RNA chain by breaking the two first base-pairs of the lower stem (Supplementary Figure S21). In the H/ACA-like motifs, the 10 base-pair constraint in the GA stem is satisfied. Structural compensations similar to those described for H/ACA motifs can be invoked to satisfy this distance constraint by stacking a bulge nucleotide to preserve 10 stacked layers in the GA stem (Supplementary Figure S22).

### H/ACA(-like) guide candidates in Pyrococcus

**Figure 11.**
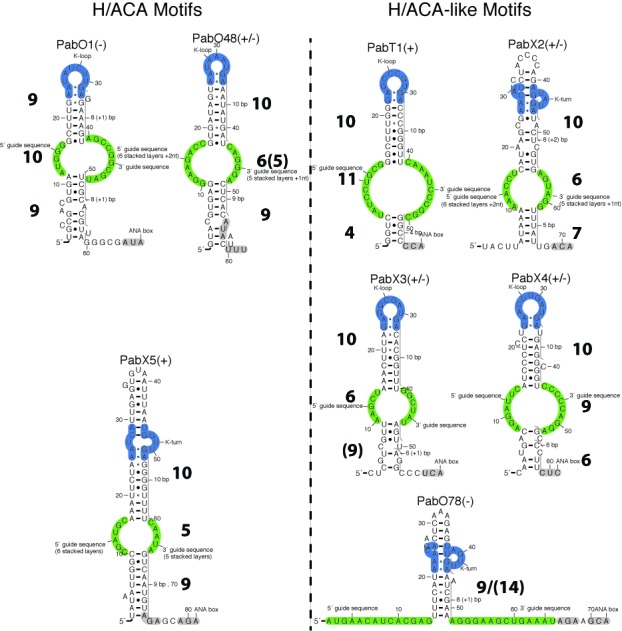
New H/ACA(-like) motifs identified in *Pyrococcus abyssi* (36). The functional annotation (+, - or ±) indicates the relevance of the H/ACA(-like) candidate based on the following criteria: (1) presence of a productive fold (’10/6/9’, ’10/5/9’ or equivalent folds), (2) distance of 14 to 15 residues from the GA stem to the ANA box, (3) presence of an ANA box, (4) absence of alternative folds due to pairings in the internal loop. (+): all four criteria are met; (±) only three criteria are met; (-) less than three criteria are met.

Based on the H/ACA-like motifs present in *Pyrobaculum* genomes, the genome of *P. abyssi* was scanned for the presence of similar motifs in transcribed regions (36) (see Figure 2). Several new H/ACA(-like) motifs were identified and ranked according to several criteria: GC-content, conservation, expression, promoter strength (36). Among the 8 new candidates thus identified, three of them look like canonical H/ACA motifs with minor variations and the five other ones correspond to H/ACA-like motifs where the lower stem is absent or mostly truncated (Figure 11). Three of the motifs were initially considered of particular interest because they satisfy at least two of the criteria listed above: PabO1 (high expression, strong promoter, conservation, medium GC-content; Supplementary Figure S26), PabO48 (high conservation, high expression; Supplementary Figure S27), PabO78 (high expression, strong promoter, conservation; Supplementary Figure S28). PabO48 looks like a canonical H/ACA motif while PabO1 and PabO78 are H/ACA-like motifs suggesting these non-canonical motifs might not be specific to Crenarchaea. However, both PabO1 or PabO78 adopt a non-productive fold (‘9/10/9’ and ‘9/14’, respectively); the ANA box signature is present in both motifs but it is not located at the proper position in PabO1. There are three tRNA genes with 12- or 13-mer sequences that could be targeted by PabO1 (tRNA-Pro^*CGG*^, tRNA-Pro^*TGG*^, tRNA-Asn^*TAA*^, data not shown) but missing a U residue at the proper position. PabO48 is the only motif that could target a ribosomal position (L2047) but it does not carry any ANA box signature at the expected position.

A fourth motif, PabT1 (Supplementary Figure S29), also meets the indicated criteria but it is embedded within the tRNA$^{Pro}_{TGG}$ gene and no potential target was found in the whole genome for this motif. The other four motifs are annotated PabX2 to PabX5 (Supplementary Figures S30–S33): they are all conserved in at least two *Pyrococcus* species and located in expressed regions except PabX2. Only PabX4 has a strong promoter and only PabX5 corresponds to a canonical H/ACA motif. PabX5 is the only canonical motif that includes a proper ANA box but no target was found in the genome (using the same energy criteria; see Materials and Methods). Only a few sub-optimal targets were identified (data not shown): one of them correspond to a gene (leuS) already targeted by three other canonical H/ACA guide RNAs (Supplementary Figure S25). PabX2 and PabX3 can adopt alternative folds involving several pairings in the internal loop suggesting they may be degenerated H/ACA-like motifs or just false-positive candidates. The structural and functional features of these 8 H/ACA(-like) motifs are summarized in the supplementary material (Table S4).

### Significance of H/ACA guide RNAs targeting CDS

The presence of genes targeted by guide RNAs (C/D box or H/ACA box sRNAs) in *Pyrobaculum* (59) suggests a possible regulatory role mediated through RNA:RNA interactions. In the case of H/ACA guide RNAs, it is tempting to assume that the interaction between the guide and a mRNA target follows the same rules of association that apply for rRNA or tRNA targets, as proposed in the case of the leucyl-tRNA synthetase (Supplementary Figure S25). The full or partial association with the proteins of the H/ACA RNP particle may stabilize the association between the guide and the target. However, such regulatory role of H/ACA guide RNAs would not require the complex to be functional for the pseudo-uridylation of the mRNA target except for a regulation of the termination of the translation. As shown in *S. cerevisiae*, the association between the snR81 H/ACA guide and some mRNA target is sub-optimal under stress conditions (61). Thus, the energetic rules could be adjusted to identify extra-ribosomal targets which may be sub-optimal with respect to the natural RNA substrates.

## CONCLUSION

A more precise structural and functional classification of archaeal H/ACA guide RNAs is proposed based on the X-ray data of H/ACA RNPs and 2D RNA predictions supported by the phylogenetic data of the known H/ACA(-like) motifs and their target(s); it is consistent with all the current data available up to now in both Euryarchaea and Crenarchaea. One of the major structural determinants is the presence of a stretch of 10 stacked layers in the GA stem from the closing base-pair of the pseudo-uridylation pocket to the G-A sheared base-pairs of the K-turn or K-loop motif. A distance of 14 or 15 nucleotides from the same closing base-pair to the ANA box proposed previously as another structural determinant (5) is the result of the conformational flexibility at the junction between the internal loop and the lower stem. The 3’ single-stranded region of the internal loop usually varies between 5 and 6 nucleotides in the typical RNA folds: in the subfamily 65, the helical twist is more pronounced at the junction than in the subfamily 66 and compensates for the shorter 3’ single-stranded region. Finally, the stability of the 5’ duplex also appears to play a key role in the formation of productive RNA-RNA complexes. It probably contributes to tether the uridine to be modified in the pseudo-uridylation pocket and to restrain its mobility in the catalytic site of Cbf5.

A more detailed structural analysis of H/ACA-like guide RNAs in the *Pyrobaculum* genus reveals a strong similarity with the canonical H/ACA guide RNAs previously identified in both Euryarchaea and Crenarchaea. Two particular sRNAs (sR201 and sR202) can actually be considered as canonical H/ACA guide RNAs. The other H/ACA-like guide RNAs (sR203 to sR210) can be classified in different subfamilies which retain to some degree the vestiges of the lower stem. The H/ACA(-like) motifs identified recently in *P. abyssi* (36) show a similar signature with a pseudo lower stem degenerated by the incorporation of mismatches and bulges. According to this current work, all the H/ACA(-like) motifs from *P. aerophilum* can be classified as productive as expected from the published data (16). However, the position L2596 is not expected to be modified by the H/ACA-like motif Pae_sR207. The presence of genes which might be regulated by H/ACA(-like) RNAs, a hypothesis which was already proposed based on experimental evidence in *Pyrobaculum* (59), remains to be tested in archaea.

## SUPPLEMENTARY DATA

Supplementary Data are available at NAR online.

SUPPLEMENTARY DATA
