# Supplementary material for: Revisiting the structure/function relationships of H/ACA(-like) RNAs: a unified model for Euryarchaea and Crenarchaea
Source: Nucleic Acids Res. 2015 Aug 3;43(16):7744–61. doi: 10.1093/nar/gkv756 (PMC4652768; doi:10.1093/nar/gkv756)
Supplement: SUPPLEMENTARY DATA [file supp_43_16_7744__index.html]

Revisiting the structure/function relationships of H/ACA(-like) RNAs: a unified model for Euryarchaea and Crenarchaea — Revisiting the structure/function relationships of H/ACA(-like) RNAs: a unified model for Euryarchaea and Crenarchaea — SUPPLEMENTARY DATA 

# Revisiting the structure/function relationships of H/ACA(-like) RNAs: a unified model for Euryarchaea and Crenarchaea

## SUPPLEMENTARY DATA

- SUPPLEMENTARY DATA
