# Supplementary material for: Revisiting the structure/function relationships of H/ACA(-like) RNAs: a unified model for Euryarchaea and Crenarchaea
Source: Nucleic Acids Res. 2015 Aug 3;43(16):7744–61. doi: 10.1093/nar/gkv756 (PMC4652768; doi:10.1093/nar/gkv756)
Supplement: SUPPLEMENTARY DATA [file supp_gkv756_nar-00719-z-2015-File002.pdf]

SUPPLEMENTARY DATA

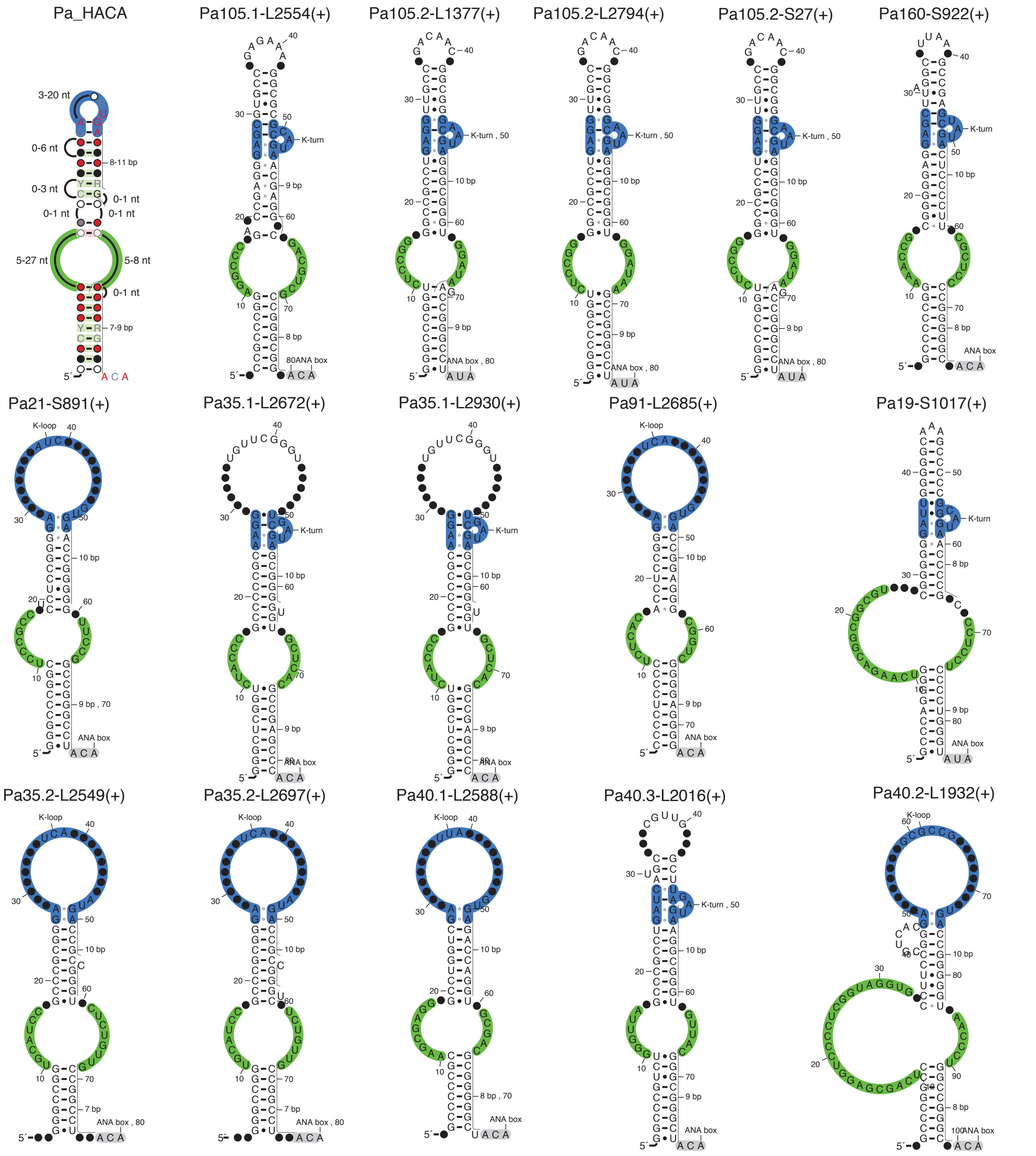

(a)

Figure S1. Structure/Function Model of H/ACA guide RNAs in *Pyrococcus abyssi* from Muller *et al* (5). (a) Details of the H/ACA folds associated with productive guide:target complexes. All the proposed guide:target pairs are included with an indication of the experimentally validated target where the expected U position is pseudo-uridylated: (+) for the “productive” duplexes, (-) for the “non-productive” duplexes.

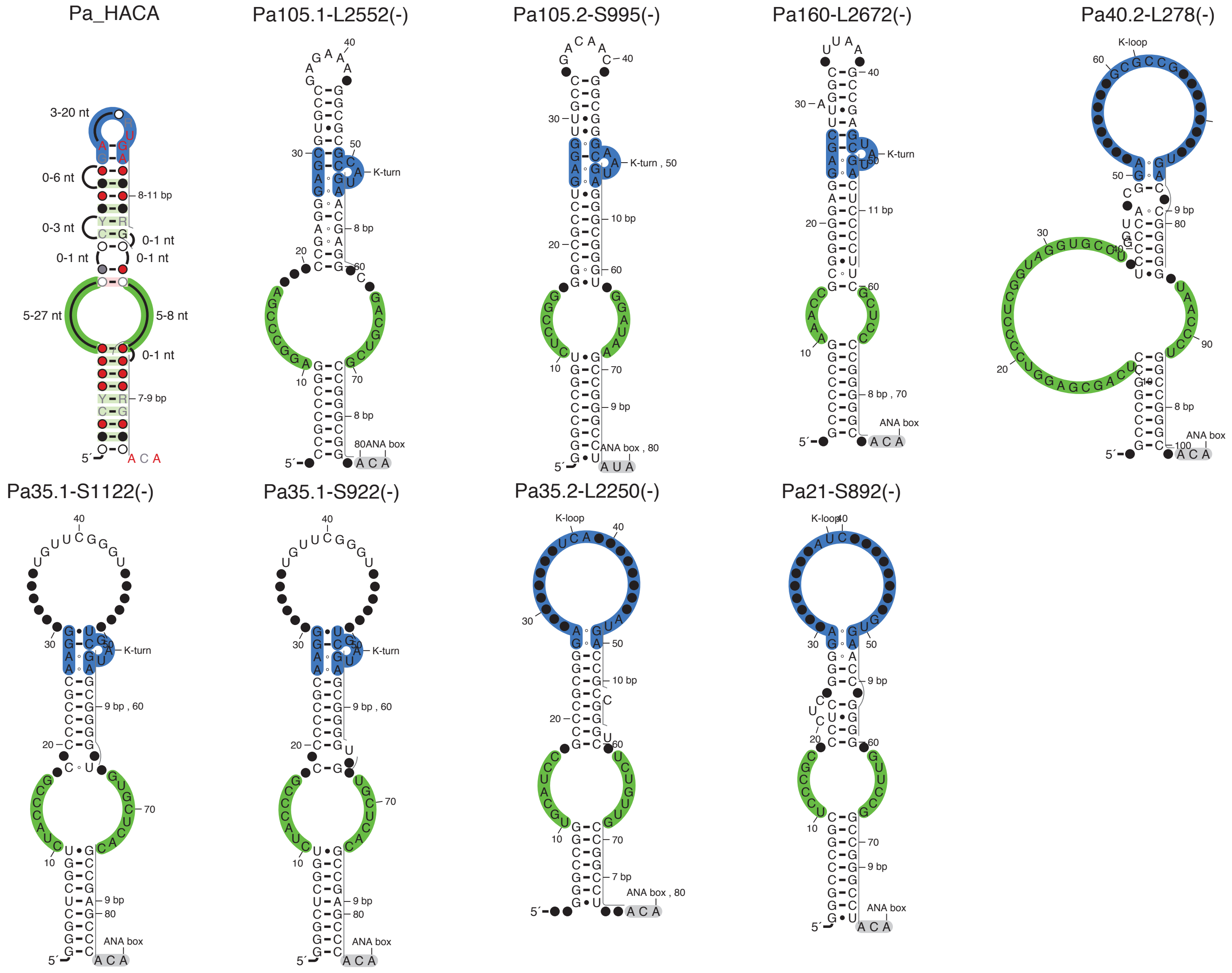

(b)

Figure S1. Structure/Function Model of H/ACA guide RNAs in *Pyrococcus abyssi* from Muller *et al* (5) (continued). (b) Details of the H/ACA folds associated with non-productive guide:target complexes.

Listing 1: Stockholm Alignment used in R2R representations of the Structure/Function Model of H/ACA guide RNAs in *Pyrococcus abyssi* (Fig. S1).

```
# STOCKHOLM 1.0

Pa19-S1017                  GCCCAGGGGUCAAGACGGCGGCGU................C...G....GGG...........GGA....UUG.G...GGGC...AAA...GCCCCCGG...CAUGA.ACCC.CG...C.CCUCCU..C.CCCUGGGU..AUA
Pa105.1-L2552               .CCGCCCGGA......GGCCCGA.................C...C....GAG...........GGA....GCG.U...GCCG..AGAAA...GGCGCGC...CAUGA.ACGA.GG...C.GACGUC.GC.CGGGCGG...ACA
Pa21-S892                   GGGCCCGGC......UCCCGC................C.CUCUCC.....GG...........GGA.................AUC.................GUGA.ACC..GG.G.G.GUUCCG..G.CCGGGCCU..ACA
Pa35.1-S922                 GGGCUCGGU.....CUACCCG................C..C...C....CCG...........CAA....GG..........UGUUCGGGU......UC...GAUGA.GCGG.GGU..G.UGCUCAC.G.CCGAGCCC..ACA
Pa35.1-S1122                GGGCUCGGU.....CUACCCG................C..C...C....CCG...........CAA....GG..........UGUUCGGGU......UC...GAUGA.GCGG.GG...U.GUGCUCACG.CCGAGCCC..ACA
Pa40.2-L278                 .GCCCGGCCUCAGCGAGGUCCCCUCGGUAGGUGCCU.U.CCG..C.GU.CA.C..........GGA................GCGCCG................UGA.C.CG.GG.G.G.UAACCCU.G.GCCGGGC...ACA
Pa21-S891                   GGGCCCGGC....UCCCGCC................CUCU...C....CGG...........GGA.................AUC.................GUGA.ACCG.GG.G.G.UUCCG...G.CCGGGCCU..ACA
Pa35.1-L2930                GGGCUCGGU......CUACCC................G.CC...C....CCG...........CAA....GG..........UGUUCGGGU......UC...GAUGA.GCGG.GGUG.U.GCUCAC..G.CCGAGCCC..ACA
Pa35.1-L2672                GGGCUCGGU......CUACCC................G.CC...C....CCG...........CAA....GG..........UGUUCGGGU......UC...GAUGA.GCGG.GGUG.U.GCUCAC..G.CCGAGCCC..ACA
Pa35.2-L2549                ..GGGCCGG....UGCAUCC................G.CC...C....GCG...........GGA.................UCA.................AUGA.CCGCCGG.G.U.CUCUGUUGC.CGGCCU....ACA
Pa35.2-L2697                ..GGGCCGG....UGCAUCC................G.CC...C....GCG...........GGA.................UCA.................AUGA.CCGCCGG.GUC..UCUGUUGC.CGGCCU....ACA
Pa35.2-L2250                ..GGGCCGG....UGCAUCC................G.CC...C....GCG...........GGA.................UCA.................AUGA.CCGCCGG.GUC.UCUGUUG.C.CGGCCU....ACA
Pa40.1-L2588                .GCCCCCGC....AAGCGAGG................G.CC...U....GGU...........CGA.................UUA.................GUGA.GACC.AG.G.U.GCGAC...G.CGGGGGCU..ACA
Pa40.2-L1932                .GCCCGGCCUCAGCGAGGUCCCCUCGGUAGGUG....C.CU...U....CCG.CGUCAC....GGA................GCGCCG................UGA.CCGG.GG.G.U.AACCCU..G.GCCGGGC...ACA
Pa40.3-L2016                GGCCCGUCU......GGGUUA................G.CC...C....GCC...........UGA....UCAUG...C...CGUUG.......GCUUA...GAUGA.AGGC.GG.G.U.GUUAC...G.GGCGGGCU..ACA
Pa91-L2685                  CCCCUCCCC......UCUCAC................A.CC...U....CCG...........GGA.................UCA.................GUGA.CCGG.AG.G.G.CGGUC...G.GGGAGGGG..ACA
Pa105.1-L2554               .CCGCCCGGA......GGCCC................GA.C...C....GAG...........GGA....GCG.U...GCC.GAGAAA....GGCGCGC...CAUGA.ACGA.GG...C.GACGUC.GC.CGGGCGG...ACA
Pa105.2-S995                GGGCCCGGU......CUCCGG................G.GC...C....GCC...........UGA....GGU.U...GCC..GACAAC...GGCGGGC...AAUGA.GGGC.GG.G.U.GGAUAA..G.CCGGGCCU..AUA
Pa105.2-L2794               GGGCCCGGU......CUCCGG................G.GC...C....GCC...........UGA....GGU.U...GCC..GACAAC...GGCGGGC...AAUGA.GGGC.GG.G.U.GGAUAA..G.CCGGGCCU..AUA
Pa105.2-L1377               GGGCCCGGU......CUCCGG................G.GC...C....GCC...........UGA....GGU.U...GCC..GACAAC...GGCGGGC...AAUGA.GGGC.GG.G.U.GGAUA...AGCCGGGCCU..AUA
Pa105.2-S27                 GGGCCCGGU......CUCCGG................G.GC...C....GCC...........UGA....GGU.U...GCC..GACAAC...GGCGGGC...AAUGA.GGGC.GG.G.U.GGAUA...AGCCGGGCCU..AUA
Pa160-S922                  .GCCCCCGG......AAACCG................C.GG...G....GGA...........GGA....GCU.U.A.GGC....UUAA...GCCGAGC...UAUGA.CUCC.CC.U.U.CGCUCC..C.CGGGGGC...ACA
Pa160-L2672                 .GCCCCCGG......AAACC................GC.GG...G....GGA...........GGA....GCU.U.A.GGC....UUAA...GCCGAGC...UAUGA.CUCC.CC.U.UC.GCUC..CC.CGGGGGC...ACA
#=GC SS_cons                <<<<<<<<<...........................<<-<<...<....<<<...........<<<....<<<.<...<<<<_________>>>>>>>>...-..>>.>>>>->>->.>>......-.>->>>>>>>>.....
#=GC R2R_LABEL              q........1.........................2..3..a.b.........4......5...II.6...p....................p......7.....HH.HHiH.HHBHc..8......0H9HHHhHHHr.....
#=GC R2R_XLABEL_KTURN       ................................................................TTTTTTTT.........................RRRRRRRRRR....................................
#=GC R2R_XLABEL_KTURNL      .......................................................................................................r.......................................
#=GC R2R_XLABEL_KLOOP       ................................................................KKKKKKKKKKKKKKKKKKKKKKKKKKKKKKKKKKKKKKKKKKK....................................
#=GC R2R_XLABEL_KLOOPL      ....................................................................................k..........................................................
#=GC R2R_XLABEL_ILOOP       .........PPPPPPPPPPPPPPPPPPPQXXXXXXXW...................................................................................YYYYYSUT.V..........ZZZ
#=GC R2R_XLABEL_ILOOPL      ...................x................w..........................................................................................y.sut.v............z
#=GC R2R_XLABEL_STEM        ...............................................................................................................................................
#=GC R2R_XLABEL_STEML       ...............................................................................................................................................

#=GF R2R keep allpairs
#=GF R2R var_backbone_range_size_fake_nucs 7 1 2
#=GF R2R var_backbone_range_size_fake_nucs 3 a b
#=GF R2R var_backbone_range_size_fake_nucs 6 8 0
#=GF R2R var_backbone_range 4 5
#=GF R2R var_backbone_range 6 7
#=GF R2R var_backbone_range_size_fake_nucs 1 3 3
#=GF R2R var_backbone_range_size_fake_nucs 1 9 9
#=GF R2R var_backbone_range_size_fake_nucs 1 c c
#=GF R2R var_backbone_range_size_fake_nucs 1 B B
#=GF R2R shade_along_backbone ILOOP:P rgb:0,255,0
#=GF R2R shade_along_backbone ILOOP:Q rgb:0,255,0
#=GF R2R shade_along_backbone ILOOP:X rgb:0,255,0
#=GF R2R shade_along_backbone ILOOP:Y rgb:0,255,0
#=GF R2R shade_along_backbone ILOOP:S rgb:0,255,0
#=GF R2R shade_along_backbone ILOOP:U rgb:0,255,0
#=GF R2R outline_nuc H
#=GF R2R outline_nuc h
#=GF R2R outline_nuc r
#=GF R2R outline_nuc i
#=GF R2R tick_label h 7-9 bp
#=GF R2R tick_label i 8-11 bp

#=GF R2R_oneseq Pa19-S1017 shade_along_backbone KTURN:T rgb:0,129,255
#=GF R2R_oneseq Pa19-S1017 shade_along_backbone KTURN:R rgb:0,129,255
#=GF R2R_oneseq Pa19-S1017 tick_label KTURNL:r K-turn
#=GF R2R_oneseq Pa19-S1017 shade_along_backbone ILOOP:Y rgb:0,255,0
#=GF R2R_oneseq Pa19-S1017 shade_along_backbone ILOOP:S rgb:0,255,0
#=GF R2R_oneseq Pa19-S1017 shade_along_backbone ILOOP:U rgb:0,255,0
#=GF R2R_oneseq Pa19-S1017 shade_along_backbone ILOOP:P rgb:0,255,0
#=GF R2R_oneseq Pa19-S1017 shade_along_backbone ILOOP:Q rgb:0,255,0
#=GF R2R_oneseq Pa19-S1017 shade_along_backbone ILOOP:X rgb:0,255,0
#=GF R2R_oneseq Pa19-S1017 shade_along_backbone ILOOP:Z rgb:200,200,200
#=GF R2R_oneseq Pa19-S1017 tick_label ILOOPL:z ANA box

#=GF R2R_oneseq Pa105.1-L2552 shade_along_backbone KTURN:T rgb:0,129,255
#=GF R2R_oneseq Pa105.1-L2552 shade_along_backbone KTURN:R rgb:0,129,255
#=GF R2R_oneseq Pa105.1-L2552 tick_label KTURNL:r K-turn
#=GF R2R_oneseq Pa105.1-L2552 shade_along_backbone ILOOP:Y rgb:0,255,0
#=GF R2R_oneseq Pa105.1-L2552 shade_along_backbone ILOOP:S rgb:0,255,0
#=GF R2R_oneseq Pa105.1-L2552 shade_along_backbone ILOOP:U rgb:0,255,0
#=GF R2R_oneseq Pa105.1-L2552 shade_along_backbone ILOOP:T rgb:0,255,0
#=GF R2R_oneseq Pa105.1-L2552 shade_along_backbone ILOOP:P rgb:0,255,0
#=GF R2R_oneseq Pa105.1-L2552 shade_along_backbone ILOOP:Q rgb:0,255,0
#=GF R2R_oneseq Pa105.1-L2552 shade_along_backbone ILOOP:X rgb:0,255,0
#=GF R2R_oneseq Pa105.1-L2552 shade_along_backbone ILOOP:Z rgb:200,200,200
#=GF R2R_oneseq Pa105.1-L2552 tick_label ILOOPL:z ANA box

#=GF R2R_oneseq Pa21-S892 shade_along_backbone KLOOP:K rgb:0,129,255
#=GF R2R_oneseq Pa21-S892 tick_label KLOOPL:k K-loop
#=GF R2R_oneseq Pa21-S892 shade_along_backbone ILOOP:Y rgb:0,255,0
#=GF R2R_oneseq Pa21-S892 shade_along_backbone ILOOP:S rgb:0,255,0
#=GF R2R_oneseq Pa21-S892 shade_along_backbone ILOOP:U rgb:0,255,0
#=GF R2R_oneseq Pa21-S892 shade_along_backbone ILOOP:P rgb:0,255,0
#=GF R2R_oneseq Pa21-S892 shade_along_backbone ILOOP:Q rgb:0,255,0
#=GF R2R_oneseq Pa21-S892 shade_along_backbone ILOOP:X rgb:0,255,0
#=GF R2R_oneseq Pa21-S892 shade_along_backbone ILOOP:Z rgb:200,200,200
#=GF R2R_oneseq Pa21-S892 tick_label ILOOPL:z ANA box

#=GF R2R_oneseq Pa35.1-S922 shade_along_backbone KTURN:T rgb:0,129,255
#=GF R2R_oneseq Pa35.1-S922 shade_along_backbone KTURN:R rgb:0,129,255
#=GF R2R_oneseq Pa35.1-S922 tick_label KTURNL:r K-turn
#=GF R2R_oneseq Pa35.1-S922 shade_along_backbone ILOOP:Y rgb:0,255,0
#=GF R2R_oneseq Pa35.1-S922 shade_along_backbone ILOOP:S rgb:0,255,0
#=GF R2R_oneseq Pa35.1-S922 shade_along_backbone ILOOP:U rgb:0,255,0
#=GF R2R_oneseq Pa35.1-S922 shade_along_backbone ILOOP:P rgb:0,255,0
#=GF R2R_oneseq Pa35.1-S922 shade_along_backbone ILOOP:Q rgb:0,255,0
#=GF R2R_oneseq Pa35.1-S922 shade_along_backbone ILOOP:X rgb:0,255,0
#=GF R2R_oneseq Pa35.1-S922 shade_along_backbone ILOOP:Z rgb:200,200,200
#=GF R2R_oneseq Pa35.1-S922 tick_label ILOOPL:z ANA box
```

```
#=GF R2R_oneseq Pa35.1-S1122 shade_along_backbone KTURN:T rgb:0,129,255
#=GF R2R_oneseq Pa35.1-S1122 shade_along_backbone KTURN:R rgb:0,129,255
#=GF R2R_oneseq Pa35.1-S1122 tick_label KTURNL:r K-turn
#=GF R2R_oneseq Pa35.1-S1122 shade_along_backbone ILOOP:Y rgb:0,255,0
#=GF R2R_oneseq Pa35.1-S1122 shade_along_backbone ILOOP:S rgb:0,255,0
#=GF R2R_oneseq Pa35.1-S1122 shade_along_backbone ILOOP:U rgb:0,255,0
#=GF R2R_oneseq Pa35.1-S1122 shade_along_backbone ILOOP:T rgb:0,255,0
#=GF R2R_oneseq Pa35.1-S1122 shade_along_backbone ILOOP:P rgb:0,255,0
#=GF R2R_oneseq Pa35.1-S1122 shade_along_backbone ILOOP:Q rgb:0,255,0
#=GF R2R_oneseq Pa35.1-S1122 shade_along_backbone ILOOP:X rgb:0,255,0
#=GF R2R_oneseq Pa35.1-S1122 shade_along_backbone ILOOP:Z rgb:200,200,200
#=GF R2R_oneseq Pa35.1-S1122 tick_label ILOOPL:z ANA box

#=GF R2R_oneseq Pa40.2-L278 shade_along_backbone KLOOP:K rgb:0,129,255
#=GF R2R_oneseq Pa40.2-L278 tick_label KLOOPL:k K-loop
#=GF R2R_oneseq Pa40.2-L278 shade_along_backbone ILOOP:Y rgb:0,255,0
#=GF R2R_oneseq Pa40.2-L278 shade_along_backbone ILOOP:S rgb:0,255,0
#=GF R2R_oneseq Pa40.2-L278 shade_along_backbone ILOOP:U rgb:0,255,0
#=GF R2R_oneseq Pa40.2-L278 shade_along_backbone ILOOP:P rgb:0,255,0
#=GF R2R_oneseq Pa40.2-L278 shade_along_backbone ILOOP:Q rgb:0,255,0
#=GF R2R_oneseq Pa40.2-L278 shade_along_backbone ILOOP:X rgb:0,255,0
#=GF R2R_oneseq Pa40.2-L278 shade_along_backbone ILOOP:Z rgb:200,200,200
#=GF R2R_oneseq Pa40.2-L278 tick_label ILOOPL:z ANA box

#=GF R2R_oneseq Pa21-S891 shade_along_backbone KLOOP:K rgb:0,129,255
#=GF R2R_oneseq Pa21-S891 tick_label KLOOPL:k K-loop
#=GF R2R_oneseq Pa21-S891 shade_along_backbone ILOOP:Y rgb:0,255,0
#=GF R2R_oneseq Pa21-S891 shade_along_backbone ILOOP:S rgb:0,255,0
#=GF R2R_oneseq Pa21-S891 shade_along_backbone ILOOP:U rgb:0,255,0
#=GF R2R_oneseq Pa21-S891 shade_along_backbone ILOOP:P rgb:0,255,0
#=GF R2R_oneseq Pa21-S891 shade_along_backbone ILOOP:Q rgb:0,255,0
#=GF R2R_oneseq Pa21-S891 shade_along_backbone ILOOP:X rgb:0,255,0
#=GF R2R_oneseq Pa21-S891 shade_along_backbone ILOOP:Z rgb:200,200,200
#=GF R2R_oneseq Pa21-S891 tick_label ILOOPL:z ANA box

#=GF R2R_oneseq Pa35.1-L2930 shade_along_backbone KTURN:T rgb:0,129,255
#=GF R2R_oneseq Pa35.1-L2930 shade_along_backbone KTURN:R rgb:0,129,255
#=GF R2R_oneseq Pa35.1-L2930 tick_label KTURNL:r K-turn
#=GF R2R_oneseq Pa35.1-L2930 shade_along_backbone ILOOP:Y rgb:0,255,0
#=GF R2R_oneseq Pa35.1-L2930 shade_along_backbone ILOOP:S rgb:0,255,0
#=GF R2R_oneseq Pa35.1-L2930 shade_along_backbone ILOOP:U rgb:0,255,0
#=GF R2R_oneseq Pa35.1-L2930 shade_along_backbone ILOOP:P rgb:0,255,0
#=GF R2R_oneseq Pa35.1-L2930 shade_along_backbone ILOOP:Q rgb:0,255,0
#=GF R2R_oneseq Pa35.1-L2930 shade_along_backbone ILOOP:X rgb:0,255,0
#=GF R2R_oneseq Pa35.1-L2930 shade_along_backbone ILOOP:Z rgb:200,200,200
#=GF R2R_oneseq Pa35.1-L2930 tick_label ILOOPL:z ANA box

#=GF R2R_oneseq Pa35.1-L2672 shade_along_backbone KTURN:T rgb:0,129,255
#=GF R2R_oneseq Pa35.1-L2672 shade_along_backbone KTURN:R rgb:0,129,255
#=GF R2R_oneseq Pa35.1-L2672 tick_label KTURNL:r K-turn
#=GF R2R_oneseq Pa35.1-L2672 shade_along_backbone ILOOP:Y rgb:0,255,0
#=GF R2R_oneseq Pa35.1-L2672 shade_along_backbone ILOOP:S rgb:0,255,0
#=GF R2R_oneseq Pa35.1-L2672 shade_along_backbone ILOOP:U rgb:0,255,0
#=GF R2R_oneseq Pa35.1-L2672 shade_along_backbone ILOOP:P rgb:0,255,0
#=GF R2R_oneseq Pa35.1-L2672 shade_along_backbone ILOOP:Q rgb:0,255,0
#=GF R2R_oneseq Pa35.1-L2672 shade_along_backbone ILOOP:X rgb:0,255,0
#=GF R2R_oneseq Pa35.1-L2672 shade_along_backbone ILOOP:Z rgb:200,200,200
#=GF R2R_oneseq Pa35.1-L2672 tick_label ILOOPL:z ANA box

#=GF R2R_oneseq Pa35.2-L2549 shade_along_backbone KLOOP:K rgb:0,129,255
#=GF R2R_oneseq Pa35.2-L2549 tick_label KLOOPL:k K-loop
#=GF R2R_oneseq Pa35.2-L2549 shade_along_backbone ILOOP:Y rgb:0,255,0
#=GF R2R_oneseq Pa35.2-L2549 shade_along_backbone ILOOP:S rgb:0,255,0
#=GF R2R_oneseq Pa35.2-L2549 shade_along_backbone ILOOP:U rgb:0,255,0
#=GF R2R_oneseq Pa35.2-L2549 shade_along_backbone ILOOP:T rgb:0,255,0
#=GF R2R_oneseq Pa35.2-L2549 shade_along_backbone ILOOP:P rgb:0,255,0
#=GF R2R_oneseq Pa35.2-L2549 shade_along_backbone ILOOP:Q rgb:0,255,0
#=GF R2R_oneseq Pa35.2-L2549 shade_along_backbone ILOOP:X rgb:0,255,0
#=GF R2R_oneseq Pa35.2-L2549 shade_along_backbone ILOOP:Z rgb:200,200,200
#=GF R2R_oneseq Pa35.2-L2549 tick_label ILOOPL:z ANA box

#=GF R2R_oneseq Pa35.2-L2697 shade_along_backbone KLOOP:K rgb:0,129,255
#=GF R2R_oneseq Pa35.2-L2697 tick_label KLOOPL:k K-loop
#=GF R2R_oneseq Pa35.2-L2697 shade_along_backbone ILOOP:Y rgb:0,255,0
#=GF R2R_oneseq Pa35.2-L2697 shade_along_backbone ILOOP:S rgb:0,255,0
#=GF R2R_oneseq Pa35.2-L2697 shade_along_backbone ILOOP:U rgb:0,255,0
#=GF R2R_oneseq Pa35.2-L2697 shade_along_backbone ILOOP:T rgb:0,255,0
#=GF R2R_oneseq Pa35.2-L2697 shade_along_backbone ILOOP:P rgb:0,255,0
#=GF R2R_oneseq Pa35.2-L2697 shade_along_backbone ILOOP:Q rgb:0,255,0
#=GF R2R_oneseq Pa35.2-L2697 shade_along_backbone ILOOP:X rgb:0,255,0
#=GF R2R_oneseq Pa35.2-L2697 shade_along_backbone ILOOP:Z rgb:200,200,200
#=GF R2R_oneseq Pa35.2-L2697 tick_label ILOOPL:z ANA box

#=GF R2R_oneseq Pa35.2-L2250 shade_along_backbone KLOOP:K rgb:0,129,255
#=GF R2R_oneseq Pa35.2-L2250 tick_label KLOOPL:k K-loop
#=GF R2R_oneseq Pa35.2-L2250 shade_along_backbone ILOOP:Y rgb:0,255,0
#=GF R2R_oneseq Pa35.2-L2250 shade_along_backbone ILOOP:S rgb:0,255,0
#=GF R2R_oneseq Pa35.2-L2250 shade_along_backbone ILOOP:U rgb:0,255,0
#=GF R2R_oneseq Pa35.2-L2250 shade_along_backbone ILOOP:T rgb:0,255,0
#=GF R2R_oneseq Pa35.2-L2250 shade_along_backbone ILOOP:P rgb:0,255,0
#=GF R2R_oneseq Pa35.2-L2250 shade_along_backbone ILOOP:Q rgb:0,255,0
#=GF R2R_oneseq Pa35.2-L2250 shade_along_backbone ILOOP:X rgb:0,255,0
#=GF R2R_oneseq Pa35.2-L2250 shade_along_backbone ILOOP:Z rgb:200,200,200
#=GF R2R_oneseq Pa35.2-L2250 tick_label ILOOPL:z ANA box

#=GF R2R_oneseq Pa40.1-L2588 shade_along_backbone KLOOP:K rgb:0,129,255
#=GF R2R_oneseq Pa40.1-L2588 tick_label KLOOPL:k K-loop
#=GF R2R_oneseq Pa40.1-L2588 shade_along_backbone ILOOP:Y rgb:0,255,0
#=GF R2R_oneseq Pa40.1-L2588 shade_along_backbone ILOOP:S rgb:0,255,0
#=GF R2R_oneseq Pa40.1-L2588 shade_along_backbone ILOOP:U rgb:0,255,0
#=GF R2R_oneseq Pa40.1-L2588 shade_along_backbone ILOOP:T rgb:0,255,0
#=GF R2R_oneseq Pa40.1-L2588 shade_along_backbone ILOOP:P rgb:0,255,0
#=GF R2R_oneseq Pa40.1-L2588 shade_along_backbone ILOOP:Q rgb:0,255,0
#=GF R2R_oneseq Pa40.1-L2588 shade_along_backbone ILOOP:X rgb:0,255,0
#=GF R2R_oneseq Pa40.1-L2588 shade_along_backbone ILOOP:Z rgb:200,200,200
#=GF R2R_oneseq Pa40.1-L2588 tick_label ILOOPL:z ANA box

#=GF R2R_oneseq Pa40.2-L1932 shade_along_backbone KLOOP:K rgb:0,129,255
#=GF R2R_oneseq Pa40.2-L1932 tick_label KLOOPL:k K-loop
#=GF R2R_oneseq Pa40.2-L1932 shade_along_backbone ILOOP:Y rgb:0,255,0
#=GF R2R_oneseq Pa40.2-L1932 shade_along_backbone ILOOP:S rgb:0,255,0
```

```
#=GF R2R_oneseq Pa40.2-L1932 shade_along_backbone ILOOP:U rgb:0,255,0
#=GF R2R_oneseq Pa40.2-L1932 shade_along_backbone ILOOP:P rgb:0,255,0
#=GF R2R_oneseq Pa40.2-L1932 shade_along_backbone ILOOP:Q rgb:0,255,0
#=GF R2R_oneseq Pa40.2-L1932 shade_along_backbone ILOOP:X rgb:0,255,0
#=GF R2R_oneseq Pa40.2-L1932 shade_along_backbone ILOOP:Z rgb:200,200,200
#=GF R2R_oneseq Pa40.2-L1932 tick_label ILOOPL:z ANA box
#=GF R2R_oneseq Pa40.2-L1932 var_backbone_range 4 5

#=GF R2R_oneseq Pa40.3-L2016 shade_along_backbone KTURN:T rgb:0,129,255
#=GF R2R_oneseq Pa40.3-L2016 shade_along_backbone KTURN:R rgb:0,129,255
#=GF R2R_oneseq Pa40.3-L2016 tick_label KTURNL:r K-turn
#=GF R2R_oneseq Pa40.3-L2016 shade_along_backbone ILOOP:Y rgb:0,255,0
#=GF R2R_oneseq Pa40.3-L2016 shade_along_backbone ILOOP:S rgb:0,255,0
#=GF R2R_oneseq Pa40.3-L2016 shade_along_backbone ILOOP:U rgb:0,255,0
#=GF R2R_oneseq Pa40.3-L2016 shade_along_backbone ILOOP:T rgb:0,255,0
#=GF R2R_oneseq Pa40.3-L2016 shade_along_backbone ILOOP:P rgb:0,255,0
#=GF R2R_oneseq Pa40.3-L2016 shade_along_backbone ILOOP:Q rgb:0,255,0
#=GF R2R_oneseq Pa40.3-L2016 shade_along_backbone ILOOP:X rgb:0,255,0
#=GF R2R_oneseq Pa40.3-L2016 shade_along_backbone ILOOP:Z rgb:200,200,200
#=GF R2R_oneseq Pa40.3-L2016 tick_label ILOOPL:z ANA box

#=GF R2R_oneseq Pa91-L2685 shade_along_backbone KLOOP:K rgb:0,129,255
#=GF R2R_oneseq Pa91-L2685 tick_label KLOOPL:k K-loop
#=GF R2R_oneseq Pa91-L2685 shade_along_backbone ILOOP:Y rgb:0,255,0
#=GF R2R_oneseq Pa91-L2685 shade_along_backbone ILOOP:S rgb:0,255,0
#=GF R2R_oneseq Pa91-L2685 shade_along_backbone ILOOP:U rgb:0,255,0
#=GF R2R_oneseq Pa91-L2685 shade_along_backbone ILOOP:T rgb:0,255,0
#=GF R2R_oneseq Pa91-L2685 shade_along_backbone ILOOP:P rgb:0,255,0
#=GF R2R_oneseq Pa91-L2685 shade_along_backbone ILOOP:Q rgb:0,255,0
#=GF R2R_oneseq Pa91-L2685 shade_along_backbone ILOOP:X rgb:0,255,0
#=GF R2R_oneseq Pa91-L2685 shade_along_backbone ILOOP:Z rgb:200,200,200
#=GF R2R_oneseq Pa91-L2685 tick_label ILOOPL:z ANA box

#=GF R2R_oneseq Pa105.1-L2554 shade_along_backbone KTURN:T rgb:0,129,255
#=GF R2R_oneseq Pa105.1-L2554 shade_along_backbone KTURN:R rgb:0,129,255
#=GF R2R_oneseq Pa105.1-L2554 tick_label KTURNL:r K-turn
#=GF R2R_oneseq Pa105.1-L2554 shade_along_backbone ILOOP:Y rgb:0,255,0
#=GF R2R_oneseq Pa105.1-L2554 shade_along_backbone ILOOP:S rgb:0,255,0
#=GF R2R_oneseq Pa105.1-L2554 shade_along_backbone ILOOP:U rgb:0,255,0
#=GF R2R_oneseq Pa105.1-L2554 shade_along_backbone ILOOP:T rgb:0,255,0
#=GF R2R_oneseq Pa105.1-L2554 shade_along_backbone ILOOP:P rgb:0,255,0
#=GF R2R_oneseq Pa105.1-L2554 shade_along_backbone ILOOP:Q rgb:0,255,0
#=GF R2R_oneseq Pa105.1-L2554 shade_along_backbone ILOOP:X rgb:0,255,0
#=GF R2R_oneseq Pa105.1-L2554 shade_along_backbone ILOOP:Z rgb:200,200,200
#=GF R2R_oneseq Pa105.1-L2554 tick_label ILOOPL:z ANA box

#=GF R2R_oneseq Pa105.2-S995 shade_along_backbone KTURN:T rgb:0,129,255
#=GF R2R_oneseq Pa105.2-S995 shade_along_backbone KTURN:R rgb:0,129,255
#=GF R2R_oneseq Pa105.2-S995 tick_label KTURNL:r K-turn
#=GF R2R_oneseq Pa105.2-S995 shade_along_backbone ILOOP:Y rgb:0,255,0
#=GF R2R_oneseq Pa105.2-S995 shade_along_backbone ILOOP:S rgb:0,255,0
#=GF R2R_oneseq Pa105.2-S995 shade_along_backbone ILOOP:U rgb:0,255,0
#=GF R2R_oneseq Pa105.2-S995 shade_along_backbone ILOOP:T rgb:0,255,0
#=GF R2R_oneseq Pa105.2-S995 shade_along_backbone ILOOP:P rgb:0,255,0
#=GF R2R_oneseq Pa105.2-S995 shade_along_backbone ILOOP:Q rgb:0,255,0
#=GF R2R_oneseq Pa105.2-S995 shade_along_backbone ILOOP:X rgb:0,255,0
#=GF R2R_oneseq Pa105.2-S995 shade_along_backbone ILOOP:Z rgb:200,200,200
#=GF R2R_oneseq Pa105.2-S995 tick_label ILOOPL:z ANA box

#=GF R2R_oneseq Pa105.2-L2794 shade_along_backbone KTURN:T rgb:0,129,255
#=GF R2R_oneseq Pa105.2-L2794 shade_along_backbone KTURN:R rgb:0,129,255
#=GF R2R_oneseq Pa105.2-L2794 tick_label KTURNL:r K-turn
#=GF R2R_oneseq Pa105.2-L2794 shade_along_backbone ILOOP:Y rgb:0,255,0
#=GF R2R_oneseq Pa105.2-L2794 shade_along_backbone ILOOP:S rgb:0,255,0
#=GF R2R_oneseq Pa105.2-L2794 shade_along_backbone ILOOP:U rgb:0,255,0
#=GF R2R_oneseq Pa105.2-L2794 shade_along_backbone ILOOP:T rgb:0,255,0
#=GF R2R_oneseq Pa105.2-L2794 shade_along_backbone ILOOP:P rgb:0,255,0
#=GF R2R_oneseq Pa105.2-L2794 shade_along_backbone ILOOP:Q rgb:0,255,0
#=GF R2R_oneseq Pa105.2-L2794 shade_along_backbone ILOOP:X rgb:0,255,0
#=GF R2R_oneseq Pa105.2-L2794 shade_along_backbone ILOOP:Z rgb:200,200,200
#=GF R2R_oneseq Pa105.2-L2794 tick_label ILOOPL:z ANA box

#=GF R2R_oneseq Pa105.2-L1377 shade_along_backbone KTURN:T rgb:0,129,255
#=GF R2R_oneseq Pa105.2-L1377 shade_along_backbone KTURN:R rgb:0,129,255
#=GF R2R_oneseq Pa105.2-L1377 tick_label KTURNL:r K-turn
#=GF R2R_oneseq Pa105.2-L1377 shade_along_backbone ILOOP:Y rgb:0,255,0
#=GF R2R_oneseq Pa105.2-L1377 shade_along_backbone ILOOP:S rgb:0,255,0
#=GF R2R_oneseq Pa105.2-L1377 shade_along_backbone ILOOP:U rgb:0,255,0
#=GF R2R_oneseq Pa105.2-L1377 shade_along_backbone ILOOP:T rgb:0,255,0
#=GF R2R_oneseq Pa105.2-L1377 shade_along_backbone ILOOP:P rgb:0,255,0
#=GF R2R_oneseq Pa105.2-L1377 shade_along_backbone ILOOP:Q rgb:0,255,0
#=GF R2R_oneseq Pa105.2-L1377 shade_along_backbone ILOOP:X rgb:0,255,0
#=GF R2R_oneseq Pa105.2-L1377 shade_along_backbone ILOOP:Z rgb:200,200,200
#=GF R2R_oneseq Pa105.2-L1377 tick_label ILOOPL:z ANA box

#=GF R2R_oneseq Pa105.2-S27 shade_along_backbone KTURN:T rgb:0,129,255
#=GF R2R_oneseq Pa105.2-S27 shade_along_backbone KTURN:R rgb:0,129,255
#=GF R2R_oneseq Pa105.2-S27 tick_label KTURNL:r K-turn
#=GF R2R_oneseq Pa105.2-S27 shade_along_backbone ILOOP:Y rgb:0,255,0
#=GF R2R_oneseq Pa105.2-S27 shade_along_backbone ILOOP:S rgb:0,255,0
#=GF R2R_oneseq Pa105.2-S27 shade_along_backbone ILOOP:U rgb:0,255,0
#=GF R2R_oneseq Pa105.2-S27 shade_along_backbone ILOOP:T rgb:0,255,0
#=GF R2R_oneseq Pa105.2-S27 shade_along_backbone ILOOP:P rgb:0,255,0
#=GF R2R_oneseq Pa105.2-S27 shade_along_backbone ILOOP:Q rgb:0,255,0
#=GF R2R_oneseq Pa105.2-S27 shade_along_backbone ILOOP:X rgb:0,255,0
#=GF R2R_oneseq Pa105.2-S27 shade_along_backbone ILOOP:Z rgb:200,200,200
#=GF R2R_oneseq Pa105.2-S27 tick_label ILOOPL:z ANA box

#=GF R2R_oneseq Pa160-S922 shade_along_backbone KTURN:T rgb:0,129,255
#=GF R2R_oneseq Pa160-S922 shade_along_backbone KTURN:R rgb:0,129,255
#=GF R2R_oneseq Pa160-S922 tick_label KTURNL:r K-turn
#=GF R2R_oneseq Pa160-S922 shade_along_backbone ILOOP:Y rgb:0,255,0
#=GF R2R_oneseq Pa160-S922 shade_along_backbone ILOOP:S rgb:0,255,0
#=GF R2R_oneseq Pa160-S922 shade_along_backbone ILOOP:U rgb:0,255,0
#=GF R2R_oneseq Pa160-S922 shade_along_backbone ILOOP:T rgb:0,255,0
#=GF R2R_oneseq Pa160-S922 shade_along_backbone ILOOP:P rgb:0,255,0
#=GF R2R_oneseq Pa160-S922 shade_along_backbone ILOOP:Q rgb:0,255,0
#=GF R2R_oneseq Pa160-S922 shade_along_backbone ILOOP:X rgb:0,255,0
#=GF R2R_oneseq Pa160-S922 shade_along_backbone ILOOP:Z rgb:200,200,200
#=GF R2R_oneseq Pa160-S922 tick_label ILOOPL:z ANA box
```

```
#=GF R2R_oneseq Pa160-L2672 shade_along_backbone KTURN:T rgb:0,129,255
#=GF R2R_oneseq Pa160-L2672 shade_along_backbone KTURN:R rgb:0,129,255
#=GF R2R_oneseq Pa160-L2672 tick_label KTURNL:r K-turn
#=GF R2R_oneseq Pa160-L2672 shade_along_backbone ILOOP:Y rgb:0,255,0
#=GF R2R_oneseq Pa160-L2672 shade_along_backbone ILOOP:S rgb:0,255,0
#=GF R2R_oneseq Pa160-L2672 shade_along_backbone ILOOP:U rgb:0,255,0
#=GF R2R_oneseq Pa160-L2672 shade_along_backbone ILOOP:T rgb:0,255,0
#=GF R2R_oneseq Pa160-L2672 shade_along_backbone ILOOP:P rgb:0,255,0
#=GF R2R_oneseq Pa160-L2672 shade_along_backbone ILOOP:Q rgb:0,255,0
#=GF R2R_oneseq Pa160-L2672 shade_along_backbone ILOOP:X rgb:0,255,0
#=GF R2R_oneseq Pa160-L2672 shade_along_backbone ILOOP:Z rgb:200,200,200
#=GF R2R_oneseq Pa160-L2672 tick_label ILOOPL:z ANA box

//
```

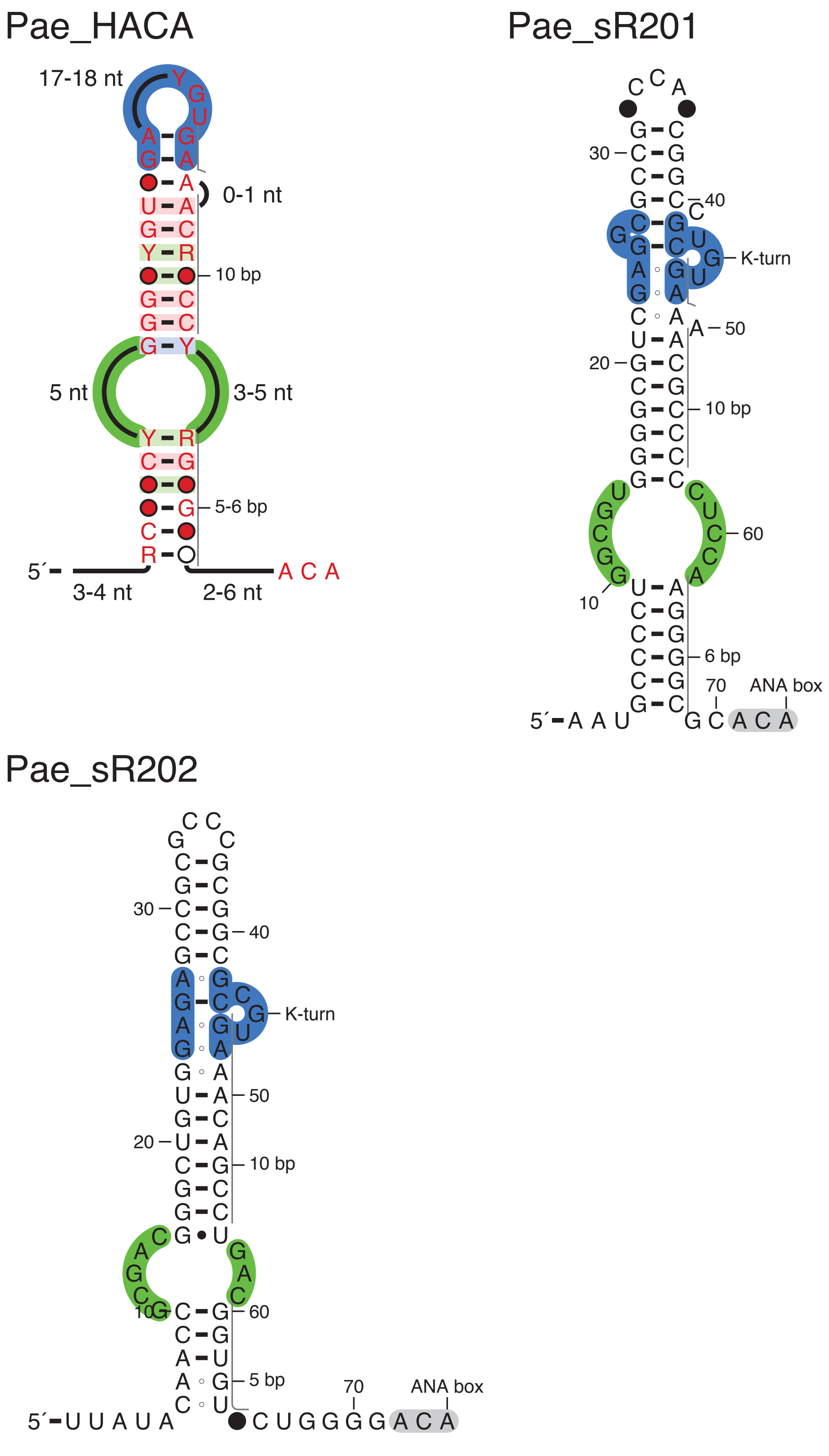

Figure S2. Structure/Function Model of H/ACA guide RNAs in *Pyrobaculum aerophilum* from Bernick *et al* (16).

Listing 2: Stockholm Aligmnent used in R2R representations of the Structure/Function Model of H/ACA guide RNAs in *Pyrobaculum aerophilum* (Fig. S2).

```
# STOCKHOLM 1.0

Pae_sR201                    AA.UGCCCCU.......GGCGUG.GGGCGUC...GA....GGCGCCG....CCA......CGGCCG.C...UGUGA.AAAC.GC.CCC.CUCCAA.GGGGCGC......ACA
Pae_sR202                    UUAUACAACC.......GCGACG.GGCUGUG...GA....G.AGCCGC..GCCC.....GCGGC.G.C...CGUGA.A.AC.AG.CCU...GACG.GUGU.CUGGGG..ACA
#=GC SS_cons                 ....<<<<<<............<-<<<<<<<...<<....<.<<<<<<___________>>>>>.>.>......>>.>->>->>->>>.....->->>>>>...........
#=GC R2R_LABEL               a..b..........1......2.3o.....v...II...4........................j...5.....HH.wlHH.HiBtp.6....7H9HHhHHc....d.....
#=GC R2R_XLABEL_KTURN        ..................................TTTTTTTTT......................RRRRRRRRRRR....................................
#=GC R2R_XLABEL_KTURNL       ........................................................................r.......................................
#=GC R2R_XLABEL_ILOOP        ...............PQXXXXX.W................................................................YYYYSU.V.............ZZZ
#=GC R2R_XLABEL_ILOOPL       ...................x...w...................................................................ysu.v...............z
#=GC R2R_XLABEL_STEM         ................................................................................................................
#=GC R2R_XLABEL_STEML        ................................................................................................................

#=GF R2R keep allpairs
#=GF R2R var_backbone_range_size_fake_nucs 5 1 2
#=GF R2R var_backbone_range_size_fake_nucs 4 6 7
#=GF R2R var_backbone_range 4 5
#=GF R2R var_backbone_range_size_fake_nucs 1 l l
#=GF R2R var_backbone_range_size_fake_nucs 4 a b
#=GF R2R var_backbone_range_size_fake_nucs 6 c d

#=GF R2R shade_along_backbone 1 rgb:0,255,0
#=GF R2R shade_along_backbone 6 rgb:0,255,0
#=GF R2R shade_along_backbone KTURN:T rgb:0,129,255
#=GF R2R shade_along_backbone KTURN:R rgb:0,129,255
#=GF R2R outline_nuc H
#=GF R2R outline_nuc h
#=GF R2R outline_nuc w
#=GF R2R outline_nuc p
#=GF R2R outline_nuc i
#=GF R2R outline_nuc t
#=GF R2R tick_label h 5-6 bp
#=GF R2R tick_label i 10 bp

#=GF R2R_oneseq Pae_sR201 shade_along_backbone KTURN:T rgb:0,129,255
#=GF R2R_oneseq Pae_sR201 shade_along_backbone KTURN:R rgb:0,129,255
#=GF R2R_oneseq Pae_sR201 tick_label KTURNL:r K-turn
#=GF R2R_oneseq Pae_sR201 shade_along_backbone ILOOP:Y rgb:0,255,0
#=GF R2R_oneseq Pae_sR201 shade_along_backbone ILOOP:S rgb:0,255,0
#=GF R2R_oneseq Pae_sR201 shade_along_backbone ILOOP:U rgb:0,255,0
#=GF R2R_oneseq Pae_sR201 shade_along_backbone ILOOP:P rgb:0,255,0
#=GF R2R_oneseq Pae_sR201 shade_along_backbone ILOOP:Q rgb:0,255,0
#=GF R2R_oneseq Pae_sR201 shade_along_backbone ILOOP:X rgb:0,255,0
#=GF R2R_oneseq Pae_sR201 shade_along_backbone ILOOP:Z rgb:200,200,200
#=GF R2R_oneseq Pae_sR201 tick_label ILOOPL:z ANA box
#=GF R2R_oneseq Pae_sR201 var_backbone_range_size_fake_nucs 1 j j 0-1 nt

#=GF R2R_oneseq Pae_sR202 shade_along_backbone KTURN:T rgb:0,129,255
#=GF R2R_oneseq Pae_sR202 shade_along_backbone KTURN:R rgb:0,129,255
#=GF R2R_oneseq Pae_sR202 tick_label KTURNL:r K-turn
#=GF R2R_oneseq Pae_sR202 shade_along_backbone ILOOP:Y rgb:0,255,0
#=GF R2R_oneseq Pae_sR202 shade_along_backbone ILOOP:S rgb:0,255,0
#=GF R2R_oneseq Pae_sR202 shade_along_backbone ILOOP:U rgb:0,255,0
#=GF R2R_oneseq Pae_sR202 shade_along_backbone ILOOP:P rgb:0,255,0
#=GF R2R_oneseq Pae_sR202 shade_along_backbone ILOOP:Q rgb:0,255,0
#=GF R2R_oneseq Pae_sR202 shade_along_backbone ILOOP:X rgb:0,255,0
#=GF R2R_oneseq Pae_sR202 shade_along_backbone ILOOP:Z rgb:200,200,200
#=GF R2R_oneseq Pae_sR202 tick_label ILOOPL:z ANA box
#=GF R2R_oneseq Pae_sR202 var_backbone_range_size_fake_nucs 1 j j 0-1 nt

//
```

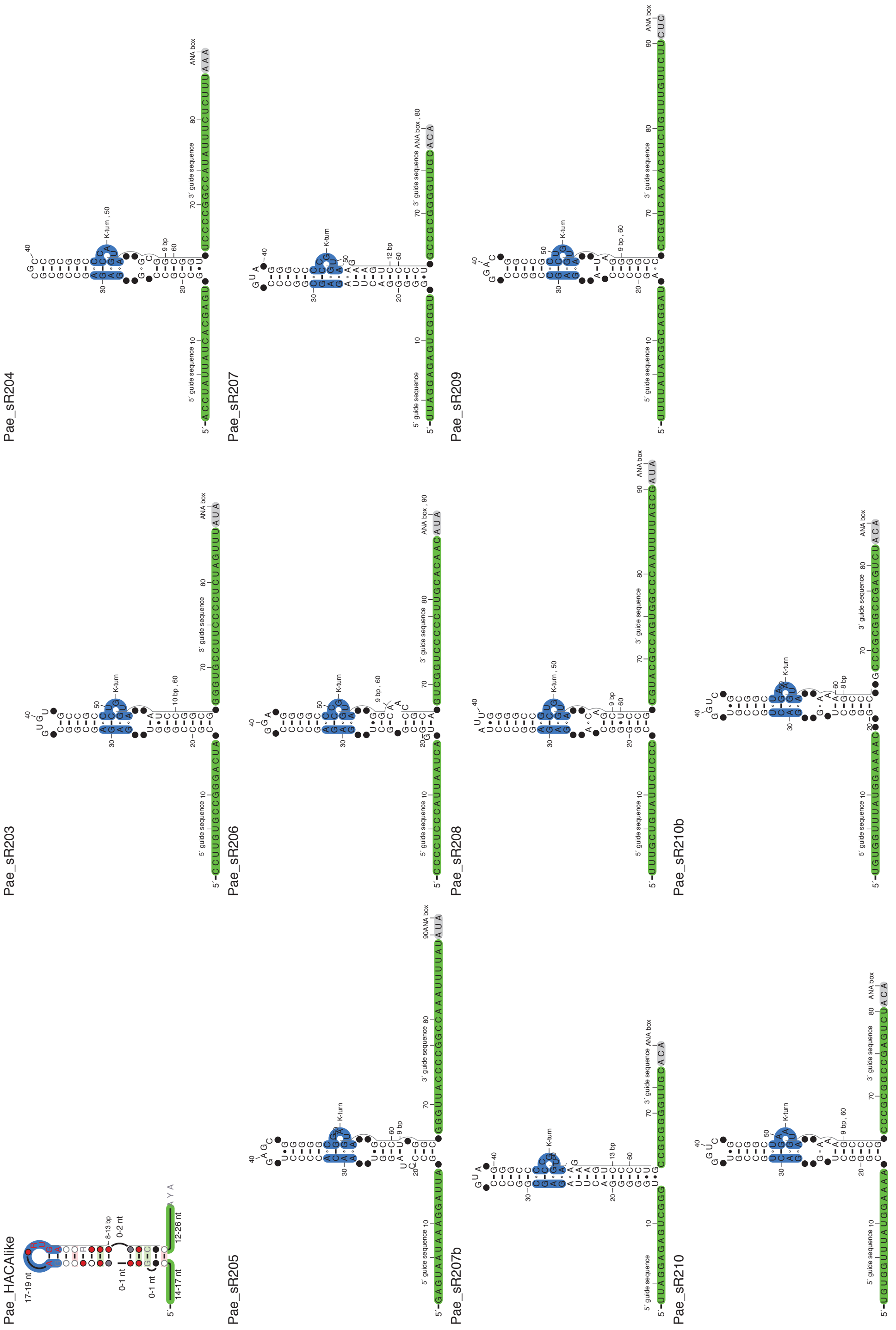

Figure S3. Structure/Function Model of H/ACA-like guide RNAs in *Pyrobaculum aerophilum* from Bernick *et al* (16).

Listing 3: Stockholm Aligmnent used in R2R representations of the Structure/Function Model of H/ACA-like guide RNAs in *Pyrobaculum aerophilum* (Fig. S3).

```
# STOCKHOLM 1.0

Pae_sR203                CCUUGU...GCCG......GGACUA.C.GCC.GGGU...GA....G.AGCGGC...GUGU....G.CCGC.C.C...UGUGA....A.UC.C..G.GCG..GGG.UGC.CUUC.CCCUCUAGUUU....AUA
Pae_sR204                ACCUAU...UAUC......ACGAGU.G.CCG.CC.G...GA....G.AGCCGCG..CGC....CG.CGGC.C.C...CAUGA....G.CG.G..C.GGU..UCC.CCG.GCCA.UAUUUCUCUUU....AAA
Pae_sR205                GAGUAA...UAAA......GGAUUA.G.CCCUAGGU...AA....C.ACCCCU...GAGC....G.GGGG.C.G...GAUGA....G.CC.U....GGC..GGGUUAC.CCCG.GCCAAAUUUUAU...AUA
Pae_sR206                CCCCUC...CCAU......UAAUCA.UGGCG..GCU...GA....G.AGCCCC...GGA.....G.GGGC.C.C...CGUGA....G.GC.AACC.GCA..GUCGGUC.CCCC.UUGCACAAC......AUA
Pae_sR207                ...UUA...GGAG.....AGUCGGGU.G.GGG.GACU.UAGA....G.CGGCCC...GUA.....G.GGCC.C.C...CGUGAAGA.A.GU.C..C.CCU..GCCGCGG.GGUUGC.............ACA
Pae_sR207b               ...UUA...GGAG......AGUCGGGUG.GGG.GACU.UAGA....G.CGGCCC...GUA.....G.GGCC.C.C...CGUGAAGA.A.GU.C..C.CCUG.CCGCGG..GGUUGC.............ACA
Pae_sR208                UUUGCU...GUAU......UCUCCC.C.GGG.GC.A...GA....G.AGGCCCU..AUU....GG.GGCC.G.C...UGUGA....C.AG.C..U.CCG..CGUACGC.CAGU.GGCCCAAUUUUAGCGAUA
Pae_sR209                UUUUAU...ACGG......CAGGAU.A.GCC.CC.A...GA....G.CCGGCC...GGAC....G.GCCG.C.C...UGUGA....U.AG.G..G.GCC..CCG.GUC.AAAA.CCUCUGUUUGUUCUUCUC
Pae_sR210                UGUGGU...UUAU......GGAAAA.C.GGG.CU.G...GA....C.UGCCGU....GGUC...G.CGGC.U.G...AAUGA....A.AA.G..C.CCG..CCC.GCG.GCCG.AGUCU..........ACA
Pae_sR210b               UGUGGU...UUAU.....GGAAAAC...GGG.CU.G...GA....C.UGCCGU....GGUC...G.CGGC.U.G...AAUGA....A.AA.G..C.CC..GCCC.GCG.GCCG.AGUCU..........ACA
#=GC SS_cons             ..........................<<.<<<.<<<<.<<<<....<.<<<<<<<_________>>.>>>>.>.>......>>>.>.>.>>.>..>.>>>>...............................
#=GC R2R_LABEL           1.......2a..b3..........4..u..mvo.g....II5..............................6.....HHH.H.h.HH.istH.HHH..7......8b..a9..............0...
#=GC R2R_XLABEL_KTURN    ....................................TTTTTTTTT......................RRRRRRRRRRR................................................
#=GC R2R_XLABEL_KTURNL   ..............................................................................r...................................................
#=GC R2R_XLABEL_ILOOP    XXXXXXXXXXXXXXXXXXXXXXXXXX..........................................................................YYYYYYYYYYYYYYYYYYYYYYYYYYYYYZZZ
#=GC R2R_XLABEL_ILOOPL   .....x...........................................................................................................y..................z

#=GF R2R keep allpairs
#=GF R2R var_backbone_range 5 6
#=GF R2R var_backbone_range_size_fake_nucs 16 1 4
#=GF R2R var_backbone_range_size_fake_nucs 26 7 0
#=GF R2R var_backbone_range_size_fake_nucs 1 u u
#=GF R2R var_backbone_range_size_fake_nucs 1 v v
#=GF R2R var_backbone_range_size_fake_nucs 2 s t
#=GF R2R outline_nuc H
#=GF R2R outline_nuc h
#=GF R2R outline_nuc i
#=GF R2R tick_label i 8-13 bp
#=GF R2R shade_along_backbone KTURN:T rgb:0,129,255
#=GF R2R shade_along_backbone KTURN:R rgb:0,129,255
#=GF R2R shade_along_backbone ILOOP:Y rgb:0,255,0
#=GF R2R shade_along_backbone ILOOP:X rgb:0,255,0

#=GF R2R_oneseq Pae_sR203 shade_along_backbone KTURN:T rgb:0,129,255
#=GF R2R_oneseq Pae_sR203 shade_along_backbone KTURN:R rgb:0,129,255
#=GF R2R_oneseq Pae_sR203 tick_label KTURNL:r K-turn
#=GF R2R_oneseq Pae_sR203 shade_along_backbone ILOOP:Y rgb:0,255,0
#=GF R2R_oneseq Pae_sR203 tick_label ILOOPL:y 3' guide sequence
#=GF R2R_oneseq Pae_sR203 shade_along_backbone ILOOP:X rgb:0,255,0
#=GF R2R_oneseq Pae_sR203 tick_label ILOOPL:x 5' guide sequence
#=GF R2R_oneseq Pae_sR203 shade_along_backbone ILOOP:Z rgb:200,200,200
#=GF R2R_oneseq Pae_sR203 tick_label ILOOPL:z ANA box

#=GF R2R_oneseq Pae_sR204 shade_along_backbone KTURN:T rgb:0,129,255
#=GF R2R_oneseq Pae_sR204 shade_along_backbone KTURN:R rgb:0,129,255
#=GF R2R_oneseq Pae_sR204 tick_label KTURNL:r K-turn
#=GF R2R_oneseq Pae_sR204 shade_along_backbone ILOOP:Y rgb:0,255,0
#=GF R2R_oneseq Pae_sR204 tick_label ILOOPL:y 3' guide sequence
#=GF R2R_oneseq Pae_sR204 shade_along_backbone ILOOP:X rgb:0,255,0
#=GF R2R_oneseq Pae_sR204 tick_label ILOOPL:x 5' guide sequence
#=GF R2R_oneseq Pae_sR204 shade_along_backbone ILOOP:Z rgb:200,200,200
#=GF R2R_oneseq Pae_sR204 tick_label ILOOPL:z ANA box

#=GF R2R_oneseq Pae_sR205 shade_along_backbone KTURN:T rgb:0,129,255
#=GF R2R_oneseq Pae_sR205 shade_along_backbone KTURN:R rgb:0,129,255
#=GF R2R_oneseq Pae_sR205 tick_label KTURNL:r K-turn
#=GF R2R_oneseq Pae_sR205 shade_along_backbone ILOOP:Y rgb:0,255,0
#=GF R2R_oneseq Pae_sR205 tick_label ILOOPL:y 3' guide sequence
#=GF R2R_oneseq Pae_sR205 shade_along_backbone ILOOP:X rgb:0,255,0
#=GF R2R_oneseq Pae_sR205 tick_label ILOOPL:x 5' guide sequence
#=GF R2R_oneseq Pae_sR205 shade_along_backbone ILOOP:Z rgb:200,200,200
#=GF R2R_oneseq Pae_sR205 tick_label ILOOPL:z ANA box

#=GF R2R_oneseq Pae_sR206 shade_along_backbone KTURN:T rgb:0,129,255
#=GF R2R_oneseq Pae_sR206 shade_along_backbone KTURN:R rgb:0,129,255
#=GF R2R_oneseq Pae_sR206 tick_label KTURNL:r K-turn
#=GF R2R_oneseq Pae_sR206 shade_along_backbone ILOOP:Y rgb:0,255,0
#=GF R2R_oneseq Pae_sR206 tick_label ILOOPL:y 3' guide sequence
#=GF R2R_oneseq Pae_sR206 shade_along_backbone ILOOP:X rgb:0,255,0
#=GF R2R_oneseq Pae_sR206 tick_label ILOOPL:x 5' guide sequence
#=GF R2R_oneseq Pae_sR206 shade_along_backbone ILOOP:Z rgb:200,200,200
#=GF R2R_oneseq Pae_sR206 tick_label ILOOPL:z ANA box

#=GF R2R_oneseq Pae_sR207 shade_along_backbone KTURN:T rgb:0,129,255
#=GF R2R_oneseq Pae_sR207 shade_along_backbone KTURN:R rgb:0,129,255
#=GF R2R_oneseq Pae_sR207 tick_label KTURNL:r K-turn
#=GF R2R_oneseq Pae_sR207 shade_along_backbone ILOOP:Y rgb:0,255,0
#=GF R2R_oneseq Pae_sR207 tick_label ILOOPL:y 3' guide sequence
#=GF R2R_oneseq Pae_sR207 shade_along_backbone ILOOP:X rgb:0,255,0
#=GF R2R_oneseq Pae_sR207 tick_label ILOOPL:x 5' guide sequence
#=GF R2R_oneseq Pae_sR207 shade_along_backbone ILOOP:Z rgb:200,200,200
#=GF R2R_oneseq Pae_sR207 tick_label ILOOPL:z ANA box

#=GF R2R_oneseq Pae_sR207b shade_along_backbone KTURN:T rgb:0,129,255
#=GF R2R_oneseq Pae_sR207b shade_along_backbone KTURN:R rgb:0,129,255
#=GF R2R_oneseq Pae_sR207b tick_label KTURNL:r K-turn
#=GF R2R_oneseq Pae_sR207b shade_along_backbone ILOOP:Y rgb:0,255,0
#=GF R2R_oneseq Pae_sR207b tick_label ILOOPL:y 3' guide sequence
#=GF R2R_oneseq Pae_sR207b shade_along_backbone ILOOP:X rgb:0,255,0
#=GF R2R_oneseq Pae_sR207b tick_label ILOOPL:x 5' guide sequence
#=GF R2R_oneseq Pae_sR207b shade_along_backbone ILOOP:Z rgb:200,200,200
#=GF R2R_oneseq Pae_sR207b tick_label ILOOPL:z ANA box

#=GF R2R_oneseq Pae_sR208 shade_along_backbone KTURN:T rgb:0,129,255
#=GF R2R_oneseq Pae_sR208 shade_along_backbone KTURN:R rgb:0,129,255
#=GF R2R_oneseq Pae_sR208 tick_label KTURNL:r K-turn
#=GF R2R_oneseq Pae_sR208 shade_along_backbone ILOOP:Y rgb:0,255,0
#=GF R2R_oneseq Pae_sR208 tick_label ILOOPL:y 3' guide sequence
#=GF R2R_oneseq Pae_sR208 shade_along_backbone ILOOP:X rgb:0,255,0
#=GF R2R_oneseq Pae_sR208 tick_label ILOOPL:x 5' guide sequence
#=GF R2R_oneseq Pae_sR208 shade_along_backbone ILOOP:Z rgb:200,200,200
#=GF R2R_oneseq Pae_sR208 tick_label ILOOPL:z ANA box

#=GF R2R_oneseq Pae_sR209 shade_along_backbone KTURN:T rgb:0,129,255
#=GF R2R_oneseq Pae_sR209 shade_along_backbone KTURN:R rgb:0,129,255
```

```
#=GF R2R_oneseq Pae_sR209 tick_label KTURNL:r K-turn
#=GF R2R_oneseq Pae_sR209 shade_along_backbone ILOOP:Y rgb:0,255,0
#=GF R2R_oneseq Pae_sR209 tick_label ILOOPL:y 3’ guide sequence
#=GF R2R_oneseq Pae_sR209 shade_along_backbone ILOOP:X rgb:0,255,0
#=GF R2R_oneseq Pae_sR209 tick_label ILOOPL:x 5’ guide sequence
#=GF R2R_oneseq Pae_sR209 shade_along_backbone ILOOP:Z rgb:200,200,200
#=GF R2R_oneseq Pae_sR209 tick_label ILOOPL:z ANA box

#=GF R2R_oneseq Pae_sR210 shade_along_backbone KTURN:T rgb:0,129,255
#=GF R2R_oneseq Pae_sR210 shade_along_backbone KTURN:R rgb:0,129,255
#=GF R2R_oneseq Pae_sR210 tick_label KTURNL:r K-turn
#=GF R2R_oneseq Pae_sR210 shade_along_backbone ILOOP:Y rgb:0,255,0
#=GF R2R_oneseq Pae_sR210 tick_label ILOOPL:y 3’ guide sequence
#=GF R2R_oneseq Pae_sR210 shade_along_backbone ILOOP:X rgb:0,255,0
#=GF R2R_oneseq Pae_sR210 tick_label ILOOPL:x 5’ guide sequence
#=GF R2R_oneseq Pae_sR210 shade_along_backbone ILOOP:Z rgb:200,200,200
#=GF R2R_oneseq Pae_sR210 tick_label ILOOPL:z ANA box

#=GF R2R_oneseq Pae_sR210b shade_along_backbone KTURN:T rgb:0,129,255
#=GF R2R_oneseq Pae_sR210b shade_along_backbone KTURN:R rgb:0,129,255
#=GF R2R_oneseq Pae_sR210b tick_label KTURNL:r K-turn
#=GF R2R_oneseq Pae_sR210b shade_along_backbone ILOOP:Y rgb:0,255,0
#=GF R2R_oneseq Pae_sR210b tick_label ILOOPL:y 3’ guide sequence
#=GF R2R_oneseq Pae_sR210b shade_along_backbone ILOOP:X rgb:0,255,0
#=GF R2R_oneseq Pae_sR210b tick_label ILOOPL:x 5’ guide sequence
#=GF R2R_oneseq Pae_sR210b shade_along_backbone ILOOP:Z rgb:200,200,200
#=GF R2R_oneseq Pae_sR210b tick_label ILOOPL:z ANA box

//
```

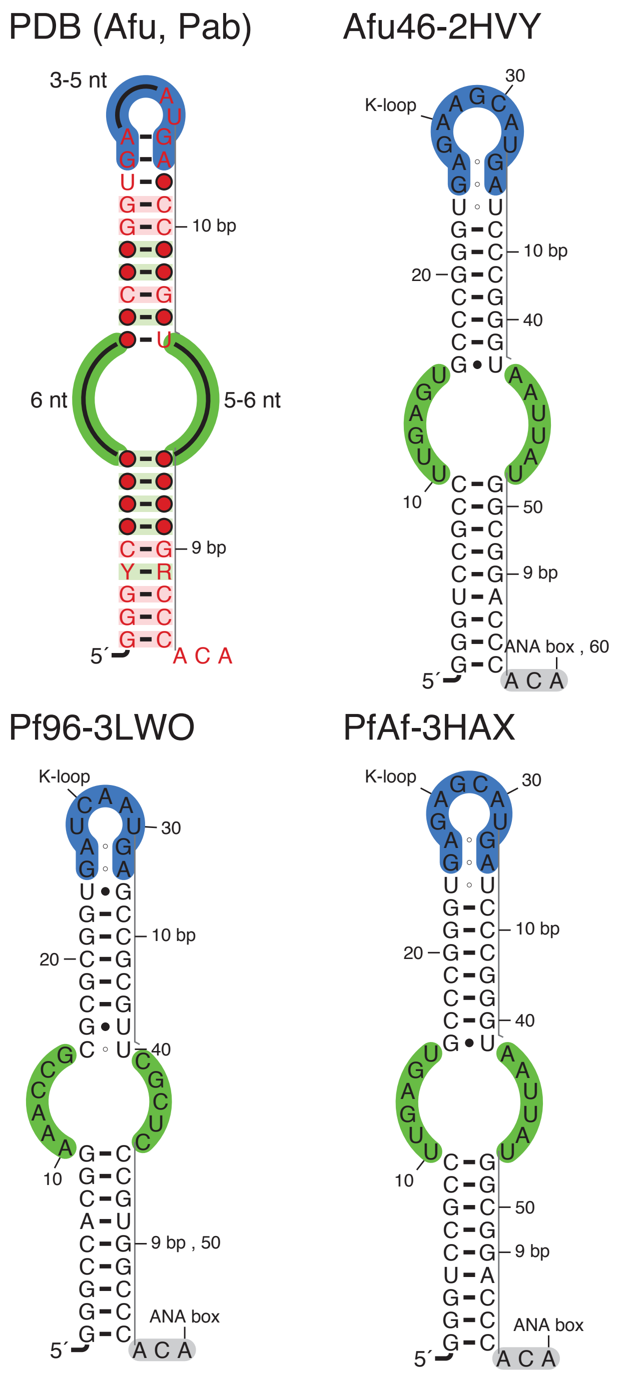

Figure S4. Structure/Function Model of H/ACA chimeric guide RNAs from *Pyrococcus furiosus* and *Archaeaglobus fulgidus* as extracted from the 3D structures (PDB IDs: 2HVY (17), 3HAX (18), 3LWO (19)).

Listing 4: Stockholm Aligmnent used in R2R representations of the Structure/Function Model of H/ACA chimeric guide RNAs from *Pyrococcus furiosus* and *Archaeaglobus fulgidus* (Fig. S4).

```
# STOCKHOLM 1.0

Pf96-3LWO                     GGGCCACGG......AAACCGC.GCGCGGU...GA..............UCA.................AUGA.GCCG.CG.UUCGCUC..C.CGUGGCCC..ACA
PfAf-3HAX                     GGGUCCGCC......UUGAGUG.CCCGGGU...GA..............GAGC................AUGA.UCCC.GG.GUAAUUAU.G.GCGGACCC..ACA
Afu46-2HVY                    GGGUCCGCC......UUGAGUG.CCCGGGU...GA..............GAAGC...............AUGA.UCCC.GG.GUAAUUAU.G.GCGGACCC..ACA
#=GC SS_cons                  <<<<<<<<<............<-<<<<<<<...<<....__________________________......>>.>>>>->>->>......->->>>>>>>>.....
#=GC R2R_LABEL                ...............1....2.3..........II...4..........................5.....HH.HHiH.HHBHH6....78H9HHHhHHHH.....
#=GC R2R_XLABEL_KLOOP         .................................KKKKKKKKKKKKKKKKKKKKKKKKKKKKKKKKKKKKKKKKK.................................
#=GC R2R_XLABEL_KLOOPL        ..................................................k.......................................................
#=GC R2R_XLABEL_ILOOP         ..............PQXXXXX.W.............................................................YYYYYSU.V..........ZZZ
#=GC R2R_XLABEL_ILOOPL        .................x....w................................................................y.su.v............z
#=GC R2R_XLABEL_STEM          ..........................................................................................................
#=GC R2R_XLABEL_STEML         ..........................................................................................................

#=GF R2R keep allpairs
#=GF R2R var_backbone_range_size_fake_nucs 6 1 2
#=GF R2R var_backbone_range_size_fake_nucs 6 6 7
#=GF R2R var_backbone_range 4 5
#=GF R2R shade_along_backbone 1 rgb:0,255,0
#=GF R2R shade_along_backbone 6 rgb:0,255,0
#=GF R2R shade_along_backbone KLOOP:K rgb:0,129,255
#=GF R2R outline_nuc H
#=GF R2R outline_nuc h
#=GF R2R outline_nuc i
#=GF R2R tick_label h 9 bp
#=GF R2R tick_label i 10 bp

#=GF R2R_oneseq Pf96-3LWO shade_along_backbone KLOOP:K rgb:0,129,255
#=GF R2R_oneseq Pf96-3LWO tick_label KLOOPL:k K-loop
#=GF R2R_oneseq Pf96-3LWO shade_along_backbone ILOOP:Y rgb:0,255,0
#=GF R2R_oneseq Pf96-3LWO shade_along_backbone ILOOP:S rgb:0,255,0
#=GF R2R_oneseq Pf96-3LWO shade_along_backbone ILOOP:U rgb:0,255,0
#=GF R2R_oneseq Pf96-3LWO shade_along_backbone ILOOP:P rgb:0,255,0
#=GF R2R_oneseq Pf96-3LWO shade_along_backbone ILOOP:Q rgb:0,255,0
#=GF R2R_oneseq Pf96-3LWO shade_along_backbone ILOOP:X rgb:0,255,0
#=GF R2R_oneseq Pf96-3LWO shade_along_backbone ILOOP:Z rgb:200,200,200
#=GF R2R_oneseq Pf96-3LWO tick_label ILOOPL:z ANA box

#=GF R2R_oneseq PfAf-3HAX shade_along_backbone KLOOP:K rgb:0,129,255
#=GF R2R_oneseq PfAf-3HAX tick_label KLOOPL:k K-loop
#=GF R2R_oneseq PfAf-3HAX shade_along_backbone ILOOP:Y rgb:0,255,0
#=GF R2R_oneseq PfAf-3HAX shade_along_backbone ILOOP:S rgb:0,255,0
#=GF R2R_oneseq PfAf-3HAX shade_along_backbone ILOOP:U rgb:0,255,0
#=GF R2R_oneseq PfAf-3HAX shade_along_backbone ILOOP:P rgb:0,255,0
#=GF R2R_oneseq PfAf-3HAX shade_along_backbone ILOOP:Q rgb:0,255,0
#=GF R2R_oneseq PfAf-3HAX shade_along_backbone ILOOP:X rgb:0,255,0
#=GF R2R_oneseq PfAf-3HAX shade_along_backbone ILOOP:Z rgb:200,200,200
#=GF R2R_oneseq PfAf-3HAX tick_label ILOOPL:z ANA box

#=GF R2R_oneseq Afu46-2HVY shade_along_backbone KLOOP:K rgb:0,129,255
#=GF R2R_oneseq Afu46-2HVY tick_label KLOOPL:k K-loop
#=GF R2R_oneseq Afu46-2HVY shade_along_backbone ILOOP:Y rgb:0,255,0
#=GF R2R_oneseq Afu46-2HVY shade_along_backbone ILOOP:S rgb:0,255,0
#=GF R2R_oneseq Afu46-2HVY shade_along_backbone ILOOP:U rgb:0,255,0
#=GF R2R_oneseq Afu46-2HVY shade_along_backbone ILOOP:P rgb:0,255,0
#=GF R2R_oneseq Afu46-2HVY shade_along_backbone ILOOP:Q rgb:0,255,0
#=GF R2R_oneseq Afu46-2HVY shade_along_backbone ILOOP:X rgb:0,255,0
#=GF R2R_oneseq Afu46-2HVY shade_along_backbone ILOOP:Z rgb:200,200,200
#=GF R2R_oneseq Afu46-2HVY tick_label ILOOPL:z ANA box

//
```

Listing 5: RNAMotif descriptor used to identify H/ACA (K-turn) Motifs in *Pyrobaculum* similar to *Pae* sR201 & sR202

```
parms
        wc += gu;
descr
        h5( minlen=5, maxlen=6 )
            ss( minlen=5, maxlen=8 )
            h5( minlen=7, maxlen=8, mispair=2, ends='mm' )
                    ss( len=4,seq="RANN" )
                            h5( minlen=2, maxlen=8 )
                                ss( minlen=3, maxlen=9 )
                            h3
                        ss( minlen=6, maxlen=7, seq="uga$" )
            h3
            ss( minlen=3, maxlen=7 )
        h3
        ss( minlen=3, maxlen=8, seq="ana$" )
```

Listing 6: RNAMotif descriptor used to identify H/ACA (K-turn) Motifs in *Pyrobaculum* similar to *Pae* sR203

```
parms
     wc += gu;
descr
     ss ( len=10 )
     h5 ( len=7, mispair=1, ends='pm')
     h5 ( len=1, pair+={"c:a","a:c","g:a","a:g","a:a"} )
        ss ( len=4, seq="rann" )
           h5 ( len=5 )
                    ss ( minlen=3, maxlen=4 )
           h3
        ss ( minlen=6, maxlen=7, seq="uga$" )
     h3
     h3
     ss ( len=18 )
```

Listing 7: RNAMotif descriptor used to identify H/ACA (K-turn) Motifs in *Pyrobaculum* similar to *Pae* sR204, sR208, sR209, sR210

```
parms
     wc += gu;
descr
     ss ( len=10 )
     h5 ( len=6, mispair=1, ends='pm' )
           h5 ( len=1, pair+={"c:a","a:c","g:a","a:g","a:a","g:g"} )
           ss ( len=4, seq="rann" )
           h5 ( minlen=5, maxlen=6 )
                    ss ( minlen=3, maxlen=4 )
           h3
           ss ( minlen=6, maxlen=7, seq="uga$" )
           h3
           ss ( minlen=0, maxlen=1 )
     h3
     ss ( len=18 )
```

Listing 8: RNAMotif descriptor used to identify H/ACA (K-turn) Motifs in *Pyrobaculum* similar to *Pae* sR205

```
parms
     wc += gu;
descr
     ss ( len=10 )
     h5 ( len=3, mispair=1, ends='pm' )
        ss ( minlen=0, maxlen=2 )
        h5 ( len=3 )
        h5 ( len=1, pair+={"c:a","a:c","g:a","a:g","a:a","g:g"} )
           ss ( len=4, seq="rann" )
           h5 ( minlen=5, maxlen=6 )
                    ss ( minlen=3, maxlen=4 )
           h3
           ss ( minlen=6, maxlen=7, seq="uga$" )
        h3
        h3
     h3
     ss ( len=18 )
```

Listing 9: RNAMotif descriptor used to identify H/ACA (K-turn) Motifs in *Pyrobaculum* similar to *Pae* sR206

```
parms
     wc += gu;
descr
     ss ( len=10 )
     h5 ( len=1, mispair=1 )
        ss ( minlen=0, maxlen=1 )
        h5 ( len=3 )
        h5 ( len=2 )
        h5 ( len=1, pair+={"c:a","a:c","g:a","a:g","a:a","g:g"} )
           ss ( len=4, seq="rann" )
           h5 ( minlen=5, maxlen=6 )
                    ss ( minlen=3, maxlen=4 )
           h3
           ss ( minlen=6, maxlen=7, seq="uga$" )
        h3
        h3
        ss ( minlen=0, maxlen=3 )
        h3
     h3
     ss ( len=18 )
```

Listing 10: RNAMotif descriptor used to identify H/ACA (K-turn) Motifs in *Pyrobaculum* similar to *Pae* sR207

```
parms
     wc += gu;
descr
     ss ( len=10 )
     h5 ( len=9, mispair=1, ends='pm')
     h5 ( len=1, pair+={"c:a","a:c","g:a","a:g","a:a"} )
        ss ( len=4, seq="rann" )
           h5 ( len=5 )
                    ss ( minlen=3, maxlen=4 )
           h3
        ss ( minlen=6, maxlen=7, seq="uga$" )
     h3
     ss ( minlen=0, maxlen=1 )
     h3
     ss ( len=18 )
```

Listing 11: RNAMotif descriptor used to identify H/ACA (K-loop) Motifs in *Pyrobaculum* similar to *Pae* sR201 & sR202

```
parms
        wc += gu;
descr
        h5( minlen=5, maxlen=6 )
            ss( minlen=5, maxlen=8 )
            h5( minlen=7, maxlen=8, mispair=2, ends='mm' )
                    ss( len=2,seq="ra" )
                                ss( minlen=4, maxlen=6 )
                    ss( len=3, seq="uga" )
            h3
            ss( minlen=3, maxlen=7 )
        h3
        ss( minlen=3, maxlen=8, seq="ana$" )
```

Listing 12: RNAMotif descriptor used to identify H/ACA (K-loop) Motifs in *Pyrobaculum* similar to *Pae* sR203

```
parms
     wc += gu;
descr
     ss ( len=10 )
     h5 ( len=7, mispair=1, ends='pm')
     h5 ( len=1, pair+={"c:a","a:c","g:a","a:g","a:a"} )
        ss ( len=2, seq="ra" )
                    ss ( minlen=4, maxlen=6 )
        ss ( len=3, seq="uga" )
     h3
     h3
     ss ( len=18 )
```

Listing 13: RNAMotif descriptor used to identify H/ACA (K-loop) Motifs in *Pyrobaculum* similar to *Pae* sR204, sR208, sR209, sR210

```
parms
     wc += gu;
descr
     ss ( len=10 )
     h5 ( len=6, mispair=1, ends='pm' )
           h5 ( len=1, pair+={"c:a","a:c","g:a","a:g","a:a","g:g"} )
           ss ( len=2, seq="ra" )
                    ss ( minlen=4, maxlen=6 )
           ss ( len=3, seq="uga" )
           h3
           ss ( minlen=0, maxlen=1 )
     h3
     ss ( len=18 )
```

Listing 14: RNAMotif descriptor used to identify H/ACA (K-loop) Motifs in *Pyrobaculum* similar to *Pae* sR205

```
parms
     wc += gu;
descr
     ss ( len=10 )
     h5 ( len=3, mispair=1, ends='pm' )
        ss ( minlen=0, maxlen=2 )
        h5 ( len=3 )
        h5 ( len=1, pair+={"c:a","a:c","g:a","a:g","a:a","g:g"} )
           ss ( len=2, seq="ra" )
                    ss ( minlen=4, maxlen=6 )
           ss ( len=3, seq="uga" )
        h3
        h3
     h3
     ss ( len=18 )
```

Listing 15: RNAMotif descriptor used to identify H/ACA (K-loop) Motifs in *Pyrobaculum* similar to *Pae* sR206

```
parms
     wc += gu;
descr
     ss ( len=10 )
     h5 ( len=1, mispair=1 )
        ss ( minlen=0, maxlen=1 )
        h5 ( len=3 )
        h5 ( len=2 )
        h5 ( len=1, pair+={"c:a","a:c","g:a","a:g","a:a","g:g"} )
           ss ( len=2, seq="ra" )
                    ss ( minlen=4, maxlen=6 )
           ss ( len=3, seq="uga" )
        h3
        h3
        ss ( minlen=0, maxlen=3 )
        h3
     h3
     ss ( len=18 )
```

Listing 16: RNAMotif descriptor used to identify H/ACA (K-loop) Motifs in *Pyrobaculum* similar to *Pae* sR207

```
parms
     wc += gu;
descr
     ss ( len=10 )
     h5 ( len=9, mispair=1, ends='pm')
     h5 ( len=1, pair+={"c:a","a:c","g:a","a:g","a:a"} )
        ss ( len=2, seq="ra" )
                 ss ( minlen=4, maxlen=6 )
        ss ( len=3, seq="uga" )
     h3
     ss ( minlen=0, maxlen=1 )
     h3
     ss ( len=18 )
```

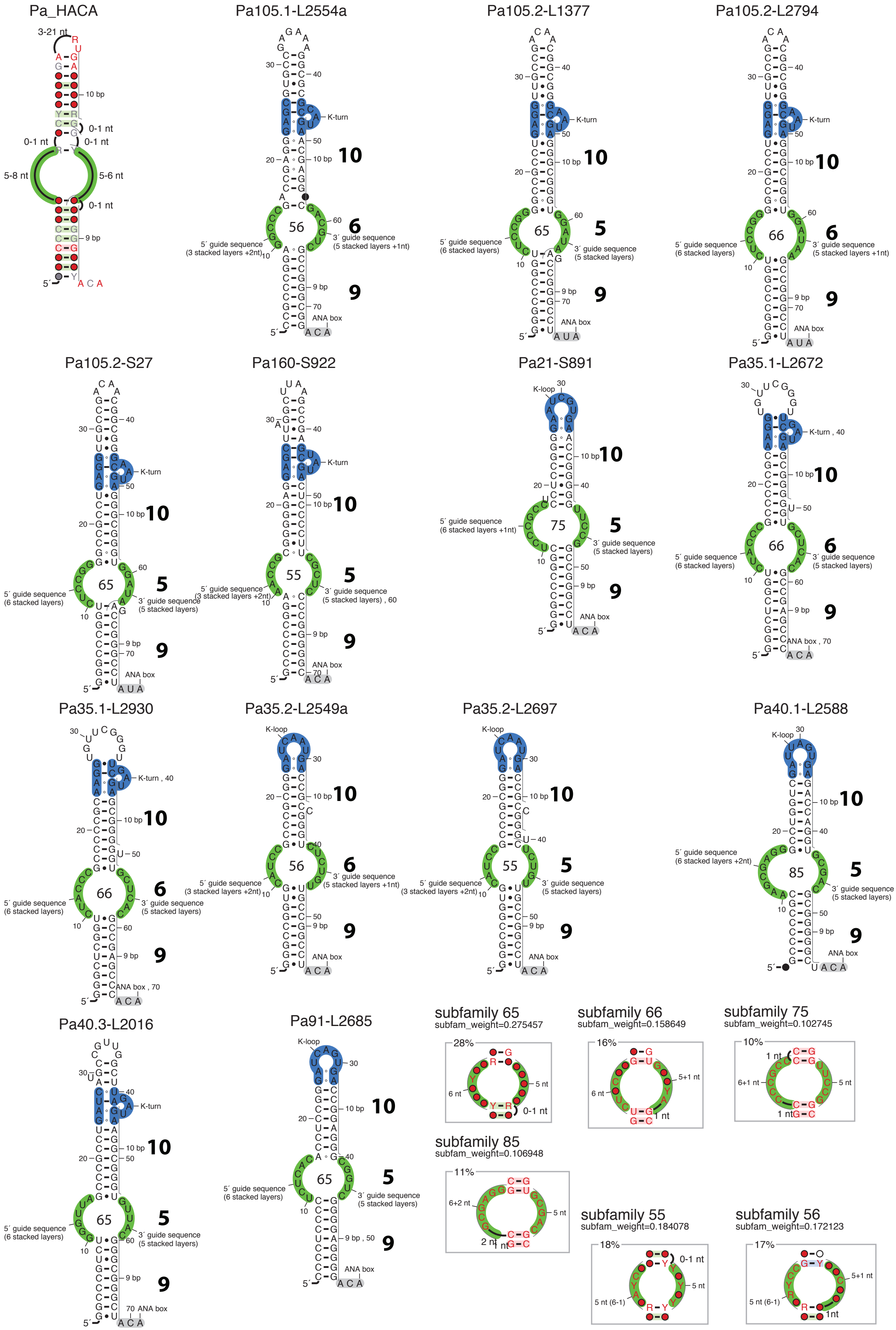

Figure S5. Structure/Function Model of H/ACA guide RNAs in *Pyrococcus abyssi*: fold family ‘10/5/9’ and its related subfamilies (55, 65, 75, 85), fold family ‘10/6/9’ and its related subfamilies (56, 66). The subfamily 65 includes: Pa91-L2685, Pa40.3-L2016, Pa105.2-L1377 (or Pa105.2-L2794, Pa105.2-S27), the subfamily 55: Pa160-S922 and Pab35.2-L2697, the subfamily 75: Pa21-S891, the subfamily 85: Pa40.1-L2588, the subfamily 66: Pa105.2-L2794 and Pa35.1-L2930 or Pab35.1-L2672, the subfamily 56: Pa35.2-L2549a and Pa105.1-L2554a. Pa35.2 can adopt two different folds: ‘10/5/9’ or ‘10/6/9’ whether the base-pair from the upper stem closing the internal loop is a G:U (Pab35.2-L2549a) or G:C (Pa35.2-L2697), respectively.

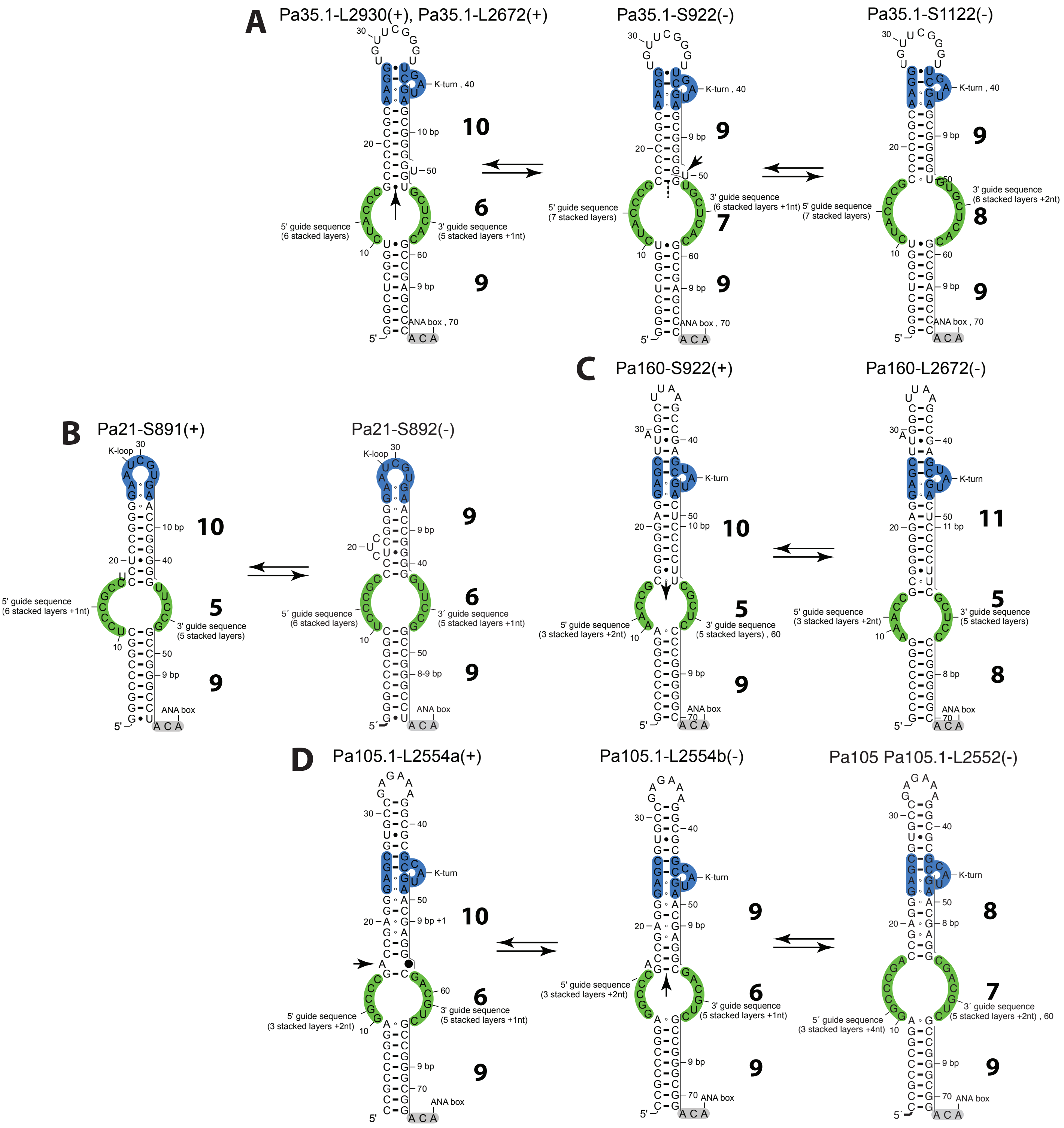

Figure S6. Relationships between RNA fold and pseudo-uridylation activity (productive/non-productive) of H/ACA guide RNAs. **A.** Productive/non-productive folds for Pa35.1 and its true (L2930, L2672) and false (S922 and S1122) targets. **B.** Productive/non-productive folds for Pa21 and its true (S891) and false (S892) targets. **C.** Productive/non-productive folds for Pa160 ants its true (S922) and false (L2672) targets. **D.** Productive/non-productive folds for Pa105.1 ands its true targets: L2554, false target: L2552. The 2D structures of the guide RNAs are shown according to the RNA fold consistent with the pairing with its associated target (the RNA targets are omitted for clarity): productive and non-productive complexes are indicated by a (+) and (-) sign, respectively. The different possible structures for a given guide RNA are shown using a double arrow. Structural changes associated with opening or closing base-pairs in the internal loop are indicated by single vertical arrows; dashed lines indicate the opening of the internal loop. Bulge nucleotides switching out/in of a stem are marked using an oblique arrow.Adding one base-pair at the bottom of the lower stem of Pa160 creates an internal loop that would accept L2672 as possible target. Pa105.1 can adopt alternative folds by opening the upper stem (10/6/9 to 9/6/9 and 8/7/9) creating an internal loop that would accept L2552 as possible target (8/7/9).

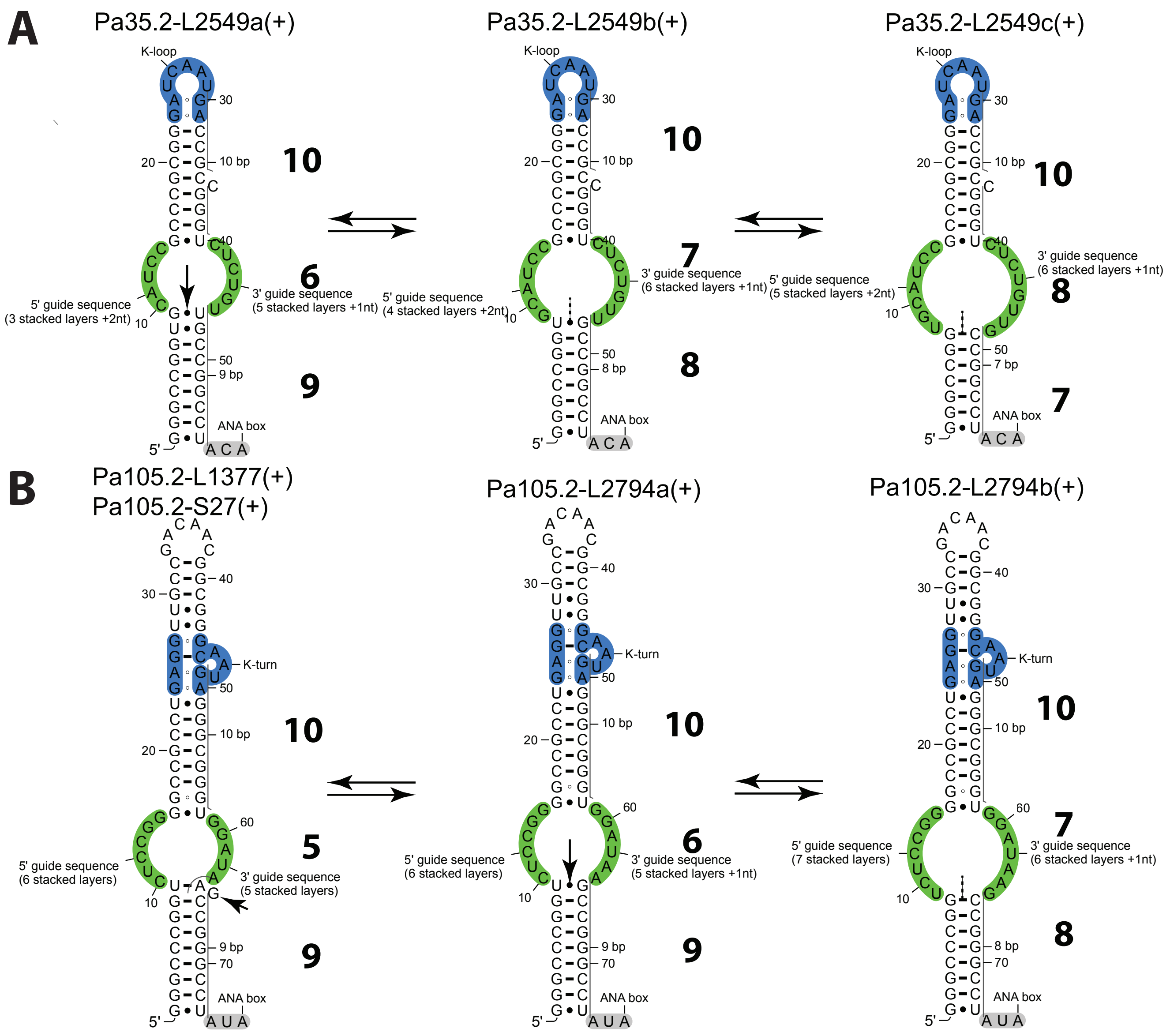

Figure S7. Models of equivalent productive folds for Pa35.2-L2549 and Pa105.2-1377, Pa105.2-S27, Pa105.2-2794.

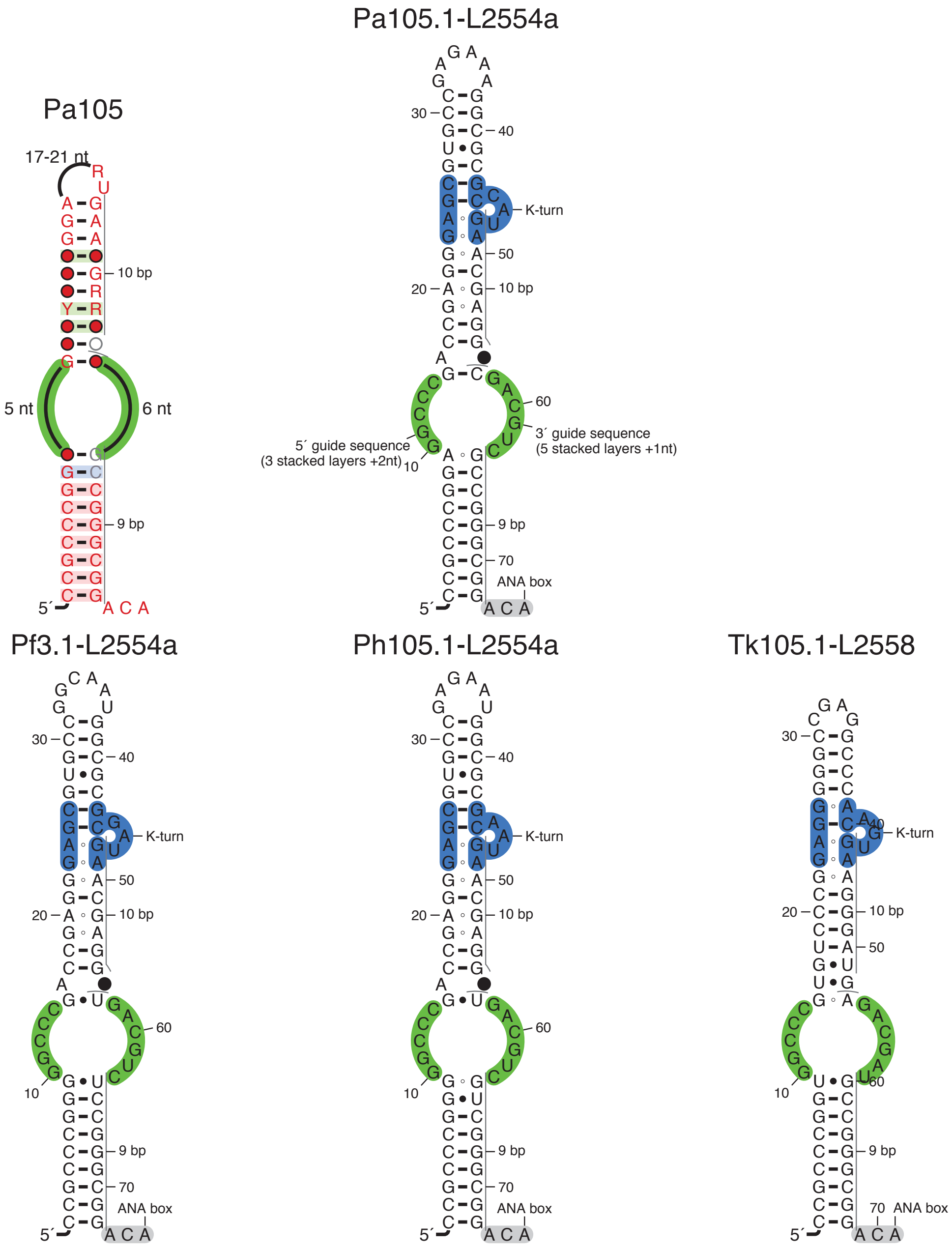

Figure S8. Structural variations in H/ACA guide RNAs between *P. abyssi* and *T. kodakaerensis*. Comparison between Pa105 motifs in *Pyrococcus* and *Thermococcus kodakaerensis*.

Listing 17: Stockholm Aligmnent used in R2R representations of the different folds and subfamilies of H/ACA guide RNAs in *Pyrococcus abyssi*.

```
# STOCKHOLM 1.0

Pa21-S891                     GGGCCCGGC.....UCCCGCCCUCUCCGGG...GA...............AUC.................GUGA.ACCG.GG.G.GUUCCG..G.CCGGGCCU..ACA
Pa91-L2685                    CCCCUCCCC......UCUCACA.CCUCCGG...GA...............UCA.................GUGA.CCGG.AG.G.GCGGUC..G.GGGAGGGG..ACA
Pa105.2-L2794                 GGGCCCGGU......CUCCGGG.GCCGCCU...GA....GGU.U.GCCG..ACAA...CGGCGGGC...AAUGA.GGGC.GG.G.UGGAUAA.G.CCGGGCCU..AUA
Pa105.2-L1377                 GGGCCCGGU......CUCCGGG.GCCGCCU...GA....GGU.U.GCCG..ACAA...CGGCGGGC...AAUGA.GGGC.GG.G.UGGAUA..AGCCGGGCCU..AUA
Pa105.2-S27                   GGGCCCGGU......CUCCGGG.GCCGCCU...GA....GGU.U.GCCG..ACAA...CGGCGGGC...AAUGA.GGGC.GG.G.UGGAUA..AGCCGGGCCU..AUA
Pa35.1-L2930                  GGGCUCGGU......CUACCCG.CCCCCGC...AA....GG........UGUUCGGGU......UC...GAUGA.GCGG.GGUG.UGCUCAC.G.CCGAGCCC..ACA
Pa35.1-L2672                  GGGCUCGGU......CUACCCG.CCCCCGC...AA....GG........UGUUCGGGU......UC...GAUGA.GCGG.GGUG.UGCUCAC.G.CCGAGCCC..ACA
Pa35.2-L2549a                 GGGCCGGUG.......CAUCCG.CCCGCGG...GA...............UCA.................AUGA.CCGCCGG.G.UCUCUGU.U.GCCGGCCU..ACA
Pa105.1-L2554a                CCGCCCGGA.......GGCCCG.ACCGAGG...GA....GCG.U.GCC.GAGAAA....GGCGCGCC...AUGA.ACGA.GG...CGACGUC.G.CCGGGCGG..ACA
Pa160-S922                    GCCCCCGGA.......AACCGC.GGGGGAG...GA....GCU.UAGGC.UUAA......GCCGAGCU...AUGA.CUCC.CC.U.UCGCUC..C.CCGGGGGC..ACA
Pa35.2-L2697                  GGGCCGGUG.......CAUCCG.CCCGCGG...GA...............UCA.................AUGA.CCGCCGG.GUCUCUGU..U.GCCGGCCU..ACA
Pa40.3-L2016                  GGCCCGUCU......GGGUUAG.CCCGCCU...GA....UCAUG.C...CGUUG.......GCUUA...GAUGA.AGGC.GG.G.UGUUAC..G.GGCGGGCU..ACA
Pa40.1-L2588                  .GCCCCCGC....AAGCGAGGG.CCUGGUC...GA...............UUA.................GUGA.GACC.AG.G.UGCGAC..G.CGGGGGCU..ACA
#=GC SS_cons                  <<<<<<<<<............<-<<<<<<<...<<....<<<.<.<<<<_________>>>>>>>>...-..>>.>>>>->>->->......->->>>>>>>>.....
#=GC R2R_LABEL                q............1......2.3s.........II...4.p..................p......5.....HH.HHiH.HHBt8H6....7.H9HHHhHHHr.....
#=GC R2R_XLABEL_KTURN         .................................TTTTTTTT.......................RRRRRRRRRR..................................
#=GC R2R_XLABEL_KTURNL        ......................................................................r.....................................
#=GC R2R_XLABEL_KLOOP         .................................KKKKKKKKKKKKKKKKKKKKKKKKKKKKKKKKKKKKKKKKK..................................
#=GC R2R_XLABEL_KLOOPL        ...................................................k........................................................
#=GC R2R_XLABEL_ILOOP         ..............PQXXXXX.W...............................................................YYYYYSU.V..........ZZZ
#=GC R2R_XLABEL_ILOOPL        .................x....w..................................................................y.su.v............z
#=GC R2R_XLABEL_STEM          ............................................................................................................
#=GC R2R_XLABEL_STEML         ............................................................................................................
#=GC SUBFAM_LABEL_TERM1       .............xxxxxxxx.......................................................................................
#=GC SUBFAM_LABEL_TERM2       ......................................................................................xxxxxxx...............
#=GC SUBFAM_GUIDE55_R2R_LABEL -------<.....1PQXxXXX.W>-----------------------------------------------------------<O.YYyYYSU.V>------------
#=GC SUBFAM_GUIDE56_R2R_LABEL -------<.....1PQXxXXX.W>-----------------------------------------------------------<O.YYyYYSU.V>------------
#=GC SUBFAM_GUIDE65_R2R_LABEL -------<.....1PQXxXXX.W>-----------------------------------------------------------<O.YYyYYSU.V>------------
#=GC SUBFAM_GUIDE66_R2R_LABEL -------<.....1PQXxXXX.W>-----------------------------------------------------------<O.YYyYYSU.V>------------
#=GC SUBFAM_GUIDE67_R2R_LABEL -------<.....1PQXxXXX.W>-----------------------------------------------------------<O.YYyYYSU.V>------------
#=GC SUBFAM_GUIDE75_R2R_LABEL -------<.....1PQXxXXX.W>-----------------------------------------------------------<O.YYyYYSU.V>------------
#=GC SUBFAM_GUIDE85_R2R_LABEL -------<.....1PQXxXXX.W>-----------------------------------------------------------<O.YYyYYSU.V>------------

#=GF R2R keep q r
#=GF R2R keep s t
#=GF R2R var_backbone_range_size_fake_nucs 7 1 2 5-8 nt
#=GF R2R var_backbone_range_size_fake_nucs 6 6 7 5-6 nt
#=GF R2R var_backbone_range 4 5
#=GF R2R var_backbone_range_size_fake_nucs 1 3 3
#=GF R2R var_backbone_range_size_fake_nucs 1 8 8
#=GF R2R var_backbone_range_size_fake_nucs 1 9 9
#=GF R2R var_backbone_range_size_fake_nucs 1 B B

#=GF SUBFAM_REGEX_PRED HAS_GUIDE5 TERM1 ^[.]*[-.][-.][-.][A-Z][A-Z][A-Z][A-Z][A-Z][.]*$
#=GF SUBFAM_REGEX_PRED HAS_GUIDE6 TERM1 ^[.]*[-.][-.][A-Z][A-Z][A-Z][A-Z][A-Z][A-Z][.]*$
#=GF SUBFAM_REGEX_PRED HAS_GUIDE7 TERM1 ^[.]*[-.][A-Z][A-Z][A-Z][A-Z][A-Z][A-Z][A-Z][.]*$
#=GF SUBFAM_REGEX_PRED HAS_GUIDE8 TERM1 ^[.]*[A-Z][A-Z][A-Z][A-Z][A-Z][A-Z][A-Z][A-Z][.]*$
#=GF SUBFAM_REGEX_PRED HAS_GUIDE6B TERM2 ^[.]*[A-Z][A-Z][A-Z][A-Z][A-Z][A-Z][-.][.]*$
#=GF SUBFAM_REGEX_PRED HAS_GUIDE7B TERM2 ^[.]*[A-Z][A-Z][A-Z][A-Z][A-Z][A-Z][A-Z][.]*$
#=GF SUBFAM_REGEX_PRED HAS_GUIDE5B TERM2 ^[.]*[A-Z][A-Z][A-Z][A-Z][A-Z][-.][-.][.]*$
#=GF SUBFAM_PERL_PRED GUIDE55 return $predValue{HAS_GUIDE5} && $predValue{HAS_GUIDE5B};
#=GF SUBFAM_PERL_PRED GUIDE56 return $predValue{HAS_GUIDE5} && $predValue{HAS_GUIDE6B};
#=GF SUBFAM_PERL_PRED GUIDE65 return $predValue{HAS_GUIDE6} && $predValue{HAS_GUIDE5B};
#=GF SUBFAM_PERL_PRED GUIDE66 return $predValue{HAS_GUIDE6} && $predValue{HAS_GUIDE6B};
#=GF SUBFAM_PERL_PRED GUIDE67 return $predValue{HAS_GUIDE6} && $predValue{HAS_GUIDE7B};
#=GF SUBFAM_PERL_PRED GUIDE75 return $predValue{HAS_GUIDE7} && $predValue{HAS_GUIDE5B};
#=GF SUBFAM_PERL_PRED GUIDE85 return $predValue{HAS_GUIDE8} && $predValue{HAS_GUIDE5B};

#=GF SUBFAM_GUIDE55_R2R no5
#=GF SUBFAM_GUIDE55_R2R set_dir pos0 -90
#=GF SUBFAM_GUIDE55_R2R shade_along_backbone ILOOP:P rgb:0,255,0
#=GF SUBFAM_GUIDE55_R2R shade_along_backbone ILOOP:Q rgb:0,255,0
#=GF SUBFAM_GUIDE55_R2R shade_along_backbone ILOOP:X rgb:0,255,0
#=GF SUBFAM_GUIDE55_R2R outline_nuc X
#=GF SUBFAM_GUIDE55_R2R outline_nuc x
#=GF SUBFAM_GUIDE55_R2R tick_label x 5 nt (6-1)
#=GF SUBFAM_GUIDE55_R2R shade_along_backbone ILOOP:Y rgb:0,255,0
#=GF SUBFAM_GUIDE55_R2R shade_along_backbone ILOOP:S rgb:0,255,0
#=GF SUBFAM_GUIDE55_R2R shade_along_backbone ILOOP:U rgb:0,255,0
#=GF SUBFAM_GUIDE55_R2R outline_nuc Y
#=GF SUBFAM_GUIDE55_R2R outline_nuc y
#=GF SUBFAM_GUIDE55_R2R tick_label y 5 nt
#=GF SUBFAM_GUIDE55_R2R var_backbone_range_size_fake_nucs 1 O O

#=GF SUBFAM_GUIDE56_R2R no5
#=GF SUBFAM_GUIDE56_R2R set_dir pos0 -90
#=GF SUBFAM_GUIDE56_R2R shade_along_backbone ILOOP:P rgb:0,255,0
#=GF SUBFAM_GUIDE56_R2R shade_along_backbone ILOOP:Q rgb:0,255,0
#=GF SUBFAM_GUIDE56_R2R shade_along_backbone ILOOP:X rgb:0,255,0
#=GF SUBFAM_GUIDE56_R2R outline_nuc X
#=GF SUBFAM_GUIDE56_R2R outline_nuc x
#=GF SUBFAM_GUIDE56_R2R tick_label x 5 nt (6-1)
#=GF SUBFAM_GUIDE56_R2R shade_along_backbone ILOOP:Y rgb:0,255,0
#=GF SUBFAM_GUIDE56_R2R shade_along_backbone ILOOP:S rgb:0,255,0
#=GF SUBFAM_GUIDE56_R2R shade_along_backbone ILOOP:U rgb:0,255,0
#=GF SUBFAM_GUIDE56_R2R outline_nuc Y
#=GF SUBFAM_GUIDE56_R2R outline_nuc y
#=GF SUBFAM_GUIDE56_R2R tick_label y 5+1 nt
#=GF SUBFAM_GUIDE56_R2R var_backbone_range_size_fake_nucs 1 S S 1nt
```

```
#=GF SUBFAM_GUIDE65_R2R no5
#=GF SUBFAM_GUIDE65_R2R set_dir pos0 -90
#=GF SUBFAM_GUIDE65_R2R shade_along_backbone ILOOP:P rgb:0,255,0
#=GF SUBFAM_GUIDE65_R2R shade_along_backbone ILOOP:Q rgb:0,255,0
#=GF SUBFAM_GUIDE65_R2R shade_along_backbone ILOOP:X rgb:0,255,0
#=GF SUBFAM_GUIDE65_R2R outline_nuc Q
#=GF SUBFAM_GUIDE65_R2R outline_nuc X
#=GF SUBFAM_GUIDE65_R2R outline_nuc x
#=GF SUBFAM_GUIDE65_R2R tick_label x 6 nt
#=GF SUBFAM_GUIDE65_R2R shade_along_backbone ILOOP:Y rgb:0,255,0
#=GF SUBFAM_GUIDE65_R2R shade_along_backbone ILOOP:S rgb:0,255,0
#=GF SUBFAM_GUIDE65_R2R shade_along_backbone ILOOP:U rgb:0,255,0
#=GF SUBFAM_GUIDE65_R2R outline_nuc Y
#=GF SUBFAM_GUIDE65_R2R outline_nuc y
#=GF SUBFAM_GUIDE65_R2R tick_label y 5 nt
#=GF SUBFAM_GUIDE65_R2R var_backbone_range_size_fake_nucs 1 V V

#=GF SUBFAM_GUIDE66_R2R no5
#=GF SUBFAM_GUIDE66_R2R set_dir pos0 -90
#=GF SUBFAM_GUIDE66_R2R shade_along_backbone ILOOP:P rgb:0,255,0
#=GF SUBFAM_GUIDE66_R2R shade_along_backbone ILOOP:X rgb:0,255,0
#=GF SUBFAM_GUIDE66_R2R shade_along_backbone ILOOP:Q rgb:0,255,0
#=GF SUBFAM_GUIDE66_R2R outline_nuc Q
#=GF SUBFAM_GUIDE66_R2R outline_nuc X
#=GF SUBFAM_GUIDE66_R2R outline_nuc x
#=GF SUBFAM_GUIDE66_R2R tick_label x 6 nt
#=GF SUBFAM_GUIDE66_R2R shade_along_backbone ILOOP:Y rgb:0,255,0
#=GF SUBFAM_GUIDE66_R2R shade_along_backbone ILOOP:S rgb:0,255,0
#=GF SUBFAM_GUIDE66_R2R shade_along_backbone ILOOP:U rgb:0,255,0
#=GF SUBFAM_GUIDE66_R2R outline_nuc Y
#=GF SUBFAM_GUIDE66_R2R outline_nuc y
#=GF SUBFAM_GUIDE66_R2R outline_nuc S
#=GF SUBFAM_GUIDE66_R2R tick_label y 5+1 nt
#=GF SUBFAM_GUIDE66_R2R var_backbone_range_size_fake_nucs 1 S S

#=GF SUBFAM_GUIDE67_R2R no5
#=GF SUBFAM_GUIDE67_R2R set_dir pos0 -90
#=GF SUBFAM_GUIDE67_R2R shade_along_backbone ILOOP:P rgb:0,255,0
#=GF SUBFAM_GUIDE67_R2R shade_along_backbone ILOOP:X rgb:0,255,0
#=GF SUBFAM_GUIDE67_R2R shade_along_backbone ILOOP:Q rgb:0,255,0
#=GF SUBFAM_GUIDE67_R2R outline_nuc Q
#=GF SUBFAM_GUIDE67_R2R outline_nuc X
#=GF SUBFAM_GUIDE67_R2R outline_nuc x
#=GF SUBFAM_GUIDE67_R2R tick_label x 6 nt
#=GF SUBFAM_GUIDE67_R2R shade_along_backbone ILOOP:Y rgb:0,255,0
#=GF SUBFAM_GUIDE67_R2R shade_along_backbone ILOOP:S rgb:0,255,0
#=GF SUBFAM_GUIDE67_R2R shade_along_backbone ILOOP:U rgb:0,255,0
#=GF SUBFAM_GUIDE67_R2R outline_nuc Y
#=GF SUBFAM_GUIDE67_R2R outline_nuc y
#=GF SUBFAM_GUIDE67_R2R outline_nuc S
#=GF SUBFAM_GUIDE67_R2R outline_nuc U
#=GF SUBFAM_GUIDE67_R2R tick_label y 5+2 nt
#=GF SUBFAM_GUIDE67_R2R var_backbone_range_size_fake_nucs 1 U U 2 nt
#=GF SUBFAM_GUIDE67_R2R var_backbone_range_size_fake_nucs 1 S S

#=GF SUBFAM_GUIDE75_R2R no5
#=GF SUBFAM_GUIDE75_R2R set_dir pos0 -90
#=GF SUBFAM_GUIDE75_R2R shade_along_backbone ILOOP:P rgb:0,255,0
#=GF SUBFAM_GUIDE75_R2R shade_along_backbone ILOOP:X rgb:0,255,0
#=GF SUBFAM_GUIDE75_R2R shade_along_backbone ILOOP:Q rgb:0,255,0
#=GF SUBFAM_GUIDE75_R2R outline_nuc Q
#=GF SUBFAM_GUIDE75_R2R outline_nuc X
#=GF SUBFAM_GUIDE75_R2R outline_nuc x
#=GF SUBFAM_GUIDE75_R2R outline_nuc P
#=GF SUBFAM_GUIDE75_R2R tick_label x 6+1 nt
#=GF SUBFAM_GUIDE75_R2R shade_along_backbone ILOOP:Y rgb:0,255,0
#=GF SUBFAM_GUIDE75_R2R shade_along_backbone ILOOP:S rgb:0,255,0
#=GF SUBFAM_GUIDE75_R2R shade_along_backbone ILOOP:U rgb:0,255,0
#=GF SUBFAM_GUIDE75_R2R outline_nuc Y
#=GF SUBFAM_GUIDE75_R2R outline_nuc y
#=GF SUBFAM_GUIDE75_R2R tick_label y 5 nt
#=GF SUBFAM_GUIDE75_R2R var_backbone_range_size_fake_nucs 1 W W
#=GF SUBFAM_GUIDE75_R2R var_backbone_range_size_fake_nucs 1 P P

#=GF SUBFAM_GUIDE85_R2R no5
#=GF SUBFAM_GUIDE85_R2R set_dir pos0 -90
#=GF SUBFAM_GUIDE85_R2R shade_along_backbone ILOOP:P rgb:0,255,0
#=GF SUBFAM_GUIDE85_R2R shade_along_backbone ILOOP:X rgb:0,255,0
#=GF SUBFAM_GUIDE85_R2R shade_along_backbone ILOOP:Q rgb:0,255,0
#=GF SUBFAM_GUIDE85_R2R outline_nuc Q
#=GF SUBFAM_GUIDE85_R2R outline_nuc X
#=GF SUBFAM_GUIDE85_R2R outline_nuc x
#=GF SUBFAM_GUIDE85_R2R outline_nuc P
#=GF SUBFAM_GUIDE85_R2R tick_label x 6+2 nt
#=GF SUBFAM_GUIDE85_R2R shade_along_backbone ILOOP:Y rgb:0,255,0
#=GF SUBFAM_GUIDE85_R2R shade_along_backbone ILOOP:S rgb:0,255,0
#=GF SUBFAM_GUIDE85_R2R shade_along_backbone ILOOP:U rgb:0,255,0
#=GF SUBFAM_GUIDE85_R2R outline_nuc Y
#=GF SUBFAM_GUIDE85_R2R outline_nuc y
#=GF SUBFAM_GUIDE85_R2R tick_label y 5 nt
#=GF SUBFAM_GUIDE85_R2R var_backbone_range_size_fake_nucs 1 P P 2 nt
#=GF SUBFAM_GUIDE85_R2R var_backbone_range_size_fake_nucs 1 1 1


#=GF R2R shade_along_backbone 1 rgb:0,255,0
```

```
#=GF R2R shade_along_backbone 6 rgb:0,255,0
#=GF R2R outline_nuc H
#=GF R2R outline_nuc h
#=GF R2R outline_nuc r
#=GF R2R outline_nuc i
#=GF R2R outline_nuc t
#=GF R2R tick_label h 9 bp
#=GF R2R tick_label i 10 bp


#=GF R2R_oneseq Pa21-S891 shade_along_backbone KLOOP:K rgb:0,129,255
#=GF R2R_oneseq Pa21-S891 tick_label KLOOPL:k K-loop
#=GF R2R_oneseq Pa21-S891 shade_along_backbone ILOOP:Y rgb:0,255,0
#=GF R2R_oneseq Pa21-S891 shade_along_backbone ILOOP:S rgb:0,255,0
#=GF R2R_oneseq Pa21-S891 shade_along_backbone ILOOP:U rgb:0,255,0
#=GF R2R_oneseq Pa21-S891 tick_label ILOOPL:y 3' guide sequence \n (5 stacked layers)
#=GF R2R_oneseq Pa21-S891 shade_along_backbone ILOOP:P rgb:0,255,0
#=GF R2R_oneseq Pa21-S891 shade_along_backbone ILOOP:Q rgb:0,255,0
#=GF R2R_oneseq Pa21-S891 shade_along_backbone ILOOP:X rgb:0,255,0
#=GF R2R_oneseq Pa21-S891 tick_label ILOOPL:x 5' guide sequence \n (6 stacked layers +1nt)
#=GF R2R_oneseq Pa21-S891 shade_along_backbone ILOOP:Z rgb:200,200,200
#=GF R2R_oneseq Pa21-S891 tick_label ILOOPL:z ANA box

#=GF R2R_oneseq Pa91-L2685 shade_along_backbone KLOOP:K rgb:0,129,255
#=GF R2R_oneseq Pa91-L2685 tick_label KLOOPL:k K-loop
#=GF R2R_oneseq Pa91-L2685 shade_along_backbone ILOOP:Y rgb:0,255,0
#=GF R2R_oneseq Pa91-L2685 shade_along_backbone ILOOP:S rgb:0,255,0
#=GF R2R_oneseq Pa91-L2685 shade_along_backbone ILOOP:U rgb:0,255,0
#=GF R2R_oneseq Pa91-L2685 tick_label ILOOPL:y 3' guide sequence \n (5 stacked layers)
#=GF R2R_oneseq Pa91-L2685 shade_along_backbone ILOOP:P rgb:0,255,0
#=GF R2R_oneseq Pa91-L2685 shade_along_backbone ILOOP:Q rgb:0,255,0
#=GF R2R_oneseq Pa91-L2685 shade_along_backbone ILOOP:X rgb:0,255,0
#=GF R2R_oneseq Pa91-L2685 tick_label ILOOPL:x 5' guide sequence \n (6 stacked layers)
#=GF R2R_oneseq Pa91-L2685 shade_along_backbone ILOOP:Z rgb:200,200,200
#=GF R2R_oneseq Pa91-L2685 tick_label ILOOPL:z ANA box

#=GF R2R_oneseq Pa105.2-L2794 shade_along_backbone KTURN:T rgb:0,129,255
#=GF R2R_oneseq Pa105.2-L2794 shade_along_backbone KTURN:R rgb:0,129,255
#=GF R2R_oneseq Pa105.2-L2794 tick_label KTURNL:r K-turn
#=GF R2R_oneseq Pa105.2-L2794 shade_along_backbone ILOOP:Y rgb:0,255,0
#=GF R2R_oneseq Pa105.2-L2794 shade_along_backbone ILOOP:S rgb:0,255,0
#=GF R2R_oneseq Pa105.2-L2794 shade_along_backbone ILOOP:U rgb:0,255,0
#=GF R2R_oneseq Pa105.2-L2794 tick_label ILOOPL:y 3' guide sequence \n (5 stacked layers +1nt)
#=GF R2R_oneseq Pa105.2-L2794 shade_along_backbone ILOOP:P rgb:0,255,0
#=GF R2R_oneseq Pa105.2-L2794 shade_along_backbone ILOOP:Q rgb:0,255,0
#=GF R2R_oneseq Pa105.2-L2794 shade_along_backbone ILOOP:X rgb:0,255,0
#=GF R2R_oneseq Pa105.2-L2794 tick_label ILOOPL:x 5' guide sequence \n (6 stacked layers)
#=GF R2R_oneseq Pa105.2-L2794 shade_along_backbone ILOOP:Z rgb:200,200,200
#=GF R2R_oneseq Pa105.2-L2794 tick_label ILOOPL:z ANA box
#=GF R2R_oneseq Pa105.2-L2794 var_backbone_range_size_fake_nucs 1 8 8

#=GF R2R_oneseq Pa105.2-L1377 shade_along_backbone KTURN:T rgb:0,129,255
#=GF R2R_oneseq Pa105.2-L1377 shade_along_backbone KTURN:R rgb:0,129,255
#=GF R2R_oneseq Pa105.2-L1377 tick_label KTURNL:r K-turn
#=GF R2R_oneseq Pa105.2-L1377 shade_along_backbone ILOOP:Y rgb:0,255,0
#=GF R2R_oneseq Pa105.2-L1377 shade_along_backbone ILOOP:S rgb:0,255,0
#=GF R2R_oneseq Pa105.2-L1377 shade_along_backbone ILOOP:U rgb:0,255,0
#=GF R2R_oneseq Pa105.2-L1377 tick_label ILOOPL:y 3' guide sequence \n (5 stacked layers)
#=GF R2R_oneseq Pa105.2-L1377 shade_along_backbone ILOOP:P rgb:0,255,0
#=GF R2R_oneseq Pa105.2-L1377 shade_along_backbone ILOOP:Q rgb:0,255,0
#=GF R2R_oneseq Pa105.2-L1377 shade_along_backbone ILOOP:X rgb:0,255,0
#=GF R2R_oneseq Pa105.2-L1377 tick_label ILOOPL:x 5' guide sequence \n (6 stacked layers)
#=GF R2R_oneseq Pa105.2-L1377 shade_along_backbone ILOOP:Z rgb:200,200,200
#=GF R2R_oneseq Pa105.2-L1377 tick_label ILOOPL:z ANA box

#=GF R2R_oneseq Pa105.2-S27 shade_along_backbone KTURN:T rgb:0,129,255
#=GF R2R_oneseq Pa105.2-S27 shade_along_backbone KTURN:R rgb:0,129,255
#=GF R2R_oneseq Pa105.2-S27 tick_label KTURNL:r K-turn
#=GF R2R_oneseq Pa105.2-S27 shade_along_backbone ILOOP:Y rgb:0,255,0
#=GF R2R_oneseq Pa105.2-S27 shade_along_backbone ILOOP:S rgb:0,255,0
#=GF R2R_oneseq Pa105.2-S27 shade_along_backbone ILOOP:U rgb:0,255,0
#=GF R2R_oneseq Pa105.2-S27 tick_label ILOOPL:y 3' guide sequence \n (5 stacked layers)
#=GF R2R_oneseq Pa105.2-S27 shade_along_backbone ILOOP:P rgb:0,255,0
#=GF R2R_oneseq Pa105.2-S27 shade_along_backbone ILOOP:Q rgb:0,255,0
#=GF R2R_oneseq Pa105.2-S27 shade_along_backbone ILOOP:X rgb:0,255,0
#=GF R2R_oneseq Pa105.2-S27 tick_label ILOOPL:x 5' guide sequence \n (6 stacked layers)
#=GF R2R_oneseq Pa105.2-S27 shade_along_backbone ILOOP:Z rgb:200,200,200
#=GF R2R_oneseq Pa105.2-S27 tick_label ILOOPL:z ANA box

#=GF R2R_oneseq Pa35.1-L2930 shade_along_backbone KTURN:T rgb:0,129,255
#=GF R2R_oneseq Pa35.1-L2930 shade_along_backbone KTURN:R rgb:0,129,255
#=GF R2R_oneseq Pa35.1-L2930 tick_label KTURNL:r K-turn
#=GF R2R_oneseq Pa35.1-L2930 shade_along_backbone ILOOP:Y rgb:0,255,0
#=GF R2R_oneseq Pa35.1-L2930 shade_along_backbone ILOOP:S rgb:0,255,0
#=GF R2R_oneseq Pa35.1-L2930 shade_along_backbone ILOOP:U rgb:0,255,0
#=GF R2R_oneseq Pa35.1-L2930 tick_label ILOOPL:y 3' guide sequence \n (5 stacked layers)
#=GF R2R_oneseq Pa35.1-L2930 shade_along_backbone ILOOP:P rgb:0,255,0
#=GF R2R_oneseq Pa35.1-L2930 shade_along_backbone ILOOP:Q rgb:0,255,0
#=GF R2R_oneseq Pa35.1-L2930 shade_along_backbone ILOOP:X rgb:0,255,0
#=GF R2R_oneseq Pa35.1-L2930 tick_label ILOOPL:x 5' guide sequence \n (6 stacked layers)
#=GF R2R_oneseq Pa35.1-L2930 shade_along_backbone ILOOP:Z rgb:200,200,200
#=GF R2R_oneseq Pa35.1-L2930 tick_label ILOOPL:z ANA box

#=GF R2R_oneseq Pa35.1-L2672 shade_along_backbone KTURN:T rgb:0,129,255
#=GF R2R_oneseq Pa35.1-L2672 shade_along_backbone KTURN:R rgb:0,129,255
```

```
#=GF R2R_oneseq Pa35.1-L2672 tick_label KTURNL:r K-turn
#=GF R2R_oneseq Pa35.1-L2672 shade_along_backbone ILOOP:Y rgb:0,255,0
#=GF R2R_oneseq Pa35.1-L2672 shade_along_backbone ILOOP:S rgb:0,255,0
#=GF R2R_oneseq Pa35.1-L2672 shade_along_backbone ILOOP:U rgb:0,255,0
#=GF R2R_oneseq Pa35.1-L2672 tick_label ILOOPL:y 3’ guide sequence \n (5 stacked layers)
#=GF R2R_oneseq Pa35.1-L2672 shade_along_backbone ILOOP:P rgb:0,255,0
#=GF R2R_oneseq Pa35.1-L2672 shade_along_backbone ILOOP:Q rgb:0,255,0
#=GF R2R_oneseq Pa35.1-L2672 shade_along_backbone ILOOP:X rgb:0,255,0
#=GF R2R_oneseq Pa35.1-L2672 tick_label ILOOPL:x 5’ guide sequence \n (6 stacked layers)
#=GF R2R_oneseq Pa35.1-L2672 shade_along_backbone ILOOP:Z rgb:200,200,200
#=GF R2R_oneseq Pa35.1-L2672 tick_label ILOOPL:z ANA box

#=GF R2R_oneseq Pa35.2-L2549a shade_along_backbone KLOOP:K rgb:0,129,255
#=GF R2R_oneseq Pa35.2-L2549a tick_label KLOOPL:k K-loop
#=GF R2R_oneseq Pa35.2-L2549a shade_along_backbone ILOOP:Y rgb:0,255,0
#=GF R2R_oneseq Pa35.2-L2549a shade_along_backbone ILOOP:S rgb:0,255,0
#=GF R2R_oneseq Pa35.2-L2549a shade_along_backbone ILOOP:U rgb:0,255,0
#=GF R2R_oneseq Pa35.2-L2549a tick_label ILOOPL:y 3’ guide sequence \n (5 stacked layers +1nt)
#=GF R2R_oneseq Pa35.2-L2549a shade_along_backbone ILOOP:P rgb:0,255,0
#=GF R2R_oneseq Pa35.2-L2549a shade_along_backbone ILOOP:Q rgb:0,255,0
#=GF R2R_oneseq Pa35.2-L2549a shade_along_backbone ILOOP:X rgb:0,255,0
#=GF R2R_oneseq Pa35.2-L2549a tick_label ILOOPL:x 5’ guide sequence \n (3 stacked layers +2nt)
#=GF R2R_oneseq Pa35.2-L2549a shade_along_backbone ILOOP:Z rgb:200,200,200
#=GF R2R_oneseq Pa35.2-L2549a tick_label ILOOPL:z ANA box

#=GF R2R_oneseq Pa35.2-L2697 shade_along_backbone KLOOP:K rgb:0,129,255
#=GF R2R_oneseq Pa35.2-L2697 tick_label KLOOPL:k K-loop
#=GF R2R_oneseq Pa35.2-L2697 shade_along_backbone ILOOP:Y rgb:0,255,0
#=GF R2R_oneseq Pa35.2-L2697 shade_along_backbone ILOOP:S rgb:0,255,0
#=GF R2R_oneseq Pa35.2-L2697 shade_along_backbone ILOOP:U rgb:0,255,0
#=GF R2R_oneseq Pa35.2-L2697 tick_label ILOOPL:y 3’ guide sequence \n (5 stacked layers)
#=GF R2R_oneseq Pa35.2-L2697 shade_along_backbone ILOOP:P rgb:0,255,0
#=GF R2R_oneseq Pa35.2-L2697 shade_along_backbone ILOOP:Q rgb:0,255,0
#=GF R2R_oneseq Pa35.2-L2697 shade_along_backbone ILOOP:X rgb:0,255,0
#=GF R2R_oneseq Pa35.2-L2697 tick_label ILOOPL:x 5’ guide sequence \n (3 stacked layers +2nt)
#=GF R2R_oneseq Pa35.2-L2697 shade_along_backbone ILOOP:Z rgb:200,200,200
#=GF R2R_oneseq Pa35.2-L2697 tick_label ILOOPL:z ANA box

#=GF R2R_oneseq Pa105.1-L2554a shade_along_backbone KTURN:T rgb:0,129,255
#=GF R2R_oneseq Pa105.1-L2554a shade_along_backbone KTURN:R rgb:0,129,255
#=GF R2R_oneseq Pa105.1-L2554a tick_label KTURNL:r K-turn
#=GF R2R_oneseq Pa105.1-L2554a shade_along_backbone ILOOP:Y rgb:0,255,0
#=GF R2R_oneseq Pa105.1-L2554a shade_along_backbone ILOOP:S rgb:0,255,0
#=GF R2R_oneseq Pa105.1-L2554a shade_along_backbone ILOOP:U rgb:0,255,0
#=GF R2R_oneseq Pa105.1-L2554a tick_label ILOOPL:y 3’ guide sequence \n (5 stacked layers +1nt)
#=GF R2R_oneseq Pa105.1-L2554a shade_along_backbone ILOOP:P rgb:0,255,0
#=GF R2R_oneseq Pa105.1-L2554a shade_along_backbone ILOOP:Q rgb:0,255,0
#=GF R2R_oneseq Pa105.1-L2554a shade_along_backbone ILOOP:X rgb:0,255,0
#=GF R2R_oneseq Pa105.1-L2554a tick_label ILOOPL:x 5’ guide sequence \n (3 stacked layers +2nt)
#=GF R2R_oneseq Pa105.1-L2554a shade_along_backbone ILOOP:Z rgb:200,200,200
#=GF R2R_oneseq Pa105.1-L2554a tick_label ILOOPL:z ANA box

#=GF R2R_oneseq Pa160-S922 shade_along_backbone KTURN:T rgb:0,129,255
#=GF R2R_oneseq Pa160-S922 shade_along_backbone KTURN:R rgb:0,129,255
#=GF R2R_oneseq Pa160-S922 tick_label KTURNL:r K-turn
#=GF R2R_oneseq Pa160-S922 shade_along_backbone ILOOP:Y rgb:0,255,0
#=GF R2R_oneseq Pa160-S922 shade_along_backbone ILOOP:S rgb:0,255,0
#=GF R2R_oneseq Pa160-S922 shade_along_backbone ILOOP:U rgb:0,255,0
#=GF R2R_oneseq Pa160-S922 tick_label ILOOPL:y 3’ guide sequence \n (5 stacked layers)
#=GF R2R_oneseq Pa160-S922 shade_along_backbone ILOOP:P rgb:0,255,0
#=GF R2R_oneseq Pa160-S922 shade_along_backbone ILOOP:Q rgb:0,255,0
#=GF R2R_oneseq Pa160-S922 shade_along_backbone ILOOP:X rgb:0,255,0
#=GF R2R_oneseq Pa160-S922 tick_label ILOOPL:x 5’ guide sequence \n (3 stacked layers +2nt)
#=GF R2R_oneseq Pa160-S922 shade_along_backbone ILOOP:Z rgb:200,200,200
#=GF R2R_oneseq Pa160-S922 tick_label ILOOPL:z ANA box

#=GF R2R_oneseq Pa40.3-L2016 shade_along_backbone KTURN:T rgb:0,129,255
#=GF R2R_oneseq Pa40.3-L2016 shade_along_backbone KTURN:R rgb:0,129,255
#=GF R2R_oneseq Pa40.3-L2016 tick_label KTURNL:r K-turn
#=GF R2R_oneseq Pa40.3-L2016 shade_along_backbone ILOOP:Y rgb:0,255,0
#=GF R2R_oneseq Pa40.3-L2016 shade_along_backbone ILOOP:S rgb:0,255,0
#=GF R2R_oneseq Pa40.3-L2016 shade_along_backbone ILOOP:U rgb:0,255,0
#=GF R2R_oneseq Pa40.3-L2016 tick_label ILOOPL:y 3’ guide sequence \n (5 stacked layers)
#=GF R2R_oneseq Pa40.3-L2016 shade_along_backbone ILOOP:P rgb:0,255,0
#=GF R2R_oneseq Pa40.3-L2016 shade_along_backbone ILOOP:Q rgb:0,255,0
#=GF R2R_oneseq Pa40.3-L2016 shade_along_backbone ILOOP:X rgb:0,255,0
#=GF R2R_oneseq Pa40.3-L2016 tick_label ILOOPL:x 5’ guide sequence \n (6 stacked layers)
#=GF R2R_oneseq Pa40.3-L2016 shade_along_backbone ILOOP:Z rgb:200,200,200
#=GF R2R_oneseq Pa40.3-L2016 tick_label ILOOPL:z ANA box

#=GF R2R_oneseq Pa40.1-L2588 shade_along_backbone KLOOP:K rgb:0,129,255
#=GF R2R_oneseq Pa40.1-L2588 tick_label KLOOPL:k K-loop
#=GF R2R_oneseq Pa40.1-L2588 shade_along_backbone ILOOP:Y rgb:0,255,0
#=GF R2R_oneseq Pa40.1-L2588 shade_along_backbone ILOOP:S rgb:0,255,0
#=GF R2R_oneseq Pa40.1-L2588 shade_along_backbone ILOOP:U rgb:0,255,0
#=GF R2R_oneseq Pa40.1-L2588 tick_label ILOOPL:y 3’ guide sequence \n (5 stacked layers)
#=GF R2R_oneseq Pa40.1-L2588 shade_along_backbone ILOOP:P rgb:0,255,0
#=GF R2R_oneseq Pa40.1-L2588 shade_along_backbone ILOOP:Q rgb:0,255,0
#=GF R2R_oneseq Pa40.1-L2588 shade_along_backbone ILOOP:X rgb:0,255,0
#=GF R2R_oneseq Pa40.1-L2588 tick_label ILOOPL:x 5’ guide sequence \n (6 stacked layers +2nt)
#=GF R2R_oneseq Pa40.1-L2588 shade_along_backbone ILOOP:Z rgb:200,200,200
#=GF R2R_oneseq Pa40.1-L2588 tick_label ILOOPL:z ANA box

//
```

Listing 18: Stockholm Alignment used in R2R representations of Pa19-S1017a.

```
# STOCKHOLM 1.0

Pa19-S1017a                  GCCCAGGGG.UCAAGACGGCGGCGUCGGGGG.GA....UUGGGGGC....AAA....GCCCCCGG..CAUGA.ACCC.CG..CCCUCCU..C.CCCUGGGU..AUA.CGGACGCGCCGAGAGGAGGUGCAUGGCCGCCGUC
Ph19-S1017a                  GCCCAGGGG.UCAAGACGGCGGCGUCGGGGG.GA....UUGGGGGC....AAA....GCCCCCGG..AAUGA.ACCC.CG..CCCUCCU..C.CCCUGGGU..AUA.CGGACGCGCCGAGAGGAGGUGCAUGGCCGCCGUC
Pf19-S1017a                  ACCCAGGGG.CCC.GACGGCGGCGUCGGGGG.GA....UUGGGGGC....AAA....GCCCCCGG..AAUGA.ACCC.CG..CCCUCCU..C.CCCUGGGU..AUA.CGGACGCGCCGAGAGGAGGUGCAUGGCCGCCGUC
#=GC SS_cons                 <<<<<<<<<..............<-<<<<<<.<<....<<<<<<<<___________>>>>>>>>.....>>.>>>>.>>..>........>.>>>>>>>>........................................
#=GC SS_cons_1               ................<<<<<<<............................................................<<<<<<...............................>>>>>>..>.>>>>>>.....
#=GC R2R_LABEL               q.......s.6....1lLLLLLL.3...........4...............................5KHH.HHiH8HHB..LLLLLLj.t.HHHhHHHr....a.0CCCC2C......GGGGGGmonGGGGGGg.....
#=GC SUBFAM_hybrid_R2R_LABEL ----------------AAaAAAAb----------------------------------------------------------chHHHHH-------------------------------DDDDDDmonEEEEEEE-----
#=GC R2R_XLABEL_KTURN        ................................TTTTTTTT.......................RRRRRRRRR.....................................................................
#=GC R2R_XLABEL_KTURNL       ....................................................................r........................................................................
#=GC R2R_XLABEL_ILOOP        ..........PPPPPPPPPQXXX............................................................YYYYRSU..V...........................YYYYYY..PPPPPPPPPPPPP
#=GC R2R_XLABEL_ILOOPL       ...................x.................................................................y..su..v................................................
#=GC R2R_XLABEL_ANA          .......................................................................................................CCC...................................

#=GF R2R ignore_ss_except_for_pairs _1 outline_only_bp
#=GF R2R place_explicit 2 2-- -45 1 0 0 0 -90
#=GF R2R place_explicit n n-- -45 1 0 0 0 -90
#=GF R2R place_explicit m m-- -45 1 0 0 0 -90
#=GF R2R tick_label a ANA box
#=GF R2R delcols 0
#=GF R2R keep q r
#=GF R2R keep s t
#=GF R2R outline_nuc L
#=GF R2R outline_nuc l
#=GF R2R inline_nuc G
#=GF R2R inline_nuc g
#=GF R2R inline_nuc n
#=GF R2R circle_nuc 2 rgb:0,0,0
#=GF R2R circle_nuc C rgb:0,0,0
#=GF R2R nuc_color 2 rgb:0,0,0
#=GF R2R nuc_color C rgb:0,0,0
#=GF R2R var_backbone_range_size_fake_nucs 1 3 3
#=GF R2R var_backbone_range_size_fake_nucs 1 6 1
#=GF R2R shade_along_backbone KTURN:T rgb:0,129,255
#=GF R2R shade_along_backbone KTURN:R rgb:0,129,255
#=GF R2R shade_along_backbone ANA:C rgb:200,200,200
#=GF R2R outline_along_backbone ILOOP:Y rgb:255,228,196
#=GF R2R outline_along_backbone ILOOP:P rgb:193,255,193
#=GF R2R outline_along_backbone ILOOP:Q rgb:193,255,193
#=GF R2R outline_along_backbone ILOOP:X rgb:193,255,193
#=GF R2R shade_along_backbone ILOOP:S rgb:255,228,196
#=GF R2R shade_along_backbone ILOOP:R rgb:255,228,196
#=GF R2R shade_along_backbone ILOOP:Y rgb:255,228,196
#=GF R2R shade_along_backbone ILOOP:P rgb:193,255,193
#=GF R2R shade_along_backbone ILOOP:Q rgb:193,255,193
#=GF R2R shade_along_backbone ILOOP:X rgb:193,255,193

#=GF SUBFAM_PERL_PRED hybrid return 1;
#=GF SUBFAM_hybrid_R2R subst_ss _1 primary outline_only_bp
#=GF SUBFAM_hybrid_R2R set_dir pos0 0
#=GF SUBFAM_hybrid_R2R place_explicit b b-- -45 2 0 0 0 -90
#=GF SUBFAM_hybrid_R2R place_explicit c c-- 90 1.5 0 0 0 90
#=GF SUBFAM_hybrid_R2R place_explicit h h-- +45 2 0 0 0 0
#=GF SUBFAM_hybrid_R2R place_explicit m m-- -45 2 0 0 0 -90
#=GF SUBFAM_hybrid_R2R place_explicit o o-- 90 1.5 0 0 0 90
#=GF SUBFAM_hybrid_R2R tick_label o target
#=GF SUBFAM_hybrid_R2R tick_label a guide
#=GF SUBFAM_hybrid_R2R shade_along_backbone A rgb:193,255,193
#=GF SUBFAM_hybrid_R2R shade_along_backbone a rgb:193,255,193
#=GF SUBFAM_hybrid_R2R shade_along_backbone E rgb:193,255,193
#=GF SUBFAM_hybrid_R2R shade_along_backbone n rgb:193,255,193
#=GF SUBFAM_hybrid_R2R shade_along_backbone D rgb:255,228,196
#=GF SUBFAM_hybrid_R2R shade_along_backbone d rgb:255,228,196
#=GF SUBFAM_hybrid_R2R shade_along_backbone h rgb:255,228,196
#=GF SUBFAM_hybrid_R2R shade_along_backbone H rgb:255,228,196

#=GF R2R_oneseq Pa19-S1017a shade_along_backbone KTURN:T rgb:0,129,255
#=GF R2R_oneseq Pa19-S1017a shade_along_backbone KTURN:R rgb:0,129,255
#=GF R2R_oneseq Pa19-S1017a tick_label KTURNL:r K-turn
#=GF R2R_oneseq Pa19-S1017a shade_along_backbone ILOOP:Y rgb:255,228,196
#=GF R2R_oneseq Pa19-S1017a shade_along_backbone ILOOP:S rgb:255,228,196
#=GF R2R_oneseq Pa19-S1017a shade_along_backbone ILOOP:U rgb:255,228,196
#=GF R2R_oneseq Pa19-S1017a shade_along_backbone ILOOP:P rgb:193,255,193
#=GF R2R_oneseq Pa19-S1017a shade_along_backbone ILOOP:Q rgb:193,255,193
#=GF R2R_oneseq Pa19-S1017a shade_along_backbone ILOOP:X rgb:193,255,193
#=GF R2R_oneseq Pa19-S1017a shade_along_backbone ANA:C rgb:200,200,200

#=GF R2R_oneseq Ph19-S1017a shade_along_backbone KTURN:T rgb:0,129,255
#=GF R2R_oneseq Ph19-S1017a shade_along_backbone KTURN:R rgb:0,129,255
#=GF R2R_oneseq Ph19-S1017a tick_label KTURNL:r K-turn
#=GF R2R_oneseq Ph19-S1017a shade_along_backbone ILOOP:Y rgb:255,228,196
#=GF R2R_oneseq Ph19-S1017a shade_along_backbone ILOOP:S rgb:255,228,196
#=GF R2R_oneseq Ph19-S1017a shade_along_backbone ILOOP:U rgb:255,228,196
#=GF R2R_oneseq Ph19-S1017a shade_along_backbone ILOOP:P rgb:193,255,193
#=GF R2R_oneseq Ph19-S1017a shade_along_backbone ILOOP:Q rgb:193,255,193
#=GF R2R_oneseq Ph19-S1017a shade_along_backbone ILOOP:X rgb:193,255,193
#=GF R2R_oneseq Ph19-S1017a shade_along_backbone ANA:C rgb:200,200,200

#=GF R2R_oneseq Pf19-S1017a shade_along_backbone KTURN:T rgb:0,129,255
#=GF R2R_oneseq Pf19-S1017a shade_along_backbone KTURN:R rgb:0,129,255
#=GF R2R_oneseq Pf19-S1017a tick_label KTURNL:r K-turn
#=GF R2R_oneseq Pf19-S1017a shade_along_backbone ILOOP:Y rgb:255,228,196
#=GF R2R_oneseq Pf19-S1017a shade_along_backbone ILOOP:S rgb:255,228,196
#=GF R2R_oneseq Pf19-S1017a shade_along_backbone ILOOP:U rgb:255,228,196
#=GF R2R_oneseq Pf19-S1017a shade_along_backbone ILOOP:P rgb:193,255,193
#=GF R2R_oneseq Pf19-S1017a shade_along_backbone ILOOP:Q rgb:193,255,193
#=GF R2R_oneseq Pf19-S1017a shade_along_backbone ILOOP:X rgb:193,255,193
#=GF R2R_oneseq Pf19-S1017a shade_along_backbone ANA:C rgb:200,200,200

//
```

Listing 19: Stockholm Aligmnent used in R2R representations of Pa19-S1017b.

```
# STOCKHOLM 1.0

Pa19-S1017b                  GCCCAGGGG.UCAAGACGGCGGCGUCGGGGG.GA....UUGGGGGC....AAA....GCCCCCGG..CAUGA.ACCC.CG..CCCUCCU..C.CCCUGGGU..AUA.CGGACGCGCCGAGAGGAGGUGCAUGGCCGCCGUC
Ph19-S1017b                  GCCCAGGGG.UCAAGACGGCGGCGUCGGGGG.GA....UUGGGGGC....AAA....GCCCCCGG..AAUGA.ACCC.CG..CCCUCCU..C.CCCUGGGU..AUA.CGGACGCGCCGAGAGGAGGUGCAUGGCCGCCGUC
Pf19-S1017b                  ACCCAGGGG.CCC.GACGGCGGCGUCGGGGG.GA....UUGGGGGC....AAA....GCCCCCGG..AAUGA.ACCC.CG..CCCUCCU..C.CCCUGGGU..AUA.CGGACGCGCCGAGAGGAGGUGCAUGGCCGCCGUC
#=GC SS_cons                 <<<<<<<<<................<<<<<<.<<....<<<<<<<<___________>>>>>>>>.....>>.>>>>.>>...........>.>>>>>>>>........................................
#=GC SS_cons_1               ..............<<<<<<<<<<<..........................................................<<<<<<...............................>>>>>>...>>>.>>>>>>>>
#=GC R2R_LABEL               q.......s.6..1lLLLLLLLLLL...........4...............................5KHH.HHiH8HHB..LLLLLLj.t.HHHhHHHr....a.0CCCC2C......GGGGGGmo.nGGGGGGGGGGg
#=GC SUBFAM_hybrid_R2R_LABEL --------------AAAAaAAAAAAb--------------------------------------------------------chHHHHH-------------------------------DDDDDDmo.nEEEEEEEEEEE
#=GC R2R_XLABEL_KTURN        ................................TTTTTTTT.......................RRRRRRRRR.....................................................................
#=GC R2R_XLABEL_KTURNL       ....................................................................r........................................................................
#=GC R2R_XLABEL_ILOOP        ..........PPPPPPPPPQXXXXX..........................................................YYYYRSU..V...........................YYYYYY...PPPPPPPPPPPP
#=GC R2R_XLABEL_ILOOPL       ...................x.................................................................y..su..v................................................
#=GC R2R_XLABEL_ANA          .......................................................................................................CCC...................................

#=GF R2R ignore_ss_except_for_pairs _1 outline_only_bp
#=GF R2R place_explicit 2 2-- -45 1 0 0 0 -90
#=GF R2R place_explicit n n-- -45 1 0 0 0 -90
#=GF R2R place_explicit m m-- -45 1 0 0 0 -90
#=GF R2R tick_label a ANA box
#=GF R2R delcols 0
#=GF R2R keep q r
#=GF R2R keep s t
#=GF R2R outline_nuc L
#=GF R2R outline_nuc l
#=GF R2R inline_nuc G
#=GF R2R inline_nuc g
#=GF R2R inline_nuc n
#=GF R2R circle_nuc 2 rgb:0,0,0
#=GF R2R circle_nuc C rgb:0,0,0
#=GF R2R nuc_color 2 rgb:0,0,0
#=GF R2R nuc_color C rgb:0,0,0
#=GF R2R var_backbone_range_size_fake_nucs 3 6 1
#=GF R2R shade_along_backbone KTURN:T rgb:0,129,255
#=GF R2R shade_along_backbone KTURN:R rgb:0,129,255
#=GF R2R shade_along_backbone ANA:C rgb:200,200,200
#=GF R2R outline_along_backbone ILOOP:Y rgb:255,228,196
#=GF R2R outline_along_backbone ILOOP:P rgb:193,255,193
#=GF R2R outline_along_backbone ILOOP:Q rgb:193,255,193
#=GF R2R outline_along_backbone ILOOP:X rgb:193,255,193
#=GF R2R shade_along_backbone ILOOP:S rgb:255,228,196
#=GF R2R shade_along_backbone ILOOP:R rgb:255,228,196
#=GF R2R shade_along_backbone ILOOP:Y rgb:255,228,196
#=GF R2R shade_along_backbone ILOOP:P rgb:193,255,193
#=GF R2R shade_along_backbone ILOOP:Q rgb:193,255,193
#=GF R2R shade_along_backbone ILOOP:X rgb:193,255,193

#=GF SUBFAM_PERL_PRED hybrid return 1;
#=GF SUBFAM_hybrid_R2R subst_ss _1 primary outline_only_bp
#=GF SUBFAM_hybrid_R2R set_dir pos0 0
#=GF SUBFAM_hybrid_R2R place_explicit b b-- -45 2 0 0 0 -90
#=GF SUBFAM_hybrid_R2R place_explicit c c-- 90 1.5 0 0 0 90
#=GF SUBFAM_hybrid_R2R place_explicit h h-- +45 2 0 0 0 0
#=GF SUBFAM_hybrid_R2R place_explicit m m-- -45 2 0 0 0 -90
#=GF SUBFAM_hybrid_R2R place_explicit o o-- 90 1.5 0 0 0 90
#=GF SUBFAM_hybrid_R2R tick_label o target
#=GF SUBFAM_hybrid_R2R tick_label a guide
#=GF SUBFAM_hybrid_R2R shade_along_backbone A rgb:193,255,193
#=GF SUBFAM_hybrid_R2R shade_along_backbone a rgb:193,255,193
#=GF SUBFAM_hybrid_R2R shade_along_backbone E rgb:193,255,193
#=GF SUBFAM_hybrid_R2R shade_along_backbone n rgb:193,255,193
#=GF SUBFAM_hybrid_R2R shade_along_backbone D rgb:255,228,196
#=GF SUBFAM_hybrid_R2R shade_along_backbone d rgb:255,228,196
#=GF SUBFAM_hybrid_R2R shade_along_backbone h rgb:255,228,196
#=GF SUBFAM_hybrid_R2R shade_along_backbone H rgb:255,228,196

#=GF R2R_oneseq Pa19-S1017b shade_along_backbone KTURN:T rgb:0,129,255
#=GF R2R_oneseq Pa19-S1017b shade_along_backbone KTURN:R rgb:0,129,255
#=GF R2R_oneseq Pa19-S1017b tick_label KTURNL:r K-turn
#=GF R2R_oneseq Pa19-S1017b shade_along_backbone ILOOP:Y rgb:255,228,196
#=GF R2R_oneseq Pa19-S1017b shade_along_backbone ILOOP:S rgb:255,228,196
#=GF R2R_oneseq Pa19-S1017b shade_along_backbone ILOOP:U rgb:255,228,196
#=GF R2R_oneseq Pa19-S1017b shade_along_backbone ILOOP:P rgb:193,255,193
#=GF R2R_oneseq Pa19-S1017b shade_along_backbone ILOOP:Q rgb:193,255,193
#=GF R2R_oneseq Pa19-S1017b shade_along_backbone ILOOP:X rgb:193,255,193
#=GF R2R_oneseq Pa19-S1017b shade_along_backbone ANA:C rgb:200,200,200

#=GF R2R_oneseq Ph19-S1017b shade_along_backbone KTURN:T rgb:0,129,255
#=GF R2R_oneseq Ph19-S1017b shade_along_backbone KTURN:R rgb:0,129,255
#=GF R2R_oneseq Ph19-S1017b tick_label KTURNL:r K-turn
#=GF R2R_oneseq Ph19-S1017b shade_along_backbone ILOOP:Y rgb:255,228,196
#=GF R2R_oneseq Ph19-S1017b shade_along_backbone ILOOP:S rgb:255,228,196
#=GF R2R_oneseq Ph19-S1017b shade_along_backbone ILOOP:U rgb:255,228,196
#=GF R2R_oneseq Ph19-S1017b shade_along_backbone ILOOP:P rgb:193,255,193
#=GF R2R_oneseq Ph19-S1017b shade_along_backbone ILOOP:Q rgb:193,255,193
#=GF R2R_oneseq Ph19-S1017b shade_along_backbone ILOOP:X rgb:193,255,193
#=GF R2R_oneseq Ph19-S1017b shade_along_backbone ANA:C rgb:200,200,200

#=GF R2R_oneseq Pf19-S1017b shade_along_backbone KTURN:T rgb:0,129,255
#=GF R2R_oneseq Pf19-S1017b shade_along_backbone KTURN:R rgb:0,129,255
#=GF R2R_oneseq Pf19-S1017b tick_label KTURNL:r K-turn
#=GF R2R_oneseq Pf19-S1017b shade_along_backbone ILOOP:Y rgb:255,228,196
#=GF R2R_oneseq Pf19-S1017b shade_along_backbone ILOOP:S rgb:255,228,196
#=GF R2R_oneseq Pf19-S1017b shade_along_backbone ILOOP:U rgb:255,228,196
#=GF R2R_oneseq Pf19-S1017b shade_along_backbone ILOOP:P rgb:193,255,193
#=GF R2R_oneseq Pf19-S1017b shade_along_backbone ILOOP:Q rgb:193,255,193
#=GF R2R_oneseq Pf19-S1017b shade_along_backbone ILOOP:X rgb:193,255,193
#=GF R2R_oneseq Pf19-S1017b shade_along_backbone ANA:C rgb:200,200,200

//
```

Table S1. Functional and structural features from known H/ACA guide RNAs in *Pyrococcus* targeting alternative positions in rRNAs and tRNAs.

guide:target pairs	*conservation*[a]	*expression*[b]	*promoter*[b]	*% GC content*	*pseudouridylation*[c]	*structure-function model*[d]	*hybrid energy*[e]	*duplex energy*[f]	*5' duplex energy*[f]
Pa19-S482	+	+	-	72	-	8/7/9-	-38	-13	-4.0
Pa19-L1563	+	+	-	72	-	8/7/9-	-42	-23	-15
Pa19-L1676	+	+	-	72	-	9/6/9-	-43	-21	-12
Pa19-L1676[1]	+	+	-	72	-	10/6/9+	-38	-21	-12
Pa19-L1676[1]	+	+	-	72	-	10/5/9+	-39	-26	-14
Pa19-L655[2]	+	+	-	72	-	10/5/9+	-39	-24	-10
Pa19-tR48(TAC)	+	+	-	72	-	9/6/9-	-41	-11	-12
Pa19-tR49(GAC)	+	+	-	72	-	9/6/9-	-41	-11	-12
Pa19-tR49(CAT)	+	+	-	72	-	9/6/9-	-43	-11	-12
Pa19-tR8(TAG)	+	+	-	72	-	10/6/9+	-42	-17	-11
Pa19-tR8(CAG)	+	+	-	72	-	10/6/9+	-42	-17	-11
Pa19-tR23(CAT)[3]	+	+	-	72	-	10/5/9+	-39	-10	-10
Pa21-S598	+	+	+	76	-	9/6/9-	-32	-15	-11
Pa21-S1314[4]	+	+	+	76	-	10/5/9+	-32	-18	-1.1
Pa21-L1576[5]	+	+	+	76	-	10/5/9+	-29	-18	-8.7
Pa21-tR21(TGT)	+	+	+	76	-	10/5/9+	-28	-17	-0.7
Pa21-tR21(CGT)	+	+	+	76	-	10/5/9+	-28	-17	-0.7
Pa21-tR4(GAA)	+	+	+	76	-	10/5/9+	-28	-17	-0.7
Pa35.1-L1318	+	+	+	70	-	9/7/9-	-28	-14	-9.5
Pa35.1-tR26(TTG)	+	+	+	70	-	9/7/9-	-28	-14	-7.8
Pa35.2-tR48(TAC)	+	+	+	72	-	9/7/9-	-27	-15	-12
Pa35.2-tR48(CAC)	+	+	+	72	-	9/7/9-	-27	-15	-12
Pa40.1-S81	+	+	-	69	-	9/6/9-	-29	-11	-5.9
Pa40.2-L2721	+	+	-	74	-	9/7/8-	-46	-13	-8.6
Pa40.2-L2646[6]	+	+	-	74	-	10/6/8+	-46	-9.4	-6.6
Pa40.2-L1677[7]	+	+	-	74	-	10/6/8+	-46	-14	-7.3
Pa40.2-L2720[6]	+	+	-	74	-	10/6/8+	-46	-15	-8.6
Pa40.3-L2611	+	+	-	63	-	9/6/9-	-30	-12	-2.9
Pa105.1-S798	+	+	+	76	-	8/7/9-	-35	-13	-1.1
Pa105.1-L1780	+	+	+	76	-	8/7/9-	-36	-12	-3.4
Pa105.1-S611	+	+	+	76	-	9/6/9-	-34	-14	-1.0
Pa105.1-L1937[4]	+	+	+	76	-	10/6/9+	-33	-11	-1.0
Pa105.2-S40	+	+	+	71	-	8/8/9-	-34	-14	-8.6
Pa105.2-L2530	+	+	+	71	-	8/8/9-	-34	-16	-2.6
Pa105.2-L1722	+	+	+	71	-	8/8/9-	-34	-16	-7.1
Pa105.2-tR27(GGG)	+	+	+	71	-	10/6/9+	-34	-14	-8.3
Pa160-L655	+	+	+	69	-	8/7/9-	-38	-21	-10
Pa160-S518	+	+	+	69	-	9/6/9-	-38	-10	-11
Pa160-L1318[2]	+	+	+	69	-	10/5/9+	-38	-16	-10

the listed pairs are selected according to the following criteria: energy cutoff of -16 kcal/mol as calculated by RIsearch (53); no bulge nucleotide allowed on the 5’ duplex of the guide RNA, maximum of 2 bulge nucleotides on the 3’ duplex.

[a] as provided by the UCSC genome browser (41, 42). [b] as determined from RNA-seq data (36). [c] experimental evidence for modified position in rRNAs or tRNAs. [d] structure-function model as proposed from the ’productive’/’non-productive’ classification (the +/- sign indicates whether it is predicted to be productive or not). [e] as calculated by RNAsnoop (kcal/mol) from the Vienna RNA package (44). [f] the duplex energy includes both the 5’ and 3’ duplex energies with a correction factor (+4.1 kcal/mol).

List of base-pairing anomalies next to the targeted U position:

[1] absence of base pair at the first position of the 3’ duplex on the guide (bulge position);

[2] presence of 3 nucleotides in the target spacer between the 5’ and 3’ duplexes;

[3] presence of a mismatch at the first position of the 3’ duplex;

[4] 5’ duplex below the -6 kcal/mol energy cutoff;

[5] presence of two successive wobble pairs in the 5’ duplex prior to the targeted position;

[6] presence of a mismatch in the 5’ duplex prior to the targeted position;

[7] presence of a mismatch in the 5’ duplex upstream to the targeted position (2nt).

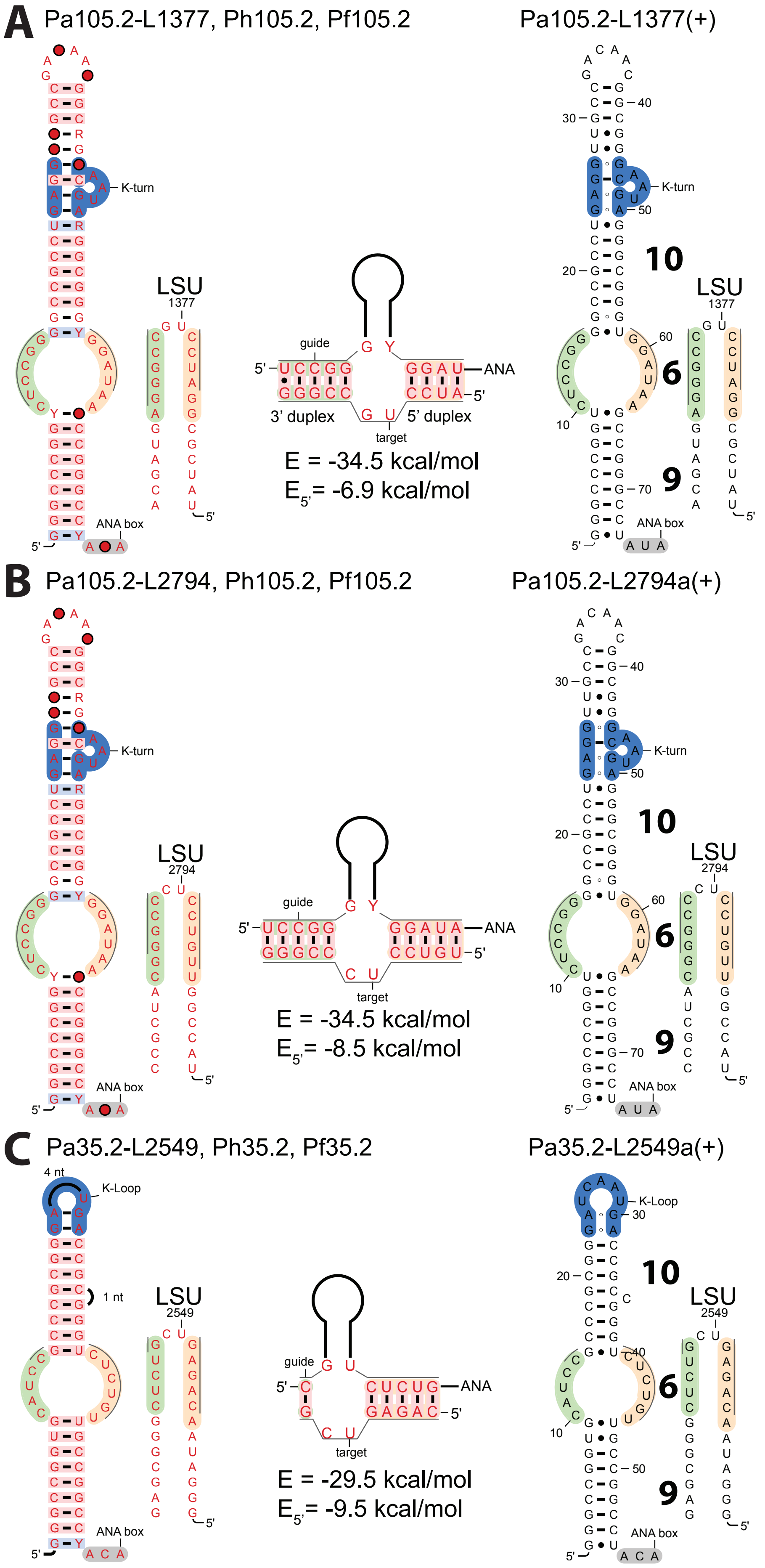

Figure S9. Models of productive (+) guide:target pairs Pa105.2-L1377, Pa105.2-L2795 and Pa35.2-L2549. **A.** Productive fold for Pa105.2 and its true target L1377. **B.** Productive fold for Pa105.2 and its false target L2795. **C.** Productive fold for Pa35.2 and its true target L2549. The duplexes are represented with the following color code: orange for the 5' duplex, green for the 3' duplex. The energy values are those calculated by RNAsnoop.

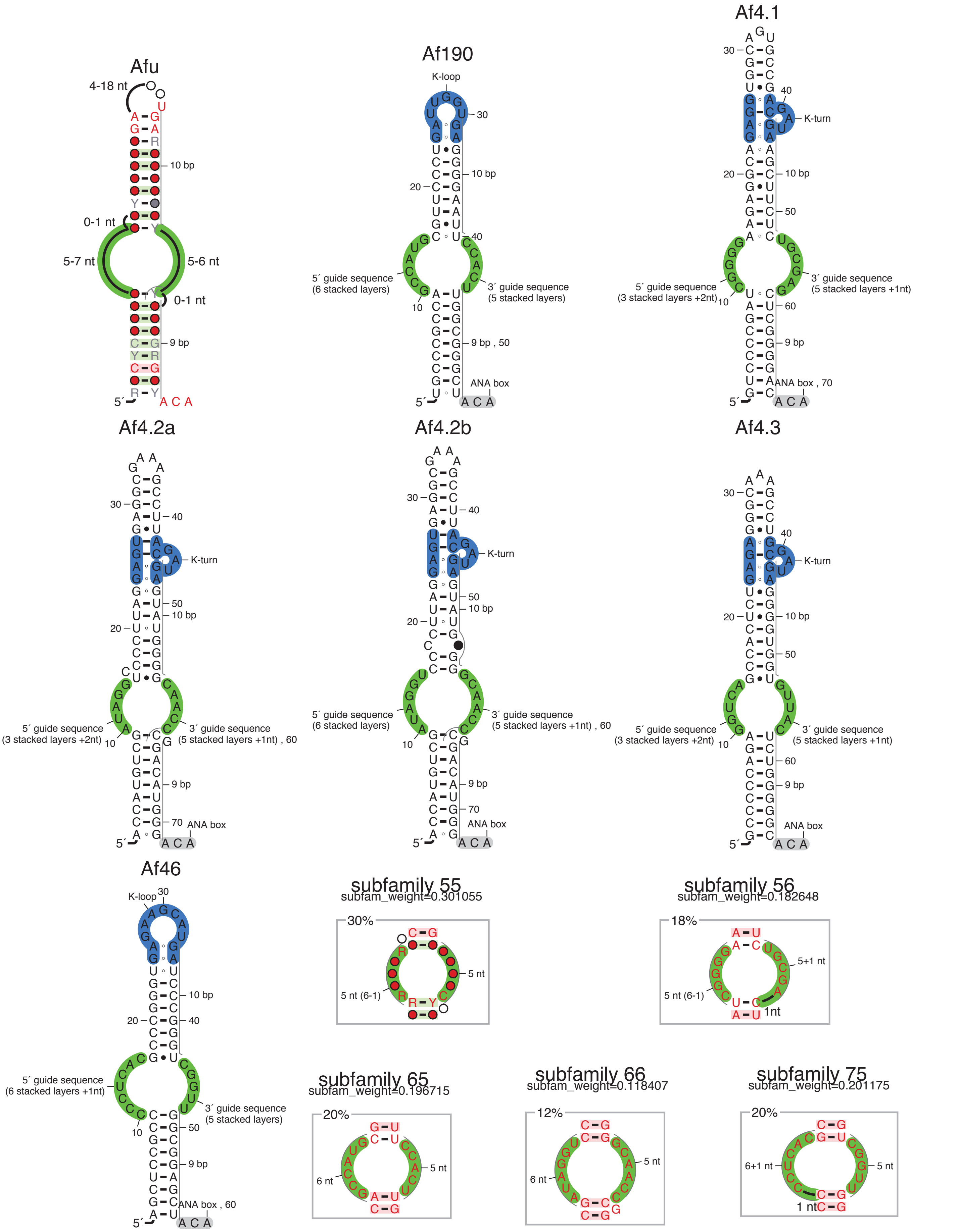

Figure S10. Classification of H/ACA motifs in *Archaeoglobus fulgidus*.

Listing 20: Stockholm Alignment used in R2R representations of the different subfamilies of H/ACA guide RNAs in *Archaeoglobus fulgidus*.

```
# STOCKHOLM 1.0

Af4.1                            GUCCCCGAU.......CGGGGA.AGAGGCA...GA....GGU.G.GC...AGU.......GCCGAC...GAUGA.AGCU.UC.UCUGCGAG.C.UCGGGGAC..ACA
Af4.2a                           ACCAUGUCG.......AUAGGUCCCCUUAG...GA....GUG.A.GGC.GAAA......GCCUUAC...GAUGA.GUAU.GG.GGCAACC..CGGACAUGGG..ACA
Af4.2b                           ACCAUGUCG......AUAGGUC.CCCUUAG...GA....GUG.A.GGC.GAAA......GCCUUAC...GAUGA.GUAU.G..GGGCAACC.CGGACAUGGG..ACA
Af4.3                            GCCCCCAGA.......GGUCAG.CCACUCU...GA....GAG.G.GC...AAA.......GCCUGC...GAUGA.GGGG.UG.GUGUUAC..U.CUGGGGGC..ACA
Af46                             AGCUCCGCC.....CCCUCACG.CCCGGGU...GA..............GAAGCA................UGA.UCCC.GG.GUCGGUU..G.GCGGAGCU..ACA
Af190                            UGCCCGCCA......GCCAUGC.GUUCCCU...GA..............UUGG..................UGA.GGGG.AA.UUCCACU..U.GGCGGGCU..ACA
#=GC SS_cons                     <<<<<<<<<............<-<<<<<<<...<<....<<<.<.<<<<_________>>>>>>>>...-..>>.>>>>->>->>......->->>>>>>>>.....
#=GC R2R_LABEL                   q............1......2.3s.........II...4.p..................p......5.....HH.HHiH.HtBHH6....78H9HHHhHHHr.....
#=GC R2R_XLABEL_KTURN            .................................TTTTTTTT.......................RRRRRRRRRR.................................
#=GC R2R_XLABEL_KTURNL           ......................................................................r....................................
#=GC R2R_XLABEL_KLOOP            .................................KKKKKKKKKKKKKKKKKKKKKKKKKKKKKKKKKKKKKKKKK.................................
#=GC R2R_XLABEL_KLOOPL           ...................................................k.......................................................
#=GC R2R_XLABEL_ILOOP            ..............PQXXXXX.W..............................................................YYYYYSU.V..........ZZZ
#=GC R2R_XLABEL_ILOOPL           .................x....w.................................................................y.su.v............z
#=GC R2R_XLABEL_STEM             ...........................................................................................................
#=GC R2R_XLABEL_STEML            ...........................................................................................................
#=GC SUBFAM_LABEL_TERM1          .............xxxxxxxx......................................................................................
#=GC SUBFAM_LABEL_TERM2          .....................................................................................xxxxxxx...............
#=GC SUBFAM_GUIDE55_R2R_LABEL  -------<.....1PQXxXXX.W>-----------------------------------------------------------<.YYyYYSU.V>------------
#=GC SUBFAM_GUIDE56_R2R_LABEL  -------<.....1PQXxXXX.W>-----------------------------------------------------------<.YYyYYSU.V>------------
#=GC SUBFAM_GUIDE65_R2R_LABEL  -------<.....1PQXxXXX.W>-----------------------------------------------------------<.YYyYYSU.V>------------
#=GC SUBFAM_GUIDE66_R2R_LABEL  -------<.....1PQXxXXX.W>-----------------------------------------------------------<.YYyYYSU.V>------------
#=GC SUBFAM_GUIDE75_R2R_LABEL  -------<.....1PQXxXXX.W>-----------------------------------------------------------<.YYyYYSU.V>------------

#=GF R2R keep q r
#=GF R2R keep s t
#=GF R2R var_backbone_range_size_fake_nucs 7 1 2 5-7 nt
#=GF R2R var_backbone_range_size_fake_nucs 6 6 7 5-6 nt
#=GF R2R var_backbone_range 4 5
#=GF R2R var_backbone_range_size_fake_nucs 1 3 3
#=GF R2R var_backbone_range_size_fake_nucs 1 9 9

#=GF SUBFAM_REGEX_PRED HAS_GUIDE5 TERM1 ^[.]*[-.][-.][-.][A-Z][A-Z][A-Z][A-Z][A-Z][.]*$
#=GF SUBFAM_REGEX_PRED HAS_GUIDE6 TERM1 ^[.]*[-.][-.][A-Z][A-Z][A-Z][A-Z][A-Z][A-Z][.]*$
#=GF SUBFAM_REGEX_PRED HAS_GUIDE7 TERM1 ^[.]*[-.][A-Z][A-Z][A-Z][A-Z][A-Z][A-Z][A-Z][.]*$
#=GF SUBFAM_REGEX_PRED HAS_GUIDE6B TERM2 ^[.]*[A-Z][A-Z][A-Z][A-Z][A-Z][A-Z][-.][.]*$
#=GF SUBFAM_REGEX_PRED HAS_GUIDE7B TERM2 ^[.]*[A-Z][A-Z][A-Z][A-Z][A-Z][A-Z][A-Z][.]*$
#=GF SUBFAM_REGEX_PRED HAS_GUIDE5B TERM2 ^[.]*[A-Z][A-Z][A-Z][A-Z][A-Z][-.][-.][.]*$
#=GF SUBFAM_PERL_PRED GUIDE55 return $predValue{HAS_GUIDE5} && $predValue{HAS_GUIDE5B};
#=GF SUBFAM_PERL_PRED GUIDE56 return $predValue{HAS_GUIDE5} && $predValue{HAS_GUIDE6B};
#=GF SUBFAM_PERL_PRED GUIDE65 return $predValue{HAS_GUIDE6} && $predValue{HAS_GUIDE5B};
#=GF SUBFAM_PERL_PRED GUIDE66 return $predValue{HAS_GUIDE6} && $predValue{HAS_GUIDE6B};
#=GF SUBFAM_PERL_PRED GUIDE75 return $predValue{HAS_GUIDE7} && $predValue{HAS_GUIDE5B};

#=GF SUBFAM_GUIDE55_R2R no5
#=GF SUBFAM_GUIDE55_R2R set_dir pos0 -90
#=GF SUBFAM_GUIDE55_R2R shade_along_backbone ILOOP:P rgb:0,255,0
#=GF SUBFAM_GUIDE55_R2R shade_along_backbone ILOOP:Q rgb:0,255,0
#=GF SUBFAM_GUIDE55_R2R shade_along_backbone ILOOP:X rgb:0,255,0
#=GF SUBFAM_GUIDE55_R2R outline_nuc X
#=GF SUBFAM_GUIDE55_R2R outline_nuc x
#=GF SUBFAM_GUIDE55_R2R tick_label x 5 nt (6-1)
#=GF SUBFAM_GUIDE55_R2R shade_along_backbone ILOOP:Y rgb:0,255,0
#=GF SUBFAM_GUIDE55_R2R shade_along_backbone ILOOP:S rgb:0,255,0
#=GF SUBFAM_GUIDE55_R2R shade_along_backbone ILOOP:U rgb:0,255,0
#=GF SUBFAM_GUIDE55_R2R outline_nuc Y
#=GF SUBFAM_GUIDE55_R2R outline_nuc y
#=GF SUBFAM_GUIDE55_R2R tick_label y 5 nt

#=GF SUBFAM_GUIDE56_R2R no5
#=GF SUBFAM_GUIDE56_R2R set_dir pos0 -90
#=GF SUBFAM_GUIDE56_R2R shade_along_backbone ILOOP:P rgb:0,255,0
#=GF SUBFAM_GUIDE56_R2R shade_along_backbone ILOOP:Q rgb:0,255,0
#=GF SUBFAM_GUIDE56_R2R shade_along_backbone ILOOP:X rgb:0,255,0
#=GF SUBFAM_GUIDE56_R2R outline_nuc X
#=GF SUBFAM_GUIDE56_R2R outline_nuc x
#=GF SUBFAM_GUIDE56_R2R tick_label x 5 nt (6-1)
#=GF SUBFAM_GUIDE56_R2R shade_along_backbone ILOOP:Y rgb:0,255,0
#=GF SUBFAM_GUIDE56_R2R shade_along_backbone ILOOP:S rgb:0,255,0
#=GF SUBFAM_GUIDE56_R2R shade_along_backbone ILOOP:U rgb:0,255,0
#=GF SUBFAM_GUIDE56_R2R outline_nuc Y
#=GF SUBFAM_GUIDE56_R2R outline_nuc y
#=GF SUBFAM_GUIDE56_R2R tick_label y 5+1 nt
#=GF SUBFAM_GUIDE56_R2R var_backbone_range_size_fake_nucs 1 S S 1nt

#=GF SUBFAM_GUIDE65_R2R no5
#=GF SUBFAM_GUIDE65_R2R set_dir pos0 -90
#=GF SUBFAM_GUIDE65_R2R shade_along_backbone ILOOP:P rgb:0,255,0
#=GF SUBFAM_GUIDE65_R2R shade_along_backbone ILOOP:Q rgb:0,255,0
#=GF SUBFAM_GUIDE65_R2R shade_along_backbone ILOOP:X rgb:0,255,0
#=GF SUBFAM_GUIDE65_R2R outline_nuc Q
#=GF SUBFAM_GUIDE65_R2R outline_nuc X
#=GF SUBFAM_GUIDE65_R2R outline_nuc x
#=GF SUBFAM_GUIDE65_R2R tick_label x 6 nt
#=GF SUBFAM_GUIDE65_R2R shade_along_backbone ILOOP:Y rgb:0,255,0
#=GF SUBFAM_GUIDE65_R2R shade_along_backbone ILOOP:S rgb:0,255,0
#=GF SUBFAM_GUIDE65_R2R shade_along_backbone ILOOP:U rgb:0,255,0
#=GF SUBFAM_GUIDE65_R2R outline_nuc Y
#=GF SUBFAM_GUIDE65_R2R outline_nuc y
#=GF SUBFAM_GUIDE65_R2R tick_label y 5 nt

#=GF SUBFAM_GUIDE66_R2R no5
#=GF SUBFAM_GUIDE66_R2R set_dir pos0 -90
#=GF SUBFAM_GUIDE66_R2R shade_along_backbone ILOOP:P rgb:0,255,0
#=GF SUBFAM_GUIDE66_R2R shade_along_backbone ILOOP:Q rgb:0,255,0
#=GF SUBFAM_GUIDE66_R2R shade_along_backbone ILOOP:X rgb:0,255,0
#=GF SUBFAM_GUIDE66_R2R outline_nuc Q
#=GF SUBFAM_GUIDE66_R2R outline_nuc X
#=GF SUBFAM_GUIDE66_R2R outline_nuc x
#=GF SUBFAM_GUIDE66_R2R tick_label x 6 nt
#=GF SUBFAM_GUIDE66_R2R shade_along_backbone ILOOP:Y rgb:0,255,0
#=GF SUBFAM_GUIDE66_R2R shade_along_backbone ILOOP:S rgb:0,255,0
#=GF SUBFAM_GUIDE66_R2R shade_along_backbone ILOOP:U rgb:0,255,0
#=GF SUBFAM_GUIDE66_R2R outline_nuc Y
#=GF SUBFAM_GUIDE66_R2R outline_nuc y
```

```
#=GF SUBFAM_GUIDE66_R2R tick_label y 5 nt

#=GF SUBFAM_GUIDE75_R2R no5
#=GF SUBFAM_GUIDE75_R2R set_dir pos0 -90
#=GF SUBFAM_GUIDE75_R2R shade_along_backbone ILOOP:P rgb:0,255,0
#=GF SUBFAM_GUIDE75_R2R shade_along_backbone ILOOP:X rgb:0,255,0
#=GF SUBFAM_GUIDE75_R2R shade_along_backbone ILOOP:Q rgb:0,255,0
#=GF SUBFAM_GUIDE75_R2R outline_nuc Q
#=GF SUBFAM_GUIDE75_R2R outline_nuc X
#=GF SUBFAM_GUIDE75_R2R outline_nuc x
#=GF SUBFAM_GUIDE75_R2R outline_nuc P
#=GF SUBFAM_GUIDE75_R2R tick_label x 6+1 nt
#=GF SUBFAM_GUIDE75_R2R shade_along_backbone ILOOP:Y rgb:0,255,0
#=GF SUBFAM_GUIDE75_R2R shade_along_backbone ILOOP:S rgb:0,255,0
#=GF SUBFAM_GUIDE75_R2R shade_along_backbone ILOOP:U rgb:0,255,0
#=GF SUBFAM_GUIDE75_R2R outline_nuc Y
#=GF SUBFAM_GUIDE75_R2R outline_nuc y
#=GF SUBFAM_GUIDE75_R2R tick_label y 5 nt
#=GF SUBFAM_GUIDE75_R2R var_backbone_range_size_fake_nucs 1 P P

#=GF R2R shade_along_backbone 1 rgb:0,255,0
#=GF R2R shade_along_backbone 6 rgb:0,255,0
#=GF R2R outline_nuc H
#=GF R2R outline_nuc h
#=GF R2R outline_nuc r
#=GF R2R outline_nuc i
#=GF R2R outline_nuc t
#=GF R2R tick_label h 9 bp
#=GF R2R tick_label i 10 bp

#=GF R2R_oneseq Af4.1 shade_along_backbone KTURN:T rgb:0,129,255
#=GF R2R_oneseq Af4.1 shade_along_backbone KTURN:R rgb:0,129,255
#=GF R2R_oneseq Af4.1 tick_label KTURNL:r K-turn
#=GF R2R_oneseq Af4.1 shade_along_backbone ILOOP:Y rgb:0,255,0
#=GF R2R_oneseq Af4.1 shade_along_backbone ILOOP:S rgb:0,255,0
#=GF R2R_oneseq Af4.1 shade_along_backbone ILOOP:U rgb:0,255,0
#=GF R2R_oneseq Af4.1 tick_label ILOOPL:y 3' guide sequence \n (5 stacked layers +1nt)
#=GF R2R_oneseq Af4.1 shade_along_backbone ILOOP:P rgb:0,255,0
#=GF R2R_oneseq Af4.1 shade_along_backbone ILOOP:Q rgb:0,255,0
#=GF R2R_oneseq Af4.1 shade_along_backbone ILOOP:X rgb:0,255,0
#=GF R2R_oneseq Af4.1 tick_label ILOOPL:x 5' guide sequence \n (6 stacked layers)
#=GF R2R_oneseq Af4.1 shade_along_backbone ILOOP:Z rgb:200,200,200
#=GF R2R_oneseq Af4.1 tick_label ILOOPL:z ANA box
#=GF R2R_oneseq Af4.1 var_backbone_range_size_fake_nucs 1 8 8

#=GF R2R_oneseq Af4.2a shade_along_backbone KTURN:T rgb:0,129,255
#=GF R2R_oneseq Af4.2a shade_along_backbone KTURN:R rgb:0,129,255
#=GF R2R_oneseq Af4.2a tick_label KTURNL:r K-turn
#=GF R2R_oneseq Af4.2a shade_along_backbone ILOOP:Y rgb:0,255,0
#=GF R2R_oneseq Af4.2a shade_along_backbone ILOOP:S rgb:0,255,0
#=GF R2R_oneseq Af4.2a shade_along_backbone ILOOP:U rgb:0,255,0
#=GF R2R_oneseq Af4.2a tick_label ILOOPL:y 3' guide sequence \n (5 stacked layers +1nt)
#=GF R2R_oneseq Af4.2a shade_along_backbone ILOOP:P rgb:0,255,0
#=GF R2R_oneseq Af4.2a shade_along_backbone ILOOP:Q rgb:0,255,0
#=GF R2R_oneseq Af4.2a shade_along_backbone ILOOP:X rgb:0,255,0
#=GF R2R_oneseq Af4.2a tick_label ILOOPL:x 5' guide sequence \n (6 stacked layers)
#=GF R2R_oneseq Af4.2a shade_along_backbone ILOOP:Z rgb:200,200,200
#=GF R2R_oneseq Af4.2a tick_label ILOOPL:z ANA box
#=GF R2R_oneseq Af4.2a var_backbone_range_size_fake_nucs 1 8 8

#=GF R2R_oneseq Af4.2b shade_along_backbone KTURN:T rgb:0,129,255
#=GF R2R_oneseq Af4.2b shade_along_backbone KTURN:R rgb:0,129,255
#=GF R2R_oneseq Af4.2b tick_label KTURNL:r K-turn
#=GF R2R_oneseq Af4.2b shade_along_backbone ILOOP:Y rgb:0,255,0
#=GF R2R_oneseq Af4.2b shade_along_backbone ILOOP:S rgb:0,255,0
#=GF R2R_oneseq Af4.2b shade_along_backbone ILOOP:U rgb:0,255,0
#=GF R2R_oneseq Af4.2b tick_label ILOOPL:y 3' guide sequence \n (5 stacked layers +1nt)
#=GF R2R_oneseq Af4.2b shade_along_backbone ILOOP:P rgb:0,255,0
#=GF R2R_oneseq Af4.2b shade_along_backbone ILOOP:Q rgb:0,255,0
#=GF R2R_oneseq Af4.2b shade_along_backbone ILOOP:X rgb:0,255,0
#=GF R2R_oneseq Af4.2b tick_label ILOOPL:x 5' guide sequence \n (6 stacked layers)
#=GF R2R_oneseq Af4.2b shade_along_backbone ILOOP:Z rgb:200,200,200
#=GF R2R_oneseq Af4.2b tick_label ILOOPL:z ANA box
#=GF R2R_oneseq Af4.2b var_backbone_range_size_fake_nucs 1 8 8

#=GF R2R_oneseq Af4.3 shade_along_backbone KTURN:T rgb:0,129,255
#=GF R2R_oneseq Af4.3 shade_along_backbone KTURN:R rgb:0,129,255
#=GF R2R_oneseq Af4.3 tick_label KTURNL:r K-turn
#=GF R2R_oneseq Af4.3 shade_along_backbone ILOOP:Y rgb:0,255,0
#=GF R2R_oneseq Af4.3 shade_along_backbone ILOOP:S rgb:0,255,0
#=GF R2R_oneseq Af4.3 shade_along_backbone ILOOP:U rgb:0,255,0
#=GF R2R_oneseq Af4.3 tick_label ILOOPL:y 3' guide sequence \n (5 stacked layers +1nt)
#=GF R2R_oneseq Af4.3 shade_along_backbone ILOOP:P rgb:0,255,0
#=GF R2R_oneseq Af4.3 shade_along_backbone ILOOP:Q rgb:0,255,0
#=GF R2R_oneseq Af4.3 shade_along_backbone ILOOP:X rgb:0,255,0
#=GF R2R_oneseq Af4.3 tick_label ILOOPL:x 5' guide sequence \n (6 stacked layers)
#=GF R2R_oneseq Af4.3 shade_along_backbone ILOOP:Z rgb:200,200,200
#=GF R2R_oneseq Af4.3 tick_label ILOOPL:z ANA box
#=GF R2R_oneseq Af4.3 var_backbone_range_size_fake_nucs 1 8 8

#=GF R2R_oneseq Af46 shade_along_backbone KLOOP:K rgb:0,129,255
#=GF R2R_oneseq Af46 tick_label KLOOPL:k K-loop
#=GF R2R_oneseq Af46 shade_along_backbone ILOOP:Y rgb:0,255,0
#=GF R2R_oneseq Af46 shade_along_backbone ILOOP:S rgb:0,255,0
#=GF R2R_oneseq Af46 shade_along_backbone ILOOP:U rgb:0,255,0
#=GF R2R_oneseq Af46 tick_label ILOOPL:y 3' guide sequence \n (5 stacked layers)
#=GF R2R_oneseq Af46 shade_along_backbone ILOOP:P rgb:0,255,0
#=GF R2R_oneseq Af46 shade_along_backbone ILOOP:Q rgb:0,255,0
#=GF R2R_oneseq Af46 shade_along_backbone ILOOP:X rgb:0,255,0
#=GF R2R_oneseq Af46 tick_label ILOOPL:x 5' guide sequence \n (6 stacked layers)
#=GF R2R_oneseq Af46 shade_along_backbone ILOOP:Z rgb:200,200,200
#=GF R2R_oneseq Af46 tick_label ILOOPL:z ANA box

#=GF R2R_oneseq Af190 shade_along_backbone KLOOP:K rgb:0,129,255
#=GF R2R_oneseq Af190 tick_label KLOOPL:k K-loop
#=GF R2R_oneseq Af190 shade_along_backbone ILOOP:Y rgb:0,255,0
#=GF R2R_oneseq Af190 shade_along_backbone ILOOP:S rgb:0,255,0
#=GF R2R_oneseq Af190 shade_along_backbone ILOOP:U rgb:0,255,0
#=GF R2R_oneseq Af190 tick_label ILOOPL:y 3' guide sequence \n (5 stacked layers)
#=GF R2R_oneseq Af190 shade_along_backbone ILOOP:P rgb:0,255,0
#=GF R2R_oneseq Af190 shade_along_backbone ILOOP:Q rgb:0,255,0
```

```
#=GF R2R_oneseq Af190 shade_along_backbone ILOOP:X rgb:0,255,0
#=GF R2R_oneseq Af190 tick_label ILOOPL:x 5' guide sequence \n (6 stacked layers)
#=GF R2R_oneseq Af190 shade_along_backbone ILOOP:Z rgb:200,200,200
#=GF R2R_oneseq Af190 tick_label ILOOPL:z ANA box

//
```

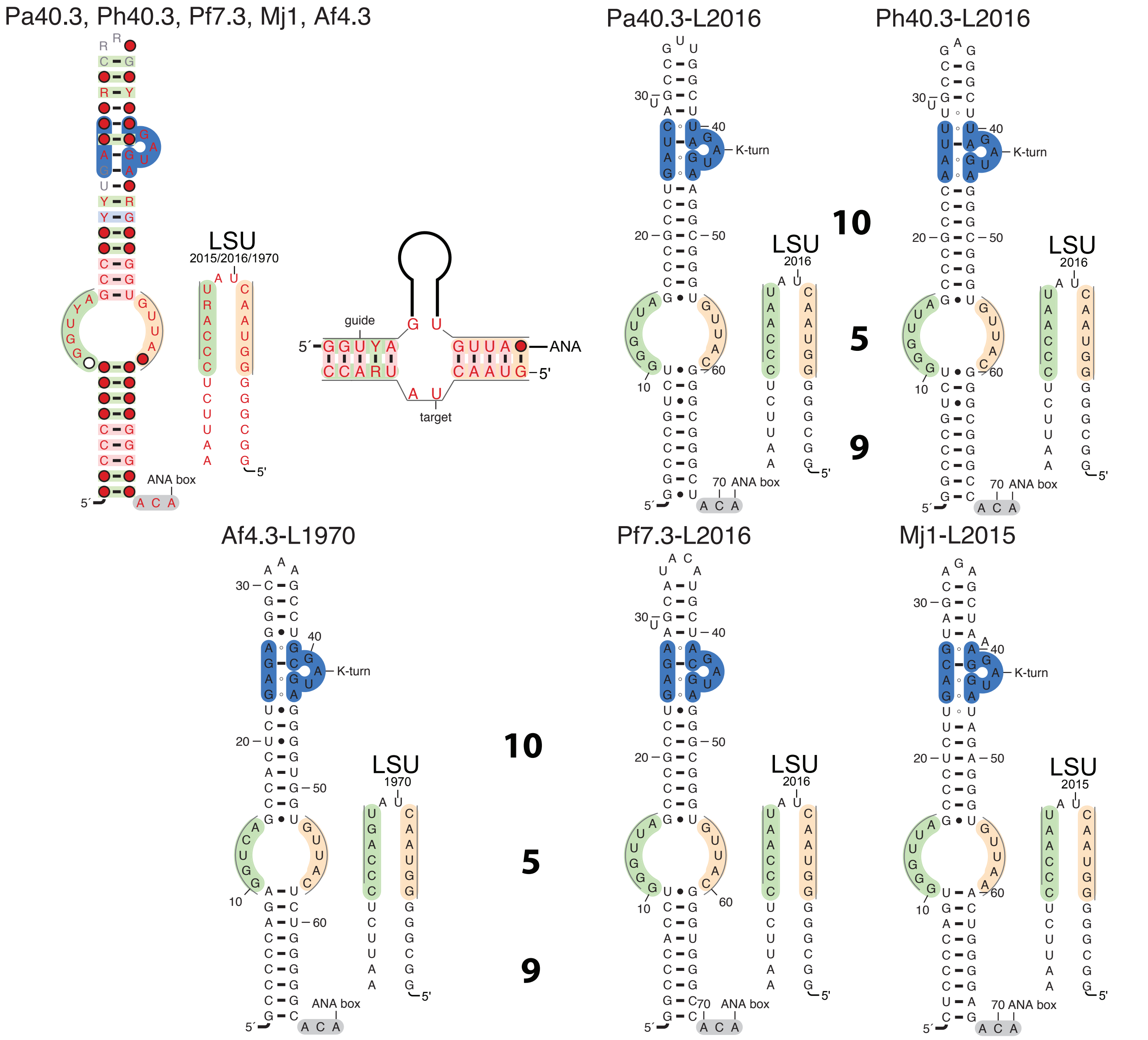

Figure S11. Models of productive guide:target pairs for Pa40.3 (*Pyrococcus abyssi*) and its orthologs in *Pyrococcus furiosus*, *Pyrococcus horikoshii*, *Archaeoglobus fulgisus*, and *Methanocaldococcus jannaschii*.

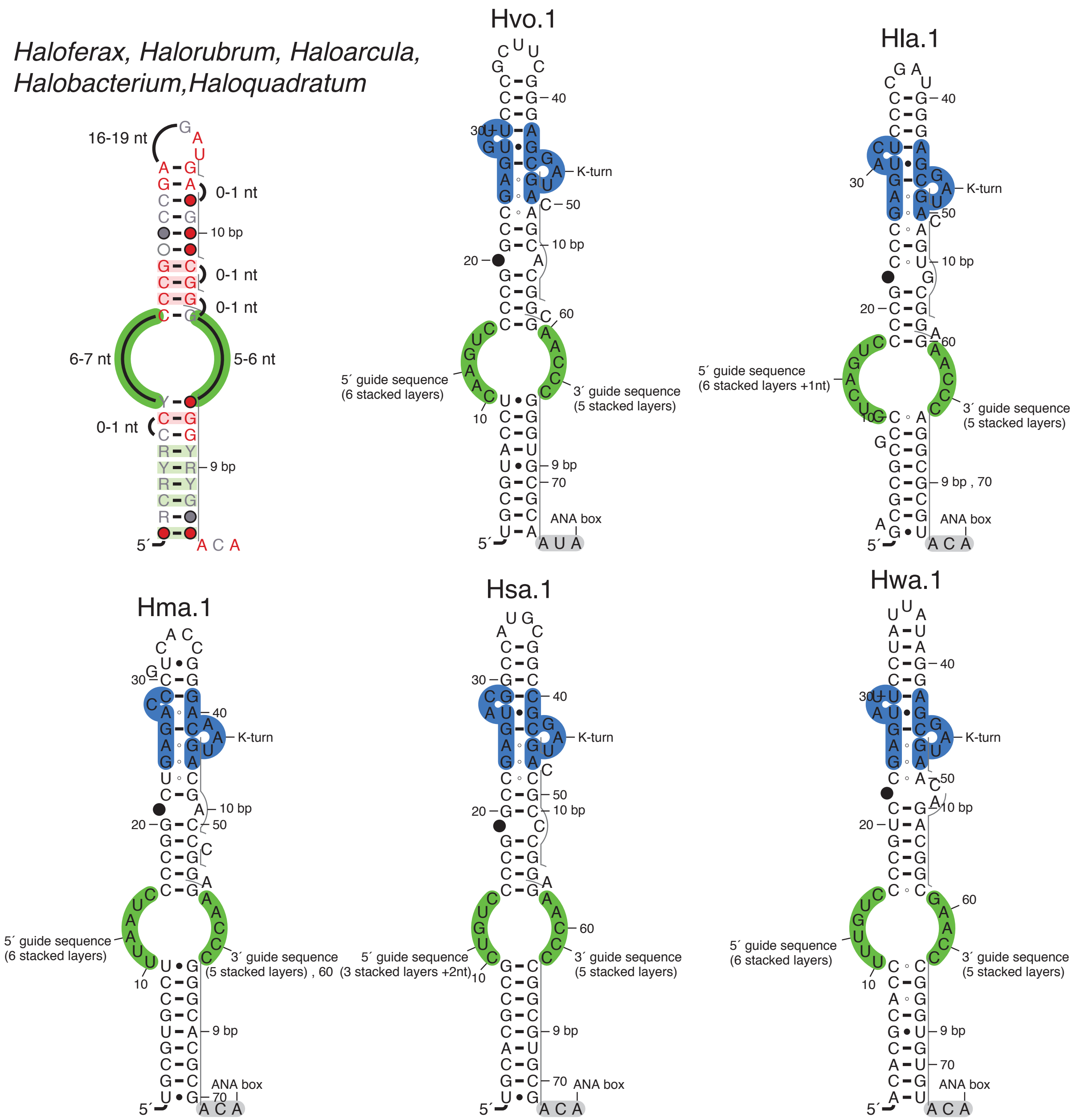

Figure S12. H/ACA motifs Hvo1 (chr:2,499,847-2,499,925) from *Haloferax volcanii* and related species: *Halorubrum lacusprofundi*, *Haloarcula marismortui*, *Halobacterium salinarum*, *Haloquadratum walsbyi*.

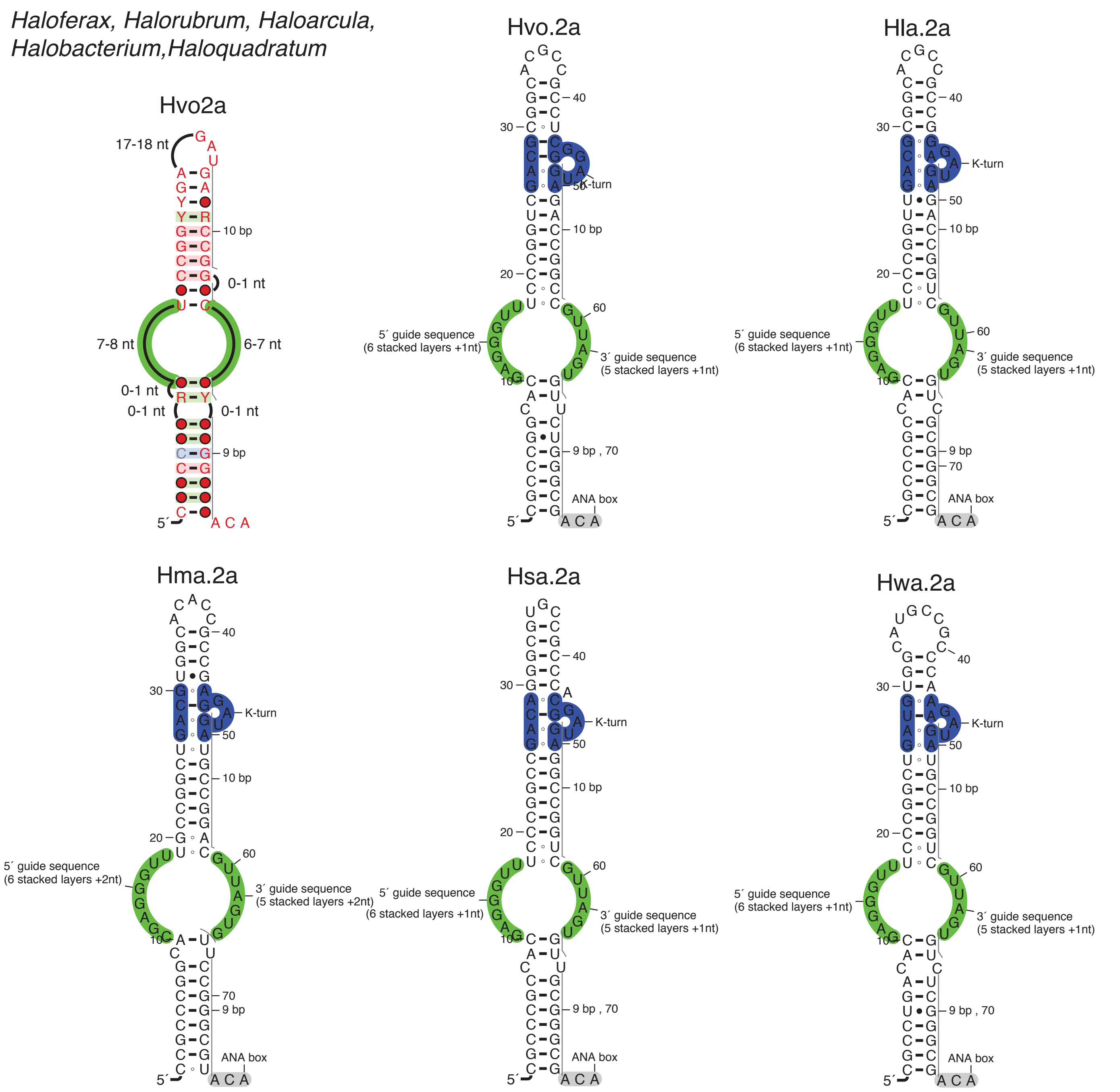

Figure S13. H/ACA motifs Hvo2a (chr:2,499,976-2,500,052) from *Haloferax volcanii* and related species: *Halorubrum lacusprofundi*, *Haloarcula marismortui*, *Halobacterium salinarum*, *Haloquadratum walsbyi*.

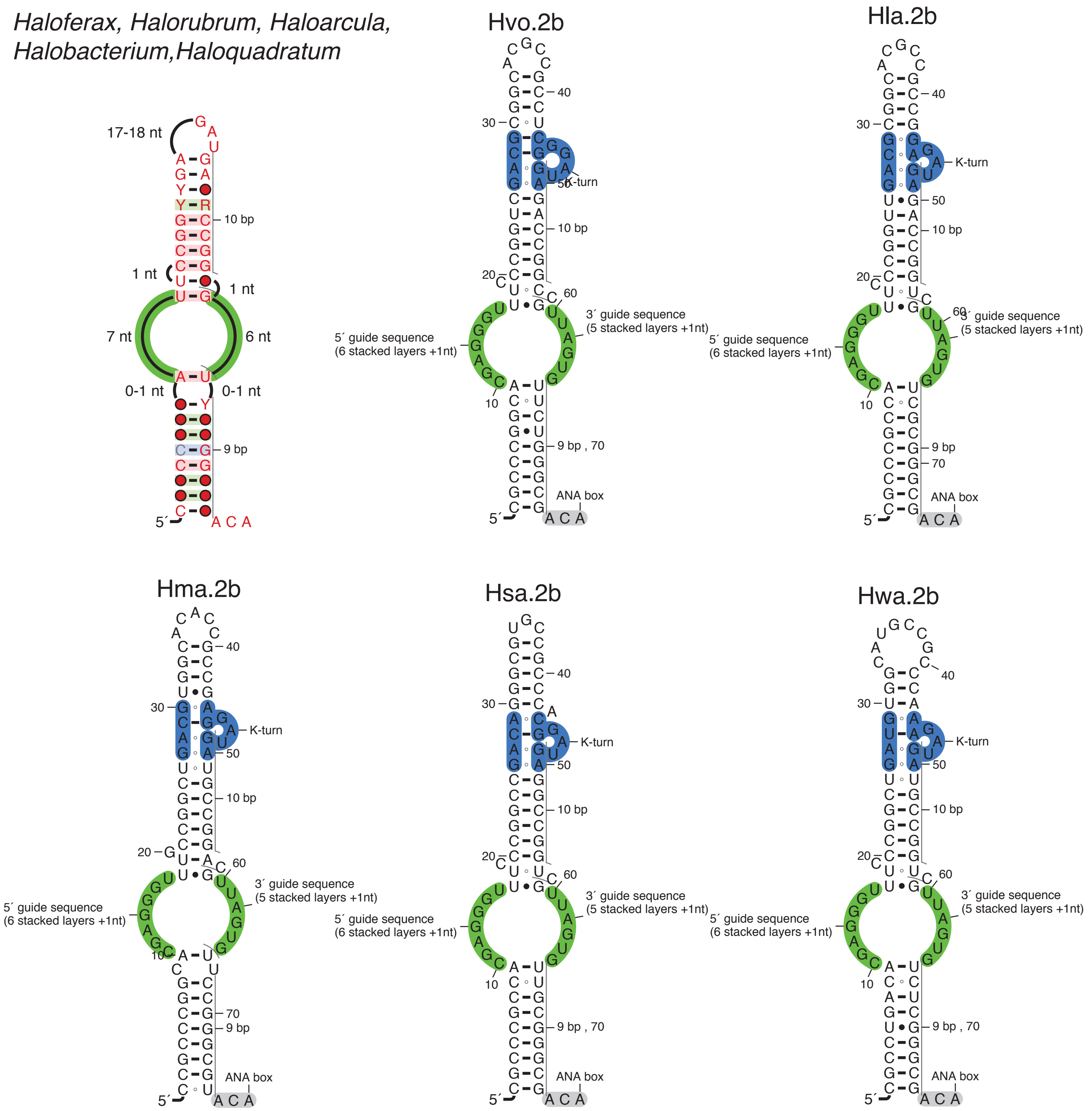

Figure S14. H/ACA motifs Hvo2b (chr:2,499,976-2,500,052) from *Haloferax volcanii* and related species: *Halorubrum lacusprofundi*, *Haloarcula marismortui*, *Halobacterium salinarum*, *Haloquadratum walsbyi*.

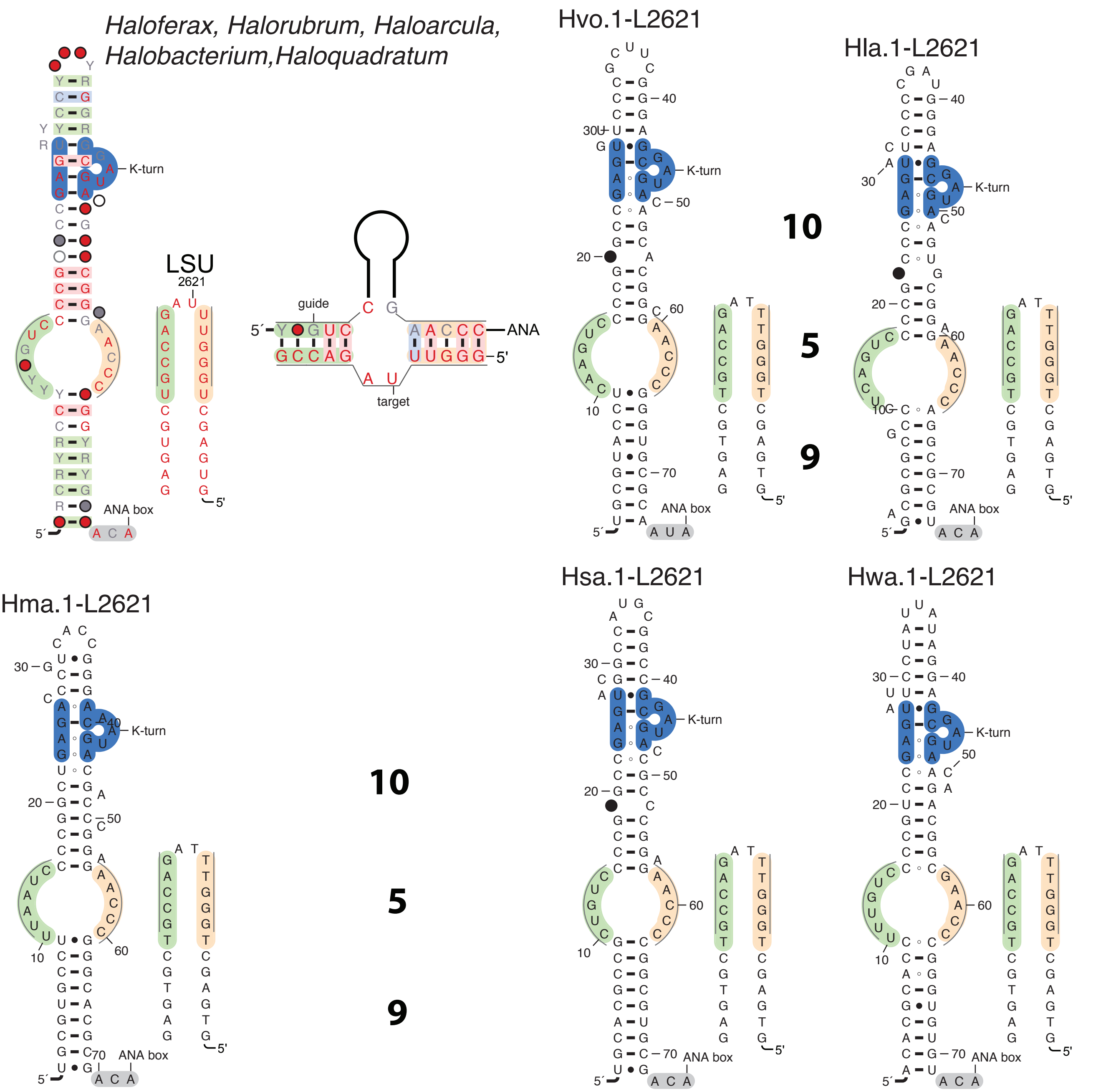

Figure S15. Model of guide:target pair Hvo1-L2621 from *Haloferax volcanii* and related species: *Halorubrum lacusprofundi*, *Haloarcula marismortui*, *Halobacterium salinarum*, *Haloquadratum walsbyi*.

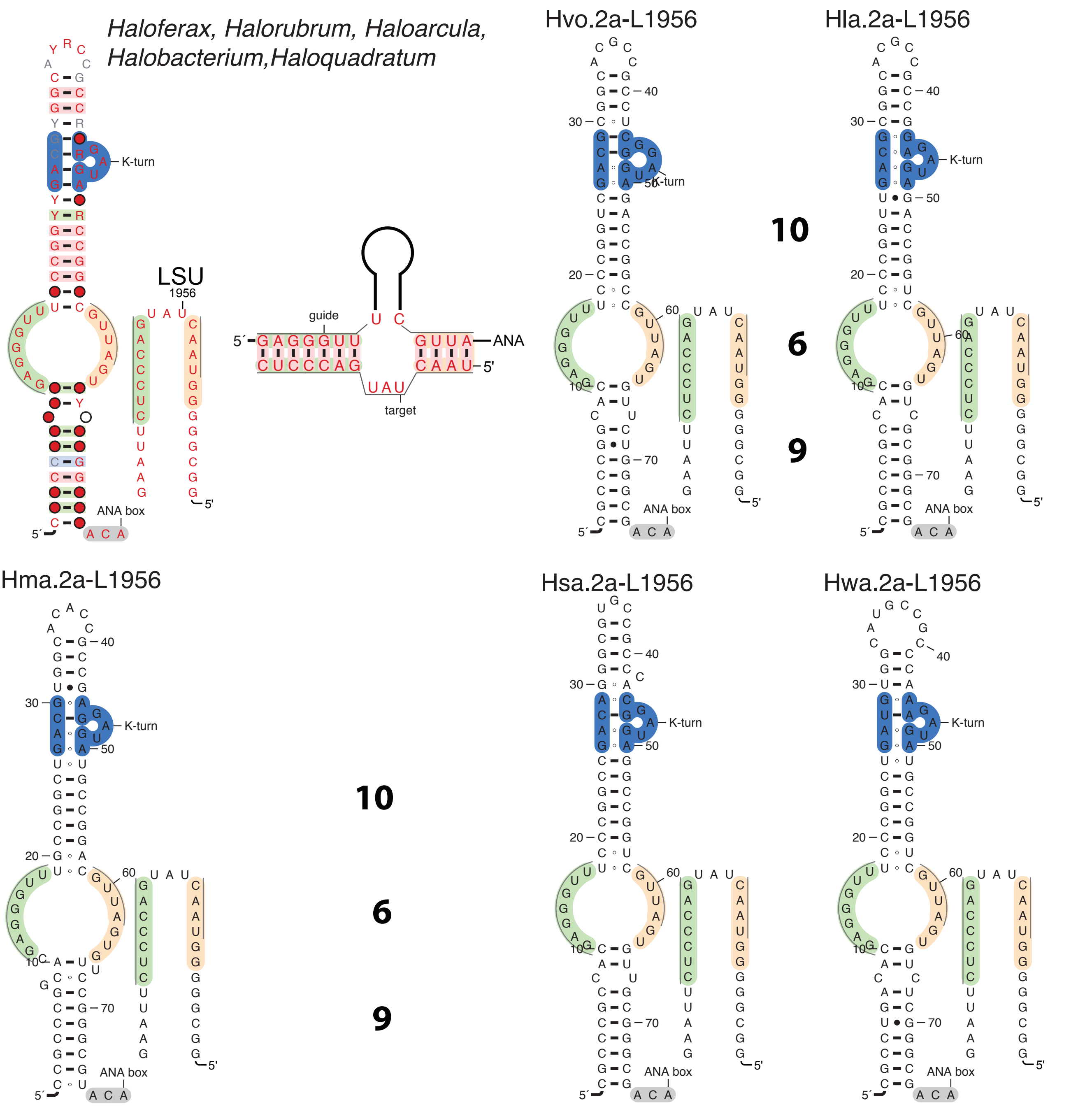

Figure S16. Model of guide:target pair Hvo2-L1956 from *Haloferax volcanii* and related species: *Halorubrum lacusprofundi*, *Haloarcula marismortui*, *Halobacterium salinarum*, *Haloquadratum walsbyi*.

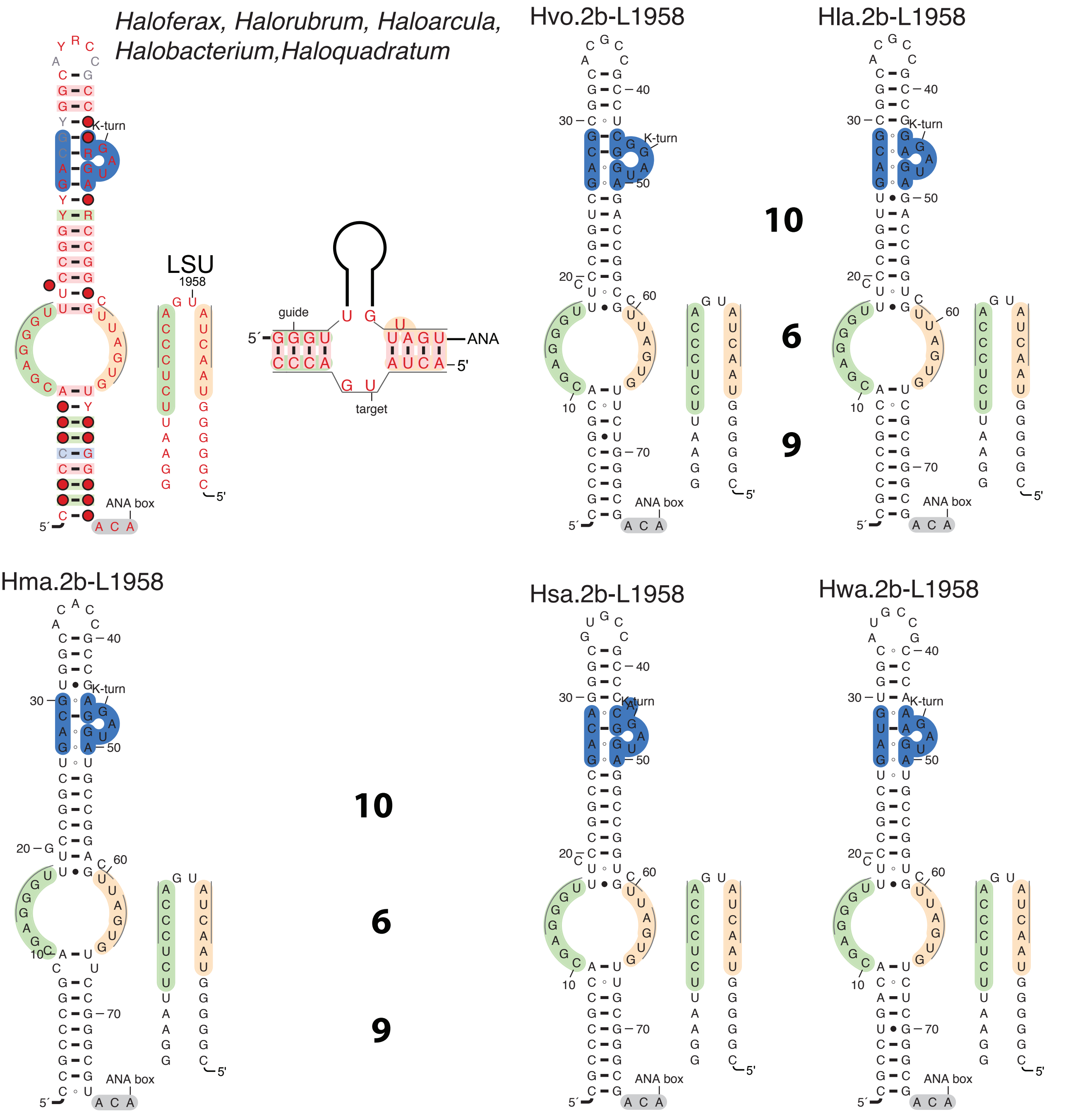

Figure S17. Model of guide:target pair Hvo2-L1958 from *Haloferax volcanii* an related species: *Halorubrum lacusprofundi*, *Haloarcula marismortui*, *Halobacterium salinarum*, *Haloquadratum walsbyi*.

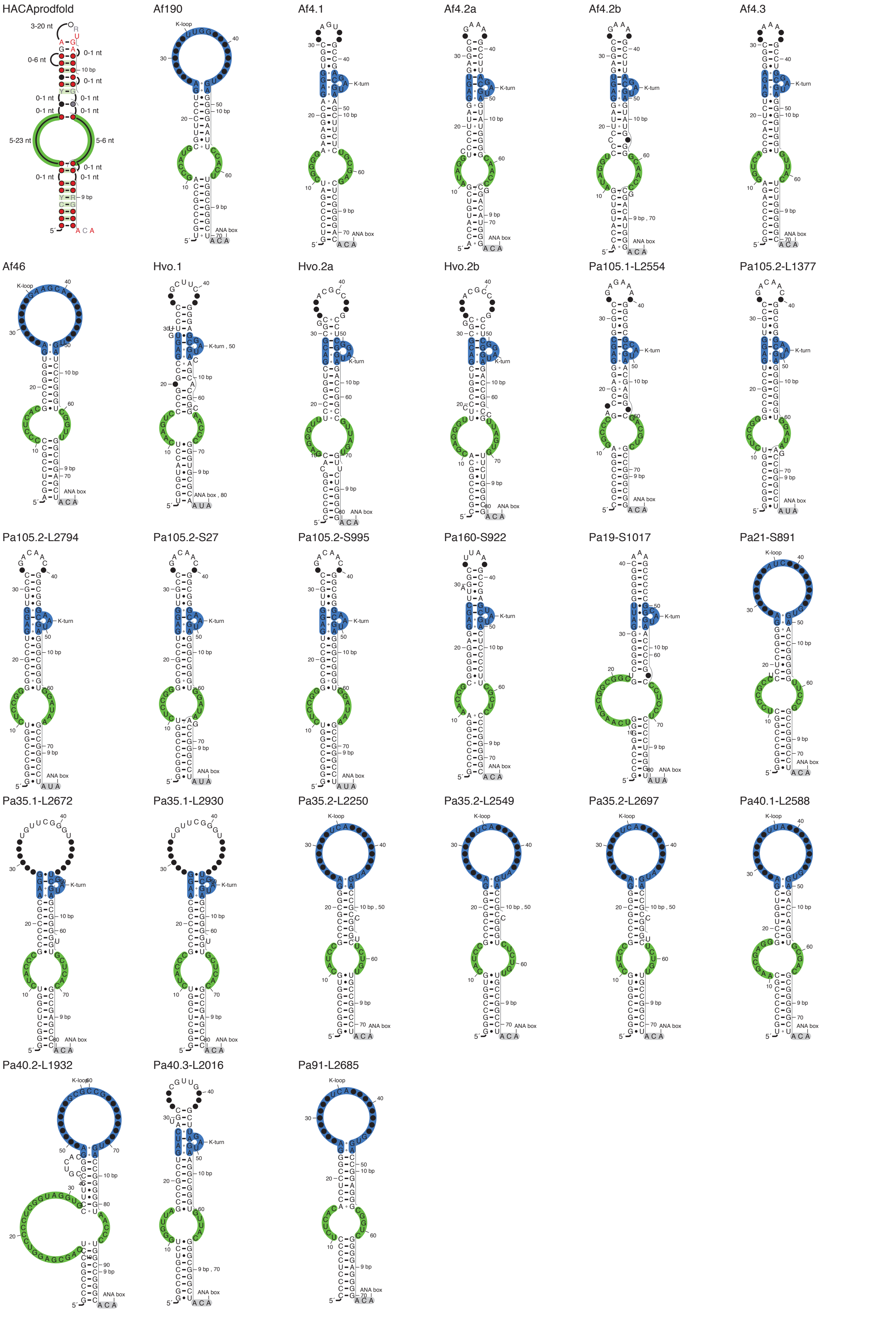

Figure S18. Productive H/ACA folds in *Pyrococcus abyssi*, *Archaeoglobus fulgidus* and *Haloferax volcanii*.

Listing 21: Stockholm Aligmnent used in R2R representations of the Structure/Function Model of productive H/ACA folds in *Pyrococcus abyssi*, *Archaeoglobus fulgidus* and *Haloferax volcanii* (Fig. 7 & Fig. S18).

```
# STOCKHOLM 1.0

Hvo.1                         UGCGUAC.CU......CAAGUC................C.C.C...G.....GC...........CGA....GUGUU.C...CC...GCUUC.....GGGAGC...GAUGACAGCA.CG.GCGAACCC....G.G.GUGCGCA..AUA
Hvo.2a                        CGCCCGGCAC.....GAGGGUU................U.C.C...C....GGU...........CGA....CG..C.G.G.C...ACGCC......GCCUCG..GGAUGA.GACC.GG.C.CGUUAGU...G.UUCUGGGCG..ACA
Hvo.2b                        CGCCCGG.CA.....CGAGGGU................U.UCC...C....GGU...........CGA....CG..C.G.G.C...ACGCC......GCCUCG..GGAUGA.GACC.GG.CCGUUAGUG...U.U.CUGGGCG..ACA
Af4.1                         GUCCCCG.AU.......CGGGG................A.A.G...A....GGC...........AGA....GG..U.G...GC.....AGU.....GCCGAC...GAUGA.AGCU.UC.U.C.UGCGAG..C.U.CGGGGAC..ACA
Af4.2a                        ACCAUGU.CG.......AUAGG................UCC.C...C....UUA...........GGA....GU..G.A...GGC...GAAA....GCCUUAC...GAUGA.GUAU.GG.G.G.CAACC...CGG.ACAUGGG..ACA
Af4.2b                        ACCAUGU.CG......AUAGGU................C.C.C...C....UUA...........GGA....GU..G.A...GGC...GAAA....GCCUUAC...GAUGA.GUAU.G..G.G.GCAACC..CGG.ACAUGGG..ACA
Af4.3                         GCCCCCA.GA.......GGUCA................G.C.C...A....CUC...........UGA....GA..G.G...GC.....AAA.....GCCUGC...GAUGA.GGGG.UG.G.U.GUUAC...U.C.UGGGGGC..ACA
Af46                          AGCUCCG.CC.....CCCUCAC................G.C.C...C....GGG...........UGA....................GAAGCA..............UGA.UCCC.GG.G.U.CGGUU...G.G.CGGAGCU..ACA
Af190                         UGCCCGC.CA......GCCAUG................C.G.U...U....CCC...........UGA....................UUGG................UGA.GGGG.AA.U.U.CCACU...U.G.GCGGGCU..ACA
Pa19-S1017                    GCCCAGG.GGUCAAGACGGCGGC...............G.U.C...G....GGG...........GGA....UU..G.G...GGGC...AAA...GCCCCCGG...CAUGA.ACCC.CG...C.CCUCCU..C.C.CCUGGGU..AUA
Pa21-S891                     GGGCCCG.GC.....UCCCGCC................CUC.U...C....CGG...........GGA...................AUC.................GUGA.ACCG.GG.G.G.UUCCG...G.C.CGGGCCU..ACA
Pa35.1-L2930                  GGGCUCG.GU......CUACCC................G.C.C...C....CCG...........CAA....GG............UGUUCGGGU......UC...GAUGA.GCGG.GGUG.U.GCUCAC..G.C.CGAGCCC..ACA
Pa35.1-L2672                  GGGCUCG.GU......CUACCC................G.C.C...C....CCG...........CAA....GG............UGUUCGGGU......UC...GAUGA.GCGG.GGUG.U.GCUCAC..G.C.CGAGCCC..ACA
Pa35.2-L2549                  GGGCCGG.UG.......CAUCC................G.C.C...C....GCG...........GGA...................UCA.................AUGA.CCGCCGG.G.U.CUCUGU..U.G.CCGGCCU..ACA
Pa35.2-L2697                  GGGCCGG.UG.......CAUCC................G.C.C...C....GCG...........GGA...................UCA.................AUGA.CCGCCGG.GUC..UCUGU..U.G.CCGGCCU..ACA
Pa35.2-L2250                  GGGCCGG.UG.......CAUCC................G.C.C...C....GCG...........GGA...................UCA.................AUGA.CCGCCGG.GUC.UCUGU...U.G.CCGGCCU..ACA
Pa40.1-L2588                  UGCCCCC.GC....AAGCGAGG................G.C.C...U....GGU...........CGA...................UUA.................GUGA.GACC.AG.G.U.GCGAC...G.C.GGGGGCU..ACA
Pa40.2-L1932                  GCCCGGC.CU.CAGCGAGGUCCCCUCGGUAGGUG....C.C.U...U....CCG.CGUCAC....GGA..................GCGCCG................UGA.CCGG.GG.G.U.AACCC...U.G.GCCGGGC..ACA
Pa40.3-L2016                  GGCCCGU.CU......GGGUUA................G.C.C...C....GCC...........UGA....UC..AUG...C...CGUUG.......GCUUA...GAUGA.AGGC.GG.G.U.GUUAC...G.G.GCGGGCU..ACA
Pa91-L2685                    CCCCUCC.CC......UCUCAC................A.C.C...U....CCG...........GGA...................UCA.................GUGA.CCGG.AG.G.G.CGGUC...G.G.GGAGGGG..ACA
Pa105.1-L2554                 CCGCCCG.GA.......GGCCC................GA..C...C....GAG...........GGA....GC..G.U...GCC.GAGAAA....GGCGCGC...CAUGA.ACGA.GG...C.GACGUC..G.C.CGGGCGG..ACA
Pa105.2-S995                  GGGCCCG.GU......CUCCGG................G.G.C...C....GCC...........UGA....GG..U.U...GCC..GACAAC...GGCGGGC...AAUGA.GGGC.GG.G.U.GGAUAA..G.C.CGGGCCU..AUA
Pa105.2-L2794                 GGGCCCG.GU......CUCCGG................G.G.C...C....GCC...........UGA....GG..U.U...GCC..GACAAC...GGCGGGC...AAUGA.GGGC.GG.G.U.GGAUAA..G.C.CGGGCCU..AUA
Pa105.2-L1377                 GGGCCCG.GU......CUCCGG................G.G.C...C....GCC...........UGA....GG..U.U...GCC..GACAAC...GGCGGGC...AAUGA.GGGC.GG.G.U.GGAUA...AGC.CGGGCCU..AUA
Pa105.2-S27                   GGGCCCG.GU......CUCCGG................G.G.C...C....GCC...........UGA....GG..U.U...GCC..GACAAC...GGCGGGC...AAUGA.GGGC.GG.G.U.GGAUA...AGC.CGGGCCU..AUA
Pa160-S922                    GCCCCCG.GA.......AACCG................C.G.G...G....GGA...........GGA....GC..U.U.A.GGC....UUAA...GCCGAGC...UAUGA.CUCC.CC.U.U.CGCUC...C.C.CGGGGGC..ACA
#=GC SS_cons                  <<<<<<<.<<............................<-<.<...<....<<<...........<<<....<<..<.<...<<<<_________>>>>>>>>...-..>>.>>>>->>->.>.......-.>->.>>>>>>>.....
#=GC R2R_LABEL                q.........1.........................2..3.d.............4......5...II.6...pab....................p......7.....HHfHHiHgHHBHcH8.......0H9HeHHhHHHr.....
#=GC R2R_XLABEL_KTURN         ..................................................................TTTTTTTT...........................RRRRRRRRRR.....................................
#=GC R2R_XLABEL_KTURNL        ...........................................................................................................r........................................
#=GC R2R_XLABEL_KLOOP         ..................................................................KKKKKKKKKKKKKKKKKKKKKKKKKKKKKKKKKKKKKKKKKKKKKK.....................................
#=GC R2R_XLABEL_KLOOPL        ........................................................................................k...........................................................
#=GC R2R_XLABEL_ILOOP         ..........PPPPPPPPPPPPPPPPPPPQXXXXXXXW.....................................................................................YYYYYYSUT.V...........ZZZ
#=GC R2R_XLABEL_ILOOPL        ....................x................w.........................................................................................y.sut.v.............z
#=GC R2R_XLABEL_STEM          ....................................................................................................................................................
#=GC R2R_XLABEL_STEML         ....................................................................................................................................................

#=GF R2R keep allpairs
#=GF R2R var_backbone_range_size_fake_nucs 10 1 2
#=GF R2R var_backbone_range_size_fake_nucs 2 a b
#=GF R2R var_backbone_range_size_fake_nucs 1 d d
#=GF R2R var_backbone_range_size_fake_nucs 1 e e
#=GF R2R var_backbone_range_size_fake_nucs 1 f f
#=GF R2R var_backbone_range_size_fake_nucs 8 8 0
#=GF R2R var_backbone_range 4 5
#=GF R2R var_backbone_range 6 7
#=GF R2R var_backbone_range_size_fake_nucs 1 3 3
#=GF R2R var_backbone_range_size_fake_nucs 1 9 9
#=GF R2R var_backbone_range_size_fake_nucs 1 c c
#=GF R2R var_backbone_range_size_fake_nucs 1 B B
#=GF R2R shade_along_backbone ILOOP:P rgb:0,255,0
#=GF R2R shade_along_backbone ILOOP:Q rgb:0,255,0
#=GF R2R shade_along_backbone ILOOP:X rgb:0,255,0
#=GF R2R shade_along_backbone ILOOP:Y rgb:0,255,0
#=GF R2R shade_along_backbone ILOOP:S rgb:0,255,0
#=GF R2R shade_along_backbone ILOOP:U rgb:0,255,0
#=GF R2R outline_nuc H
#=GF R2R outline_nuc h
#=GF R2R outline_nuc r
#=GF R2R outline_nuc i
#=GF R2R tick_label h 9 bp
#=GF R2R tick_label i 10 bp

#=GF R2R_oneseq Pa19-S1017 shade_along_backbone KTURN:T rgb:0,129,255
#=GF R2R_oneseq Pa19-S1017 shade_along_backbone KTURN:R rgb:0,129,255
#=GF R2R_oneseq Pa19-S1017 tick_label KTURNL:r K-turn
#=GF R2R_oneseq Pa19-S1017 shade_along_backbone ILOOP:Y rgb:0,255,0
#=GF R2R_oneseq Pa19-S1017 shade_along_backbone ILOOP:S rgb:0,255,0
#=GF R2R_oneseq Pa19-S1017 shade_along_backbone ILOOP:U rgb:0,255,0
#=GF R2R_oneseq Pa19-S1017 shade_along_backbone ILOOP:P rgb:0,255,0
#=GF R2R_oneseq Pa19-S1017 shade_along_backbone ILOOP:Q rgb:0,255,0
#=GF R2R_oneseq Pa19-S1017 shade_along_backbone ILOOP:X rgb:0,255,0
#=GF R2R_oneseq Pa19-S1017 shade_along_backbone ILOOP:Z rgb:200,200,200
#=GF R2R_oneseq Pa19-S1017 tick_label ILOOPL:z ANA box

#=GF R2R_oneseq Pa35.1-S1122 shade_along_backbone KTURN:T rgb:0,129,255
#=GF R2R_oneseq Pa35.1-S1122 shade_along_backbone KTURN:R rgb:0,129,255
#=GF R2R_oneseq Pa35.1-S1122 tick_label KTURNL:r K-turn
#=GF R2R_oneseq Pa35.1-S1122 shade_along_backbone ILOOP:Y rgb:0,255,0
#=GF R2R_oneseq Pa35.1-S1122 shade_along_backbone ILOOP:S rgb:0,255,0
#=GF R2R_oneseq Pa35.1-S1122 shade_along_backbone ILOOP:U rgb:0,255,0
#=GF R2R_oneseq Pa35.1-S1122 shade_along_backbone ILOOP:T rgb:0,255,0
#=GF R2R_oneseq Pa35.1-S1122 shade_along_backbone ILOOP:P rgb:0,255,0
#=GF R2R_oneseq Pa35.1-S1122 shade_along_backbone ILOOP:Q rgb:0,255,0
#=GF R2R_oneseq Pa35.1-S1122 shade_along_backbone ILOOP:X rgb:0,255,0
#=GF R2R_oneseq Pa35.1-S1122 shade_along_backbone ILOOP:Z rgb:200,200,200
#=GF R2R_oneseq Pa35.1-S1122 tick_label ILOOPL:z ANA box

#=GF R2R_oneseq Pa21-S891 shade_along_backbone KLOOP:K rgb:0,129,255
#=GF R2R_oneseq Pa21-S891 tick_label KLOOPL:k K-loop
#=GF R2R_oneseq Pa21-S891 shade_along_backbone ILOOP:Y rgb:0,255,0
#=GF R2R_oneseq Pa21-S891 shade_along_backbone ILOOP:S rgb:0,255,0
#=GF R2R_oneseq Pa21-S891 shade_along_backbone ILOOP:U rgb:0,255,0
#=GF R2R_oneseq Pa21-S891 shade_along_backbone ILOOP:P rgb:0,255,0
#=GF R2R_oneseq Pa21-S891 shade_along_backbone ILOOP:Q rgb:0,255,0
#=GF R2R_oneseq Pa21-S891 shade_along_backbone ILOOP:X rgb:0,255,0
#=GF R2R_oneseq Pa21-S891 shade_along_backbone ILOOP:Z rgb:200,200,200
#=GF R2R_oneseq Pa21-S891 tick_label ILOOPL:z ANA box

#=GF R2R_oneseq Pa35.1-L2930 shade_along_backbone KTURN:T rgb:0,129,255
#=GF R2R_oneseq Pa35.1-L2930 shade_along_backbone KTURN:R rgb:0,129,255
#=GF R2R_oneseq Pa35.1-L2930 tick_label KTURNL:r K-turn
#=GF R2R_oneseq Pa35.1-L2930 shade_along_backbone ILOOP:Y rgb:0,255,0
#=GF R2R_oneseq Pa35.1-L2930 shade_along_backbone ILOOP:S rgb:0,255,0
#=GF R2R_oneseq Pa35.1-L2930 shade_along_backbone ILOOP:U rgb:0,255,0
```

```
#=GF R2R_oneseq Pa35.1-L2930 shade_along_backbone ILOOP:P rgb:0,255,0
#=GF R2R_oneseq Pa35.1-L2930 shade_along_backbone ILOOP:Q rgb:0,255,0
#=GF R2R_oneseq Pa35.1-L2930 shade_along_backbone ILOOP:X rgb:0,255,0
#=GF R2R_oneseq Pa35.1-L2930 shade_along_backbone ILOOP:Z rgb:200,200,200
#=GF R2R_oneseq Pa35.1-L2930 tick_label ILOOPL:z ANA box

#=GF R2R_oneseq Pa35.1-L2672 shade_along_backbone KTURN:T rgb:0,129,255
#=GF R2R_oneseq Pa35.1-L2672 shade_along_backbone KTURN:R rgb:0,129,255
#=GF R2R_oneseq Pa35.1-L2672 tick_label KTURNL:r K-turn
#=GF R2R_oneseq Pa35.1-L2672 shade_along_backbone ILOOP:Y rgb:0,255,0
#=GF R2R_oneseq Pa35.1-L2672 shade_along_backbone ILOOP:S rgb:0,255,0
#=GF R2R_oneseq Pa35.1-L2672 shade_along_backbone ILOOP:U rgb:0,255,0
#=GF R2R_oneseq Pa35.1-L2672 shade_along_backbone ILOOP:P rgb:0,255,0
#=GF R2R_oneseq Pa35.1-L2672 shade_along_backbone ILOOP:Q rgb:0,255,0
#=GF R2R_oneseq Pa35.1-L2672 shade_along_backbone ILOOP:X rgb:0,255,0
#=GF R2R_oneseq Pa35.1-L2672 shade_along_backbone ILOOP:Z rgb:200,200,200
#=GF R2R_oneseq Pa35.1-L2672 tick_label ILOOPL:z ANA box

#=GF R2R_oneseq Pa35.2-L2549 shade_along_backbone KLOOP:K rgb:0,129,255
#=GF R2R_oneseq Pa35.2-L2549 tick_label KLOOPL:k K-loop
#=GF R2R_oneseq Pa35.2-L2549 shade_along_backbone ILOOP:Y rgb:0,255,0
#=GF R2R_oneseq Pa35.2-L2549 shade_along_backbone ILOOP:S rgb:0,255,0
#=GF R2R_oneseq Pa35.2-L2549 shade_along_backbone ILOOP:U rgb:0,255,0
#=GF R2R_oneseq Pa35.2-L2549 shade_along_backbone ILOOP:T rgb:0,255,0
#=GF R2R_oneseq Pa35.2-L2549 shade_along_backbone ILOOP:P rgb:0,255,0
#=GF R2R_oneseq Pa35.2-L2549 shade_along_backbone ILOOP:Q rgb:0,255,0
#=GF R2R_oneseq Pa35.2-L2549 shade_along_backbone ILOOP:X rgb:0,255,0
#=GF R2R_oneseq Pa35.2-L2549 shade_along_backbone ILOOP:Z rgb:200,200,200
#=GF R2R_oneseq Pa35.2-L2549 tick_label ILOOPL:z ANA box

#=GF R2R_oneseq Pa35.2-L2697 shade_along_backbone KLOOP:K rgb:0,129,255
#=GF R2R_oneseq Pa35.2-L2697 tick_label KLOOPL:k K-loop
#=GF R2R_oneseq Pa35.2-L2697 shade_along_backbone ILOOP:Y rgb:0,255,0
#=GF R2R_oneseq Pa35.2-L2697 shade_along_backbone ILOOP:S rgb:0,255,0
#=GF R2R_oneseq Pa35.2-L2697 shade_along_backbone ILOOP:U rgb:0,255,0
#=GF R2R_oneseq Pa35.2-L2697 shade_along_backbone ILOOP:T rgb:0,255,0
#=GF R2R_oneseq Pa35.2-L2697 shade_along_backbone ILOOP:P rgb:0,255,0
#=GF R2R_oneseq Pa35.2-L2697 shade_along_backbone ILOOP:Q rgb:0,255,0
#=GF R2R_oneseq Pa35.2-L2697 shade_along_backbone ILOOP:X rgb:0,255,0
#=GF R2R_oneseq Pa35.2-L2697 shade_along_backbone ILOOP:Z rgb:200,200,200
#=GF R2R_oneseq Pa35.2-L2697 tick_label ILOOPL:z ANA box

#=GF R2R_oneseq Pa35.2-L2250 shade_along_backbone KLOOP:K rgb:0,129,255
#=GF R2R_oneseq Pa35.2-L2250 tick_label KLOOPL:k K-loop
#=GF R2R_oneseq Pa35.2-L2250 shade_along_backbone ILOOP:Y rgb:0,255,0
#=GF R2R_oneseq Pa35.2-L2250 shade_along_backbone ILOOP:S rgb:0,255,0
#=GF R2R_oneseq Pa35.2-L2250 shade_along_backbone ILOOP:U rgb:0,255,0
#=GF R2R_oneseq Pa35.2-L2250 shade_along_backbone ILOOP:T rgb:0,255,0
#=GF R2R_oneseq Pa35.2-L2250 shade_along_backbone ILOOP:P rgb:0,255,0
#=GF R2R_oneseq Pa35.2-L2250 shade_along_backbone ILOOP:Q rgb:0,255,0
#=GF R2R_oneseq Pa35.2-L2250 shade_along_backbone ILOOP:X rgb:0,255,0
#=GF R2R_oneseq Pa35.2-L2250 shade_along_backbone ILOOP:Z rgb:200,200,200
#=GF R2R_oneseq Pa35.2-L2250 tick_label ILOOPL:z ANA box

#=GF R2R_oneseq Pa40.1-L2588 shade_along_backbone KLOOP:K rgb:0,129,255
#=GF R2R_oneseq Pa40.1-L2588 tick_label KLOOPL:k K-loop
#=GF R2R_oneseq Pa40.1-L2588 shade_along_backbone ILOOP:Y rgb:0,255,0
#=GF R2R_oneseq Pa40.1-L2588 shade_along_backbone ILOOP:S rgb:0,255,0
#=GF R2R_oneseq Pa40.1-L2588 shade_along_backbone ILOOP:U rgb:0,255,0
#=GF R2R_oneseq Pa40.1-L2588 shade_along_backbone ILOOP:T rgb:0,255,0
#=GF R2R_oneseq Pa40.1-L2588 shade_along_backbone ILOOP:P rgb:0,255,0
#=GF R2R_oneseq Pa40.1-L2588 shade_along_backbone ILOOP:Q rgb:0,255,0
#=GF R2R_oneseq Pa40.1-L2588 shade_along_backbone ILOOP:X rgb:0,255,0
#=GF R2R_oneseq Pa40.1-L2588 shade_along_backbone ILOOP:Z rgb:200,200,200
#=GF R2R_oneseq Pa40.1-L2588 tick_label ILOOPL:z ANA box

#=GF R2R_oneseq Pa40.2-L1932 shade_along_backbone KLOOP:K rgb:0,129,255
#=GF R2R_oneseq Pa40.2-L1932 tick_label KLOOPL:k K-loop
#=GF R2R_oneseq Pa40.2-L1932 shade_along_backbone ILOOP:Y rgb:0,255,0
#=GF R2R_oneseq Pa40.2-L1932 shade_along_backbone ILOOP:S rgb:0,255,0
#=GF R2R_oneseq Pa40.2-L1932 shade_along_backbone ILOOP:U rgb:0,255,0
#=GF R2R_oneseq Pa40.2-L1932 shade_along_backbone ILOOP:P rgb:0,255,0
#=GF R2R_oneseq Pa40.2-L1932 shade_along_backbone ILOOP:Q rgb:0,255,0
#=GF R2R_oneseq Pa40.2-L1932 shade_along_backbone ILOOP:X rgb:0,255,0
#=GF R2R_oneseq Pa40.2-L1932 shade_along_backbone ILOOP:Z rgb:200,200,200
#=GF R2R_oneseq Pa40.2-L1932 tick_label ILOOPL:z ANA box

#=GF R2R_oneseq Pa40.3-L2016 shade_along_backbone KTURN:T rgb:0,129,255
#=GF R2R_oneseq Pa40.3-L2016 shade_along_backbone KTURN:R rgb:0,129,255
#=GF R2R_oneseq Pa40.3-L2016 tick_label KTURNL:r K-turn
#=GF R2R_oneseq Pa40.3-L2016 shade_along_backbone ILOOP:Y rgb:0,255,0
#=GF R2R_oneseq Pa40.3-L2016 shade_along_backbone ILOOP:S rgb:0,255,0
#=GF R2R_oneseq Pa40.3-L2016 shade_along_backbone ILOOP:U rgb:0,255,0
#=GF R2R_oneseq Pa40.3-L2016 shade_along_backbone ILOOP:T rgb:0,255,0
#=GF R2R_oneseq Pa40.3-L2016 shade_along_backbone ILOOP:P rgb:0,255,0
#=GF R2R_oneseq Pa40.3-L2016 shade_along_backbone ILOOP:Q rgb:0,255,0
#=GF R2R_oneseq Pa40.3-L2016 shade_along_backbone ILOOP:X rgb:0,255,0
#=GF R2R_oneseq Pa40.3-L2016 shade_along_backbone ILOOP:Z rgb:200,200,200
#=GF R2R_oneseq Pa40.3-L2016 tick_label ILOOPL:z ANA box

#=GF R2R_oneseq Pa91-L2685 shade_along_backbone KLOOP:K rgb:0,129,255
#=GF R2R_oneseq Pa91-L2685 tick_label KLOOPL:k K-loop
#=GF R2R_oneseq Pa91-L2685 shade_along_backbone ILOOP:Y rgb:0,255,0
#=GF R2R_oneseq Pa91-L2685 shade_along_backbone ILOOP:S rgb:0,255,0
#=GF R2R_oneseq Pa91-L2685 shade_along_backbone ILOOP:U rgb:0,255,0
#=GF R2R_oneseq Pa91-L2685 shade_along_backbone ILOOP:T rgb:0,255,0
#=GF R2R_oneseq Pa91-L2685 shade_along_backbone ILOOP:P rgb:0,255,0
#=GF R2R_oneseq Pa91-L2685 shade_along_backbone ILOOP:Q rgb:0,255,0
#=GF R2R_oneseq Pa91-L2685 shade_along_backbone ILOOP:X rgb:0,255,0
#=GF R2R_oneseq Pa91-L2685 shade_along_backbone ILOOP:Z rgb:200,200,200
#=GF R2R_oneseq Pa91-L2685 tick_label ILOOPL:z ANA box

#=GF R2R_oneseq Pa105.1-L2554 shade_along_backbone KTURN:T rgb:0,129,255
#=GF R2R_oneseq Pa105.1-L2554 shade_along_backbone KTURN:R rgb:0,129,255
#=GF R2R_oneseq Pa105.1-L2554 tick_label KTURNL:r K-turn
#=GF R2R_oneseq Pa105.1-L2554 shade_along_backbone ILOOP:Y rgb:0,255,0
#=GF R2R_oneseq Pa105.1-L2554 shade_along_backbone ILOOP:S rgb:0,255,0
#=GF R2R_oneseq Pa105.1-L2554 shade_along_backbone ILOOP:U rgb:0,255,0
#=GF R2R_oneseq Pa105.1-L2554 shade_along_backbone ILOOP:T rgb:0,255,0
#=GF R2R_oneseq Pa105.1-L2554 shade_along_backbone ILOOP:P rgb:0,255,0
#=GF R2R_oneseq Pa105.1-L2554 shade_along_backbone ILOOP:Q rgb:0,255,0
```

```
#=GF R2R_oneseq Pa105.1-L2554 shade_along_backbone ILOOP:X rgb:0,255,0
#=GF R2R_oneseq Pa105.1-L2554 shade_along_backbone ILOOP:Z rgb:200,200,200
#=GF R2R_oneseq Pa105.1-L2554 tick_label ILOOPL:z ANA box

#=GF R2R_oneseq Pa105.2-S995 shade_along_backbone KTURN:T rgb:0,129,255
#=GF R2R_oneseq Pa105.2-S995 shade_along_backbone KTURN:R rgb:0,129,255
#=GF R2R_oneseq Pa105.2-S995 tick_label KTURNL:r K-turn
#=GF R2R_oneseq Pa105.2-S995 shade_along_backbone ILOOP:Y rgb:0,255,0
#=GF R2R_oneseq Pa105.2-S995 shade_along_backbone ILOOP:S rgb:0,255,0
#=GF R2R_oneseq Pa105.2-S995 shade_along_backbone ILOOP:U rgb:0,255,0
#=GF R2R_oneseq Pa105.2-S995 shade_along_backbone ILOOP:T rgb:0,255,0
#=GF R2R_oneseq Pa105.2-S995 shade_along_backbone ILOOP:P rgb:0,255,0
#=GF R2R_oneseq Pa105.2-S995 shade_along_backbone ILOOP:Q rgb:0,255,0
#=GF R2R_oneseq Pa105.2-S995 shade_along_backbone ILOOP:X rgb:0,255,0
#=GF R2R_oneseq Pa105.2-S995 shade_along_backbone ILOOP:Z rgb:200,200,200
#=GF R2R_oneseq Pa105.2-S995 tick_label ILOOPL:z ANA box

#=GF R2R_oneseq Pa105.2-L2794 shade_along_backbone KTURN:T rgb:0,129,255
#=GF R2R_oneseq Pa105.2-L2794 shade_along_backbone KTURN:R rgb:0,129,255
#=GF R2R_oneseq Pa105.2-L2794 tick_label KTURNL:r K-turn
#=GF R2R_oneseq Pa105.2-L2794 shade_along_backbone ILOOP:Y rgb:0,255,0
#=GF R2R_oneseq Pa105.2-L2794 shade_along_backbone ILOOP:S rgb:0,255,0
#=GF R2R_oneseq Pa105.2-L2794 shade_along_backbone ILOOP:U rgb:0,255,0
#=GF R2R_oneseq Pa105.2-L2794 shade_along_backbone ILOOP:T rgb:0,255,0
#=GF R2R_oneseq Pa105.2-L2794 shade_along_backbone ILOOP:P rgb:0,255,0
#=GF R2R_oneseq Pa105.2-L2794 shade_along_backbone ILOOP:Q rgb:0,255,0
#=GF R2R_oneseq Pa105.2-L2794 shade_along_backbone ILOOP:X rgb:0,255,0
#=GF R2R_oneseq Pa105.2-L2794 shade_along_backbone ILOOP:Z rgb:200,200,200
#=GF R2R_oneseq Pa105.2-L2794 tick_label ILOOPL:z ANA box

#=GF R2R_oneseq Pa105.2-L1377 shade_along_backbone KTURN:T rgb:0,129,255
#=GF R2R_oneseq Pa105.2-L1377 shade_along_backbone KTURN:R rgb:0,129,255
#=GF R2R_oneseq Pa105.2-L1377 tick_label KTURNL:r K-turn
#=GF R2R_oneseq Pa105.2-L1377 shade_along_backbone ILOOP:Y rgb:0,255,0
#=GF R2R_oneseq Pa105.2-L1377 shade_along_backbone ILOOP:S rgb:0,255,0
#=GF R2R_oneseq Pa105.2-L1377 shade_along_backbone ILOOP:U rgb:0,255,0
#=GF R2R_oneseq Pa105.2-L1377 shade_along_backbone ILOOP:T rgb:0,255,0
#=GF R2R_oneseq Pa105.2-L1377 shade_along_backbone ILOOP:P rgb:0,255,0
#=GF R2R_oneseq Pa105.2-L1377 shade_along_backbone ILOOP:Q rgb:0,255,0
#=GF R2R_oneseq Pa105.2-L1377 shade_along_backbone ILOOP:X rgb:0,255,0
#=GF R2R_oneseq Pa105.2-L1377 shade_along_backbone ILOOP:Z rgb:200,200,200
#=GF R2R_oneseq Pa105.2-L1377 tick_label ILOOPL:z ANA box

#=GF R2R_oneseq Pa105.2-S27 shade_along_backbone KTURN:T rgb:0,129,255
#=GF R2R_oneseq Pa105.2-S27 shade_along_backbone KTURN:R rgb:0,129,255
#=GF R2R_oneseq Pa105.2-S27 tick_label KTURNL:r K-turn
#=GF R2R_oneseq Pa105.2-S27 shade_along_backbone ILOOP:Y rgb:0,255,0
#=GF R2R_oneseq Pa105.2-S27 shade_along_backbone ILOOP:S rgb:0,255,0
#=GF R2R_oneseq Pa105.2-S27 shade_along_backbone ILOOP:U rgb:0,255,0
#=GF R2R_oneseq Pa105.2-S27 shade_along_backbone ILOOP:T rgb:0,255,0
#=GF R2R_oneseq Pa105.2-S27 shade_along_backbone ILOOP:P rgb:0,255,0
#=GF R2R_oneseq Pa105.2-S27 shade_along_backbone ILOOP:Q rgb:0,255,0
#=GF R2R_oneseq Pa105.2-S27 shade_along_backbone ILOOP:X rgb:0,255,0
#=GF R2R_oneseq Pa105.2-S27 shade_along_backbone ILOOP:Z rgb:200,200,200
#=GF R2R_oneseq Pa105.2-S27 tick_label ILOOPL:z ANA box

#=GF R2R_oneseq Pa160-S922 shade_along_backbone KTURN:T rgb:0,129,255
#=GF R2R_oneseq Pa160-S922 shade_along_backbone KTURN:R rgb:0,129,255
#=GF R2R_oneseq Pa160-S922 tick_label KTURNL:r K-turn
#=GF R2R_oneseq Pa160-S922 shade_along_backbone ILOOP:Y rgb:0,255,0
#=GF R2R_oneseq Pa160-S922 shade_along_backbone ILOOP:S rgb:0,255,0
#=GF R2R_oneseq Pa160-S922 shade_along_backbone ILOOP:U rgb:0,255,0
#=GF R2R_oneseq Pa160-S922 shade_along_backbone ILOOP:T rgb:0,255,0
#=GF R2R_oneseq Pa160-S922 shade_along_backbone ILOOP:P rgb:0,255,0
#=GF R2R_oneseq Pa160-S922 shade_along_backbone ILOOP:Q rgb:0,255,0
#=GF R2R_oneseq Pa160-S922 shade_along_backbone ILOOP:X rgb:0,255,0
#=GF R2R_oneseq Pa160-S922 shade_along_backbone ILOOP:Z rgb:200,200,200
#=GF R2R_oneseq Pa160-S922 tick_label ILOOPL:z ANA box

#=GF R2R_oneseq Af4.1 shade_along_backbone KTURN:T rgb:0,129,255
#=GF R2R_oneseq Af4.1 shade_along_backbone KTURN:R rgb:0,129,255
#=GF R2R_oneseq Af4.1 tick_label KTURNL:r K-turn
#=GF R2R_oneseq Af4.1 shade_along_backbone ILOOP:Y rgb:0,255,0
#=GF R2R_oneseq Af4.1 shade_along_backbone ILOOP:S rgb:0,255,0
#=GF R2R_oneseq Af4.1 shade_along_backbone ILOOP:U rgb:0,255,0
#=GF R2R_oneseq Af4.1 shade_along_backbone ILOOP:P rgb:0,255,0
#=GF R2R_oneseq Af4.1 shade_along_backbone ILOOP:Q rgb:0,255,0
#=GF R2R_oneseq Af4.1 shade_along_backbone ILOOP:X rgb:0,255,0
#=GF R2R_oneseq Af4.1 shade_along_backbone ILOOP:Z rgb:200,200,200
#=GF R2R_oneseq Af4.1 tick_label ILOOPL:z ANA box

#=GF R2R_oneseq Af4.2a shade_along_backbone KTURN:T rgb:0,129,255
#=GF R2R_oneseq Af4.2a shade_along_backbone KTURN:R rgb:0,129,255
#=GF R2R_oneseq Af4.2a tick_label KTURNL:r K-turn
#=GF R2R_oneseq Af4.2a shade_along_backbone ILOOP:Y rgb:0,255,0
#=GF R2R_oneseq Af4.2a shade_along_backbone ILOOP:S rgb:0,255,0
#=GF R2R_oneseq Af4.2a shade_along_backbone ILOOP:U rgb:0,255,0
#=GF R2R_oneseq Af4.2a shade_along_backbone ILOOP:P rgb:0,255,0
#=GF R2R_oneseq Af4.2a shade_along_backbone ILOOP:Q rgb:0,255,0
#=GF R2R_oneseq Af4.2a shade_along_backbone ILOOP:X rgb:0,255,0
#=GF R2R_oneseq Af4.2a shade_along_backbone ILOOP:Z rgb:200,200,200
#=GF R2R_oneseq Af4.2a tick_label ILOOPL:z ANA box

#=GF R2R_oneseq Af4.2b shade_along_backbone KTURN:T rgb:0,129,255
#=GF R2R_oneseq Af4.2b shade_along_backbone KTURN:R rgb:0,129,255
#=GF R2R_oneseq Af4.2b tick_label KTURNL:r K-turn
#=GF R2R_oneseq Af4.2b shade_along_backbone ILOOP:Y rgb:0,255,0
#=GF R2R_oneseq Af4.2b shade_along_backbone ILOOP:S rgb:0,255,0
#=GF R2R_oneseq Af4.2b shade_along_backbone ILOOP:U rgb:0,255,0
#=GF R2R_oneseq Af4.2b shade_along_backbone ILOOP:P rgb:0,255,0
#=GF R2R_oneseq Af4.2b shade_along_backbone ILOOP:Q rgb:0,255,0
#=GF R2R_oneseq Af4.2b shade_along_backbone ILOOP:X rgb:0,255,0
#=GF R2R_oneseq Af4.2b shade_along_backbone ILOOP:Z rgb:200,200,200
#=GF R2R_oneseq Af4.2b tick_label ILOOPL:z ANA box

#=GF R2R_oneseq Af4.3 shade_along_backbone KTURN:T rgb:0,129,255
#=GF R2R_oneseq Af4.3 shade_along_backbone KTURN:R rgb:0,129,255
#=GF R2R_oneseq Af4.3 tick_label KTURNL:r K-turn
#=GF R2R_oneseq Af4.3 shade_along_backbone ILOOP:Y rgb:0,255,0
#=GF R2R_oneseq Af4.3 shade_along_backbone ILOOP:S rgb:0,255,0
#=GF R2R_oneseq Af4.3 shade_along_backbone ILOOP:U rgb:0,255,0
```

```
#=GF R2R_oneseq Af4.3 shade_along_backbone ILOOP:P rgb:0,255,0
#=GF R2R_oneseq Af4.3 shade_along_backbone ILOOP:Q rgb:0,255,0
#=GF R2R_oneseq Af4.3 shade_along_backbone ILOOP:X rgb:0,255,0
#=GF R2R_oneseq Af4.3 shade_along_backbone ILOOP:Z rgb:200,200,200
#=GF R2R_oneseq Af4.3 tick_label ILOOPL:z ANA box

#=GF R2R_oneseq Af46 shade_along_backbone KLOOP:K rgb:0,129,255
#=GF R2R_oneseq Af46 tick_label KLOOPL:k K-loop
#=GF R2R_oneseq Af46 shade_along_backbone ILOOP:Y rgb:0,255,0
#=GF R2R_oneseq Af46 shade_along_backbone ILOOP:S rgb:0,255,0
#=GF R2R_oneseq Af46 shade_along_backbone ILOOP:U rgb:0,255,0
#=GF R2R_oneseq Af46 shade_along_backbone ILOOP:P rgb:0,255,0
#=GF R2R_oneseq Af46 shade_along_backbone ILOOP:Q rgb:0,255,0
#=GF R2R_oneseq Af46 shade_along_backbone ILOOP:X rgb:0,255,0
#=GF R2R_oneseq Af46 shade_along_backbone ILOOP:Z rgb:200,200,200
#=GF R2R_oneseq Af46 tick_label ILOOPL:z ANA box

#=GF R2R_oneseq Af190 shade_along_backbone KLOOP:K rgb:0,129,255
#=GF R2R_oneseq Af190 tick_label KLOOPL:k K-loop
#=GF R2R_oneseq Af190 shade_along_backbone ILOOP:Y rgb:0,255,0
#=GF R2R_oneseq Af190 shade_along_backbone ILOOP:S rgb:0,255,0
#=GF R2R_oneseq Af190 shade_along_backbone ILOOP:U rgb:0,255,0
#=GF R2R_oneseq Af190 shade_along_backbone ILOOP:P rgb:0,255,0
#=GF R2R_oneseq Af190 shade_along_backbone ILOOP:Q rgb:0,255,0
#=GF R2R_oneseq Af190 shade_along_backbone ILOOP:X rgb:0,255,0
#=GF R2R_oneseq Af190 shade_along_backbone ILOOP:Z rgb:200,200,200
#=GF R2R_oneseq Af190 tick_label ILOOPL:z ANA box

#=GF R2R_oneseq Hvo.1 shade_along_backbone KTURN:T rgb:0,129,255
#=GF R2R_oneseq Hvo.1 shade_along_backbone KTURN:R rgb:0,129,255
#=GF R2R_oneseq Hvo.1 tick_label KTURNL:r K-turn
#=GF R2R_oneseq Hvo.1 shade_along_backbone ILOOP:Y rgb:0,255,0
#=GF R2R_oneseq Hvo.1 shade_along_backbone ILOOP:S rgb:0,255,0
#=GF R2R_oneseq Hvo.1 shade_along_backbone ILOOP:U rgb:0,255,0
#=GF R2R_oneseq Hvo.1 shade_along_backbone ILOOP:P rgb:0,255,0
#=GF R2R_oneseq Hvo.1 shade_along_backbone ILOOP:Q rgb:0,255,0
#=GF R2R_oneseq Hvo.1 shade_along_backbone ILOOP:X rgb:0,255,0
#=GF R2R_oneseq Hvo.1 shade_along_backbone ILOOP:Z rgb:200,200,200
#=GF R2R_oneseq Hvo.1 tick_label ILOOPL:z ANA box

#=GF R2R_oneseq Hvo.2a shade_along_backbone KTURN:T rgb:0,129,255
#=GF R2R_oneseq Hvo.2a shade_along_backbone KTURN:R rgb:0,129,255
#=GF R2R_oneseq Hvo.2a tick_label KTURNL:r K-turn
#=GF R2R_oneseq Hvo.2a shade_along_backbone ILOOP:Y rgb:0,255,0
#=GF R2R_oneseq Hvo.2a shade_along_backbone ILOOP:S rgb:0,255,0
#=GF R2R_oneseq Hvo.2a shade_along_backbone ILOOP:U rgb:0,255,0
#=GF R2R_oneseq Hvo.2a shade_along_backbone ILOOP:P rgb:0,255,0
#=GF R2R_oneseq Hvo.2a shade_along_backbone ILOOP:Q rgb:0,255,0
#=GF R2R_oneseq Hvo.2a shade_along_backbone ILOOP:X rgb:0,255,0
#=GF R2R_oneseq Hvo.2a shade_along_backbone ILOOP:Z rgb:200,200,200
#=GF R2R_oneseq Hvo.2a tick_label ILOOPL:z ANA box

#=GF R2R_oneseq Hvo.2b shade_along_backbone KTURN:T rgb:0,129,255
#=GF R2R_oneseq Hvo.2b shade_along_backbone KTURN:R rgb:0,129,255
#=GF R2R_oneseq Hvo.2b tick_label KTURNL:r K-turn
#=GF R2R_oneseq Hvo.2b shade_along_backbone ILOOP:Y rgb:0,255,0
#=GF R2R_oneseq Hvo.2b shade_along_backbone ILOOP:S rgb:0,255,0
#=GF R2R_oneseq Hvo.2b shade_along_backbone ILOOP:U rgb:0,255,0
#=GF R2R_oneseq Hvo.2b shade_along_backbone ILOOP:P rgb:0,255,0
#=GF R2R_oneseq Hvo.2b shade_along_backbone ILOOP:Q rgb:0,255,0
#=GF R2R_oneseq Hvo.2b shade_along_backbone ILOOP:X rgb:0,255,0
#=GF R2R_oneseq Hvo.2b shade_along_backbone ILOOP:Z rgb:200,200,200
#=GF R2R_oneseq Hvo.2b tick_label ILOOPL:z ANA box

//
```

Listing 22: RNAMotif descriptor used to identify H/ACA Motifs in archaea from the H1H2 subfamily (3’ guide sequence between 5 and 8 nucleotides)

```
parms
        wc += gu;
descr
        h5 ( len=9, mispair=2, ends='mm' )
        ss ( minlen=5, maxlen=8 )
                  h5 ( len=8, mispair=3, ends='mm' )
                  ss ( len=2, seq="^RA" )
                  ss ( minlen=4, maxlen=22 )
                  ss ( len=3, seq="UGA$" )
                  h3
                  ss ( minlen=5, maxlen=6 )
        h3
        ss ( len=3, seq="ANA$")
```

Listing 23: RNAMotif descriptor used to identify H/ACA Motifs in archaea from the S1ac subfamily (3’ guide sequence between 5 and 8 nucleotides)

```
parms
        wc += gu;
descr
        h5 ( len=7, mispair=2, ends='mm' )
        ss ( len=1 )
               h5 ( len=2, mispair=1, ends='mm' )
               ss ( minlen=5, maxlen=8 )
                  h5 ( len=8, mispair=3, ends='mm' )
                  ss ( len=2, seq="^RA" )
                  ss ( minlen=4, maxlen=22 )
                  ss ( len=3, seq="UGA$" )
                  h3
                  ss ( minlen=5, maxlen=6 )
               h3
               ss ( len=1 )
        h3
        ss ( len=3, seq="ANA$")
```

Listing 24: RNAMotif descriptor used to identify H/ACA Motifs in archaea from the S1b subfamily (3’ guide sequence between 5 and 8 nucleotides)

```
parms
        wc += gu;
descr
        h5 ( len=8, mispair=2, ends='mm' )
        h5 ( len=1 )
        ss ( minlen=5, maxlen=8 )
                  h5 ( len=8, mispair=3, ends='mm' )
                  ss ( len=2, seq="^RA" )
                  ss ( minlen=4, maxlen=22 )
                  ss ( len=3, seq="UGA$" )
                  h3
                  ss ( minlen=5, maxlen=6 )
        h3
        ss ( len=1 )
        h3
        ss ( len=3, seq="ANA$")
```

Listing 25: RNAMotif descriptor used to identify H/ACA Motifs in archaea from the S1bM2b subfamily (3’ guide sequence between 5 and 8 nucleotides)

```
parms
        wc += gu;
descr
        h5 ( len=8, mispair=2, ends='mm' )
        h5 ( len=1, pair+={"c:u","u:c","g:a","a:g","a:c","c:a"} )
        ss ( minlen=5, maxlen=8 )
                  h5 ( len=2, pair+={"c:u","u:c","g:a","a:g","a:c","c:a"} )
                  ss ( len =1 )
                  h5 ( len=5, mispair=1, ends='mm' )
                  ss ( len=2, seq="^RA" )
                  ss ( minlen=4, maxlen=22 )
                  ss ( len=3, seq="UGA$" )
                  h3
                  h3
                  ss ( minlen=5, maxlen=6 )
        h3
        ss ( len=1 )
        h3
        ss ( len=3, seq="ANA$")
```

Listing 26: RNAMotif descriptor used to identify H/ACA Motifs in archaea from the M2a subfamily (3’ guide sequence between 5 and 8 nucleotides)

```
parms
        wc += gu;
descr
        h5 ( len=9, mispair=2, ends='mm' )
        ss ( minlen=5, maxlen=8 )
                h5 ( len=1, pair+={"c:u","u:c","g:a","a:g","a:c","c:a"} )
                ss ( len=1 )
                h5 ( len=5, mispair=1, ends='mm' )
                ss ( len=2, seq="^RA" )
                ss ( minlen=4, maxlen=22 )
                ss ( len=3, seq="UGA$" )
                h3
                h3
                ss ( minlen=5, maxlen=6 )
        h3
        ss ( len=3, seq="ANA$")
```

Listing 27: RNAMotif descriptor used to identify H/ACA Motifs in archaea from the S1b2a subfamily (3’ guide sequence between 5 and 8 nucleotides)

```
parms
        wc += gu;
descr
        h5 ( len=8, mispair=2, ends='mm' )
        h5 ( len=1, pair+={"c:u","u:c","g:a","a:g","a:c","c:a"} )
        ss ( minlen=5, maxlen=8 )
                h5 ( len=1, pair+={"c:u","u:c","g:a","a:g","a:c","c:a"} )
                ss ( len=1 )
                h5 ( len=7, mispair=2, ends='mm' )
                ss ( len=2, seq="^RA" )
                ss ( minlen=4, maxlen=22 )
                ss ( len=3, seq="UGA$" )
                h3
                h3
                ss ( minlen=5, maxlen=6 )
        h3
        ss ( len=1 )
        h3
        ss ( len=3, seq="ANA$")
```

Listing 28: RNAMotif descriptor used to identify H/ACA Motifs in archaea from the S2a subfamily (3’ guide sequence between 5 and 8 nucleotides)

```
parms
        wc += gu;
descr
        h5 ( len=9, mispair=2, ends='mm' )
        ss ( minlen=5, maxlen=8 )
                h5 ( len=1, pair+={"c:u","u:c","g:a","a:g","a:c","c:a"} )
                ss ( len=1 )
                h5 ( len=7, mispair=2, ends='mm' )
                ss ( len=2, seq="^RA" )
                ss ( minlen=4, maxlen=22 )
                ss ( len=3, seq="UGA$" )
                h3
                h3
                ss ( minlen=5, maxlen=6 )
        h3
        ss ( len=3, seq="ANA$")
```

Listing 29: RNAMotif descriptor used to identify H/ACA Motifs in archaea from the S2bg subfamily (3’ guide sequence between 5 and 8 nucleotides)

```
parms
        wc += gu;
descr
        h5 ( len=9, mispair=2, ends='mm' )
        ss ( minlen=5, maxlen=8 )
                h5 ( len=1, pair+={"c:u","u:c","g:a","a:g","a:c","c:a"})
                h5 ( len=1, pair+={"c:u","u:c","g:a","a:g","a:c","c:a"})
                ss ( len=1 )
                h5 ( len=6, mispair=2, ends='mm' )
                ss ( len=2, seq="^RA" )
                ss ( minlen=4, maxlen=22 )
                ss ( len=3, seq="UGA$" )
                h3
                h3
                ss ( len=1 )
                h3
        ss ( minlen=5, maxlen=6 )
        h3
        ss ( len=3, seq="ANA$")
```

Listing 30: RNAMotif descriptor used to identify H/ACA Motifs in archaea from the S2e subfamily (3’ guide sequence between 5 and 8 nucleotides)

```
parms
        wc += gu;
descr
        h5 ( len=9, mispair=2, ends='mm' )
        ss ( minlen=5, maxlen=8 )
                h5 ( len=4, mispair=1, ends='mm' )
                h5 ( len=4, mispair=1, ends='mm' )
                ss ( len=2, seq="^RA" )
                ss ( minlen=4, maxlen=22 )
                ss ( len=3, seq="UGA$" )
                h3
                ss ( len=1 )
                h3
                ss ( minlen=5, maxlen=6 )
        h3
        ss ( len=3, seq="ANA$")
```

Listing 31: RNAMotif descriptor used to identify H/ACA Motifs in archaea from the S2c subfamily (3’ guide sequence between 5 and 8 nucleotides)

```
parms
        wc += gu;
descr
        h5 ( len=9, mispair=2, ends='mm' )
        ss ( minlen=5, maxlen=8 )
                h5 ( len=7, mispair=2, ends='mm' )
                ss ( minlen=1, maxlen=6 )
                h5 ( len=1, pair+={"c:u","u:c","g:a","a:g","a:c","c:a"})
                ss ( len=2, seq="^RA" )
                ss ( minlen=4, maxlen=22 )
                ss ( len=3, seq="UGA$" )
                h3
                h3
                ss ( minlen=5, maxlen=6 )
        h3
        ss ( len=3, seq="ANA$")
```

Listing 32: RNAMotif descriptor used to identify H/ACA Motifs in archaea from the M2cS2dg subfamily (3’ guide sequence between 5 and 8 nucleotides)

```
parms
        wc += gu;
descr
        h5 ( len=9, mispair=2, ends='mm' )
        ss ( minlen=5, maxlen=8 )
           h5 ( len=1, pair+={"c:u","u:c","g:a","a:g","a:c","c:a"})
                h5 ( len=3, mispair=1, ends='mm' )
                h5 ( len=3, mispair=1, ends='mm' )
                ss ( len=2, seq="^RA" )
                ss ( minlen=4, maxlen=22 )
                ss ( len=3, seq="UGA$" )
                ss ( len=1 )
                h3
                ss ( len=1 )
                h3
                ss ( len=1 )
           h3
           ss ( minlen=5, maxlen=6 )
        h3
        ss ( len=3, seq="ANA$")
```

Listing 33: RNAMotif descriptor used to identify H/ACA Motifs in archaea from the S2f subfamily (3’ guide sequence between 5 and 8 nucleotides)

```
parms
        wc += gu;
descr
        h5 ( len=9, mispair=2, ends='mm' )
        ss ( minlen=5, maxlen=8 )
                h5 ( len=2, mispair=1, ends='mm' )
                h5 ( len=6, mispair=2, ends='mm' )
                ss ( len=2, seq="^RA" )
                ss ( minlen=4, maxlen=22 )
                ss ( len=3, seq="UGA$" )
                h3
                ss ( len=1 )
                h3
                ss ( minlen=5, maxlen=6 )
        h3
        ss ( len=3, seq="ANA$")
```

Listing 34: RNAMotif descriptor used to identify H/ACA Motifs in archaea from the S2eg subfamily (3’ guide sequence between 5 and 8 nucleotides)

```
parms
        wc += gu;
descr
        h5 ( len=9, mispair=2, ends='mm' )
        ss ( minlen=5, maxlen=8 )
                h5 ( len=1, pair+={"c:u","u:c","g:a","a:g","a:c","c:a"})
                h5 ( len=3, mispair=1, ends='mm' )
                h5 ( len=4, mispair=1, ends='mm' )
                ss ( len=2, seq="^RA" )
                ss ( minlen=4, maxlen=22 )
                ss ( len=3, seq="UGA$" )
                h3
                ss ( len=1 )
                h3
                ss ( len=1 )
                h3
                ss ( minlen=5, maxlen=6 )
        h3
        ss ( len=3, seq="ANA$")
```

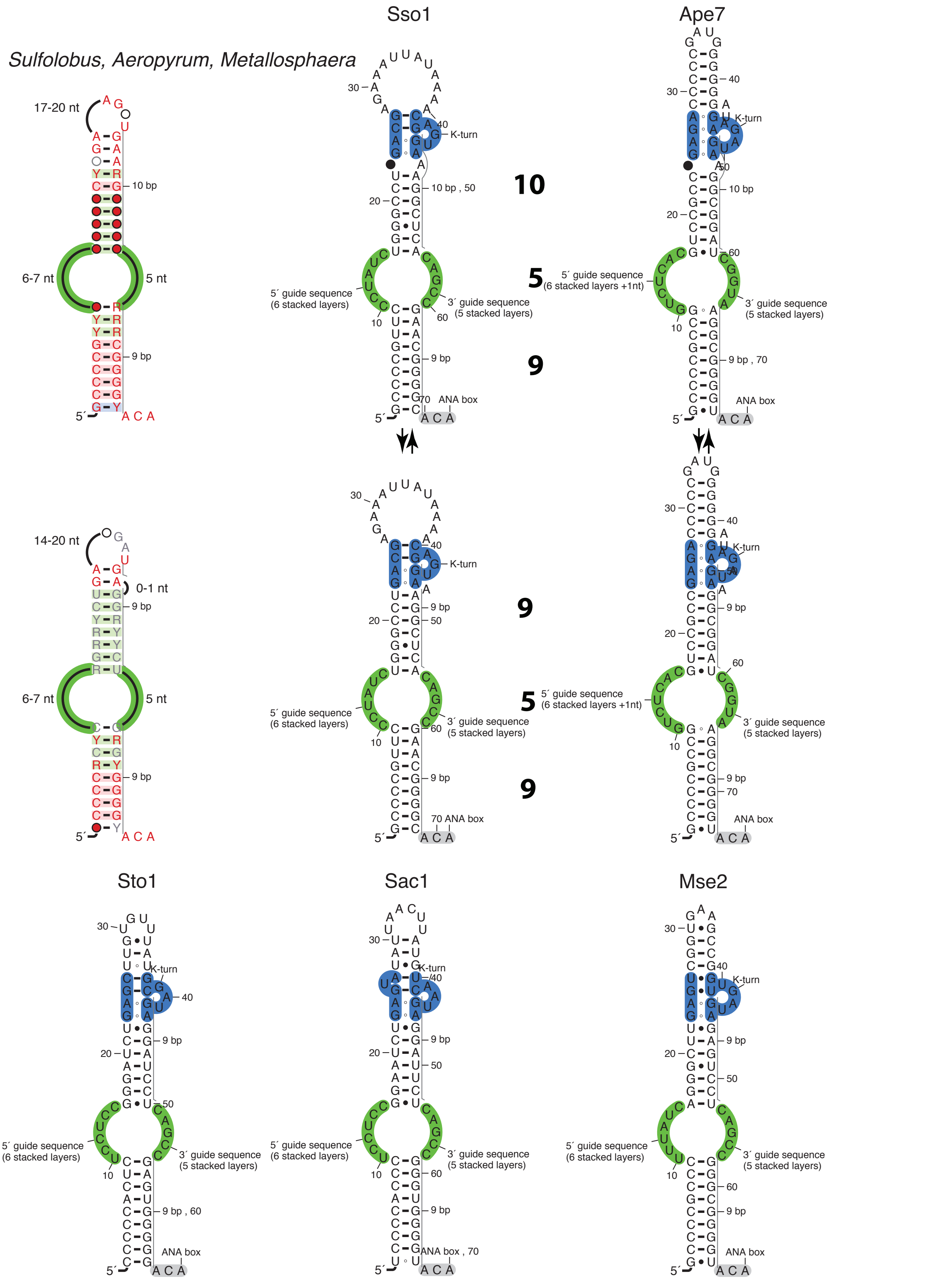

Figure S19. H/ACA motifs from Sulfolobales as guide RNAs targeting the position U35 in tRNA$^{Tyr}_{GUA}$. Two alternative folds are shown: 10/5/9 (*S. solfataricus* and *A. pernix*) and 9/5/9 (*S. tokodaii*, *S. acidocaldarius*, *M. sedula*).

Listing 35: Stockholm Alignment of H/ACA sRNA (sR201 & sR202) in *Pyrocabulum*.

```
# STOCKHOLM 1.0

Pae_sR201                    AA.UGCCCCU.......GGCGUG.GGGCGUC...GA....GGCGCCG....CCA.....CGGCCG.C...UGUGA.AAAC.GC.CCC.CUCCAA.GG.GGCGC...ACA
Psp1860_sR201                CG.CGCCCCU.......GGCGUG.GGGUGUC...GA....GGCGCCG....UCU.....CGGCGG.C...UGUGA.AAAC.GC.CCC.CUCCAA.GG.GGCGG...ACA
Pog_sR201                    CA.CGCCCCU.......GGCGUG.GGGUGUC...GA....GUCGCCG....UGU.....GGGCGG.C...UGUGA.AAAC.GC.CCC.CUCCGC.GG.GGCGU...ACA
Par_sR201                    CA.CGCCCCU.......GGCGUG.GGGUGUC...GA....GUCGCCG....UGU.....GGGCGG.C...UGUGA.AAAC.GC.CCC.CUCCGC.GG.GGCGU...ACA
Pca_sR201                    AG.UGCCCCU.......GGCGUG.GGGUGUC...GA....GCCGCCG....CAC.....AGGC.GCC...GGUGA.ACAC.GC.CCC.CUCCAA.GG.GGCGU...ACA
Pis_sR201                    UC.UGCCCCU.......GGCGUG.GGGUGUC...GA....GGCGCCG....UGU.....UGGCGG.C...UAUGA.AAAC.GC.CCC.CUCCGA.GG.GGCAG...AUA
Pae_sR202                    UUAUACAACC.......GCGACG.GGCUGUG...GA....G.AGCCGC..GCCC....GCGGC.G.C...CGUGA.A.AC.AG.CCU.GACGGU.GUCUGGGG...ACA
Psp1860_sR202                UUACACCACC.......GCGACG.GGCUGG....GA....G.GGCCGC..ACAC....GCGGC.G.C...CGUGA.G.AC.AG.CCC.GACGGU.GUCUGGGG...ACA
Pog_sR202                    UAACACAGCG.......ACAGGC.UGUUGA....GA....G.CCGCCU..CAGA....GGCGG.C.G..CUGUGA.G.AC.GG.CCU.GACGGU.GUCUGGGU...ACA
Par_sR202a                   UAACACACGA.......CAGGCU.GUUGA.....GA....G.CCGCCU..CAGA....GGCGG.C.G..CUGUGA...GA.CG.GCCUGACGGU.GUCUGGGU...ACA
Par_sR202b                   UAACACACGA.......CAGGCU..GUUGA....GA....G.CCGCCU..CAGA....GGCGG.C.G..CUGUGA.G.AC.GG.CCU.GACGGU.GUCUGGGU...ACA
Pca_sR202a                   AUAAACGCCG.......CGACGG.GCUGUC....GA....G.AGCCGC..GUCU....GCGGC.G.C...UGUGA...GA.CG.GCCUGACGGC.GU.CUGGG...CCA
Pca_sR202b                   AUAAACGCCG.......CGACGG..GCUGUC...GA....G.AGCCGC..GUCU....GCGGC.G.C...UGUGA.G.AC.GG.CCU.GACGGC.GU.CUGGG...CCA
Pis_sR202                    .CAAAAAUUC.......UAGAUG.GGCUACA...GA....G.AGCCGC..UUAC....GCGGC.A.C...UGUGA.U.GU.AG.CCU.GACGGU.GUCUGGGG...ACA
#=GC SS_cons                 .<.<<<<<<<............<-<<<<<<<...<<....<.<<<<<<__________>>>>>.>.>......>>.>->>->>->>>.....->->>.>>>>>......
#=GC R2R_LABEL               .q8...........1......2.3o.....v...II...4.......................j...5.....HH.wlHH.HiBtp.6....7H9HH0HhHHr......
#=GC R2R_XLABEL_KTURN        ..................................TTTTTTTT......................RRRRRRRRRRR..................................
#=GC R2R_XLABEL_KTURNL       ........................................................................r....................................
#=GC R2R_XLABEL_ILOOP        ...............PQXXXXX.W...............................................................YYYYSU.V...........ZZZ
#=GC R2R_XLABEL_ILOOPL       ..................x...w..................................................................ysu.v.............z
#=GC R2R_XLABEL_STEM         .............................................................................................................
#=GC R2R_XLABEL_STEML        .............................................................................................................
#=GC SUBFAM_LABEL_TERM       .......................................................................................x.....................
#=GC SUBFAM_GUID55_R2R_LABEL --------<.....1PQXxXXX.W>------------------------------------------------------------<.YYyYSU.V>-------------
#=GC SUBFAM_GUID56_R2R_LABEL --------<.....1PQXxXXX.W>------------------------------------------------------------<.YYyYSU.V>-------------

#=GF R2R keep q r
#=GF R2R keep s t
#=GF R2R keep v w
#=GF R2R keep o p
#=GF R2R var_backbone_range_size_fake_nucs 5 1 2
#=GF R2R var_backbone_range_size_fake_nucs 6 6 7
#=GF R2R var_backbone_range 4 5
#=GF R2R var_backbone_range_size_fake_nucs 1 1 l
#=GF R2R var_backbone_range_size_fake_nucs 1 8 8
#=GF R2R var_backbone_range_size_fake_nucs 1 0 0

#=GF R2R shade_along_backbone 1 rgb:0,255,0
#=GF R2R shade_along_backbone 6 rgb:0,255,0
#=GF R2R outline_nuc H
#=GF R2R outline_nuc h
#=GF R2R outline_nuc w
#=GF R2R outline_nuc p
#=GF R2R outline_nuc r
#=GF R2R outline_nuc i
#=GF R2R outline_nuc t
#=GF R2R tick_label h 8 bp
#=GF R2R tick_label i 10 bp

#=GF SUBFAM_REGEX_PRED HAS_GUID56 TERM [A-Z]
#=GF SUBFAM_REGEX_PRED HAS_GUID55 TERM [.]
#=GF SUBFAM_PERL_PRED GUID56 return $predValue{HAS_GUID56}
#=GF SUBFAM_PERL_PRED GUID55 return $predValue{HAS_GUID55}

#=GF SUBFAM_GUID56_R2R no5
#=GF SUBFAM_GUID56_R2R set_dir pos0 -90
#=GF SUBFAM_GUID56_R2R shade_along_backbone ILOOP:P rgb:0,255,0
#=GF SUBFAM_GUID56_R2R shade_along_backbone ILOOP:Q rgb:0,255,0
#=GF SUBFAM_GUID56_R2R shade_along_backbone ILOOP:X rgb:0,255,0
#=GF SUBFAM_GUID56_R2R outline_nuc X
#=GF SUBFAM_GUID56_R2R outline_nuc x
#=GF SUBFAM_GUID56_R2R tick_label x 5 nt (6-1)
#=GF SUBFAM_GUID56_R2R shade_along_backbone ILOOP:Y rgb:0,255,0
#=GF SUBFAM_GUID56_R2R shade_along_backbone ILOOP:S rgb:0,255,0
#=GF SUBFAM_GUID56_R2R shade_along_backbone ILOOP:U rgb:0,255,0
#=GF SUBFAM_GUID56_R2R outline_nuc Y
#=GF SUBFAM_GUID56_R2R outline_nuc y
#=GF SUBFAM_GUID56_R2R outline_nuc S
#=GF SUBFAM_GUID56_R2R outline_nuc U
#=GF SUBFAM_GUID56_R2R tick_label y 6 nt

#=GF SUBFAM_GUID55_R2R no5
#=GF SUBFAM_GUID55_R2R set_dir pos0 -90
#=GF SUBFAM_GUID55_R2R shade_along_backbone ILOOP:P rgb:0,255,0
#=GF SUBFAM_GUID55_R2R shade_along_backbone ILOOP:Q rgb:0,255,0
#=GF SUBFAM_GUID55_R2R shade_along_backbone ILOOP:X rgb:0,255,0
#=GF SUBFAM_GUID55_R2R outline_nuc X
#=GF SUBFAM_GUID55_R2R outline_nuc x
#=GF SUBFAM_GUID55_R2R tick_label x 5 nt (6-1)
#=GF SUBFAM_GUID55_R2R shade_along_backbone ILOOP:Y rgb:0,255,0
#=GF SUBFAM_GUID55_R2R shade_along_backbone ILOOP:S rgb:0,255,0
#=GF SUBFAM_GUID55_R2R shade_along_backbone ILOOP:U rgb:0,255,0
#=GF SUBFAM_GUID55_R2R outline_nuc Y
#=GF SUBFAM_GUID55_R2R outline_nuc y
#=GF SUBFAM_GUID55_R2R outline_nuc S
#=GF SUBFAM_GUID55_R2R outline_nuc U
#=GF SUBFAM_GUID55_R2R tick_label y 5 nt

#=GF R2R_oneseq Pae_sR201 shade_along_backbone KTURN:T rgb:0,129,255
#=GF R2R_oneseq Pae_sR201 shade_along_backbone KTURN:R rgb:0,129,255
#=GF R2R_oneseq Pae_sR201 tick_label KTURNL:r K-turn
#=GF R2R_oneseq Pae_sR201 shade_along_backbone ILOOP:Y rgb:0,255,0
#=GF R2R_oneseq Pae_sR201 shade_along_backbone ILOOP:S rgb:0,255,0
#=GF R2R_oneseq Pae_sR201 shade_along_backbone ILOOP:U rgb:0,255,0
#=GF R2R_oneseq Pae_sR201 tick_label ILOOPL:y 3' guide sequence \n (5 stacked layers)
#=GF R2R_oneseq Pae_sR201 shade_along_backbone ILOOP:P rgb:0,255,0
#=GF R2R_oneseq Pae_sR201 shade_along_backbone ILOOP:Q rgb:0,255,0
#=GF R2R_oneseq Pae_sR201 shade_along_backbone ILOOP:X rgb:0,255,0
#=GF R2R_oneseq Pae_sR201 tick_label ILOOPL:x 5' guide sequence \n (3 stacked layers +2nt)
#=GF R2R_oneseq Pae_sR201 shade_along_backbone ILOOP:Z rgb:200,200,200
#=GF R2R_oneseq Pae_sR201 tick_label ILOOPL:z ANA box
#=GF R2R_oneseq Pae_sR201 var_backbone_range_size_fake_nucs 1 j j 0-1 nt

#=GF R2R_oneseq Psp1860_sR201 shade_along_backbone KTURN:T rgb:0,129,255
#=GF R2R_oneseq Psp1860_sR201 shade_along_backbone KTURN:R rgb:0,129,255
#=GF R2R_oneseq Psp1860_sR201 tick_label KTURNL:r K-turn
#=GF R2R_oneseq Psp1860_sR201 shade_along_backbone ILOOP:Y rgb:0,255,0
```

```
#=GF R2R_oneseq Psp1860_sR201 shade_along_backbone ILOOP:S rgb:0,255,0
#=GF R2R_oneseq Psp1860_sR201 shade_along_backbone ILOOP:U rgb:0,255,0
#=GF R2R_oneseq Psp1860_sR201 tick_label ILOOPL:y 3' guide sequence \n (5 stacked layers)
#=GF R2R_oneseq Psp1860_sR201 shade_along_backbone ILOOP:P rgb:0,255,0
#=GF R2R_oneseq Psp1860_sR201 shade_along_backbone ILOOP:Q rgb:0,255,0
#=GF R2R_oneseq Psp1860_sR201 shade_along_backbone ILOOP:X rgb:0,255,0
#=GF R2R_oneseq Psp1860_sR201 tick_label ILOOPL:x 5' guide sequence \n (3 stacked layers +2nt)
#=GF R2R_oneseq Psp1860_sR201 shade_along_backbone ILOOP:Z rgb:200,200,200
#=GF R2R_oneseq Psp1860_sR201 tick_label ILOOPL:z ANA box

#=GF R2R_oneseq Pog_sR201 shade_along_backbone KTURN:T rgb:0,129,255
#=GF R2R_oneseq Pog_sR201 shade_along_backbone KTURN:R rgb:0,129,255
#=GF R2R_oneseq Pog_sR201 tick_label KTURNL:r K-turn
#=GF R2R_oneseq Pog_sR201 shade_along_backbone ILOOP:Y rgb:0,255,0
#=GF R2R_oneseq Pog_sR201 shade_along_backbone ILOOP:S rgb:0,255,0
#=GF R2R_oneseq Pog_sR201 shade_along_backbone ILOOP:U rgb:0,255,0
#=GF R2R_oneseq Pog_sR201 tick_label ILOOPL:y 3' guide sequence \n (5 stacked layers)
#=GF R2R_oneseq Pog_sR201 shade_along_backbone ILOOP:P rgb:0,255,0
#=GF R2R_oneseq Pog_sR201 shade_along_backbone ILOOP:Q rgb:0,255,0
#=GF R2R_oneseq Pog_sR201 shade_along_backbone ILOOP:X rgb:0,255,0
#=GF R2R_oneseq Pog_sR201 tick_label ILOOPL:x 5' guide sequence \n (3 stacked layers +2nt)
#=GF R2R_oneseq Pog_sR201 shade_along_backbone ILOOP:Z rgb:200,200,200
#=GF R2R_oneseq Pog_sR201 tick_label ILOOPL:z ANA box
#=GF R2R_oneseq Pog_sR201 var_backbone_range_size_fake_nucs 1 j j 0-1 nt

#=GF R2R_oneseq Par_sR201 shade_along_backbone KTURN:T rgb:0,129,255
#=GF R2R_oneseq Par_sR201 shade_along_backbone KTURN:R rgb:0,129,255
#=GF R2R_oneseq Par_sR201 tick_label KTURNL:r K-turn
#=GF R2R_oneseq Par_sR201 shade_along_backbone ILOOP:Y rgb:0,255,0
#=GF R2R_oneseq Par_sR201 shade_along_backbone ILOOP:S rgb:0,255,0
#=GF R2R_oneseq Par_sR201 shade_along_backbone ILOOP:U rgb:0,255,0
#=GF R2R_oneseq Par_sR201 tick_label ILOOPL:y 3' guide sequence \n (5 stacked layers)
#=GF R2R_oneseq Par_sR201 shade_along_backbone ILOOP:P rgb:0,255,0
#=GF R2R_oneseq Par_sR201 shade_along_backbone ILOOP:Q rgb:0,255,0
#=GF R2R_oneseq Par_sR201 shade_along_backbone ILOOP:X rgb:0,255,0
#=GF R2R_oneseq Par_sR201 tick_label ILOOPL:x 5' guide sequence \n (3 stacked layers +2nt)
#=GF R2R_oneseq Par_sR201 shade_along_backbone ILOOP:Z rgb:200,200,200
#=GF R2R_oneseq Par_sR201 tick_label ILOOPL:z ANA box
#=GF R2R_oneseq Par_sR201 var_backbone_range_size_fake_nucs 1 j j 0-1 nt

#=GF R2R_oneseq Pca_sR201 shade_along_backbone KTURN:T rgb:0,129,255
#=GF R2R_oneseq Pca_sR201 shade_along_backbone KTURN:R rgb:0,129,255
#=GF R2R_oneseq Pca_sR201 tick_label KTURNL:r K-turn
#=GF R2R_oneseq Pca_sR201 shade_along_backbone ILOOP:Y rgb:0,255,0
#=GF R2R_oneseq Pca_sR201 shade_along_backbone ILOOP:S rgb:0,255,0
#=GF R2R_oneseq Pca_sR201 shade_along_backbone ILOOP:U rgb:0,255,0
#=GF R2R_oneseq Pca_sR201 tick_label ILOOPL:y 3' guide sequence \n (5 stacked layers)
#=GF R2R_oneseq Pca_sR201 shade_along_backbone ILOOP:P rgb:0,255,0
#=GF R2R_oneseq Pca_sR201 shade_along_backbone ILOOP:Q rgb:0,255,0
#=GF R2R_oneseq Pca_sR201 shade_along_backbone ILOOP:X rgb:0,255,0
#=GF R2R_oneseq Pca_sR201 tick_label ILOOPL:x 5' guide sequence \n (3 stacked layers +2nt)
#=GF R2R_oneseq Pca_sR201 shade_along_backbone ILOOP:Z rgb:200,200,200
#=GF R2R_oneseq Pca_sR201 tick_label ILOOPL:z ANA box
#=GF R2R_oneseq Pca_sR201 var_backbone_range_size_fake_nucs 1 j j 0-1 nt

#=GF R2R_oneseq Pis_sR201 shade_along_backbone KTURN:T rgb:0,129,255
#=GF R2R_oneseq Pis_sR201 shade_along_backbone KTURN:R rgb:0,129,255
#=GF R2R_oneseq Pis_sR201 tick_label KTURNL:r K-turn
#=GF R2R_oneseq Pis_sR201 shade_along_backbone ILOOP:Y rgb:0,255,0
#=GF R2R_oneseq Pis_sR201 shade_along_backbone ILOOP:S rgb:0,255,0
#=GF R2R_oneseq Pis_sR201 shade_along_backbone ILOOP:U rgb:0,255,0
#=GF R2R_oneseq Pis_sR201 tick_label ILOOPL:y 3' guide sequence \n (5 stacked layers)
#=GF R2R_oneseq Pis_sR201 shade_along_backbone ILOOP:P rgb:0,255,0
#=GF R2R_oneseq Pis_sR201 shade_along_backbone ILOOP:Q rgb:0,255,0
#=GF R2R_oneseq Pis_sR201 shade_along_backbone ILOOP:X rgb:0,255,0
#=GF R2R_oneseq Pis_sR201 tick_label ILOOPL:x 5' guide sequence \n (3 stacked layers +2nt)
#=GF R2R_oneseq Pis_sR201 shade_along_backbone ILOOP:Z rgb:200,200,200
#=GF R2R_oneseq Pis_sR201 tick_label ILOOPL:z ANA box
#=GF R2R_oneseq Pis_sR201 var_backbone_range_size_fake_nucs 1 j j 0-1 nt

#=GF R2R_oneseq Pae_sR202 shade_along_backbone KTURN:T rgb:0,129,255
#=GF R2R_oneseq Pae_sR202 shade_along_backbone KTURN:R rgb:0,129,255
#=GF R2R_oneseq Pae_sR202 tick_label KTURNL:r K-turn
#=GF R2R_oneseq Pae_sR202 shade_along_backbone ILOOP:Y rgb:0,255,0
#=GF R2R_oneseq Pae_sR202 shade_along_backbone ILOOP:S rgb:0,255,0
#=GF R2R_oneseq Pae_sR202 shade_along_backbone ILOOP:U rgb:0,255,0
#=GF R2R_oneseq Pae_sR202 tick_label ILOOPL:y 3' guide sequence \n (5 stacked layers)
#=GF R2R_oneseq Pae_sR202 shade_along_backbone ILOOP:P rgb:0,255,0
#=GF R2R_oneseq Pae_sR202 shade_along_backbone ILOOP:Q rgb:0,255,0
#=GF R2R_oneseq Pae_sR202 shade_along_backbone ILOOP:X rgb:0,255,0
#=GF R2R_oneseq Pae_sR202 tick_label ILOOPL:x 5' guide sequence \n (3 stacked layers +2nt)
#=GF R2R_oneseq Pae_sR202 shade_along_backbone ILOOP:Z rgb:200,200,200
#=GF R2R_oneseq Pae_sR202 tick_label ILOOPL:z ANA box
#=GF R2R_oneseq Pae_sR202 var_backbone_range_size_fake_nucs 1 j j 0-1 nt

#=GF R2R_oneseq Psp1860_sR202 shade_along_backbone KTURN:T rgb:0,129,255
#=GF R2R_oneseq Psp1860_sR202 shade_along_backbone KTURN:R rgb:0,129,255
#=GF R2R_oneseq Psp1860_sR202 tick_label KTURNL:r K-turn
#=GF R2R_oneseq Psp1860_sR202 shade_along_backbone ILOOP:Y rgb:0,255,0
#=GF R2R_oneseq Psp1860_sR202 shade_along_backbone ILOOP:S rgb:0,255,0
#=GF R2R_oneseq Psp1860_sR202 shade_along_backbone ILOOP:U rgb:0,255,0
#=GF R2R_oneseq Psp1860_sR202 tick_label ILOOPL:y 3' guide sequence \n (5 stacked layers)
#=GF R2R_oneseq Psp1860_sR202 shade_along_backbone ILOOP:P rgb:0,255,0
#=GF R2R_oneseq Psp1860_sR202 shade_along_backbone ILOOP:Q rgb:0,255,0
#=GF R2R_oneseq Psp1860_sR202 shade_along_backbone ILOOP:X rgb:0,255,0
#=GF R2R_oneseq Psp1860_sR202 tick_label ILOOPL:x 5' guide sequence \n (3 stacked layers +2nt)
#=GF R2R_oneseq Psp1860_sR202 shade_along_backbone ILOOP:Z rgb:200,200,200
#=GF R2R_oneseq Psp1860_sR202 tick_label ILOOPL:z ANA box

#=GF R2R_oneseq Pog_sR202 shade_along_backbone KTURN:T rgb:0,129,255
#=GF R2R_oneseq Pog_sR202 shade_along_backbone KTURN:R rgb:0,129,255
#=GF R2R_oneseq Pog_sR202 tick_label KTURNL:r K-turn
#=GF R2R_oneseq Pog_sR202 shade_along_backbone ILOOP:Y rgb:0,255,0
#=GF R2R_oneseq Pog_sR202 shade_along_backbone ILOOP:S rgb:0,255,0
#=GF R2R_oneseq Pog_sR202 shade_along_backbone ILOOP:U rgb:0,255,0
#=GF R2R_oneseq Pog_sR202 tick_label ILOOPL:y 3' guide sequence \n (5 stacked layers)
#=GF R2R_oneseq Pog_sR202 shade_along_backbone ILOOP:P rgb:0,255,0
#=GF R2R_oneseq Pog_sR202 shade_along_backbone ILOOP:Q rgb:0,255,0
#=GF R2R_oneseq Pog_sR202 shade_along_backbone ILOOP:X rgb:0,255,0
#=GF R2R_oneseq Pog_sR202 tick_label ILOOPL:x 5' guide sequence \n (3 stacked layers +2nt)
#=GF R2R_oneseq Pog_sR202 shade_along_backbone ILOOP:Z rgb:200,200,200
```

```
#=GF R2R_oneseq Pog_sR202 tick_label ILOOPL:z ANA box
#=GF R2R_oneseq Pog_sR202 var_backbone_range_size_fake_nucs 1 j j 0-1 nt

#=GF R2R_oneseq Par_sR202a shade_along_backbone KTURN:T rgb:0,129,255
#=GF R2R_oneseq Par_sR202a shade_along_backbone KTURN:R rgb:0,129,255
#=GF R2R_oneseq Par_sR202a tick_label KTURNL:r K-turn
#=GF R2R_oneseq Par_sR202a shade_along_backbone ILOOP:Y rgb:0,255,0
#=GF R2R_oneseq Par_sR202a shade_along_backbone ILOOP:S rgb:0,255,0
#=GF R2R_oneseq Par_sR202a shade_along_backbone ILOOP:U rgb:0,255,0
#=GF R2R_oneseq Par_sR202a tick_label ILOOPL:y 3' guide sequence \n (5 stacked layers)
#=GF R2R_oneseq Par_sR202a shade_along_backbone ILOOP:P rgb:0,255,0
#=GF R2R_oneseq Par_sR202a shade_along_backbone ILOOP:Q rgb:0,255,0
#=GF R2R_oneseq Par_sR202a shade_along_backbone ILOOP:X rgb:0,255,0
#=GF R2R_oneseq Par_sR202a tick_label ILOOPL:x 5' guide sequence \n (3 stacked layers +2nt)
#=GF R2R_oneseq Par_sR202a shade_along_backbone ILOOP:Z rgb:200,200,200
#=GF R2R_oneseq Par_sR202a tick_label ILOOPL:z ANA box
#=GF R2R_oneseq Par_sR202a var_backbone_range_size_fake_nucs 1 j j 0-1 nt

#=GF R2R_oneseq Pca_sR202a shade_along_backbone KTURN:T rgb:0,129,255
#=GF R2R_oneseq Pca_sR202a shade_along_backbone KTURN:R rgb:0,129,255
#=GF R2R_oneseq Pca_sR202a tick_label KTURNL:r K-turn
#=GF R2R_oneseq Pca_sR202a shade_along_backbone ILOOP:Y rgb:0,255,0
#=GF R2R_oneseq Pca_sR202a shade_along_backbone ILOOP:S rgb:0,255,0
#=GF R2R_oneseq Pca_sR202a shade_along_backbone ILOOP:U rgb:0,255,0
#=GF R2R_oneseq Pca_sR202a tick_label ILOOPL:y 3' guide sequence \n (5 stacked layers)
#=GF R2R_oneseq Pca_sR202a shade_along_backbone ILOOP:P rgb:0,255,0
#=GF R2R_oneseq Pca_sR202a shade_along_backbone ILOOP:Q rgb:0,255,0
#=GF R2R_oneseq Pca_sR202a shade_along_backbone ILOOP:X rgb:0,255,0
#=GF R2R_oneseq Pca_sR202a tick_label ILOOPL:x 5' guide sequence \n (3 stacked layers +2nt)
#=GF R2R_oneseq Pca_sR202a shade_along_backbone ILOOP:Z rgb:200,200,200
#=GF R2R_oneseq Pca_sR202a tick_label ILOOPL:z ANA box
#=GF R2R_oneseq Pca_sR202a var_backbone_range_size_fake_nucs 1 j j 0-1 nt

#=GF R2R_oneseq Par_sR202b shade_along_backbone KTURN:T rgb:0,129,255
#=GF R2R_oneseq Par_sR202b shade_along_backbone KTURN:R rgb:0,129,255
#=GF R2R_oneseq Par_sR202b tick_label KTURNL:r K-turn
#=GF R2R_oneseq Par_sR202b shade_along_backbone ILOOP:Y rgb:0,255,0
#=GF R2R_oneseq Par_sR202b shade_along_backbone ILOOP:S rgb:0,255,0
#=GF R2R_oneseq Par_sR202b shade_along_backbone ILOOP:U rgb:0,255,0
#=GF R2R_oneseq Par_sR202b tick_label ILOOPL:y 3' guide sequence \n (5 stacked layers)
#=GF R2R_oneseq Par_sR202b shade_along_backbone ILOOP:P rgb:0,255,0
#=GF R2R_oneseq Par_sR202b shade_along_backbone ILOOP:Q rgb:0,255,0
#=GF R2R_oneseq Par_sR202b shade_along_backbone ILOOP:X rgb:0,255,0
#=GF R2R_oneseq Par_sR202b tick_label ILOOPL:x 5' guide sequence \n (3 stacked layers +2nt)
#=GF R2R_oneseq Par_sR202b shade_along_backbone ILOOP:Z rgb:200,200,200
#=GF R2R_oneseq Par_sR202b tick_label ILOOPL:z ANA box
#=GF R2R_oneseq Par_sR202b var_backbone_range_size_fake_nucs 1 j j 0-1 nt

#=GF R2R_oneseq Pca_sR202b shade_along_backbone KTURN:T rgb:0,129,255
#=GF R2R_oneseq Pca_sR202b shade_along_backbone KTURN:R rgb:0,129,255
#=GF R2R_oneseq Pca_sR202b tick_label KTURNL:r K-turn
#=GF R2R_oneseq Pca_sR202b shade_along_backbone ILOOP:Y rgb:0,255,0
#=GF R2R_oneseq Pca_sR202b shade_along_backbone ILOOP:S rgb:0,255,0
#=GF R2R_oneseq Pca_sR202b shade_along_backbone ILOOP:U rgb:0,255,0
#=GF R2R_oneseq Pca_sR202b tick_label ILOOPL:y 3' guide sequence \n (5 stacked layers)
#=GF R2R_oneseq Pca_sR202b shade_along_backbone ILOOP:P rgb:0,255,0
#=GF R2R_oneseq Pca_sR202b shade_along_backbone ILOOP:Q rgb:0,255,0
#=GF R2R_oneseq Pca_sR202b shade_along_backbone ILOOP:X rgb:0,255,0
#=GF R2R_oneseq Pca_sR202b tick_label ILOOPL:x 5' guide sequence \n (3 stacked layers +2nt)
#=GF R2R_oneseq Pca_sR202b shade_along_backbone ILOOP:Z rgb:200,200,200
#=GF R2R_oneseq Pca_sR202b tick_label ILOOPL:z ANA box
#=GF R2R_oneseq Pca_sR202b var_backbone_range_size_fake_nucs 1 j j 0-1 nt

#=GF R2R_oneseq Pis_sR202 shade_along_backbone KTURN:T rgb:0,129,255
#=GF R2R_oneseq Pis_sR202 shade_along_backbone KTURN:R rgb:0,129,255
#=GF R2R_oneseq Pis_sR202 tick_label KTURNL:r K-turn
#=GF R2R_oneseq Pis_sR202 shade_along_backbone ILOOP:Y rgb:0,255,0
#=GF R2R_oneseq Pis_sR202 shade_along_backbone ILOOP:S rgb:0,255,0
#=GF R2R_oneseq Pis_sR202 shade_along_backbone ILOOP:U rgb:0,255,0
#=GF R2R_oneseq Pis_sR202 tick_label ILOOPL:y 3' guide sequence \n (5 stacked layers)
#=GF R2R_oneseq Pis_sR202 shade_along_backbone ILOOP:P rgb:0,255,0
#=GF R2R_oneseq Pis_sR202 shade_along_backbone ILOOP:Q rgb:0,255,0
#=GF R2R_oneseq Pis_sR202 shade_along_backbone ILOOP:X rgb:0,255,0
#=GF R2R_oneseq Pis_sR202 tick_label ILOOPL:x 5' guide sequence \n (3 stacked layers +2nt)
#=GF R2R_oneseq Pis_sR202 shade_along_backbone ILOOP:Z rgb:200,200,200
#=GF R2R_oneseq Pis_sR202 tick_label ILOOPL:z ANA box
#=GF R2R_oneseq Pis_sR202 var_backbone_range_size_fake_nucs 1 j j 0-1 nt

//
```

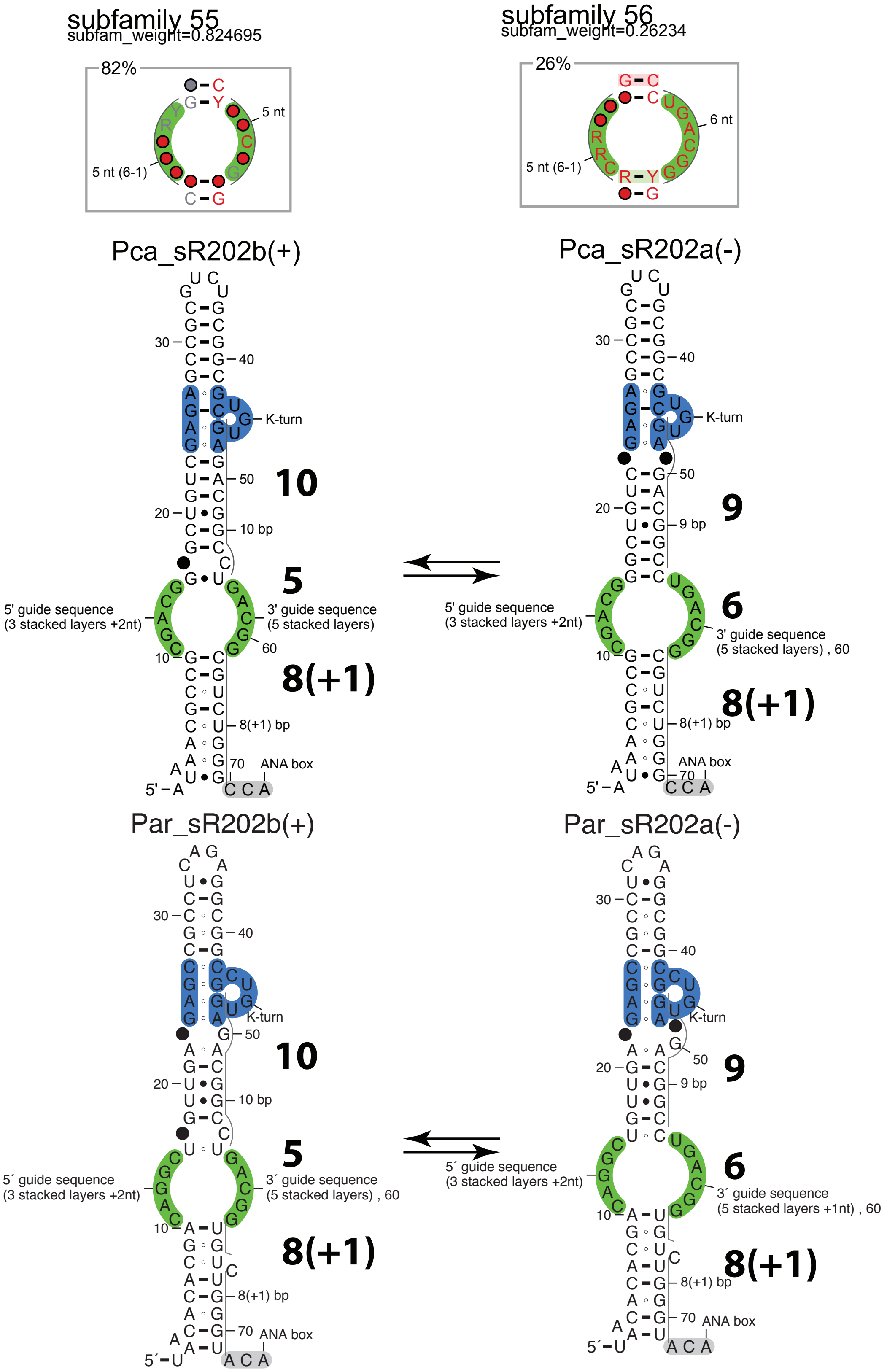

Figure S20. Switching between the subfamilies 55 and 56 in the canonical H/ACA motifs from *Pyrobaculum*.

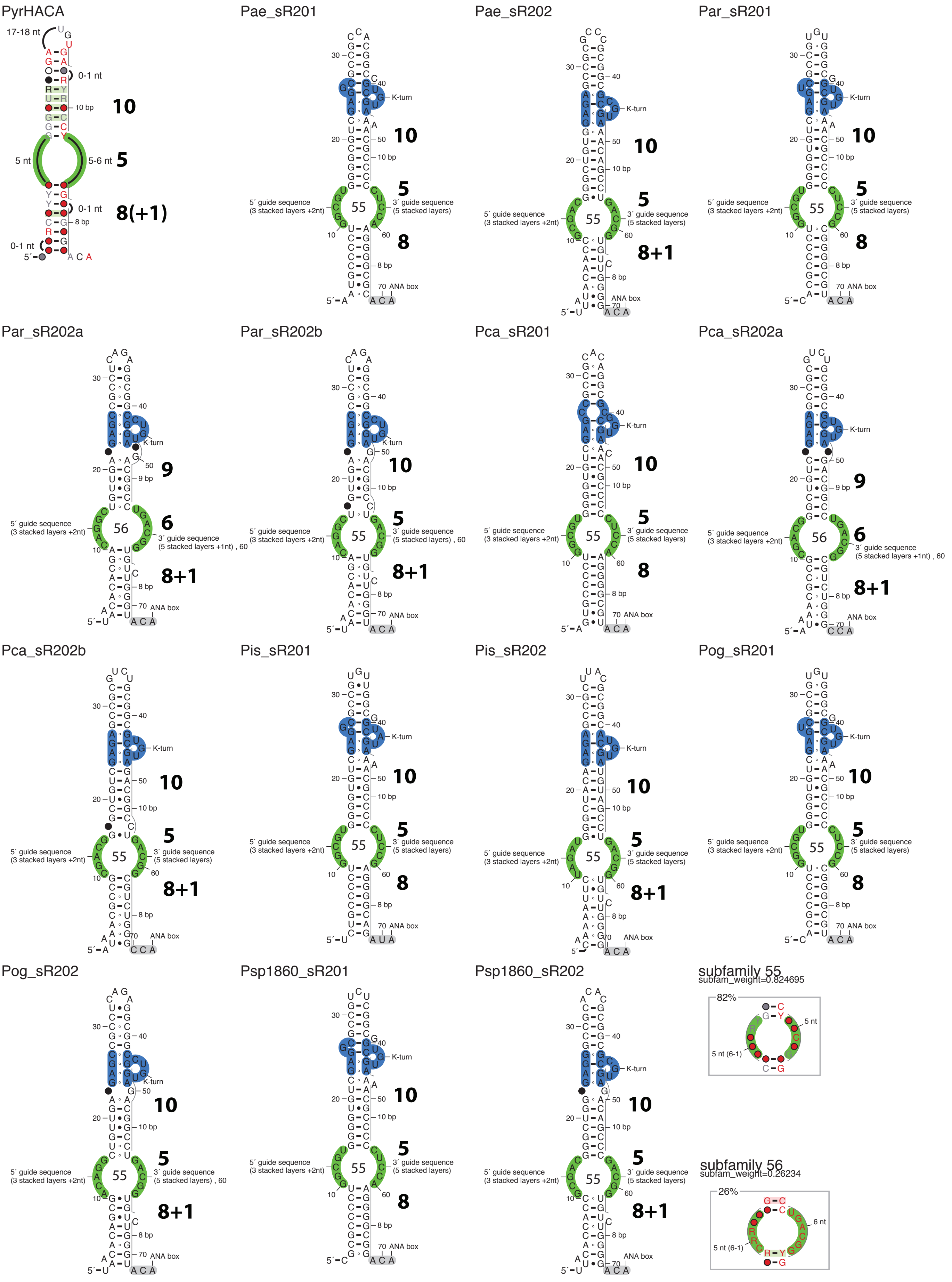

Figure S21. Full List of Canonical H/ACA Motifs (sR201 and sR202) in *Pyrobaculum*.

Listing 36: Stockholm Alignment of H/ACA-like sRNA (sR203 to sR210, except sR207) in *Pyrocabulum*.

```
# STOCKHOLM 1.0

Pis_sR204                  UUCUAU..C.UU.A.......UAGAGUG.CCG.CU.G.GA....G.UGCCGCC..CUA....GG.CGGC.U.C...UAUGA.U.AG.U..C.GGC..CCCCC....G.GC.C.UUAA.............AUUUU.....AAA
Pis_sR205                  AUGUAA..A.AG.A.......AAAACGC.CCU.AGGU.GA....C.ACCCCU...GCAA....G.GGGG.C.G...UAUGA.A.CC.U....GGC..GGGUUAC..C.CC.G.GCC...........AGA............
Pog_sR208                  CAAAAA..A.AC.U.......GUUCUCU.GGG.GC.U.GA....G.UAGCCCC..UAC....GG.GGCG.U.C...GAUGA.A.AG.C..U.CCG..CGUACGCC.A.GU.G.G.............UCA.........GCA
Pae_sR206                  CCCCUC..C.CA.U.......UAAUCAUGGCG..GCU.GA....G.AGCCCC...GGA.....G.GGGC.C.C...CGUGA.G.GC.AACC.GCA..GUCGGUC..C.CC.C.UUGC..........ACA............
Pae_sR209                  UUUUAU..A.CG.G.......CAGGAUC.AGC.CC.A.GA....G.CCGGCC...GGAC....G.GCCG.C.C...UGUGA.U.AG.G..G.GCC..CCG.GUC..A.AA.A.CCUC.............UGUUUGU......
Psp1860_sR209              CCAAGG..A.GG.C.......AGGAUCG.GCC.CC.G.GA....G.ACGGCCC..GCC....GG.GCCG.C.C...GGUGA.U.AG.G..G.GCU..CCG.GCU..A.AA.A.CCUC.............CUCCUAU......
Pog_sR209                  UAUACA..A.GG.C.......AGGAUCG.GCCCCAGA.GA...CG.GCCG.....AGGCG......CCG.C.C...UAUGA.G.GG.G..G.GCU..CCG.GUU..C.CU.C.UACG.............GCGU......AAA
Par_sR209                  UAUGCA..A.GG.C.......AGGAUCG.GCCCCAGA.GA...CG.GCCG.....AGGCG......CCG.C.C...UGUGA.G.GG.G..G.GCU..CCG.GUU..C.CU.C.UACG.............GCGU......AAA
Pis_sR209                  AAAAAA..C.GG.C.......AGGAUCA.GUCCCCGA.GA...CG.GCCG.....UAGG.......CCG.C.C...UAUGA.A.AG.G..G.GCU..CCG.GAC..A.AC.U.ACGC..........AAA.........AAA
Pog_sR204                  CUAGUC...UCU.A.......CAGAGUG.CCG.CC.C.GA....G.AGCCGC...UUCU....G.CGGC.C.C...AGUGA.C.CG.G..C.GGU..UCC.CCG..G.CUA..UUAA.............GCCUACUUCGCCA
Par_sR204                  CUAGUC...UCU.A.......CAGAGUG.CCG.CC.C.GA....G.AGCCGC...UUCU....G.CGGC.C.C...AGUGA.C.CG.G..C.GGU..UCC.CCG..G.CUA..UUAA.............GCCUACUUCGCCA
Psp1860_sR205              UGGAUA...UAU.A.......GGAAACG.CCCUGGGC.AA....C.UCCCCU...GUAG....G.GGGG.C.G...AAUGA.G.CC.U....GGU..GGGUUAC..C.CCG..GCC...........AAA..UUUUAU..AUA
Psp1860_sR206              AGCGCU...AGA.C.......AGUACACGGCG..GCU.GA....G.AGCCCC...GGA.....G.GGGC.C.C...GGUGA.G.CG.CCGC.AGU..CGGUCC...C.CCU..UGU...........ACA.........ACA
Pis_sR210                  AGAAUA...UCC.A.......AAGUUCU.GGG.CU.U.GA....C.AGCCGC...GUCU....G.CGGU.U.G...UAUGA.C.AA.G..C.CCG..CCC.GCG..G.CCG..AGUGU..........ACA............
Pae_sR208                  UUUGCU...GUA.U.......UCUCCCC.GGG.GC.A.GA....G.AGGCCCU..AUU....GG.GGCC.G.C...UGUGA.C.AG.C..U.CCG..CGUACGC..C.AGU..GGC...........CCA............
Pae_sR205                  GAGUAA...UAA.A.......GGAUUAG.CCCUAGGU.AA....C.ACCCCU...GAGC....G.GGGG.C.G...GAUGA.G.CC.U....GGC..GGGUUAC..C.CCG..GCC...........AAA............
Psp1860_sR204              CCUCAU..C.CAU........CUGAGUG.CCG.CC.G.GA....G.AGCCGCU..CCC....AG.CGGC.C.C...CAUGA.G.AG.G..C.GGU..UCC.CCG...GCC.U.CUUU..........AAA..UAG.....AAA
Pca_sR204                  GCUUAU..U.CUC........UUGAGUG.CCG.CC.G.GA....G.UGCCGCA..GAUG...UG.CGGG.C.C...GAUGA.A.AG.G..C.GGU..UCC.CC....GGC.U.UUUAU..............GCGG....AAA
Par_sR208                  ACCAAA..A.ACU........GUUCUCU.GGG.GC.U.GA....G.UAGCCCC..UAC....GG.GGCG.U.C...GAUGA.A.AG.C..U.CCG..CGUACGCC..AGU.G.G.............UCA.........GCA
Psp1860_sR203              UCUCUU...GCCG.......GGACUAC.GCC.GGAC.GA....G.GGCGGC...GGAC....G.CCGC.G.C...UGUGA.A.UC.C..G.GCG..GGG.CAC...CGCC..CCG...........UCA..GUUU....AUA
Pog_sR203                  UGUAGC...GCCG.......GGACUAC.GCC.GGGU.GA....G.AGCGGC...UGGC....G.CCGC.A.C...UGUGG.G.CC.C..G.GCG..GGG.UGC...CUUC..CCC...........AAA............
Par_sR203                  UGUAGC...GCCG.......GGACUAC.GCC.GGGU.GA....G.AGCGGC...UGGC....G.CCGC.A.C...UGUGG.G.CC.C..G.GCG..GGG.UGC...CUUC..CCUC.............AAGUUUUUA....
Pca_sR203                  CCCACU...GCCG.......GGACUAC.GCC.GGGC.AA....G.GCCGGCG..CCG....UG.CCGC.G.C...UGUGA.C.CC.C..G.GCG..GGG.CGC...CUCC..CCUC.............UCUUUA.......
Pog_sR205                  GAGUUG...UUAG.......AUAACAC.CCU.GGGC.AA....C.ACCCCU...GUAGG...G.GGGG.C.G...AAUGA.G.CC.U....GGU..GGGUUAC...CCCG..GCC...........AUA..UGUUU...AUA
Par_sR205                  GAGUUG...UUAG.......AUAACAC.CCU.GGGC.AA....C.ACCCCU...GUAG....G.GGGG.C.G...AAUGA.G.CC.U....GGU..GGGUUAC...CCCG..GCC...........AUA..UGUUU...AUA
Pae_sR210                  UGUGGU...UUAU.......GGAAAAC.GGG.CU.G.GA....C.UGCCGU...GGUC....G.CGGC.U.G...AAUGA.A.AA.G..C.CCG..CCC.GCG...GCCG..AGUCU..........ACA............
Psp1860_sR210              UGUUUG...UACU.......GUGGAUC.GGG.CU.G.GA....C.UGCCGU...GGAU....G.CGGC.U.G...GAUGA.G.GG.G..C.CCG..CCC.GCG...GCCG..AGUCU..........ACA............
Pae_sR203                  CCUUGU...GCCG.......GGACUAC.GCC.GGGU.GA....G.AGCGGC...GUGU....G.CCGC.C.C...UGUGA.A.UC.C..G.GCG..GGG.UGC...CUUC..CCCU.............CUAGUUU...AUA
Pae_sR204                  ACCUAU...UAUC.......ACGAGUG.CCG.CC.G.GA....G.AGCCGCG..CGC....CG.CGGC.C.C...CAUGA.G.CG.G..C.GGU..UCC.CCG...GCCA..UAUU.............UCUCUUU...AAA
#=GC SS_cons               .........<..<..............<.<<<.<<<<.<<....<.<<<<<<<_________>>.>>>>.>.>......>>.>.>>.>..>.>>>............>..>..............................
#=GC R2R_LABEL             1.......2a..b3...........4.u..mvo.g..II5...............................6.....HH.H.hi.pstn.HHH7..........8b..a9..............0...............
#=GC SUBFAM_LABEL_TERM1    .........x.......................................................................................x.......................................
#=GC SUBFAM_LABEL_TERM2    ............x..................................................................................x........................................
#=GC R2R_XLABEL_KTURN      ..................................TTTTTTTTT.....................RRRRRRRRRRR..........................................................
#=GC R2R_XLABEL_KTURNL     ..............................................................r.......................................................................
#=GC R2R_XLABEL_ILOOP      XXXXXXXXXXXXXXXXXXXXXXXXXXX..........................................................YYYYYYYYYYYYYYYYYYYYYYYYYYYYYYYYYYZZZ..........ZZZ
#=GC R2R_XLABEL_ILOOPL     ......................x.....................................................................y..........................................
#=GC SUBFAM_NONE_R2R_LABEL 1.XXXXXX2............XXXXX..>----------------------------------------------------------------<................9XXXXXXXXXXXXXXX0ZZZ..........ZZZ
#=GC SUBFAM_BP1_R2R_LABEL  1.XXXXXX2............XXXXX..>----------------------------------------------------------------<................9XXXXXXXXXXXXXXX0ZZZ..........ZZZ
#=GC SUBFAM_BP2_R2R_LABEL  1.XXXXXX2............XXXXX..>----------------------------------------------------------------<................9XXXXXXXXXXXXXXX0ZZZ..........ZZZ
#=GC SUBFAM_BPS_R2R_LABEL  1.XXXXXX2............XXXXX..>----------------------------------------------------------------<................9XXXXXXXXXXXXXXX0ZZZ..........ZZZ

#=GF R2R keep g h
#=GF R2R keep m n
#=GF R2R keep o p
#=GF R2R var_backbone_range 5 6
#=GF R2R var_backbone_range_size_fake_nucs 6 1 2
#=GF R2R var_backbone_range_size_fake_nucs 15 9 0
#=GF R2R var_backbone_range_size_fake_nucs 1 u u
#=GF R2R var_backbone_range_size_fake_nucs 1 v v
#=GF R2R var_backbone_range_size_fake_nucs 2 s t
#=GF R2R outline_nuc H
#=GF R2R outline_nuc h
#=GF R2R outline_nuc i
#=GF R2R outline_nuc n
#=GF R2R outline_nuc p
#=GF R2R tick_label i 10 bp
#=GF R2R shade_along_backbone ILOOP:X rgb:0,255,0
#=GF R2R shade_along_backbone ILOOP:Y rgb:0,255,0

#=GF SUBFAM_REGEX_PRED HAS_BP1 TERM1 [A-Z][A-Z]
#=GF SUBFAM_REGEX_PRED HAS_BP2 TERM2 [A-Z][A-Z]
#=GF SUBFAM_REGEX_PRED HAS_NOBP1 TERM1 [-.][-.]
#=GF SUBFAM_REGEX_PRED HAS_NOBP2 TERM2 [-.][-.]
#=GF SUBFAM_PERL_PRED NONE return ( $predValue{HAS_NOBP1} && $predValue{HAS_NOBP2} );
#=GF SUBFAM_PERL_PRED BP1 return    $predValue{HAS_BP1};
#=GF SUBFAM_PERL_PRED BP2 return    $predValue{HAS_BP2};
#=GF SUBFAM_PERL_PRED BPS return  ( $predValue{HAS_BP1} && $predValue{HAS_BP2} );


#=GF SUBFAM_NONE_R2R no5
#=GF SUBFAM_NONE_R2R set_dir pos0 0
#=GF SUBFAM_NONE_R2R shade_along_backbone ILOOP:X rgb:0,255,0
#=GF SUBFAM_NONE_R2R shade_along_backbone ILOOP:Y rgb:0,255,0
#=GF SUBFAM_NONE_R2R var_backbone_range_size_fake_nucs 6 1 2
#=GF SUBFAM_NONE_R2R var_backbone_range_size_fake_nucs 15 9 0

#=GF SUBFAM_BP1_R2R no5
#=GF SUBFAM_BP1_R2R set_dir pos0 0
#=GF SUBFAM_BP1_R2R shade_along_backbone ILOOP:X rgb:0,255,0
#=GF SUBFAM_BP1_R2R shade_along_backbone ILOOP:Y rgb:0,255,0
#=GF SUBFAM_BP1_R2R var_backbone_range_size_fake_nucs 6 1 2
#=GF SUBFAM_BP1_R2R var_backbone_range_size_fake_nucs 15 9 0

#=GF SUBFAM_BP2_R2R no5
#=GF SUBFAM_BP2_R2R set_dir pos0 0
#=GF SUBFAM_BP2_R2R shade_along_backbone ILOOP:X rgb:0,255,0
#=GF SUBFAM_BP2_R2R shade_along_backbone ILOOP:Y rgb:0,255,0
#=GF SUBFAM_BP2_R2R var_backbone_range_size_fake_nucs 6 1 2
#=GF SUBFAM_BP2_R2R var_backbone_range_size_fake_nucs 15 9 0

#=GF SUBFAM_BPS_R2R no5
#=GF SUBFAM_BPS_R2R set_dir pos0 0
#=GF SUBFAM_BPS_R2R shade_along_backbone ILOOP:X rgb:0,255,0
#=GF SUBFAM_BPS_R2R shade_along_backbone ILOOP:Y rgb:0,255,0
#=GF SUBFAM_BPS_R2R var_backbone_range_size_fake_nucs 6 1 2
#=GF SUBFAM_BPS_R2R var_backbone_range_size_fake_nucs 15 9 0


#=GF R2R_oneseq Pae_sR203 shade_along_backbone KTURN:T rgb:0,129,255
#=GF R2R_oneseq Pae_sR203 shade_along_backbone KTURN:R rgb:0,129,255
#=GF R2R_oneseq Pae_sR203 tick_label KTURNL:r K-turn
#=GF R2R_oneseq Pae_sR203 shade_along_backbone ILOOP:Y rgb:0,255,0
#=GF R2R_oneseq Pae_sR203 tick_label ILOOPL:y 3' guide sequence
#=GF R2R_oneseq Pae_sR203 shade_along_backbone ILOOP:X rgb:0,255,0
#=GF R2R_oneseq Pae_sR203 tick_label ILOOPL:x 5' guide sequence
#=GF R2R_oneseq Pae_sR203 shade_along_backbone ILOOP:Z rgb:200,200,200
```

```
#=GF R2R_oneseq Psp1860_sR203 shade_along_backbone KTURN:T rgb:0,129,255
#=GF R2R_oneseq Psp1860_sR203 shade_along_backbone KTURN:R rgb:0,129,255
#=GF R2R_oneseq Psp1860_sR203 tick_label KTURNL:r K-turn
#=GF R2R_oneseq Psp1860_sR203 shade_along_backbone ILOOP:Y rgb:0,255,0
#=GF R2R_oneseq Psp1860_sR203 tick_label ILOOPL:y 3' guide sequence
#=GF R2R_oneseq Psp1860_sR203 shade_along_backbone ILOOP:X rgb:0,255,0
#=GF R2R_oneseq Psp1860_sR203 tick_label ILOOPL:x 5' guide sequence
#=GF R2R_oneseq Psp1860_sR203 shade_along_backbone ILOOP:Z rgb:200,200,200

#=GF R2R_oneseq Pog_sR203 shade_along_backbone KTURN:T rgb:0,129,255
#=GF R2R_oneseq Pog_sR203 shade_along_backbone KTURN:R rgb:0,129,255
#=GF R2R_oneseq Pog_sR203 tick_label KTURNL:r K-turn
#=GF R2R_oneseq Pog_sR203 shade_along_backbone ILOOP:Y rgb:0,255,0
#=GF R2R_oneseq Pog_sR203 tick_label ILOOPL:y 3' guide sequence
#=GF R2R_oneseq Pog_sR203 shade_along_backbone ILOOP:X rgb:0,255,0
#=GF R2R_oneseq Pog_sR203 tick_label ILOOPL:x 5' guide sequence
#=GF R2R_oneseq Pog_sR203 shade_along_backbone ILOOP:Z rgb:200,200,200

#=GF R2R_oneseq Par_sR203 shade_along_backbone KTURN:T rgb:0,129,255
#=GF R2R_oneseq Par_sR203 shade_along_backbone KTURN:R rgb:0,129,255
#=GF R2R_oneseq Par_sR203 tick_label KTURNL:r K-turn
#=GF R2R_oneseq Par_sR203 shade_along_backbone ILOOP:Y rgb:0,255,0
#=GF R2R_oneseq Par_sR203 tick_label ILOOPL:y 3' guide sequence
#=GF R2R_oneseq Par_sR203 shade_along_backbone ILOOP:X rgb:0,255,0
#=GF R2R_oneseq Par_sR203 tick_label ILOOPL:x 5' guide sequence
#=GF R2R_oneseq Par_sR203 shade_along_backbone ILOOP:Z rgb:200,200,200

#=GF R2R_oneseq Pca_sR203 shade_along_backbone KTURN:T rgb:0,129,255
#=GF R2R_oneseq Pca_sR203 shade_along_backbone KTURN:R rgb:0,129,255
#=GF R2R_oneseq Pca_sR203 tick_label KTURNL:r K-turn
#=GF R2R_oneseq Pca_sR203 shade_along_backbone ILOOP:Y rgb:0,255,0
#=GF R2R_oneseq Pca_sR203 tick_label ILOOPL:y 3' guide sequence
#=GF R2R_oneseq Pca_sR203 shade_along_backbone ILOOP:X rgb:0,255,0
#=GF R2R_oneseq Pca_sR203 tick_label ILOOPL:x 5' guide sequence
#=GF R2R_oneseq Pca_sR203 shade_along_backbone ILOOP:Z rgb:200,200,200

#=GF R2R_oneseq Pae_sR204 shade_along_backbone KTURN:T rgb:0,129,255
#=GF R2R_oneseq Pae_sR204 shade_along_backbone KTURN:R rgb:0,129,255
#=GF R2R_oneseq Pae_sR204 tick_label KTURNL:r K-turn
#=GF R2R_oneseq Pae_sR204 shade_along_backbone ILOOP:Y rgb:0,255,0
#=GF R2R_oneseq Pae_sR204 tick_label ILOOPL:y 3' guide sequence
#=GF R2R_oneseq Pae_sR204 shade_along_backbone ILOOP:X rgb:0,255,0
#=GF R2R_oneseq Pae_sR204 tick_label ILOOPL:x 5' guide sequence
#=GF R2R_oneseq Pae_sR204 shade_along_backbone ILOOP:Z rgb:200,200,200

#=GF R2R_oneseq Psp1860_sR204 shade_along_backbone KTURN:T rgb:0,129,255
#=GF R2R_oneseq Psp1860_sR204 shade_along_backbone KTURN:R rgb:0,129,255
#=GF R2R_oneseq Psp1860_sR204 tick_label KTURNL:r K-turn
#=GF R2R_oneseq Psp1860_sR204 shade_along_backbone ILOOP:Y rgb:0,255,0
#=GF R2R_oneseq Psp1860_sR204 tick_label ILOOPL:y 3' guide sequence
#=GF R2R_oneseq Psp1860_sR204 shade_along_backbone ILOOP:X rgb:0,255,0
#=GF R2R_oneseq Psp1860_sR204 tick_label ILOOPL:x 5' guide sequence
#=GF R2R_oneseq Psp1860_sR204 shade_along_backbone ILOOP:Z rgb:200,200,200

#=GF R2R_oneseq Pog_sR204 shade_along_backbone KTURN:T rgb:0,129,255
#=GF R2R_oneseq Pog_sR204 shade_along_backbone KTURN:R rgb:0,129,255
#=GF R2R_oneseq Pog_sR204 tick_label KTURNL:r K-turn
#=GF R2R_oneseq Pog_sR204 shade_along_backbone ILOOP:Y rgb:0,255,0
#=GF R2R_oneseq Pog_sR204 tick_label ILOOPL:y 3' guide sequence
#=GF R2R_oneseq Pog_sR204 shade_along_backbone ILOOP:X rgb:0,255,0
#=GF R2R_oneseq Pog_sR204 tick_label ILOOPL:x 5' guide sequence
#=GF R2R_oneseq Pog_sR204 shade_along_backbone ILOOP:Z rgb:200,200,200

#=GF R2R_oneseq Par_sR204 shade_along_backbone KTURN:T rgb:0,129,255
#=GF R2R_oneseq Par_sR204 shade_along_backbone KTURN:R rgb:0,129,255
#=GF R2R_oneseq Par_sR204 tick_label KTURNL:r K-turn
#=GF R2R_oneseq Par_sR204 shade_along_backbone ILOOP:Y rgb:0,255,0
#=GF R2R_oneseq Par_sR204 tick_label ILOOPL:y 3' guide sequence
#=GF R2R_oneseq Par_sR204 shade_along_backbone ILOOP:X rgb:0,255,0
#=GF R2R_oneseq Par_sR204 tick_label ILOOPL:x 5' guide sequence
#=GF R2R_oneseq Par_sR204 shade_along_backbone ILOOP:Z rgb:200,200,200

#=GF R2R_oneseq Pca_sR204 shade_along_backbone KTURN:T rgb:0,129,255
#=GF R2R_oneseq Pca_sR204 shade_along_backbone KTURN:R rgb:0,129,255
#=GF R2R_oneseq Pca_sR204 tick_label KTURNL:r K-turn
#=GF R2R_oneseq Pca_sR204 shade_along_backbone ILOOP:Y rgb:0,255,0
#=GF R2R_oneseq Pca_sR204 tick_label ILOOPL:y 3' guide sequence
#=GF R2R_oneseq Pca_sR204 shade_along_backbone ILOOP:X rgb:0,255,0
#=GF R2R_oneseq Pca_sR204 tick_label ILOOPL:x 5' guide sequence
#=GF R2R_oneseq Pca_sR204 shade_along_backbone ILOOP:Z rgb:200,200,200

#=GF R2R_oneseq Pis_sR204 shade_along_backbone KTURN:T rgb:0,129,255
#=GF R2R_oneseq Pis_sR204 shade_along_backbone KTURN:R rgb:0,129,255
#=GF R2R_oneseq Pis_sR204 tick_label KTURNL:r K-turn
#=GF R2R_oneseq Pis_sR204 shade_along_backbone ILOOP:Y rgb:0,255,0
#=GF R2R_oneseq Pis_sR204 tick_label ILOOPL:y 3' guide sequence
#=GF R2R_oneseq Pis_sR204 shade_along_backbone ILOOP:X rgb:0,255,0
#=GF R2R_oneseq Pis_sR204 tick_label ILOOPL:x 5' guide sequence
#=GF R2R_oneseq Pis_sR204 shade_along_backbone ILOOP:Z rgb:200,200,200

#=GF R2R_oneseq Pae_sR205 shade_along_backbone KTURN:T rgb:0,129,255
#=GF R2R_oneseq Pae_sR205 shade_along_backbone KTURN:R rgb:0,129,255
#=GF R2R_oneseq Pae_sR205 tick_label KTURNL:r K-turn
#=GF R2R_oneseq Pae_sR205 shade_along_backbone ILOOP:Y rgb:0,255,0
#=GF R2R_oneseq Pae_sR205 tick_label ILOOPL:y 3' guide sequence
#=GF R2R_oneseq Pae_sR205 shade_along_backbone ILOOP:X rgb:0,255,0
#=GF R2R_oneseq Pae_sR205 tick_label ILOOPL:x 5' guide sequence
#=GF R2R_oneseq Pae_sR205 shade_along_backbone ILOOP:Z rgb:200,200,200

#=GF R2R_oneseq Psp1860_sR205 shade_along_backbone KTURN:T rgb:0,129,255
#=GF R2R_oneseq Psp1860_sR205 shade_along_backbone KTURN:R rgb:0,129,255
#=GF R2R_oneseq Psp1860_sR205 tick_label KTURNL:r K-turn
#=GF R2R_oneseq Psp1860_sR205 shade_along_backbone ILOOP:Y rgb:0,255,0
#=GF R2R_oneseq Psp1860_sR205 tick_label ILOOPL:y 3' guide sequence
#=GF R2R_oneseq Psp1860_sR205 shade_along_backbone ILOOP:X rgb:0,255,0
#=GF R2R_oneseq Psp1860_sR205 tick_label ILOOPL:x 5' guide sequence
#=GF R2R_oneseq Psp1860_sR205 shade_along_backbone ILOOP:Z rgb:200,200,200

#=GF R2R_oneseq Pog_sR205 shade_along_backbone KTURN:T rgb:0,129,255
#=GF R2R_oneseq Pog_sR205 shade_along_backbone KTURN:R rgb:0,129,255
```

```
#=GF R2R_oneseq Pog_sR205 tick_label KTURNL:r K-turn
#=GF R2R_oneseq Pog_sR205 shade_along_backbone ILOOP:Y rgb:0,255,0
#=GF R2R_oneseq Pog_sR205 tick_label ILOOPL:y 3' guide sequence
#=GF R2R_oneseq Pog_sR205 shade_along_backbone ILOOP:X rgb:0,255,0
#=GF R2R_oneseq Pog_sR205 tick_label ILOOPL:x 5' guide sequence
#=GF R2R_oneseq Pog_sR205 shade_along_backbone ILOOP:Z rgb:200,200,200

#=GF R2R_oneseq Par_sR205 shade_along_backbone KTURN:T rgb:0,129,255
#=GF R2R_oneseq Par_sR205 shade_along_backbone KTURN:R rgb:0,129,255
#=GF R2R_oneseq Par_sR205 tick_label KTURNL:r K-turn
#=GF R2R_oneseq Par_sR205 shade_along_backbone ILOOP:Y rgb:0,255,0
#=GF R2R_oneseq Par_sR205 tick_label ILOOPL:y 3' guide sequence
#=GF R2R_oneseq Par_sR205 shade_along_backbone ILOOP:X rgb:0,255,0
#=GF R2R_oneseq Par_sR205 tick_label ILOOPL:x 5' guide sequence
#=GF R2R_oneseq Par_sR205 shade_along_backbone ILOOP:Z rgb:200,200,200

#=GF R2R_oneseq Pca_sR205 shade_along_backbone KTURN:T rgb:0,129,255
#=GF R2R_oneseq Pca_sR205 shade_along_backbone KTURN:R rgb:0,129,255
#=GF R2R_oneseq Pca_sR205 tick_label KTURNL:r K-turn
#=GF R2R_oneseq Pca_sR205 shade_along_backbone ILOOP:Y rgb:0,255,0
#=GF R2R_oneseq Pca_sR205 tick_label ILOOPL:y 3' guide sequence
#=GF R2R_oneseq Pca_sR205 shade_along_backbone ILOOP:X rgb:0,255,0
#=GF R2R_oneseq Pca_sR205 tick_label ILOOPL:x 5' guide sequence
#=GF R2R_oneseq Pca_sR205 shade_along_backbone ILOOP:Z rgb:200,200,200

#=GF R2R_oneseq Pis_sR205 shade_along_backbone KTURN:T rgb:0,129,255
#=GF R2R_oneseq Pis_sR205 shade_along_backbone KTURN:R rgb:0,129,255
#=GF R2R_oneseq Pis_sR205 tick_label KTURNL:r K-turn
#=GF R2R_oneseq Pis_sR205 shade_along_backbone ILOOP:Y rgb:0,255,0
#=GF R2R_oneseq Pis_sR205 tick_label ILOOPL:y 3' guide sequence
#=GF R2R_oneseq Pis_sR205 shade_along_backbone ILOOP:X rgb:0,255,0
#=GF R2R_oneseq Pis_sR205 tick_label ILOOPL:x 5' guide sequence
#=GF R2R_oneseq Pis_sR205 shade_along_backbone ILOOP:Z rgb:200,200,200

#=GF R2R_oneseq Pae_sR206 shade_along_backbone KTURN:T rgb:0,129,255
#=GF R2R_oneseq Pae_sR206 shade_along_backbone KTURN:R rgb:0,129,255
#=GF R2R_oneseq Pae_sR206 tick_label KTURNL:r K-turn
#=GF R2R_oneseq Pae_sR206 shade_along_backbone ILOOP:Y rgb:0,255,0
#=GF R2R_oneseq Pae_sR206 tick_label ILOOPL:y 3' guide sequence
#=GF R2R_oneseq Pae_sR206 shade_along_backbone ILOOP:X rgb:0,255,0
#=GF R2R_oneseq Pae_sR206 tick_label ILOOPL:x 5' guide sequence
#=GF R2R_oneseq Pae_sR206 shade_along_backbone ILOOP:Z rgb:200,200,200

#=GF R2R_oneseq Psp1860_sR206 shade_along_backbone KTURN:T rgb:0,129,255
#=GF R2R_oneseq Psp1860_sR206 shade_along_backbone KTURN:R rgb:0,129,255
#=GF R2R_oneseq Psp1860_sR206 tick_label KTURNL:r K-turn
#=GF R2R_oneseq Psp1860_sR206 shade_along_backbone ILOOP:Y rgb:0,255,0
#=GF R2R_oneseq Psp1860_sR206 tick_label ILOOPL:y 3' guide sequence
#=GF R2R_oneseq Psp1860_sR206 shade_along_backbone ILOOP:X rgb:0,255,0
#=GF R2R_oneseq Psp1860_sR206 tick_label ILOOPL:x 5' guide sequence
#=GF R2R_oneseq Psp1860_sR206 shade_along_backbone ILOOP:Z rgb:200,200,200

#=GF R2R_oneseq Pae_sR208 shade_along_backbone KTURN:T rgb:0,129,255
#=GF R2R_oneseq Pae_sR208 shade_along_backbone KTURN:R rgb:0,129,255
#=GF R2R_oneseq Pae_sR208 tick_label KTURNL:r K-turn
#=GF R2R_oneseq Pae_sR208 shade_along_backbone ILOOP:Y rgb:0,255,0
#=GF R2R_oneseq Pae_sR208 tick_label ILOOPL:y 3' guide sequence
#=GF R2R_oneseq Pae_sR208 shade_along_backbone ILOOP:X rgb:0,255,0
#=GF R2R_oneseq Pae_sR208 tick_label ILOOPL:x 5' guide sequence
#=GF R2R_oneseq Pae_sR208 shade_along_backbone ILOOP:Z rgb:200,200,200

#=GF R2R_oneseq Pog_sR208 shade_along_backbone KTURN:T rgb:0,129,255
#=GF R2R_oneseq Pog_sR208 shade_along_backbone KTURN:R rgb:0,129,255
#=GF R2R_oneseq Pog_sR208 tick_label KTURNL:r K-turn
#=GF R2R_oneseq Pog_sR208 shade_along_backbone ILOOP:Y rgb:0,255,0
#=GF R2R_oneseq Pog_sR208 tick_label ILOOPL:y 3' guide sequence
#=GF R2R_oneseq Pog_sR208 shade_along_backbone ILOOP:X rgb:0,255,0
#=GF R2R_oneseq Pog_sR208 tick_label ILOOPL:x 5' guide sequence
#=GF R2R_oneseq Pog_sR208 shade_along_backbone ILOOP:Z rgb:200,200,200

#=GF R2R_oneseq Par_sR208 shade_along_backbone KTURN:T rgb:0,129,255
#=GF R2R_oneseq Par_sR208 shade_along_backbone KTURN:R rgb:0,129,255
#=GF R2R_oneseq Par_sR208 tick_label KTURNL:r K-turn
#=GF R2R_oneseq Par_sR208 shade_along_backbone ILOOP:Y rgb:0,255,0
#=GF R2R_oneseq Par_sR208 tick_label ILOOPL:y 3' guide sequence
#=GF R2R_oneseq Par_sR208 shade_along_backbone ILOOP:X rgb:0,255,0
#=GF R2R_oneseq Par_sR208 tick_label ILOOPL:x 5' guide sequence
#=GF R2R_oneseq Par_sR208 shade_along_backbone ILOOP:Z rgb:200,200,200

#=GF R2R_oneseq Pae_sR209 shade_along_backbone KTURN:T rgb:0,129,255
#=GF R2R_oneseq Pae_sR209 shade_along_backbone KTURN:R rgb:0,129,255
#=GF R2R_oneseq Pae_sR209 tick_label KTURNL:r K-turn
#=GF R2R_oneseq Pae_sR209 shade_along_backbone ILOOP:Y rgb:0,255,0
#=GF R2R_oneseq Pae_sR209 tick_label ILOOPL:y 3' guide sequence
#=GF R2R_oneseq Pae_sR209 shade_along_backbone ILOOP:X rgb:0,255,0
#=GF R2R_oneseq Pae_sR209 tick_label ILOOPL:x 5' guide sequence
#=GF R2R_oneseq Pae_sR209 shade_along_backbone ILOOP:Z rgb:200,200,200

#=GF R2R_oneseq Psp1860_sR209 shade_along_backbone KTURN:T rgb:0,129,255
#=GF R2R_oneseq Psp1860_sR209 shade_along_backbone KTURN:R rgb:0,129,255
#=GF R2R_oneseq Psp1860_sR209 tick_label KTURNL:r K-turn
#=GF R2R_oneseq Psp1860_sR209 shade_along_backbone ILOOP:Y rgb:0,255,0
#=GF R2R_oneseq Psp1860_sR209 tick_label ILOOPL:y 3' guide sequence
#=GF R2R_oneseq Psp1860_sR209 shade_along_backbone ILOOP:X rgb:0,255,0
#=GF R2R_oneseq Psp1860_sR209 tick_label ILOOPL:x 5' guide sequence
#=GF R2R_oneseq Psp1860_sR209 shade_along_backbone ILOOP:Z rgb:200,200,200

#=GF R2R_oneseq Pog_sR209 shade_along_backbone KTURN:T rgb:0,129,255
#=GF R2R_oneseq Pog_sR209 shade_along_backbone KTURN:R rgb:0,129,255
#=GF R2R_oneseq Pog_sR209 tick_label KTURNL:r K-turn
#=GF R2R_oneseq Pog_sR209 shade_along_backbone ILOOP:Y rgb:0,255,0
#=GF R2R_oneseq Pog_sR209 tick_label ILOOPL:y 3' guide sequence
#=GF R2R_oneseq Pog_sR209 shade_along_backbone ILOOP:X rgb:0,255,0
#=GF R2R_oneseq Pog_sR209 tick_label ILOOPL:x 5' guide sequence
#=GF R2R_oneseq Pog_sR209 shade_along_backbone ILOOP:Z rgb:200,200,200

#=GF R2R_oneseq Par_sR209 shade_along_backbone KTURN:T rgb:0,129,255
#=GF R2R_oneseq Par_sR209 shade_along_backbone KTURN:R rgb:0,129,255
#=GF R2R_oneseq Par_sR209 tick_label KTURNL:r K-turn
#=GF R2R_oneseq Par_sR209 shade_along_backbone ILOOP:Y rgb:0,255,0
#=GF R2R_oneseq Par_sR209 tick_label ILOOPL:y 3' guide sequence
```

```
#=GF R2R_oneseq Par_sR209 shade_along_backbone ILOOP:X rgb:0,255,0
#=GF R2R_oneseq Par_sR209 tick_label ILOOPL:x 5’ guide sequence
#=GF R2R_oneseq Par_sR209 shade_along_backbone ILOOP:Z rgb:200,200,200

#=GF R2R_oneseq Pca_sR209 shade_along_backbone KTURN:T rgb:0,129,255
#=GF R2R_oneseq Pca_sR209 shade_along_backbone KTURN:R rgb:0,129,255
#=GF R2R_oneseq Pca_sR209 tick_label KTURNL:r K-turn
#=GF R2R_oneseq Pca_sR209 shade_along_backbone ILOOP:Y rgb:0,255,0
#=GF R2R_oneseq Pca_sR209 tick_label ILOOPL:y 3’ guide sequence
#=GF R2R_oneseq Pca_sR209 shade_along_backbone ILOOP:X rgb:0,255,0
#=GF R2R_oneseq Pca_sR209 tick_label ILOOPL:x 5’ guide sequence
#=GF R2R_oneseq Pca_sR209 shade_along_backbone ILOOP:Z rgb:200,200,200

#=GF R2R_oneseq Pis_sR209 shade_along_backbone KTURN:T rgb:0,129,255
#=GF R2R_oneseq Pis_sR209 shade_along_backbone KTURN:R rgb:0,129,255
#=GF R2R_oneseq Pis_sR209 tick_label KTURNL:r K-turn
#=GF R2R_oneseq Pis_sR209 shade_along_backbone ILOOP:Y rgb:0,255,0
#=GF R2R_oneseq Pis_sR209 tick_label ILOOPL:y 3’ guide sequence
#=GF R2R_oneseq Pis_sR209 shade_along_backbone ILOOP:X rgb:0,255,0
#=GF R2R_oneseq Pis_sR209 tick_label ILOOPL:x 5’ guide sequence
#=GF R2R_oneseq Pis_sR209 shade_along_backbone ILOOP:Z rgb:200,200,200

#=GF R2R_oneseq Pae_sR210 shade_along_backbone KTURN:T rgb:0,129,255
#=GF R2R_oneseq Pae_sR210 shade_along_backbone KTURN:R rgb:0,129,255
#=GF R2R_oneseq Pae_sR210 tick_label KTURNL:r K-turn
#=GF R2R_oneseq Pae_sR210 shade_along_backbone ILOOP:Y rgb:0,255,0
#=GF R2R_oneseq Pae_sR210 tick_label ILOOPL:y 3’ guide sequence
#=GF R2R_oneseq Pae_sR210 shade_along_backbone ILOOP:X rgb:0,255,0
#=GF R2R_oneseq Pae_sR210 tick_label ILOOPL:x 5’ guide sequence
#=GF R2R_oneseq Pae_sR210 shade_along_backbone ILOOP:Z rgb:200,200,200

#=GF R2R_oneseq Psp1860_sR210 shade_along_backbone KTURN:T rgb:0,129,255
#=GF R2R_oneseq Psp1860_sR210 shade_along_backbone KTURN:R rgb:0,129,255
#=GF R2R_oneseq Psp1860_sR210 tick_label KTURNL:r K-turn
#=GF R2R_oneseq Psp1860_sR210 shade_along_backbone ILOOP:Y rgb:0,255,0
#=GF R2R_oneseq Psp1860_sR210 tick_label ILOOPL:y 3’ guide sequence
#=GF R2R_oneseq Psp1860_sR210 shade_along_backbone ILOOP:X rgb:0,255,0
#=GF R2R_oneseq Psp1860_sR210 tick_label ILOOPL:x 5’ guide sequence
#=GF R2R_oneseq Psp1860_sR210 shade_along_backbone ILOOP:Z rgb:200,200,200

#=GF R2R_oneseq Pis_sR210 shade_along_backbone KTURN:T rgb:0,129,255
#=GF R2R_oneseq Pis_sR210 shade_along_backbone KTURN:R rgb:0,129,255
#=GF R2R_oneseq Pis_sR210 tick_label KTURNL:r K-turn
#=GF R2R_oneseq Pis_sR210 shade_along_backbone ILOOP:Y rgb:0,255,0
#=GF R2R_oneseq Pis_sR210 tick_label ILOOPL:y 3’ guide sequence
#=GF R2R_oneseq Pis_sR210 shade_along_backbone ILOOP:X rgb:0,255,0
#=GF R2R_oneseq Pis_sR210 tick_label ILOOPL:x 5’ guide sequence
#=GF R2R_oneseq Pis_sR210 shade_along_backbone ILOOP:Z rgb:200,200,200

//
```

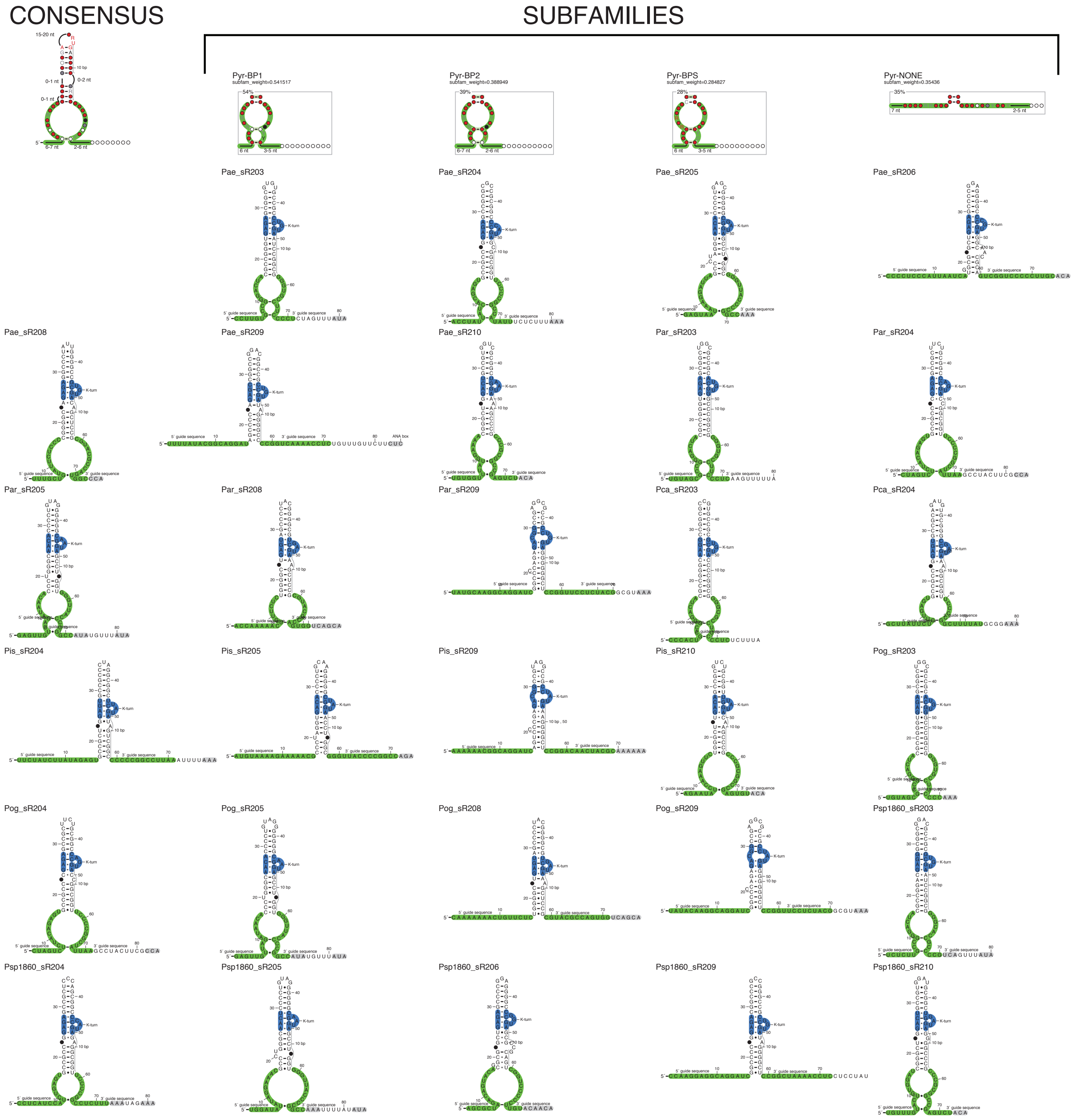

Figure S22. Full List of H/ACA-like Motifs in *Pyrobaculum* from sR201 to sR206 and sR208 to sR210.

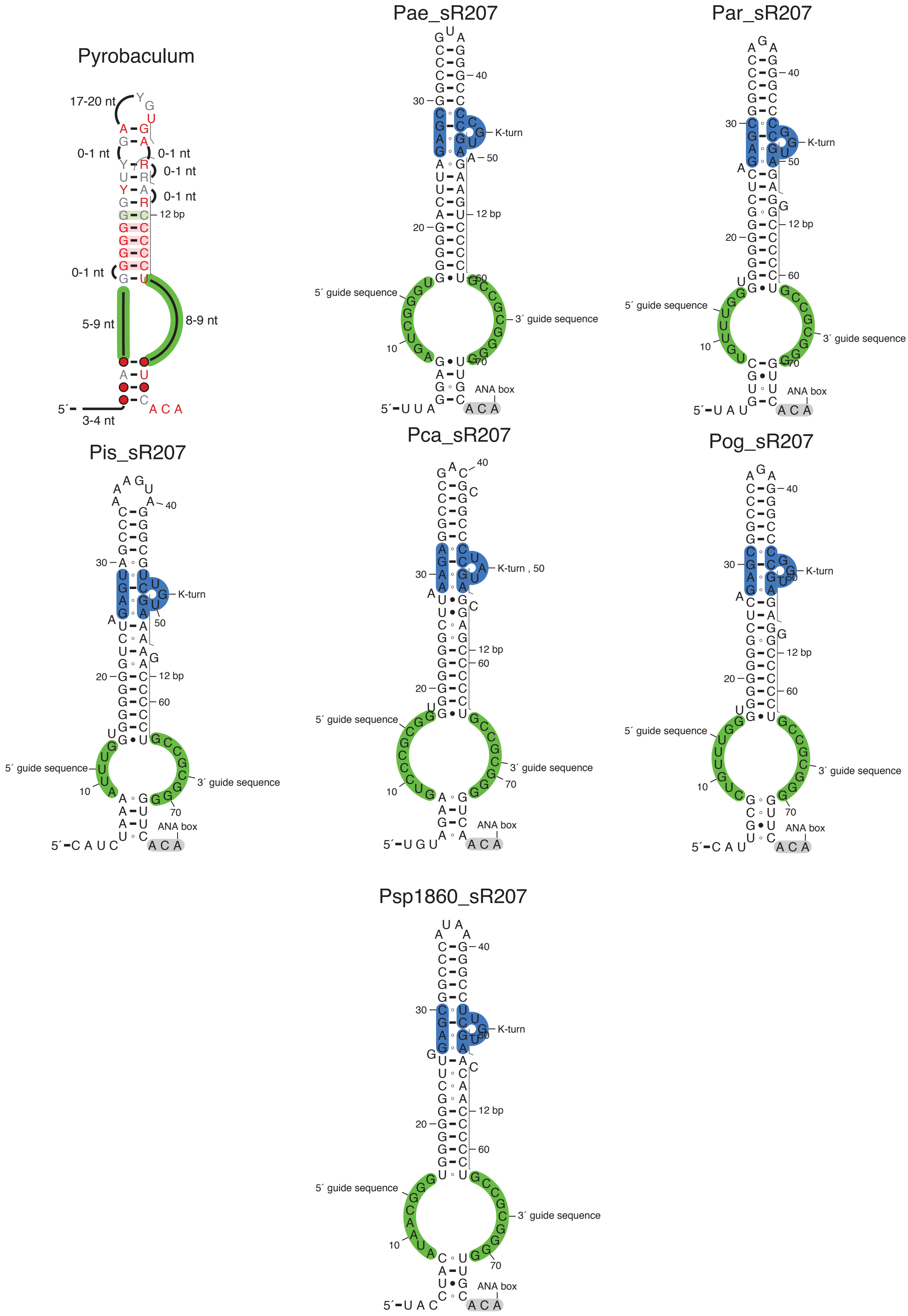

Figure S23. Full List of H/ACA-like Motifs in *Pyrobaculum* from sR207 compatible with the 12/(13)- pairing model to target L2597.

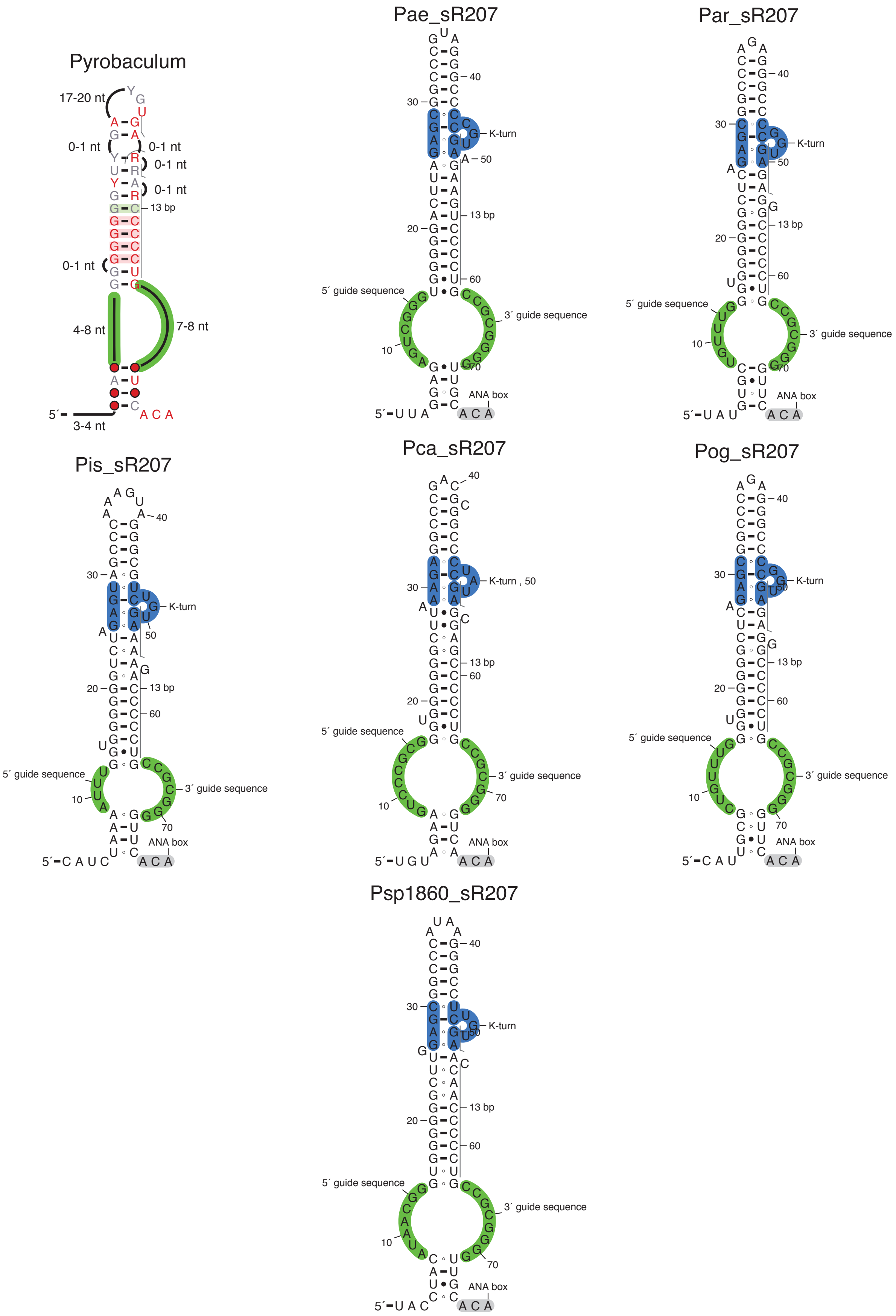

Figure S24. Full List of H/ACA-like Motifs in *Pyrobaculum* from sR207 compatible with the 13/(12)- pairing model to target L2596.

Listing 37: Stockholm Alignment of H/ACA-like sRNA (sR207) in *Pyrocabulum*.

```
# STOCKHOLM 1.0

Pae_sR207b                UUA..GGAGAG......UCGGGUG.GGGGA.CUUA.GA....G.CGGCCC...GUA.....G.GGCC.C.C..CGU.GAAGA.AGU..CCCCU.GCCGCGGGGUUGC...ACA
Psp1860_sR207b            UAC..CUACAU......AACGGGU.GGGGG.GCUUGGA....G.CGGCCC...AUAA....G.GGCC.U.C..UGU.GAACC.AAC..CCCCU.GCCGCGGGGUUGC...ACA
Pog_sR207b                CAU..UGCGCU......GUUUGGGUGGGGGGCUCA.GA....G.CGGCCC...AGA.....G.GGCC.C.C..GGU.GA.GAGGGC..CCCCU.GCCGC.GGGGUUC...ACA
Par_sR207b                UAU..GUGCUG.......UUUGGGUGGGGGGCUCA.GA....G.CGGCCC...AGA.....G.GGCC.C.C..GGU.GA.GAGGGC..CCCCU.GCCGC.GGGGUUC...ACA
Pca_sR207b                UGU..AGAAGUC.....CCGCGGGUGGGGGGCUUA.AA....G.AGGCCC...GAC.....GCGGCC.C.C..UAU.GA.GCGAGC..CCCCU.GCCGC.GGGGUCA...ACA
Pis_sR207b                CAUC.UAAAAU.........UUGGUGGGGGGU.CUAGA....G.UAGCCC...AAAGUA..G.GGCG.U.C..UGU.GA.AAAGAC..CCCCU.GCCGC.GGGGUUC...ACA
#=GC SS_cons              .....<<<<.............<-<<<<<-<-<..<<....<.<<<<<<___________>.>>>>.>.>......>>....>>>..>>>>>..........>>>>......
#=GC R2R_LABEL            1...2q...3............4.a.....b.c.deII5....p.................p........6.....HH0..9HHi..HHHHH.7.......8...r......
#=GC R2R_XLABEL_KTURN     ..................................TTTTTTTTT......................RRRRRRRRRRR.................................
#=GC R2R_XLABEL_KTURNL    ......................................................................r.....................................
#=GC R2R_XLABEL_ILOOP     ........PPPPPPPQXXXXXXW.................................................................YYYYYYYYSU.V.....ZZZ
#=GC R2R_XLABEL_ILOOPL    ...................x..w.........................................................................y..su.v.......z

#=GF R2R keep q r
#=GF R2R var_backbone_range_size_fake_nucs 3 1 2
#=GF R2R var_backbone_range_size_fake_nucs 5 3 4
#=GF R2R var_backbone_range_size_fake_nucs 8 7 8
#=GF R2R var_backbone_range_size_fake_nucs 3 0 9
#=GF R2R var_backbone_range_size_fake_nucs 1 a a
#=GF R2R var_backbone_range_size_fake_nucs 1 b b
#=GF R2R var_backbone_range_size_fake_nucs 1 c c
#=GF R2R var_backbone_range_size_fake_nucs 2 d e
#=GF R2R var_backbone_range 5 6
#=GF R2R outline_nuc H
#=GF R2R outline_nuc i
#=GF R2R tick_label i 10 bp
#=GF R2R shade_along_backbone 3 rgb:0,255,0
#=GF R2R shade_along_backbone 7 rgb:0,255,0

#=GF R2R_oneseq Pae_sR207b shade_along_backbone KTURN:T rgb:0,129,255
#=GF R2R_oneseq Pae_sR207b shade_along_backbone KTURN:R rgb:0,129,255
#=GF R2R_oneseq Pae_sR207b tick_label KTURNL:r K-turn
#=GF R2R_oneseq Pae_sR207b shade_along_backbone ILOOP:Y rgb:0,255,0
#=GF R2R_oneseq Pae_sR207b shade_along_backbone ILOOP:S rgb:0,255,0
#=GF R2R_oneseq Pae_sR207b shade_along_backbone ILOOP:U rgb:0,255,0
#=GF R2R_oneseq Pae_sR207b tick_label ILOOPL:y 3' guide sequence
#=GF R2R_oneseq Pae_sR207b shade_along_backbone ILOOP:P rgb:0,255,0
#=GF R2R_oneseq Pae_sR207b shade_along_backbone ILOOP:Q rgb:0,255,0
#=GF R2R_oneseq Pae_sR207b shade_along_backbone ILOOP:X rgb:0,255,0
#=GF R2R_oneseq Pae_sR207b tick_label ILOOPL:x 5' guide sequence
#=GF R2R_oneseq Pae_sR207b shade_along_backbone ILOOP:Z rgb:200,200,200
#=GF R2R_oneseq Pae_sR207b tick_label ILOOPL:z ANA box
#=GF R2R_oneseq Pae_sR207b var_backbone_range_size_fake_nucs 1 j j 0-1 nt

#=GF R2R_oneseq Psp1860_sR207b shade_along_backbone KTURN:T rgb:0,129,255
#=GF R2R_oneseq Psp1860_sR207b shade_along_backbone KTURN:R rgb:0,129,255
#=GF R2R_oneseq Psp1860_sR207b tick_label KTURNL:r K-turn
#=GF R2R_oneseq Psp1860_sR207b shade_along_backbone ILOOP:Y rgb:0,255,0
#=GF R2R_oneseq Psp1860_sR207b shade_along_backbone ILOOP:S rgb:0,255,0
#=GF R2R_oneseq Psp1860_sR207b shade_along_backbone ILOOP:U rgb:0,255,0
#=GF R2R_oneseq Psp1860_sR207b tick_label ILOOPL:y 3' guide sequence
#=GF R2R_oneseq Psp1860_sR207b shade_along_backbone ILOOP:P rgb:0,255,0
#=GF R2R_oneseq Psp1860_sR207b shade_along_backbone ILOOP:Q rgb:0,255,0
#=GF R2R_oneseq Psp1860_sR207b shade_along_backbone ILOOP:X rgb:0,255,0
#=GF R2R_oneseq Psp1860_sR207b tick_label ILOOPL:x 5' guide sequence
#=GF R2R_oneseq Psp1860_sR207b shade_along_backbone ILOOP:Z rgb:200,200,200
#=GF R2R_oneseq Psp1860_sR207b tick_label ILOOPL:z ANA box

#=GF R2R_oneseq Pog_sR207b shade_along_backbone KTURN:T rgb:0,129,255
#=GF R2R_oneseq Pog_sR207b shade_along_backbone KTURN:R rgb:0,129,255
#=GF R2R_oneseq Pog_sR207b tick_label KTURNL:r K-turn
#=GF R2R_oneseq Pog_sR207b shade_along_backbone ILOOP:Y rgb:0,255,0
#=GF R2R_oneseq Pog_sR207b shade_along_backbone ILOOP:S rgb:0,255,0
#=GF R2R_oneseq Pog_sR207b shade_along_backbone ILOOP:U rgb:0,255,0
#=GF R2R_oneseq Pog_sR207b tick_label ILOOPL:y 3' guide sequence
#=GF R2R_oneseq Pog_sR207b shade_along_backbone ILOOP:P rgb:0,255,0
#=GF R2R_oneseq Pog_sR207b shade_along_backbone ILOOP:Q rgb:0,255,0
#=GF R2R_oneseq Pog_sR207b shade_along_backbone ILOOP:X rgb:0,255,0
#=GF R2R_oneseq Pog_sR207b tick_label ILOOPL:x 5' guide sequence
#=GF R2R_oneseq Pog_sR207b shade_along_backbone ILOOP:Z rgb:200,200,200
#=GF R2R_oneseq Pog_sR207b tick_label ILOOPL:z ANA box
#=GF R2R_oneseq Pog_sR207b var_backbone_range_size_fake_nucs 1 j j 0-1 nt

#=GF R2R_oneseq Par_sR207b shade_along_backbone KTURN:T rgb:0,129,255
#=GF R2R_oneseq Par_sR207b shade_along_backbone KTURN:R rgb:0,129,255
#=GF R2R_oneseq Par_sR207b tick_label KTURNL:r K-turn
#=GF R2R_oneseq Par_sR207b shade_along_backbone ILOOP:Y rgb:0,255,0
#=GF R2R_oneseq Par_sR207b shade_along_backbone ILOOP:S rgb:0,255,0
#=GF R2R_oneseq Par_sR207b shade_along_backbone ILOOP:U rgb:0,255,0
#=GF R2R_oneseq Par_sR207b tick_label ILOOPL:y 3' guide sequence
#=GF R2R_oneseq Par_sR207b shade_along_backbone ILOOP:P rgb:0,255,0
#=GF R2R_oneseq Par_sR207b shade_along_backbone ILOOP:Q rgb:0,255,0
#=GF R2R_oneseq Par_sR207b shade_along_backbone ILOOP:X rgb:0,255,0
#=GF R2R_oneseq Par_sR207b tick_label ILOOPL:x 5' guide sequence
#=GF R2R_oneseq Par_sR207b shade_along_backbone ILOOP:Z rgb:200,200,200
#=GF R2R_oneseq Par_sR207b tick_label ILOOPL:z ANA box
#=GF R2R_oneseq Par_sR207b var_backbone_range_size_fake_nucs 1 j j 0-1 nt

#=GF R2R_oneseq Pca_sR207b shade_along_backbone KTURN:T rgb:0,129,255
#=GF R2R_oneseq Pca_sR207b shade_along_backbone KTURN:R rgb:0,129,255
#=GF R2R_oneseq Pca_sR207b tick_label KTURNL:r K-turn
#=GF R2R_oneseq Pca_sR207b shade_along_backbone ILOOP:Y rgb:0,255,0
#=GF R2R_oneseq Pca_sR207b shade_along_backbone ILOOP:S rgb:0,255,0
#=GF R2R_oneseq Pca_sR207b shade_along_backbone ILOOP:U rgb:0,255,0
#=GF R2R_oneseq Pca_sR207b tick_label ILOOPL:y 3' guide sequence
#=GF R2R_oneseq Pca_sR207b shade_along_backbone ILOOP:P rgb:0,255,0
#=GF R2R_oneseq Pca_sR207b shade_along_backbone ILOOP:Q rgb:0,255,0
#=GF R2R_oneseq Pca_sR207b shade_along_backbone ILOOP:X rgb:0,255,0
#=GF R2R_oneseq Pca_sR207b tick_label ILOOPL:x 5' guide sequence
#=GF R2R_oneseq Pca_sR207b shade_along_backbone ILOOP:Z rgb:200,200,200
#=GF R2R_oneseq Pca_sR207b tick_label ILOOPL:z ANA box
#=GF R2R_oneseq Pca_sR207b var_backbone_range_size_fake_nucs 1 j j 0-1 nt

#=GF R2R_oneseq Pis_sR207b shade_along_backbone KTURN:T rgb:0,129,255
#=GF R2R_oneseq Pis_sR207b shade_along_backbone KTURN:R rgb:0,129,255
#=GF R2R_oneseq Pis_sR207b tick_label KTURNL:r K-turn
#=GF R2R_oneseq Pis_sR207b shade_along_backbone ILOOP:Y rgb:0,255,0
```

```
#=GF R2R_oneseq Pis_sR207b shade_along_backbone ILOOP:S rgb:0,255,0
#=GF R2R_oneseq Pis_sR207b shade_along_backbone ILOOP:U rgb:0,255,0
#=GF R2R_oneseq Pis_sR207b tick_label ILOOPL:y 3’ guide sequence
#=GF R2R_oneseq Pis_sR207b shade_along_backbone ILOOP:P rgb:0,255,0
#=GF R2R_oneseq Pis_sR207b shade_along_backbone ILOOP:Q rgb:0,255,0
#=GF R2R_oneseq Pis_sR207b shade_along_backbone ILOOP:X rgb:0,255,0
#=GF R2R_oneseq Pis_sR207b tick_label ILOOPL:x 5’ guide sequence
#=GF R2R_oneseq Pis_sR207b shade_along_backbone ILOOP:Z rgb:200,200,200
#=GF R2R_oneseq Pis_sR207b tick_label ILOOPL:z ANA box
#=GF R2R_oneseq Pis_sR207b var_backbone_range_size_fake_nucs 1 j j 0-1 nt

//
```

Table S2. Functional and structural features from known H/ACA(-like) guide RNAs in *Pyrobaculum* targeting alternative positions in rRNAs and tRNAs.

guide:target pairs	*conservation*[a]	*expression*[b]	*promoter*[a]	*% GC content*	*pseudouridylation*[b]	*structure-function model*[c]	*hybrid energy*[d]	*duplex energy*[e]	*5' duplex energy*[e]
Pae201-S1724[1]	+	+	+	73	ND	10/6/8+	-26	-16	-8.2
Pae201-tR38[2]	+	+	+	73	ND	10/6/8+	-26	-11	-5.9
Pae204-S1452[1,3]	+	+	+	63	ND	10/(14)+	-35	-5.4	-7.2
Pae204-L13[2,4]	+	+	+	63	ND	10/(14)+	-35	-12	-5.2
Pae205-S762[2]	+	+	+	53	ND	10/(14)+	-32	-6.4	-2.3
Pae205-S1395[4]	+	+	+	53	ND	10/(14)+	-32	-5.4	-8.2
Pae206-S395[5]	+	+	+	65	ND	10/(15)+	NC	NC	NC
Pae207-S582[1,2]	+	+	+	67	ND	12/(13)-	-22	-14	-2.8
Pae207-S1192[6,7]	+	+	+	67	ND	12/(13)-	-23	-26	-12
Pae208-L965[2,4,8]	+	+	+	59	ND	10/(14)+	-22	-4.6	-0.7
Pae208-L1307[2,4,6]	+	+	+	59	ND	10/(14)+	-22	-13	-2.4
Pae209-L2801[3]	+	+	+	59	ND	10/(14)+	-25	-13	-10

ND: no experimental evidence. NC: no calculation possible using RNAsnoop (too weak contraints). Target annotations: S: small subunit of the ribosome (16S); L: large subunit of the ribosome (23S); tR: tRNA. tR38: position U38 of tRNA-Pro(CGG) (position U39 in the genomic sequence; genomic positions: 320,578-320,651). [a] as provided by the UCSC genome browser (41, 42). [b] as determined from high-throughput pyrosequencing (expression) and experimental validation of RNA targets (productive) (16). [c] structure-function model as proposed from the 'productive'/'non-productive' classification; the +/- sign indicates whether it is predicted to be productive or not. [d] as calculated by RNAsnoop (kcal/mol) from the Vienna RNA package (44). [e] the duplex energy includes both the 5' and 3' duplex energies with a correction factor (+4.1 kcal/mol).

List of base-pairing anomalies next to the targeted U position:
[1] 1nt bulge on 3' duplex.
[2] 5' duplex below the energy cutoff of -6 kcal/mol.
[3] 1 mismatch on 3' duplex.
[4] 1nt bulge on 5' duplex.
[5] 3nt bulge on 5' duplex.
[6] 1 mismatch on 5' duplex.
[7] 3nt target spacer.
[8] 2 mismatches on 5' duplex.

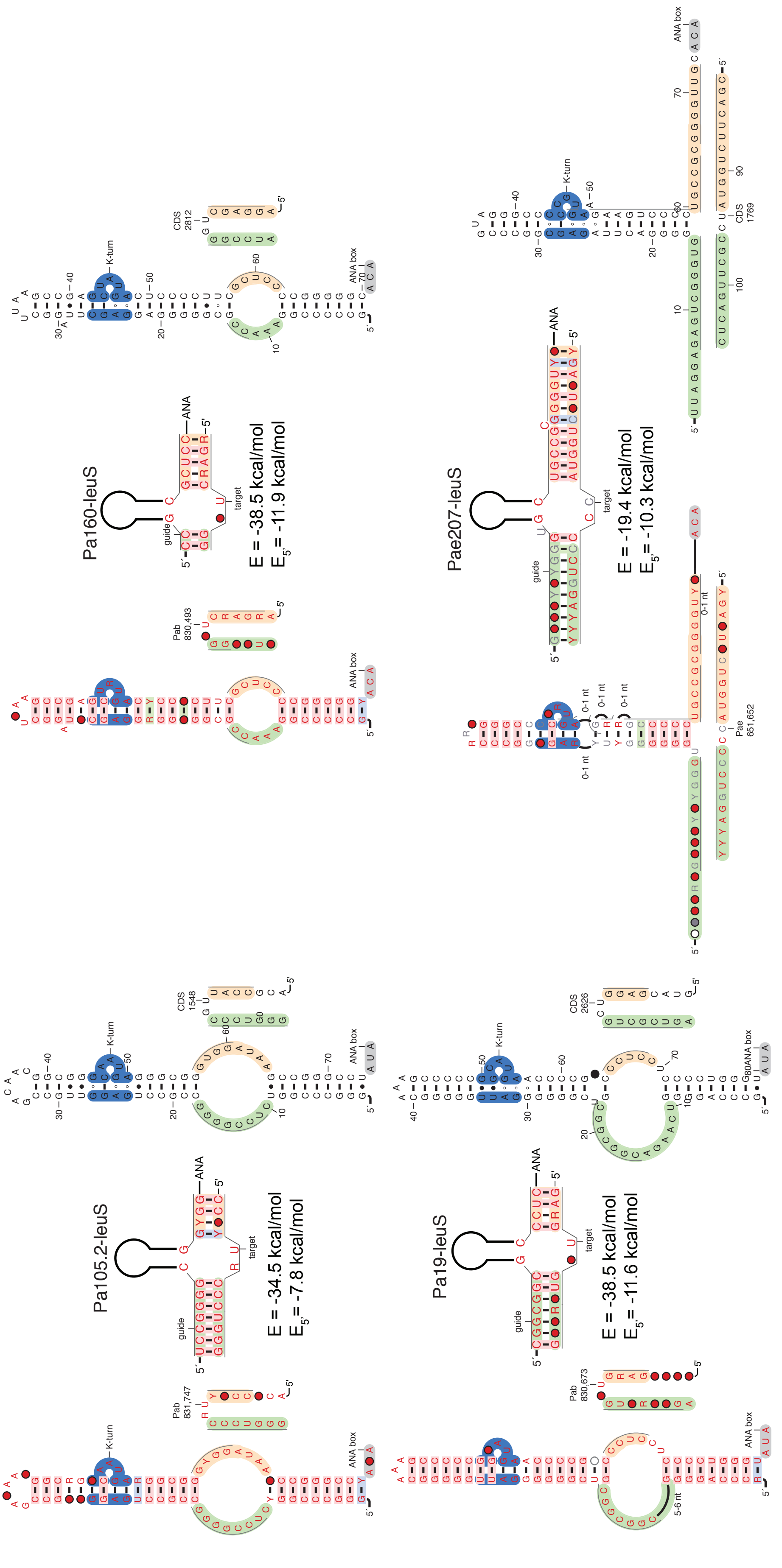

Figure S25. Leucyl-tRNA synthetase as potential extra-ribosomal target of H/ACA guide RNAs: Pab105.2, Pab19, Pab160 (*Pyrococcus*), and Pae207 (*Pyrobaculum*). The consensus structure is built from the sequences of three Pyrococcus: *P. abyssi*, *P. horikoshii* and *P. furiosus* and five Pyrobaculum: *P. aerophilum*, *P. calidifontis*, *P. arsenaticum*, *P. islandicum* and *P. oguniense*. The energies are calculated using the same method. The target positions are indicated with the coordinate in the genome and in the CDS.

Table S3. Helical Parameters of the Lower Stem in the 65 and 66 subfamilies of H/ACA guide RNAs.

	H/ACA subfamily 65 (PDB ID: 3LWO)	H/ACA subfamily 66 (PDB ID: 3HAX)
total bend(°)	7.4	10.9
total h-Rise(Å)	21.6	20.9
local h-Rise(Å)	2.51	2.32
total h-Twist(°)	274	259
local h-Twist(°)	43.1	30.8

the total values are given for the whole stem, the local values for the junction between the stem and the internal loop; the helical parameters of the two guide RNAs co-crystallized with a target in the RNP particle (PDB IDs: 3LWO (19) and 3HAX (18)) are calculated using X3DNA (version 2.1) (47) and Curves+ (48).

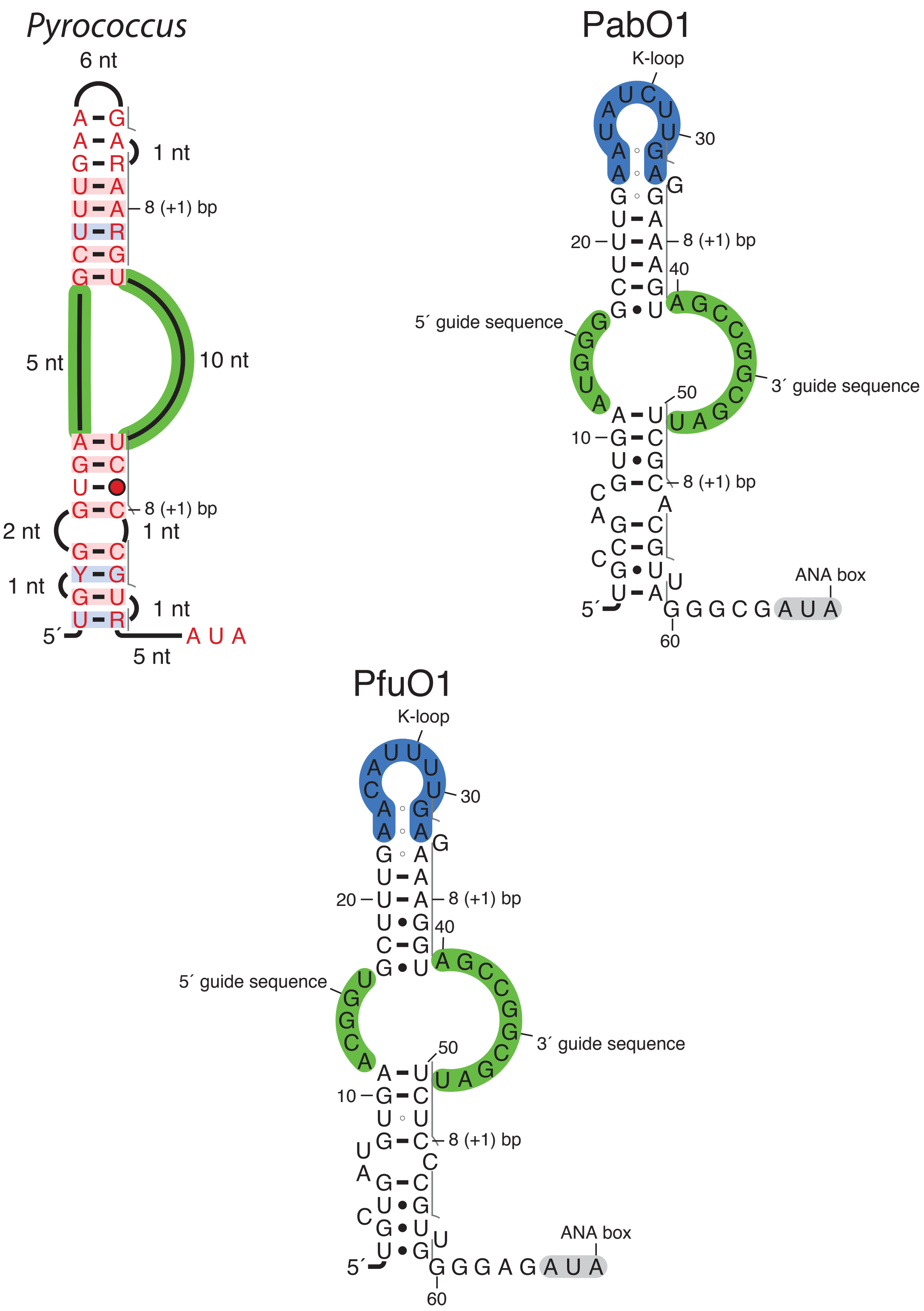

Figure S26. PabO1 H/ACA Motif from *Pyrococcus*. Pab: *P. abyssi*, Pho: *P. horikoshii*.

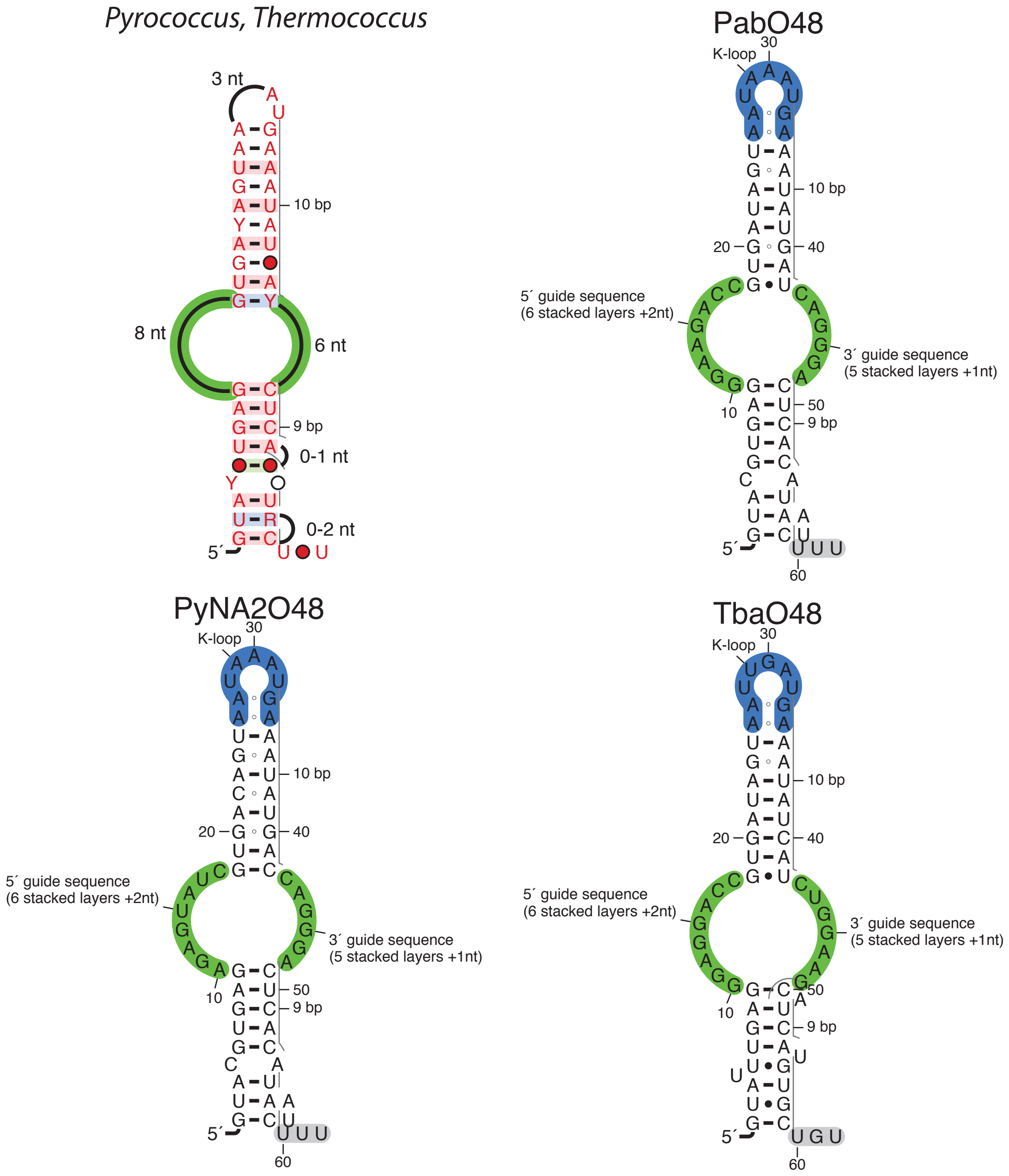

Figure S27. PabO48 H/ACA Motif from *Pyrococcus* and *Thermococcus*. Pab: *P. abyssi*, PyNA: *P. sp. NA2*, Tba: *T. barophilus*.

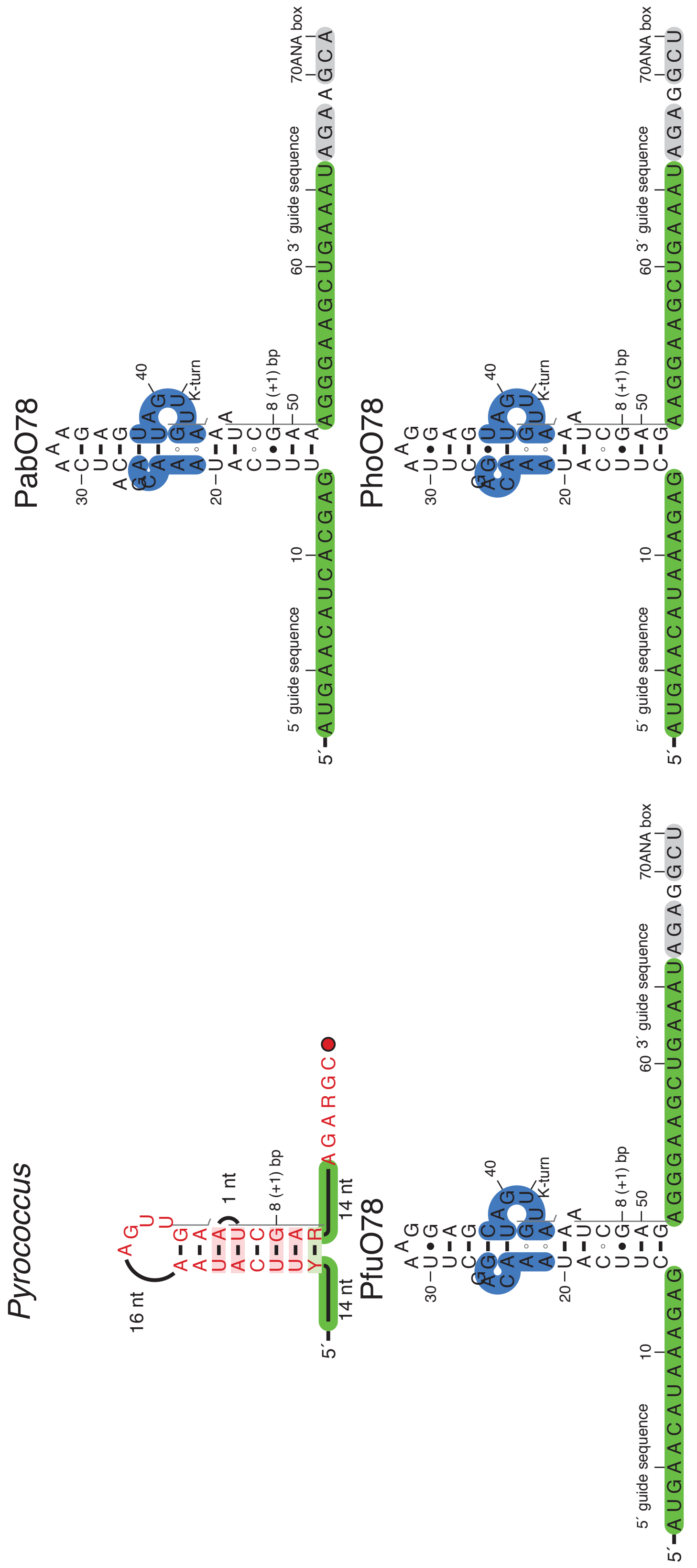

Figure S28. PabO78 H/ACA-like Motif from *Pyrococcus*. Pab: *P. abyssi*, Pho: *P. horikoshii*, Pfu: *P. furiosus*.

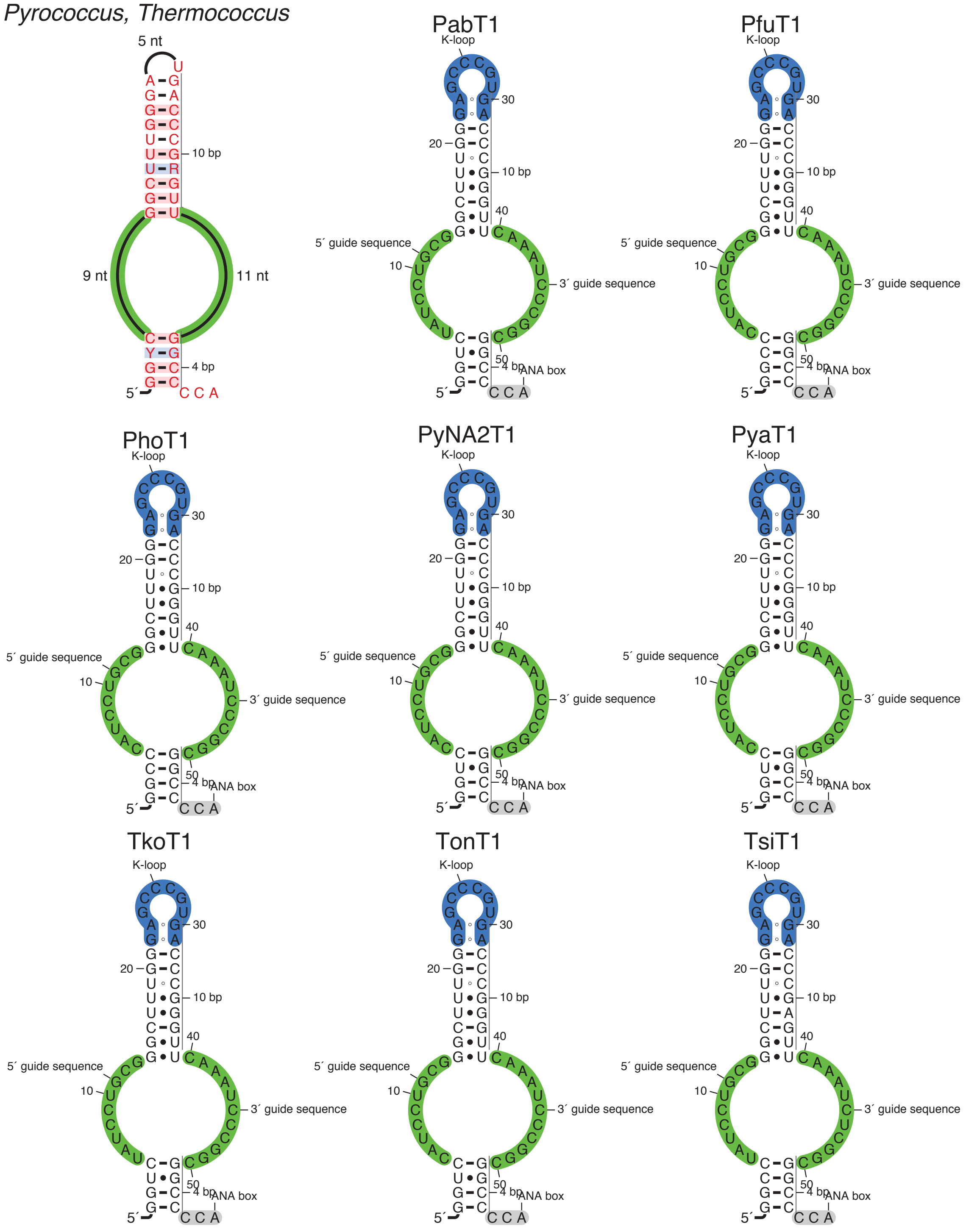

Figure S29. PabT1 H/ACA Motif from *Pyrococcus* and *Thermococcus*. Pab: *P. abyssi*, Pho: *P. horikoshii*, Pfu: *P. furiosus*, PyNA: *P. sp. NA2*, Pya: *P. yayanosii*, Tko: *T. kodakarensis*, Ton: *T. onnurineus*, Tsi: *T. sibiricus*.

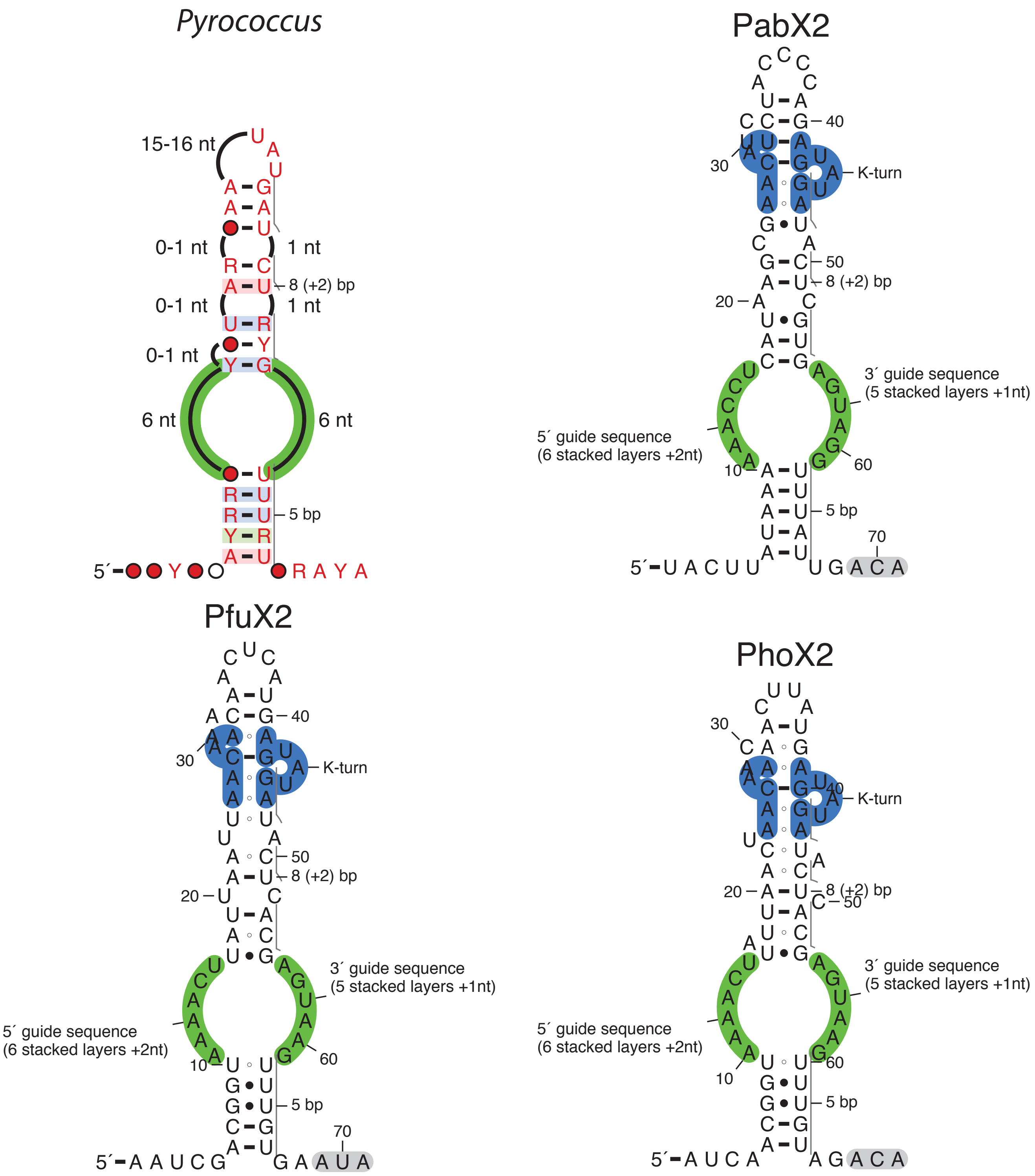

Figure S30. PabX2 H/ACA-like Motif from *Pyrococcus*. Pab: *P. abyssi*, Pho: *P. horikoshii*, Pfu: *P. furiosus*.

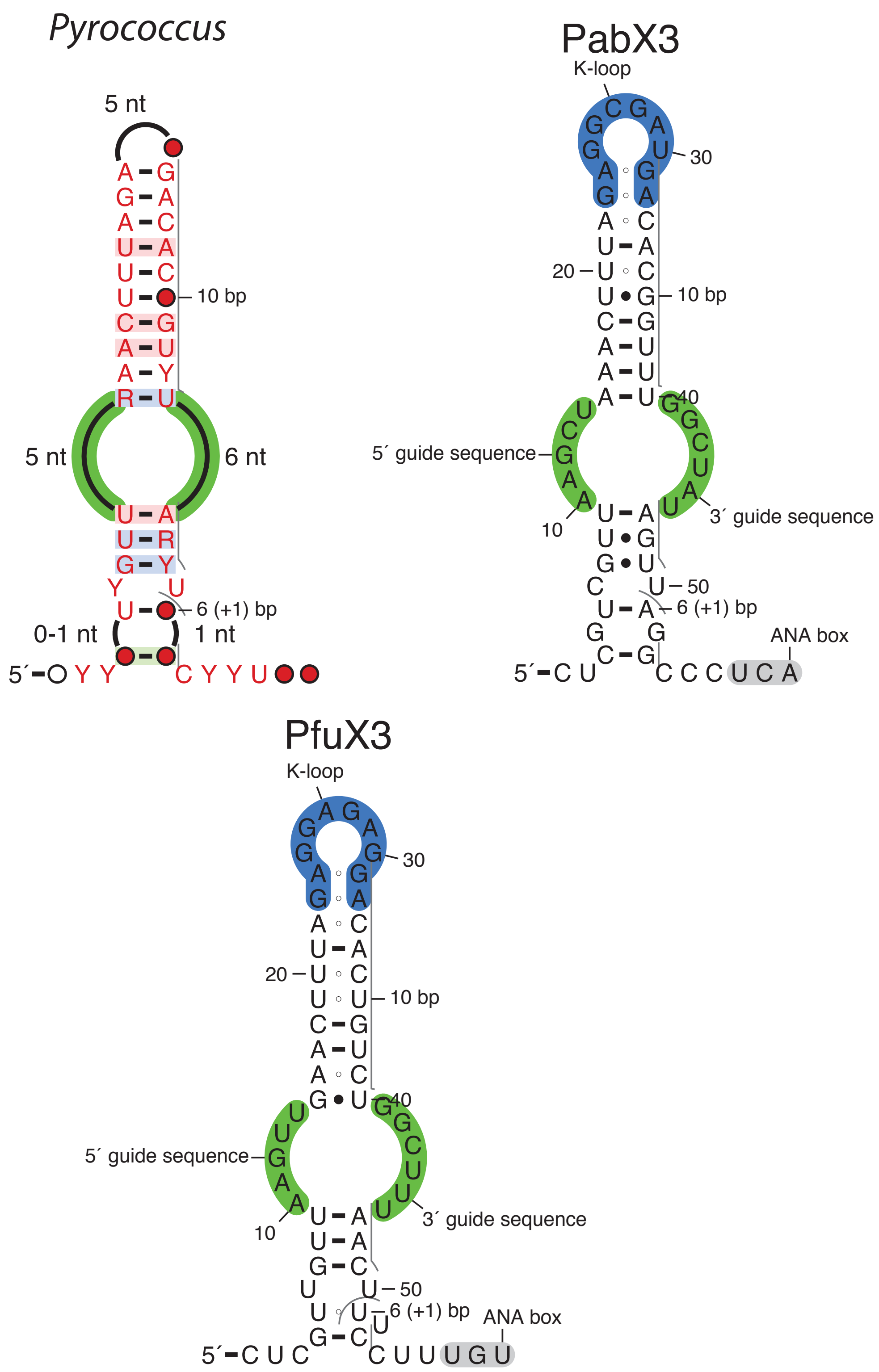

Figure S31. PabX3 H/ACA-like Motif from *Pyrococcus*. Pab: *P. abyssi*, Pfu: *P. furiosus*.

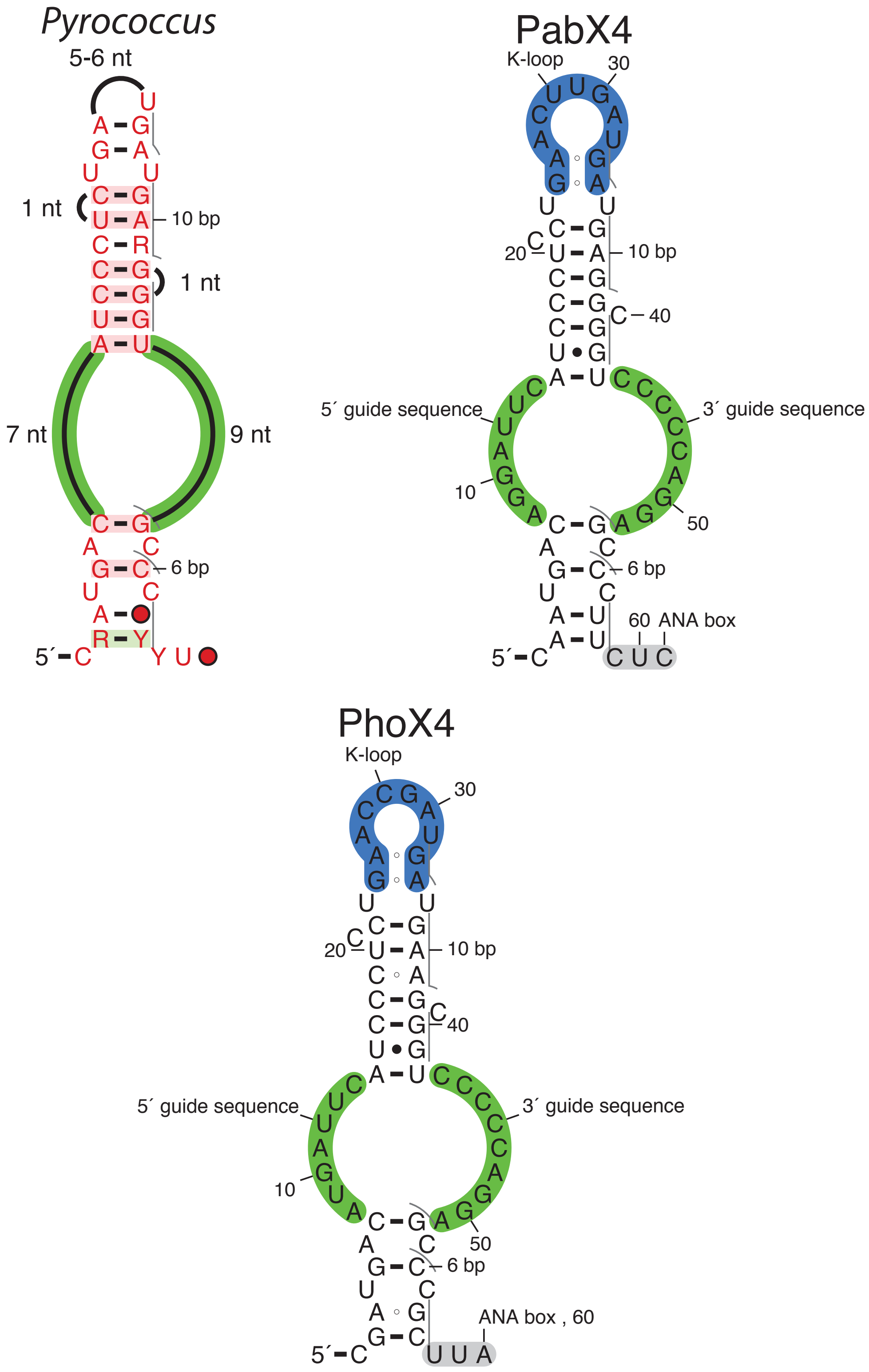

Figure S32. PabX4 H/ACA-like Motif from *Pyrococcus*. Pab: *P. abyssi*, Pho: *P. horikoshii*.

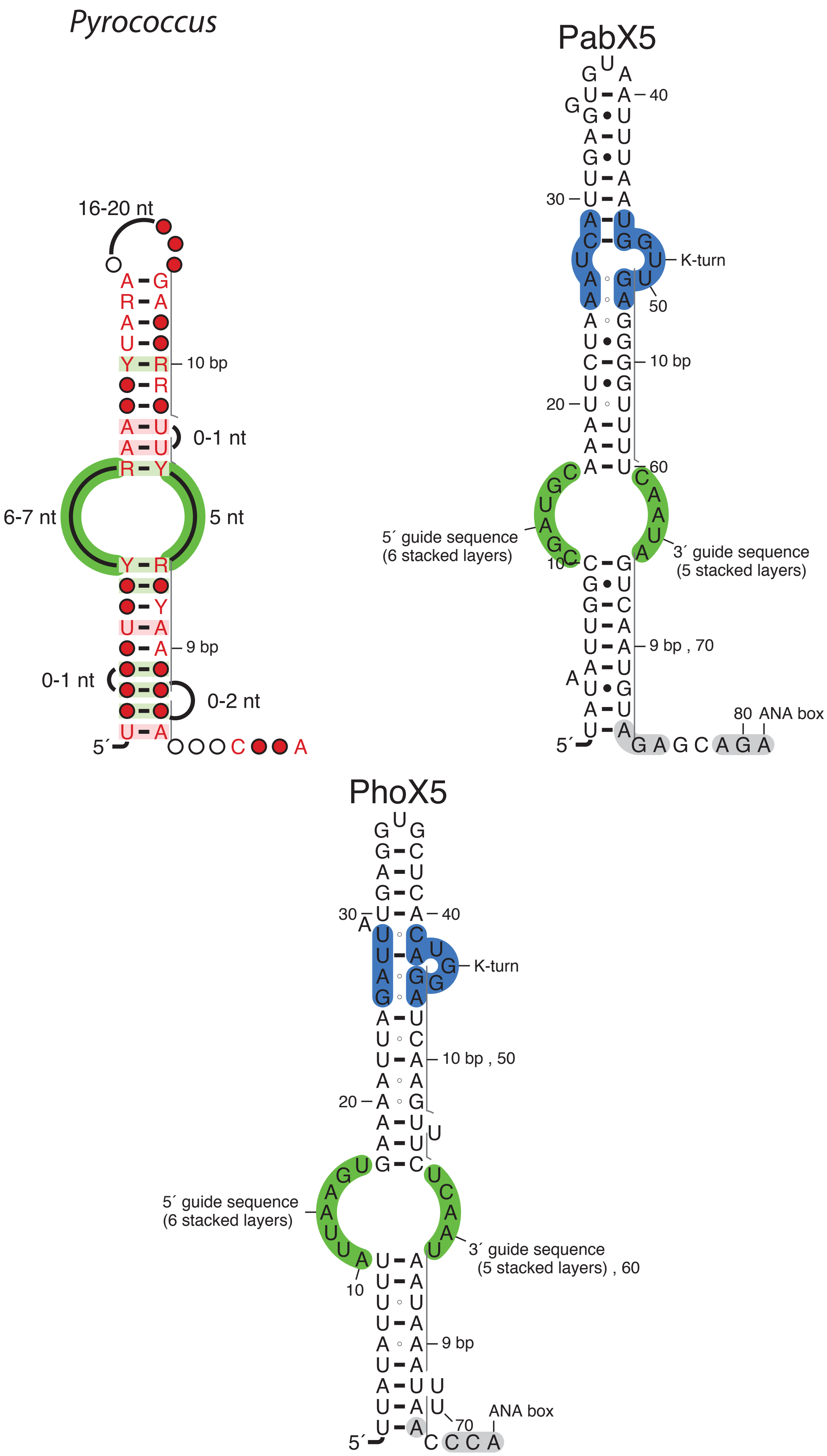

Figure S33. PabX5 H/ACA Motif from *Pyrococcus*. Pab: *P. abyssi*, Pho: *P. horikoshii*.

Table S4. Functional and structural features of new H/ACA(-like) motifs in *Pyrococcus*.

guide	conservation[a]	expression[b]	promoter[b]	% GC content	structure-function model[d]
PabO1	+	+	+	51	9/10/9-
PabT1	+	+	+	69	10/11/4+
PabO48	+	+	-	38	10/5-6/9+
PabO78	+	-	+	37	9/14-
PabX2	+	-	-	38	10/6/7-
PabX3	+	-	-	48	10/6/9+
PabX4	+	+	+	58	10/9/6+
PabX5	+	-	-	35	10/5/9+

[a] as provided by the UCSC genome browser (41, 42).
[b] as determined from RNA-seq data (36).
